# Supplementary figures and images for: Genome-wide characterization, evolutionary analysis of WRKY genes in Cucurbitaceae species and assessment of its roles in resisting to powdery mildew disease
Source: PLoS One. 2018 Dec 27;13(12):e0199851. doi: 10.1371/journal.pone.0199851 (PMC6307730; doi:10.1371/journal.pone.0199851)

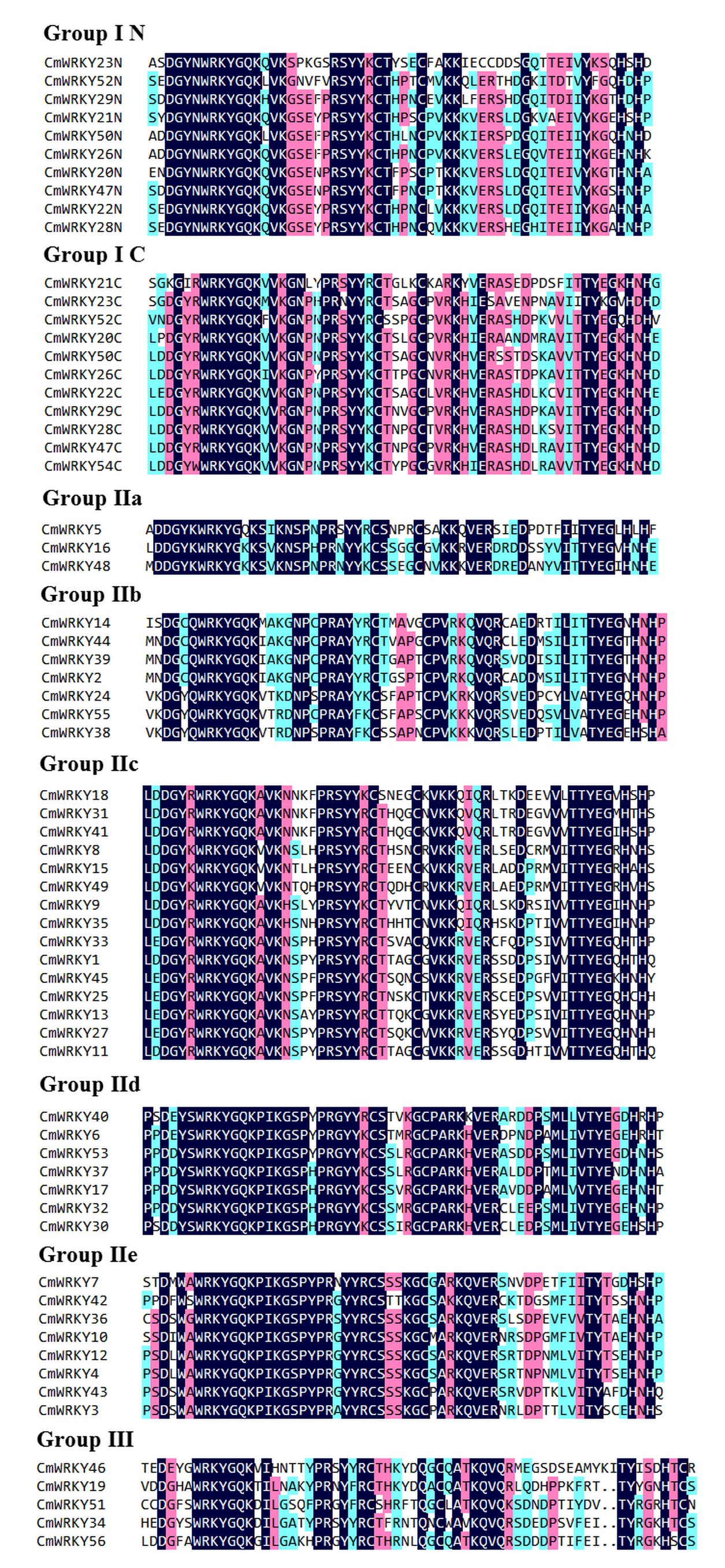

Supplement: S1 Fig — Highly conserved amino acids in WRKY domain are shown in black. (TIFF) [file pone.0199851.s001.tiff]

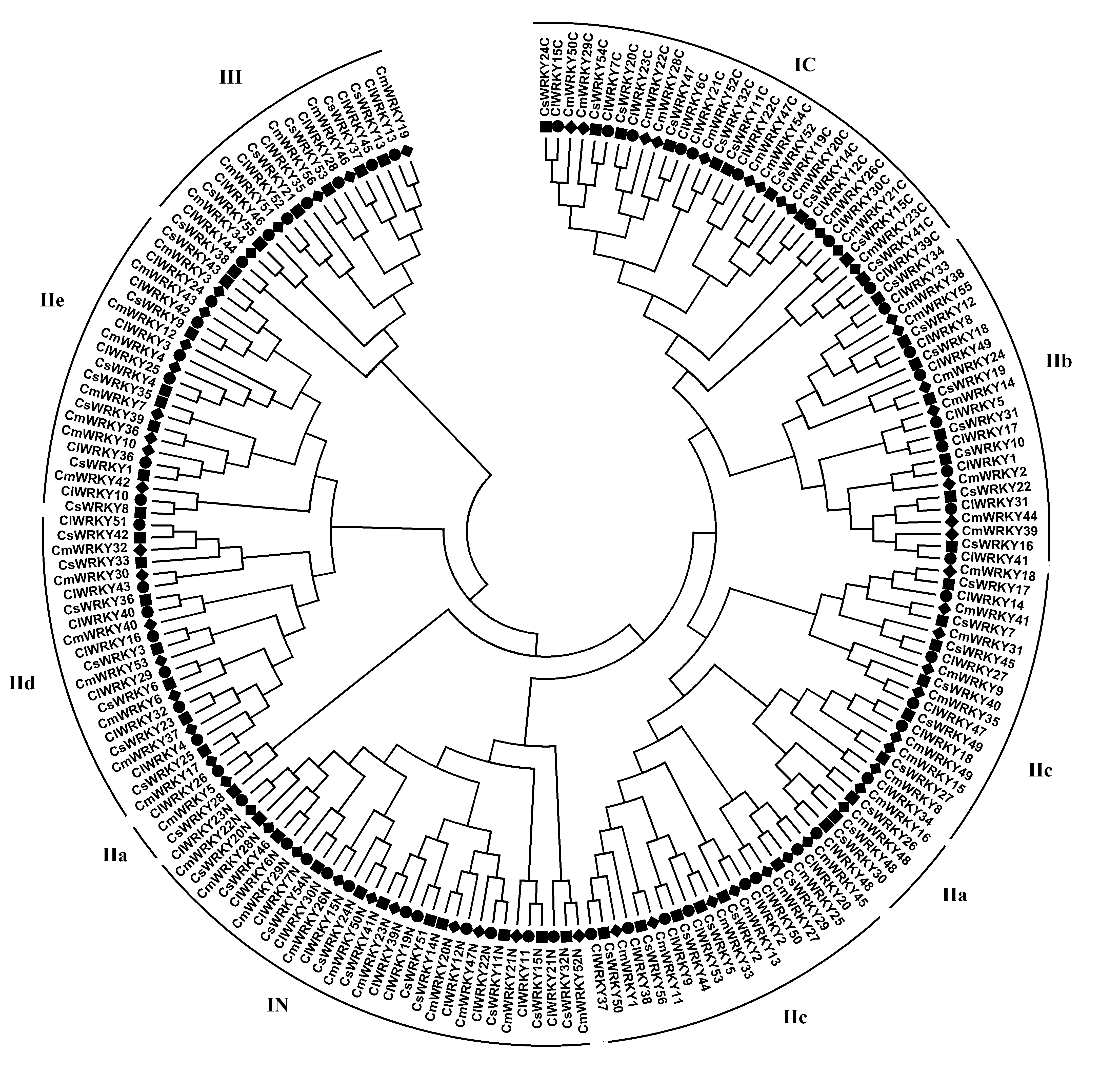

Supplement: S2 Fig — The domains were clustered into three major groups I, II, III, and five subgroups (a, b, c, d, and e) in group II. (TIF) [file pone.0199851.s002.tif]
