# Supplementary material for: Genome-wide characterization, evolutionary analysis of WRKY genes in Cucurbitaceae species and assessment of its roles in resisting to powdery mildew disease
Source: PLoS One. 2018 Dec 27;13(12):e0199851. doi: 10.1371/journal.pone.0199851 (PMC6307730; doi:10.1371/journal.pone.0199851)
Supplement: S2 Table — (DOCX) [file pone.0199851.s004.docx]

**The nucleotide sequences of all identified WRKY families** **in three cucurbitaceae species melon, cucumber and watermelon**

>CmWRKY1

ATGTATTTTGTATGGAGGAAGAGAAGAAGGAAGAAGATTTTGGGAAATAATAGAGATCAGAATTCGATGGGAAATCCTGGATTGTTATTCTCCGATGCGATTATTCCCAATAATCATAATAATAATTTGTTCGATTTTCCATTAATCGACTCCATTGATTCTTCTTTCAAGCCTTCCACTTTCTTGGATTTGTTAGCTACTCAAGATTATACCCCTTCTTTGTTCGATCTTTTCTCTCCTCCGCCTCCGCCGCCGCAACCCCCGCTTCCTCCGCCATCCTCCGCCGTACCGGAGTCTTCTGAAGTTCTTAATACTCCTCCCACTCCTAACTCCTCCTCCGTCTCTTGCTCCTCCACCGAACGTGCTTTCGACAACGATGACGTGGATCGGGATAAGTCCCCTTTCAACAAACAATTGAAAGCAAAAAAGAACCAAAAAAAAAATGGAAGAGAACCAAGATTTGCGTTCATGACAAAAAGCGAAGTGGATCACTTAGACGACGGTTACAGATGGCGAAAATACGGTCAAAAAGCCGTTAAAAACAGCCCTTACCCAAGAAGTTATTACCGTTGCACCACCGCCGGCTGCGGCGTCAAAAAACGAGTCGAACGATCTTCCGACGACCCTTCCATCGTCGTCACCACTTACGAAGGCCAACACACTCATCAAAGTCCAATCATGCCACGTGGAGCTTTATCCTCCACCGCCTACACCGCCTCCCCACAACAACACCAACAACAACCGCCGCTTGTTTTCTCATCACAGCCGCAACAATTATACCGCAACCAATTTACGTACGCTCCGGCGCCGCCCGTGGACGTTGTCACGTGCGGCGGAGGGTTCGGTCACGTGTTTCACAGTTTTGGAGAAGAACGACGACGCATCGATGATAGAACTACACCTGATTCTTTCCAAGACCATGGCCTTCTTCAAGATATGATCGTACCGTTCCCAGAAGAAGAAAAAAAAGTTAATTGA

>CmWRKY2

ATGGAGGCTGCTGCTGCTTTTGGCCGTCCAAGGCCCGTTGTCAAGACCGAGAAACCTCCCGTTCGCGACGTTAGTCATGACGACGATCGGGATTCTCCCTCTAAACAACAACAACAACAACAACATCTCCTCGTTAAGAGAGCTGGGAACAACCATGCAAAACAAGAACATGATACTGAAGATAAAACAAGTTGTTCATCTGATAAAAAGGATTTGAGCTGCATCAAACTGCAGGAAGATCAATTGGAATCTGCTCGAGCAGAAATGGGGGAAGTAAGAGAAGAAAACCAAAGACTAAAACAAAGTTTAAACCAAATCATGAAGGATTATGAAGCTTTGAAAATGCAATTCCTAGGGATTGTCGGACGAGACTCTAAGAAATTACAAGACGACGACAACGATGTGAATAAGGAACAACAACAACAACAACAACACGATGACGATCAAATCGAACTGGTTTCACTTTCTTTGGGGAGGTTTCCGGTGTCGGAGAAGATCAAAAAAGTAGCTGATGAGAAAAGCTCTATGAATATCATCATAGGCGGTGGTGACCAGGACGAAGAAGCCGCGTGTAAAGAGGCTTTATCTCTCGGTTTGAACTGCAAATTCGAACGGGAAGAATCGATGGTGGCTGTCGTTAAAGAAGTCGATTCTCCAAATAGTTTTGATCATGAGGCGACGAAGGAGGAAGCTGGAGAGACAAATTGGCCATCTAAAGGGGGGAAGACAATGAGAAGTGTTGAAGATGATGTTACACCGCAAAACCCACCCAAACGCGCTAGAGTTTGCGTTAGAGCCCGATGTGAAACCGCCACGATGAACGATGGTTGCCAATGGAGGAAATATGGGCAAAAGATAGCAAAAGGAAACCCATGTCCACGAGCATATTATCGTTGCACAGGTTCACCTACATGTCCGGTAAGAAAACAAGTCCAAAGATGCGCTGACGACATGTCGATTTTGATCACCACCTATGAAGGTAATCACAACCACCCATTACCTGCCTCTGCCAACGCCATGGCCTCTACCACCTCCGCCGCTGCCTCCATGCTTCTCTCTGGCTCCACCACCTCCGCAACGACGGCGGCTTCGTCATCCACCGCATCGAATACTCTCCACGGCCTAAACTTTTACGCAAATAATTCTAAACCTAATTTTTACTTACCCAATAATAGTTCCTCTATAATTTCATCCACTTCCCCAACTCACCCCACAATCACTTTGGACCTCACTTCAAATCCTTCTTCCTCTTCTTCAAATTCCTCAACCCATTTTGGTAAGTTCACATCTAACTTTCCCAATTCTCGTTACCCTTTCACTGGCCAACTCGATTTTGGATCTTCTAGAAACAATGTGTTGTCGTGGAATAATGGTCTTCTTAGTTACAACAGAAATAACCACCCTACTACTACTACAACTGCCAACAATATCTATCAAAACTACATCCAACAACAACAACGAAACCCCACCACCTCACTACAACATCAGCAACCGCCTTTACCCGACACCATTGCTGCCGCTACCAAGGCCATCACGGCCGATCCGAGCTTTCAATCTGCTCTTGCCGCTGCACTTACATCGATCATTGGCACCGGGGGTGCTAGTGCAAGTGCTGGCCTCACCAAGTCATCGTCGGGAAGAGGCGAACAATCGTTGTTTCAATTGATGACGACGACGGCTACTACGAATAAAGGAAATGGATGTGGGACGAGCTTTTTGAACAACATTACGACGACCACCACCACCACCACGAGTAATTCACCACCAACAGGGAATATGGTGTTTGTTCCAACGAATTCAAAAAGTGCGTCGGCTTCTCCAGGTGATCATATTGATCTTACCCATTAA

>CmWRKY3

ATGGATGACGTTTCCTTCAACCATAATAACCACCACCAATTCTATTCATCTGATCCATTCGAAGACTCCGACGAATTAAAACCCACCCCCGACTCTCCTGCTCCTAGTTCCACCATCGCCGCCGCCGCCACCAAAAAGGGCAGAAGAGGAATGAAGAAGAAAATTATATCAGTGAAAATTAACGGCGACAGCCCAAGAAATTCGTCCGGTTCCGCAACTCCACCTTCAGATTCATGGGCTTGGAGAAAGTATGGTCAAAAACCCATCAAAGGCTCCCCTTATCCGAGGGCGTATTATAGGTGTAGTAGTTCAAAGGGATGTCCAGCGAGGAAGCAAGTAGAGAGGAATCGGCTTGATCCAACCACGTTGGTCATTACATATTCGTGTGAGCATAATCATTCCGGTCCGGTTTCTAGAAACAACAACAACAACAATAATCAGAATAACCAAATCGTTTTGATGAAGCCCGGTTCACCAGAGACGGTTGCGGTTCATCAAGAACCGGAAGTGGAAGAGAAGTTTGTGGAGATTGGAGGAGAGGAGTCGTTGATTACGGCGGATGAGTTTAGTTGGTTTGGGGAAATGGAAACGACGTCGTCTACAGTACTCGAAAGCTCCATTTTTTCCGGCAGAGCGTCCACCGGATTGGTGGATCATTCGATTTCTAGTGACGTGGCGATGCTTTTTCCAATGGGGGATGATGACGTGGACGAGTCACTCTTTGCGGATCTCGGGGAGTTGCCGGAGTGTTCACTCGTGTTCCGGCGCGGCGGAGGTCGTGGATTACCGGTGGATGAACAACCGGCGGCAGCCCAGCGGCGGATCACGCCTTGGTGTGGCACCACCACATGA

>CmWRKY4

ATGGACAATAATAATTATCAGCAAGCTGGTGATTTGACTGATGTGATTCGACCGACTTCCGCCCCCGTCGCCGGTCATTTCTCCTCCGAATTCTCTCTCGACTCGTTTTCCGGTGAGGGACGACTCTGGTCGCACCTTCCTGCTGACAATTCTTCCATGAATTTTGGAGATCCGTTGTCGTTTCAGACTCGAGATCCATTTCTTCTTCCCCATTTTTCTCCCTTCAACTTTGCTTCTGATGGCGGCGGTGAGGGTTTGGCGGCGGACCAGCCGTCTAATTTACTTTCGCATATGTTGCAGATCTCTCCGAGTTCTGGTGATGGTGTAATTAGTACTCCTCCATGTGAGTCGCTCGCCTCAGCAGTGGGGAATTCTCCGAGCTCGACTTCAGTTCGTGGTGGCGGTGGTGTTGTGGTTCCGACCGGTGGCTCGAACCCTCTTTGTTTAATGGAGAATTCCGGGATTCAGATCTCTTCTCCACGAAATTCCGCAAATAAAAGAAGGAAGAGCCAAGTGAAGAAAGTGGTGTGTATTCCGGCACCGGCACCGGCGAACAGTCGATCAAGCAGCGGCGAAGTGGTTCCTTCTGATCTATGGGCATGGAGGAAGTACGGGCAAAAGCCAATCAAAGGATCCCCATATCCAAGGGGATATTATAGGTGTAGTAGTTCCAAGGGTTGCTCCGCTCGAAAGCAGGTTGAGCGAAGTCGTACAAACCCCAACATGCTTGTCATCACCTACACTTCGGAGCACAACCACCCATGGCCTACTCAACGCAATGCCCTTGCAGGTTCTACTCGTTCCCATCCTTCTCGAACCACCACCACTACCGCCACTAATAAAACCAGCCCGAAACAAGAGCGCATTAATGACATCCTGCCAACGACAATGATAAAGGAGGAAGAGATAGATGAAGACCAAACGACAGATAAAGCAGCGACAACAACAAATGATAACCAGGAGGAGGGTGATCATCAAGATTTCCCTTTTGACTTAATTTTCACTGAATTTGCAGATCAAATGAACGACCTTGAGCACGAGAACGACCACGACCATAGTCATCATCTTTCAGCAGACCCTCTGAACAACAATATGTTGATGTTTGGTAGCCATGGATTTAGTACAGGAAATGGAGAAGGCAGTAAAGATCCATTTTTGGAGCTCTATGACTGGGCAGAAAATTCAAGTGGAAGTTTGTTCAAAGAAGCCAAGGGAGGTTGA

>CmWRKY5

ATGGATTCTCTAGCAGCCATTCCCTGGTCAGATAGTTACACCACTGTCTCGTCCGATGACCACCTTTTCACCCTCACTAACCTTCTCCACGACGACGACAACCACGACTCCTCTCCACTCTTCCTCCTCCCCCAAGATAATGCCGACGACAATAATCGATCCATCAGGCTCCCTGTCCCCGGTGGTGCAACCTACTTTGGGCCGACAATTGAGGACATTGAGAATGCACTTTCCATTGGTACTCCTAGATCAAAAGACCTCCATTCCCACGCTCACATTTCTCATACCGGATTTTCCATTGTGGAAAGAGGGAGCTTGAATAAGGTTGAGCATAAGTATAGCCTTAGAATCAAGAGCTGTGGAGGGAATATGGTGGCTGATGATGGATATAAGTGGAGGAAATATGGTCAGAAGTCCATAAAAAATAGCCCCAATCCTAGGAGCTACTATAGGTGTTCAAATCCAAGATGCAGTGCAAAGAAGCAAGTAGAAAGGTCGATAGAAGATCCAGACACCTTCATCATAACCTACGAAGGCCTTCACCTCCACTTTGCTTATCCATTCTTCCTAATGGGCCAAAACCCACAACCCCAATCTCCAACCAAAAAGCCCAAGACAATCGACCCAGAGCCTGAAGCCCATGAAAAACCACCTTTCCTCGACCCAATAGAATCAACCGGCACACAAGGCTTGCTGGAGGACATGGTCCCATGGTTGATTCGAAATCCATCTACCCATCACAACGCTCTATCAAATTCTTCCTCTTGCTTATCCCACCGCTCACCTCCACCCACCCCTCCATCACCCTCCACGTCTCCTTCCTTCACGACTTCCTGTTTTTGA

>CmWRKY6

ATGGCGGTCGATCTGATGAGTTTTCCGAAGATGGATGATCAAATCGCTATACAAGAGGCAGCGTCGCAAGGTTTGAAGAGTATGGAGCATTTGATTCGTCTTCTTTCTCACAAGCAATCTTCAAGCCATGTCGATTGTTCTGACCTAACCGATGCAACCGTTTCCAAGTTTAAGAAAGTTATTTCTCTCCTCAATCGAACCGGTCACGCCAGATTCCGCCGAGGTCCGATTTCTTCGACTTCCTCATCGTCTTCCGGTTCATCTGCTCATCTCTCTCAAAACCAGGCCATGACTCTCACTCCTACTCCGTTTACTTCACCGCCGAATGTTCCTGCTCCGCCGTTCACCGCTCCGGCTACTATCGCTCAGCCACAGACGAAAGTCGTCGCTGCGGCCGCGAACTTTCTACCTCAGCCTCAGAGTATGACTCTCGATTTCACTAGACCGAATATTTTGAACTCCAACCCTAAGGGAACCGATTTGGAATTTTCCAAAGAGACCTTCAGTGTGTCTTCGAGCTCATCGTTCATGTCCTCTGCTATCACCGGAGATGGAAGTGTATCCAACGGAAAATTAGGAACGTCTATCTTTTTAGCACCAGCACCGACTGCTTCCGGTGGTAAGCCACCGCTCTCGGTCGCACCTTACAAGAAGAGGTGTCACGAACACGATCATTCCGAGGACTTATCCGGAAAATTCTCCGGTTCAACATCAATTTCCGGGAAGTGCCATTGCTCGAAGAGAAGAAAAAATCGGATGAAGAAGACGATCCGAGTCCCGGCTATTAGTTCAAAAATTGCCGATATTCCACCGGACGAGTACTCATGGAGGAAGTACGGTCAGAAGCCGATCAAAGGATCTCCATACCCGCGGGGGTATTATAAGTGTAGCACAATGAGGGGATGTCCGGCGAGGAAACACGTGGAGAGAGATCCGAACGATCCGGCGATGTTGATTGTAACGTACGAAGGGGAACACCGCCATACACAAAGCTCGTTACCGGAAAATATGGCCGCGGCGGCTGGAGTAGCTTTAGTTTTTGAGTCAACTTGA

>CmWRKY7

ATGGCTGCCGGAAACGACGATTGGGATCTTTCCGCCGTCGTCCGGAGTTGCAATTCAGCAGCTTCCGCTACAGACCCAACTTCCGCCGCCGCGGAATCCGCCTTGTCTTGTCTCGCTTCTTTAACATTCGACGACGATCCAGACGACGTTGCATTCTCCTTTTCCGACATCTTTCAACCCAAACAACCAAACGGCGGCTTCCATGAATTACACCAAGCCTTCGTCTCTTTTCTCCCTAACCCCTCCGCCACCGACACCACCACCACCACCACCACCACCGTCACCGCCGTACCCGTCTCCGAGCCCGAGATCCCTTACCCAACCCCCCCGAATCGCCATTTCCGTCAGGGAATCAAACCCATTCGCCCAAACCCACCTCCTGTGGCGCTGCAGCAGCACCCCCACCACCGGCAACCGCCGTTTTCGCCGGACCTCCCTAATTCGCCAATGACGCAATCTCTCATACCTAAATCGAGAAAAAGGCAAAACCAACAAAAGAGAAGGGTATGCCATGTTACGGCAGATAACCTCTCAACGGACATGTGGGCTTGGCGTAAATATGGGCAAAAGCCCATTAAAGGATCTCCGTATCCAAGGAACTATTACCGTTGCAGCAGCTCAAAAGGGTGTGGAGCAAGAAAGCAAGTTGAACGCAGCAACGTCGATCCCGAAACTTTCATCATCACATACACCGGCGACCACTCTCATCCCCGTCCAACTCACCGTAATTCCCTCGCCGGAAGTTCCCGCAACAGGTCCTCCTCCTCCTCCTCCCGACACCCCACCCCGGGAGATTTCGATCCCTCGATGACCGCCTCCGCTCTCGTACCCTCTTCCTCTTCCCCTGCAGCTTCTCCCATCACACCGCTGAACGATTACGAGGGTCCAACCGGAGAGAAAGATGGAGAGATGTTTGAAGACATGCCGATTGATAGTGACGAGGAAGAGGACGATGAGGATATTCTGATTCCGAACTTGACGGTGAGAGATGAAATCTTTGTTGGGTTTGAGGAGGTTGGGAGGGGGAGGGGGAGGTCATCGTAA

> CmWRKY8

ATGGAAACTGGAACCCAGACCCAAAGATCATCAGGAAGAGGGGAGGATTATGAAGTTCAAGTGTCGTTCCACCTCTCAAATGATCCACACCACATCCACGAAATGGGATTTGTGCAGTTGGAAGAGCACAGCCAGGTTTTGAGCTTCTTGGCACCCAACAACAACAACAACAACGCCACCAATATGCCACCTCCTCCCTTTACTACTACTTCTCCCCATTCCAACATCCTCCCTCGACCACCTTCTTGGACTAATCACCAGCTGCCTGGAACACTGGATCCCAAGCCCGGTAATGACGAAAACTGCACGGCTACTGCTACTGATGCCACCAATTCATGGTGGAGAAACACAAACGCGGATAAGAGTAAGGTGAAGGTAAGGAGAAAGCTGAGAGAACCACGTTTCTGTTTTCAGACCAGAAGCGATGTTGATGTGCTGGACGATGGCTATAAATGGAGAAAATATGGCCAAAAAGTTGTCAAGAACAGCCTTCATCCTAGAAGCTACTATCGTTGCACGCATAGCAACTGCCGAGTGAAGAAGAGGGTAGAGCGACTGTCGGAGGATTGCCGGATGGTGATAACCACTTACGAAGGTAGACACAACCACTCTCCTTGCGATGACTCCAATTCTTCGGAGCACGAACCCTTTACATCATTCTGA

>CmWRKY9

ATGGAGGAGGAACATCACCAACTACAACAACCATCGCCGCCGCCGCCTCCATGTTCGGATCCTCTTGCAACCTCTCTAGAGATCGATTGGATCGCAGTTCTCTATGGGCAAGAGGCCATCGGAGACTTACCACCAGCGTCATCAACTTGTGAGTCATCAGAAAGGAGAAGGGACGAAGAGAAAACGAATCGGAGGAAGAATGGTGGCCGACGGTGGAGAAAGGCCGCTGGCCGTCGAAGGTTTGAGTTTCAGACAAGGAGTACCGAAGATATTCTTGATGATGGATATCGGTGGCGCAAGTACGGGCAAAAAGCTGTCAAACATAGCCTCTATCCCAGGAGCTACTATAAGTGCACATATGTAACATGCAATGTGAAGAAACAAATTCAAAGGCTATCAAAAGACAGAAGCATTGTTGTGACAACTTATGAGGGAATTCACAACCATCCTTCTCATATCCTCATGCAAACCCTAACTCCTCTTCTCAAACAAATTCACACTTCCTTTCCACTTTCTAAATTATTTATGAATTATAATTAG

>CmWRKY10

ATGGAAGTCGATTGGGATCTTCATGCCGTCGTCAGAGGTTACTCCGCCGCGCCCTCTGCCGCCACCATTGTTCCTTCTTCTTCTTCTTTTTCCAATAACTCTGTCCCTTTTTCTTTTGGTAGAGACCTAACCAACAATCAAACCAAAAACCATTTCTTCTCTCTTCAAGATCCATTTCAACCCTCTAATTGTAATTCCACTCAAGAATTGCATGAGCTTTTCAAACCCTTTTTCCCTAAATCTCAACCCTCTTCTTCTTCTCCTACGCCGCCTCCTCCTCCTCCACCGCCTGCTGCTCCCCCGCTTCTTTCTTCTCCGGCACCTAAAATTTTGACTCATCACAAACAGAGCACCCATCTCCCTAAACAGCTCCATTCAACCTCTGTTTCTGCTCCGAGATCCAAACGCCGGAAGAATCAGCTGAAGAAGGTTTGTCAAGTTCCGGCGGAGTCTCTATCTTCGGATATTTGGGCTTGGCGAAAATATGGACAAAAACCCATTAAAGGATCTCCATATCCAAGGGGATATTATAGATGTAGCAGTTCGAAGGGTTGCATGGCCCGAAAACAAGTGGAGAGGAACAGATCCGACCCGGGAATGTTCATAGTCACTTACACGGCTGAGCACAACCACCCGGCGCCCACTCACCGCAACTCTCTCGCCGGCTCCACCCGTCAGAAACCCGTCACGCCGACCACCACCGCGTCCGGATCTGAAAAACTCGACCCGAAACAGCCGGTCTGCTCGTCGGAAGAACAGAGTACGATCACGGAGAGCAAAGAAGAGAAAGAGGAATTATTAATGGCCGAAGATGAAGAAGACGACGATCTGGGAGTCTCCGATTTGATCGTGAACGACGATTTCTATGTGGGTTTTGAAGAACTCGACAGCCCAATCACCGACGACTGCTTTTCCGATCAATTCCCGGCGAACTTTGACCTTCCATGGTTGTTTAACGGAAATCCAGACGGCGAAATTGTAAAACTCCCCTGTTAA

>CmWRKY11

ATGACAAAGAGTGATATTGATCATCTCGACGACGGTTACCGGTGGCGAAAGTACGGTCAAAAAGCTGTGAAGAATAGCCCTTACCCCAGAAGCTATTATCGTTGTACAACGGCAGGGTGTGGTGTGAAGAAGAGAGTAGAGCGATCGTCCGGCGACCATACGATAGTCGTTACGACGTACGAAGGCCAACACACTCACCAAAGTCCGATAATGCCGCGAGGGAGTCTCAGAGTATTACCAGAATCCACCAACAACAGCCTCACCGTCGATCACGACACCACCACCACCGGACTTTTATTCCAACACAACACTAGTTCCCAACCTTTCATGTATAGCTCTCCACCCTCACCATTTCTAACAATCAACTCGTCATCGGTGGCGGCCACGAGTACTCCCCATCCTCCTCCTCCTATTTCGTTTCAGCCACCGTCTTTGCCGCAAGCTTCCGTTCGAGACCACGGGCTTTTACAGGACTTAGTGCCATTGCAAATGAGAAATGAACCAAAAGATGAGCAAGATGGATGA

>CmWRKY12

ATGGAGAATTATGATCAAGAAGTAGGTGATTTGACTGATATTGTAAGAGGAAGATCAACAACAACAACAACAAGAAGCTCTACTACTACTACTACTACTTCTTCTTCTTCTTCTAATTGCAAGACTGAAATACTAGCTGATCATCATCTTCAAGATTCCACCTCTTTCTATTATTCTTCTTCATCACAATTGCTGCAACTACAAGATCATCAAGATCATCATCATCATCACCAATACAGTTTTGGAGATCCATTTTGCAGTGTTGTTGCTCCTTCAGTACTTGATCATCATCTTCATCAACATCATCATCATCAGCTGGTGGATAATAATACTAATATTAATGCTTTTTTCAATGGAATTTCTGCTTCTGCTGCTACTACTACTGCTGCTGCTGCTCATCAAGATCATGAAGATCATCACGAAGTTATGAAAAGCAGCCCTTGTAATTCCAATTTATTCTCACGAATGCTTCAGATCTCTCCTTCATCCAACAAATTCCAAACCATTTCTTCACTTACTAATAATTCCCCACCTCCTTCTAATTTCTTAATTTCAAATCATTCTCCTACCACTACGACTACTCCACTGCATCCTGATCATCACCTTCATCATTTTCTTCATCATCATGATCAAAATAATAATAATAATAATAATAATACCTCTGCCGCCCTTCACATCTCTTCTCCCCGGAACCCCCCCGGTATCAAGCGAAGGAAGAGTCAAGCAAGGAAGGTGGTTTGTGTTCCAGCTCCAGTTGCTGCCAGTAGTAGACCAAATGGGGAAGTGATTCCTTCTGATCTTTGGGCTTGGAGGAAATATGGTCAGAAACCCATTAAAGGTTCTCCATATCCTAGGGGGTACTATAGATGCAGCAGCTCAAAAGGATGTTCAGCAAGAAAACAAGTGGAGAGAAGTAGAACAGATCCAAACATGTTGGTGATTACTTACACATCTGAACACAACCATCCATGGCCAACTCAAAGAAATGCTCTTGCCGGCTCTTCAAGATCATCTCAGCATTCATCAAAAAACAACACTTCTACTACTACTACTACTACATCTACTACAACACAACCCAATTCTTCAAAATTACTCCATCATAAAAATAAACAAGAAGTTCAAGAAGAAGAAGAAGACCAAGATCATGAAAACAACAACAATGGCACGACGACGGTATTATTATCATCAACAGCAGCAGCAGATGAAGAAGCATCAAATAAGAATAATAATAATGTTAAGGAAGAGGAGATGATTGAGAATGAATTGATGATGAGTAGTACTAGTGAAGGAGGGTTAATTGATGAAGATCATGATTTCTTTGCAGATTTGGAAGAATTAGAAACAGACCCATTAACTCTCTTGTTCAATACCACTACTACTACACAACAACAACAACATCAACAACAACCACCTCACAAATTAGAGCAAATAATCAAAGGATCAGCTGCAGCCGCCTGCTTACACGACGTCGTTCCATTCAATCATCTTTTTGATTGGCCTCCTCCACCACACCAGGAACAACCACCACCTAGTTCTCCTACCAACAGAGGTTTTTATTAA

>CmWRKY13

ATGTCGGATGAAATGTTTAAAGATTTATTTTATGGTGGGATGGATGAGTATGAGTCTATAGTGAGAGCTTTTGGAATAACATCGGATTATTCGAATAATAATAATGAAATATCGGGGACGACTGCAATGAATTCTTCTTGTTCGTTTTCATCTTCGGATGCTGGAGGAGGCGAGGACGATGATTCTGTGAAAGAGAAGGAGAAACATATCAGTAAAGATGTTGTGGAAGATAATGGAGGAGAGAATTCCAAGGCAGCTGGATCGGGTAAATCGAAGAAGAAAGGAGAGAAAAGAGAGAGAGAAGCGAGAGTCGCTTTCATGACAAAGAGTGAGGTTGATCATCTTGAAGATGGATATAGATGGAGAAAATATGGACAGAAGGCTGTCAAGAACAGTGCTTATCCTAGGAGCTATTACAGATGCACAACTCAAAAATGCGGAGTGAAAAAACGAGTAGAAAGATCCTACGAAGATCCATCCATTGTAATTACAACCTACGAAGGTCAACACAACCACCCAATTCCGGCGACATTAAGGGGGAACCTGTCGGCGGCCAGCGGGACATTTCCGCCGTCCATGCTGACGCCAATGCCTGTGGTCGGTGGCGTTGGGTTTCTGCCTGCAGAACTATTGAGCAATGCTTCCAGCAACAACCAAGCAGTCGGTGGCGGTGCCACGGTTTATTCACACAACAGCTTCGACTATACTTATAACGGTCGGCAACCGGAATATGGGCTTCTGCAGGATATTTTTCCGGCTCCGTCGTCGTTTTTCAACCGGCAGCCGTGA

>CmWRKY14

ATGGACAAAGGATGGGGTCTAACTCTTCGTGATTCTGATCATCAGTCAATTGGGTTCTTCTCAAACAAGCAACCACCACCCCCACCCCCACCCACTCTCAATTCCTTCCAGAGAATGTTTCAAGGTTTAGAATTCTCCGCAAAACTTGGCCACACCGACTCCACTTCCGACGATAATAATCGTTTGGCCGTCGAGGTCGATTTTTTCTCCGCTAAGAAAAGACTGGTTGATGATCTTGAGGCCGATCAAGACTCCAAACCTACTTCTACTACTTCTATCATTAAAGACGATAAAGCTTTGACCCCTCCGCCTCCCCCCACAACTTCCTTCAATCTTGTTAACACTGGATTGCATCTGTTAACTGCTAACACCGGAAGCCATCAATCAACCGTTGATGATGGAATTTCATCAGATGGTGAAGATAAACGAGCCAAAAACGAGCTAGCGCAGCTACAAGTGGAGCTTCAGCGAATGAACGCAGAGAATCACAAGCTAAGGGACATGCTAAGCCATGTGAGCAACAACTATAGTTCTTTACAAATGCATCTCTTGACCTTAATGCAACAGCAGCAGCAACAACAAAATCACTCCTCCGAACCCGCTAATCAACGAGAGATTGCTGGGGAGAAGAAATCCACGGAAATAAAACATGAAGTTGGAAAGGTAATGGTACCAAGACAATTTATGGATCTAGGACCAAGTGGAAATAACAACAACATGGGTGAAAGCGAAGAATTATTATGTAATTCCTCATCGGATGAAAGAACTCGTTCTGGGTCTCCATTGAACATCAACAACAACAATACCGAAACCGCTTCCAAAAAAAGAGATCACGCGGAAATCATGCCTCCTAATTCCGATCACGAAAATTCCAAAAGATCCATCCCCAGAGAAGATAGCCCCGAATCAGAATCTCAGGGTTGGGGTCCCAACCATAAGACCCCCCGCTTCAACAATTCTTCTAATTCTAAACCAATTGACCAATCCACCGAAGCTACCATGCGTAAAGCCCGTGTCTCAGTCCGTGCTCGTTCCGAAGCTCCCATGATTTCCGATGGGTGTCAATGGCGGAAATATGGCCAAAAAATGGCTAAAGGAAACCCTTGTCCACGAGCCTATTATCGCTGCACAATGGCTGTGGGTTGCCCTGTTCGCAAACAAGTCCAACGTTGTGCTGAAGATAGGACTATATTGATAACAACTTACGAAGGCAACCACAACCACCCACTTCCGCCGGCGGCGATGGCGATGGCGTCAACCACGACAGCGGCGGCTACTATGTTATTATCAGGGTCTATGTCAAGTGCGGACCATAATTTAATGAACCCAAATTTATTAGCTCGAGCCATACTTCCATGTTCTTCAAGCATGGCTACAATTTCAGCTTCAGCTCCATTTCCAACAATCACATTAGACCTCACTCACAGTCCAAACCCATTGCAGTTTCAAAGACCCACCGCAGCGCCTTTCCACGTGCCGTTCCCTGGCGGACAACCACCATCAGCCGCCGCCCAATTGCCTCAAGTTTTGGGACAAGCCTTATATAACAATCAGTCAAAATTCTCCGGGCTACAGCTTTCTCATGAAATGGGAGCTAATTCCTCTCCCTTGGGTCATCACCAAATTACACAACCCGCAACTCCAGCCCAACCTGGTGGTGCTTCTTTTGCTGACACGTTGAGTGCCGCAACGGCTGCTATCACCGCTGATCCCAATTTCACAGCCGCGCTCGCCGCCGCTATCTCCTCCATTATCGGCGGAACCCATCCCAACAATAATAGCAACACCAATACCAGTAATAATACAACAACCAACAACAACGGAAGCAGCAACAACAACAGCAAAATTAGCAGTTTTCCTGGAAATTAA

>CmWRKY15

ATGCAGGTTAGAGAGATTAGGGATGAGAGGGGGTTGAGCTTTATGAGAGGGAGAAATGCGATTGGAAATTATGGCGGTGATGACGACAAGGAAAATGACGGTAAGCCTCGGTTGAGGGTGTCGACGATGAAGATGAAGAGGATAAAAGGTAGGAAGAAAGTGAGAGAGCCAAGATTCAGCTTCAAAACCATGACGGATGTGGATGTTCTTGACGATGGTTACAAATGGAGAAAGTATGGCCAAAAAGTTGTTAAGAACACTCTCCATCCAAGGAGTTACTATCGTTGCACGGAAGAGAATTGTAAGGTAAAAAAGAGAGTAGAAAGGTTAGCCGATGATCCAAGAATGGTGATAACAACTTATGAAGGAAGACATGCTCATTCCCCTTCGGATCATAATTTAGAAGATCCTATAATGGGTCACTTACCCTCTTCCCACCTCACCAGTTTCTTCTGCTAG

>CmWRKY16

ATGAACTCCCTTCAAAACCCTACTTTCTTCTTTGACCACCATCAACAACTTGATCAAGACTCGTCTTCTTCATTCATGGATTTCCTTAATTTCTCAGGGTACCCGCTTCCCGATTTCGGCCTTGAAGCCGAGACCACCATGTTTTCGTTGTCCGAAGCGGGAACCGGCGATGGGAGTAGATCCATGAAAGCAACATCCATAGACAATAATACCATAGATGATGGGTGGTTTGAGGGTAAGGGTGTAAAGAGAAAAAAAGAGAGAGGAAATGGGTGTAATCATAAAGTTGCATTTATAACAAAATCTGAATTGGAAATCTTGGATGATGGCTACAAATGGAGGAAGTATGGCAAAAAATCTGTCAAGAACAGCCCTCATCCAAGGAATTACTACAAATGCTCAAGTGGAGGGTGCGGAGTGAAAAAGAGAGTAGAAAGAGACAGAGATGATTCAAGCTATGTTATAACAACATATGAAGGAGTTCACAACCACGAGAGCCCTTTTTTGATGTATTCCAATGGTTCAAAATTATGTCATCCTCATCCTCAGCCCATTTGCCCTAATTCCTCTTCTCCCGATCCCTATTCTTCTACTACTACCCTTTGA

>CmWRKY17

ATGGCCGTGGACCTCATCTCCCACCTTTCCCCACTCCCCAATATGGAACCCAACGCCGTCCAAGAAGCCACTTCCGGCCTCGAAAGCGTCCACAAACTCATCCGTTTACTCTCCATTCCCAATCCCCATTCCTTACCTTCCTCCTCTCAGTCCCCAATCGATTTCCCTTCCGACTGCCGCGCCGCCGCCGACGCTGCCGTCTCCAAGTTCAAAAAAGTCATTTCCCTCCTCGGCCGTAGCCGCCTCGGCCACGCTCGCTTCCGCAGAGCTCCTCTGCCTCAACAACCTCATTACGTTACTCCCATCCAACAGATCCCACCTCATCCCCATCCCCATCTTAACAGCAACAACAGCAACGATGAATCTCTTAATTTCTCCGCTCACAATTCCTTCATTTCTTCTTTGACCGGCGACGCCGATACCAAGCATCCTTCTTCCTCTTCCTCGCCTTTTCTCATTTCCAACCTCTCCCAGGTCTCCTCTGCTGGGAAGCCCCCTCTTTCTAGTTCTTCCCTTAAGAGGAAGTGCAGCTCCGACAACTTGGGATCCGGCAAGTGCGCCGCTGCTTCTTCCTCTGCTCGATGTCACTGTTCCAAAAAGAGAAAATTGAGGGTGAAGAGAGTGGTGAGAGTTCCAGCAATAAGCTTGAAAATGGCGGACATCCCACCGGACGATTATTCATGGAGGAAGTATGGTCAAAAGCCCATTAAAGGCTCTCCACATCCCAGGGGTTATTACAAGTGTAGCAGTGTAAGAGGTTGTCCAGCCCGGAAACACGTAGAACGGGCCGTAGACGATCCGGCGATGCTAGTAGTGACTTACGAAGGAGAACACAATCACACCCTCTCTCTGCCCGAAACCTCTAGTCTCATCCTCGAGTCCTCGTAG

>CmWRKY18

ATGGATAGTAGTTATATAAACTTTCTCCCAACTTCCTCTTCCAGCTTCTTCCATAACTCATTAGCTATGGACGACCACGACGATGAGTTGGAAGAAGAAAGCAGTTTGAAAAAAATTAAAAGTGAAGTGTCTGGAGGGAAATTGAAGAAGAAGAAGACAAGAAAAAGGAGATTCGCATTTGAAACGAGGAGCCAAGTTGATGTTCTTGATGATGGGTATCGTTGGAGAAAATATGGTCAAAAGGCTGTTAAGAACAACAAGTTTCCCAGGAGCTATTACAAATGTTCGAACGAAGGATGCAAAGTGAAGAAACAAATTCAACGGCTAACAAAGGATGAAGAAGTTGTATTGACAACCTATGAAGGAGTTCATTCCCATCCTATTGAAAAACCTCATGATTCCTTTCAAAATATCTTGACCCATATGCATATTTACTCTTCTTAG

>CmWRKY19

ATGGATTGCTCCTGGCCTGACACCTCCCCCTTTGATCGAAGAAAAGCAGCCGATGAATTGCTTCGTGGCCGTGAACTTGCACAACAACTACGAGCGTATCTACAGAGAAGTTCCACCCCGGCCTCCCAAGATCTACTCACCAGAATCCTATCCTCTTTCTCCAAAACACTTTCCATCTTGAATCATCGCTGCGACTCCGATGACATAAATGGTTCTATTGTCGACTCACCCGAGGATCACGGTAGTAGAAAATCCGAGGAATCTGGAGACAGTTGCAAGAGCTCCACCCCTAATAATGATCGCAGGGGTTGCTATAAGAGAAGAAAGAGTTGCCAGAGTTGGGCAAGAGAAACCTGCAGCCTAGTGGACGATGGGCACGCGTGGAGGAAGTACGGGCAGAAGACGATTCTGAATGCCAAATACCCGAGAAACTACTTCAGATGCACCCATAAATACGACCAGGCCTGCCAAGCCACAAAACAAGTCCAGCGACTGCAAGACCATCCCCCCAAGTTCCGTACTACTTATTATGGCAATCACACATGCTCCAATTTCCTCAAGGCTTCCGACATAGTGCTCGGCTCCTCCAATTTCGACGATTCCTGTGGAGTGCTCCTCAGTTTTGACACCACTGCAGCACCCAACTTTTTTCTCCCACACCATCCTACGTTGGTTAAGAAGGAAGAAGTTGTAACCCCCGAAGCTGGCAGCGGCAGGGATGACGAGGCAGTCTGTTCCCCCTCCGATTACATGAGTACGGCCGACGACCATCTCTCCGAGGTTTTCATGGGTTCCGTCGTCGATTTTGAGGACGATGTCTTACCATTCCACTTTGATCCAATTAATTTTAATAATACCCCTTCCGATCCCCTTGATCTTCCTAGTTGA

>CmWRKY20

ATGAATAGCATAAACCAAACAATAAATACACTCGCTGGGGGATCTTCTGACAACAGAACCAATAATTTCGCCATGGAAGTTCCTAAATTCAAGTCTCTTCAACCTCCTCCATTTCCCATGTCTCCTTCTTCTTACCTTTCCTCTTTCTCTTCTGGTTTAAGCCCCACTGAGATTCTAAATTCCCCTCTTCTCTTTTCCTTTGGTGTTTTTCCATCTCCTACTACCGGTGCTTTAAATTTGAGAAATGATTGTGAGGAGGTTGACCAACAAGAAATGAAGGGAGATGTTAAAAATTACTCTGTTTCTGCGTATAACCCACAAACCGGATCCTCTCTTTCGTCTTATTTTCAGTCCTCTTCTTCCAACGTGACTCTACTGAATCCAAGCGGTTTGTCGTGTGATGAAAGTGGGGCTAAATCAGAATTTGTTAACACAGAAATGGCGGCAGCTGAATCAAAGCAAAATTCTCAGTTAGCAATTTACAACAGAGAACAACAAAAATCAGAGAACGATGGATATAATTGGAGAAAATATGGGCAAAAACAAGTGAAAGGAAGCGAAAATCCACGAAGCTATTACAAGTGCACGTTTCCAAGTTGCCCAACAAAGAAAAAAGTTGAAAGATCATTAGATGGCCAAATTACCGAGATTGTTTATAAGGGAACCCATAATCACGCCAAGCCTCAGCCGACAAGACGTTCCGGTAACTCCGGCGTTTACGATCCATCGGCGGCGGAAAGCGGGGTATTGCAGGAGGATTGTTCGGTCTCGGTGGGGGAGGAGGAAATCGAACCCAATTCGCCGTTTAGCAACTCGATTGAGGAGAATGAAAAGGAACCGGAAGCTAAGAGATGGAAAGGGGAAAATGAGAATGAGGGATATTGTGGGGGAGGAAGCAGAACAGTGAAAGAGCCAAGAATTGTTGTTCAAACAACAAGTGAAATTGACATACTGCCTGATGGTTACAGATGGAGGAAATACGGACAAAAAGTCGTCAAAGGAAATCCAAATCCCAGGAGCTACTACAAATGCACATCTTTAGGCTGCCCGGTGAGGAAACACATCGAGAGAGCAGCCAACGACATGAGGGCTGTGATCACTACCTACGAAGGCAAACACAACCACGAAGTTCCAGCAGCACGTGGCAGCGGTGGCGGTGGCTATAACACCATCAACCGACCTATACCAACCAACATTCCAATGGCATTGAGGCCATTATCCGTTGTCACCAGTGATTCTTTCCCGGCAAATTTTCCAGCTGCATTCCGGCCAGGAAATTTAGGAATGTCAGAAATAGGAACACAAGCATCATCATTCCCATTTCAAACCTCACAAGGAGGGCCTCCAAGTTTCCAAGTGTCAGGATTTGGATCGGCAGCCAAGGAAGAAGTAAGAGACGACACGTACTTCATCAACTCATTTCTATCTTAG

>CmWRKY21

ATGGACAATAAAGCGGCAGAGAGAGTTGTTATTGCCAGACCAGTAGCTTCAAGGCCAACATGTTCCAGTTTCAAGTCGTTCTCTGATATTCTTACGTGTGCGTTCGATACTTCTCCACCAAATACGTCATCCGAAACCAAGATTGCTGCCATTAGACCAAAGACAGTGAGGTTTAAGGTGAAGGATAATCCTGGTCCATCATCCGGGGGAAAAATATCAGAAACGGTCCCCAGAACGAACTCTCATGGTTCATCTGATACTCTCGCTGTATCAGACAGCAAAACCACTGTTATATTCAAACCTTTGGCGAAGCATGGGAACACCAATTTGCAAAACTGTTTACCACTTCCACCAGTTGAGGTCTGTATTCAATGTCCGAATCAAGATGATGTCAATTTCCAATCTGCACTGACGTCAAATCTCTGTATTCAATGTCCAAATCAAGATAGTGACAACTTCCAATCTGCGTTGACCTCAAATCTTCCTCAGGACATCACATCCACTGTTGAAAACAGTCAATCTATTAGAAGCTCAAGGGTTGCTTTAAGTTATAGCAAAAAAGATCCAACATCATCGCTTCGTCCTCAAATTAGTGGTGCTCAGCCTTCTTATGACGGATATAATTGGCGGAAGTATGGGCAAAAGCAAGTAAAAGGAAGCGAGTATCCACGGAGTTATTACAAGTGCACACATCCAAGCTGTCCTGTCAAAAAAAAGGTTGAGAGATCATTGGATGGGAAGGTTGCTGAAATTGTTTACAAAGGTGAGCACAGCCACCCAAAGCCTCAGCCTCTAAAGCAAAACTCATCTGGGACACAAAGGGAAGGGTCAATATCAAATGGAACTACACGAGATACGAATCCGGAGTTATGGCTCAATTACCTCAATGGGCGGATAGAAGGTTGTGAAAGTAGGATTGAGAATCACATCGAAAAAGCTTGTCAAGGTAGAGGGGTAATACCCTTCGATCCGTTTTCAAATCGAGAAGTCAATGCTGGATGTGGAATCTCCGATAATTCATGCGGTCTAAGTGTAGAATGTGAAGAAGGAAGAAAAGGACTTGAGTCCATGGATGATAAATTGCGAAATAAAAAAAGGGGTGGAAAAAATCCAACAAATGAAGGTGAGACATCAATTGAAGGTGTTAATGAGCACCATGCAATGAATCGAGGTTCCACTGGCATCGAGATTTCTGGCAAAGGCATTCGCTGGAGAAAATATGGGCAGAAAGTTGTGAAGGGTAATCTATACCCTCGAAGTTACTATAGATGTACTGGCCTCAAATGCAAAGCACGCAAGTATGTCGAACGAGCATCTGAGGATCCAGATTCTTTCATCACAACTTATGAAGGAAAACACAATCATGGTATTTCACTTGAAACTTCAATTTCTGTAGCTCCTGAAATGGAATAA

>CmWRKY22

ATGGAAAGTTGTCTTTCTTCTATAACTACTAACCAACCATGTATACATGAAGAATCGACACAAGGAGATGACATTGACACACAACATCCTTTAGAAGATGAACAGAAAGGGTCTTATATTCCTATGGGAATGTTAAGGACATCAGAAGATGGATACAACTGGAGGAAATATGGACAGAAACAGGTCAAAGGTAGCGAATATCCTAGAAGCTACTATAAATGTACGCATCCAAATTGTCTCGTAAAGAAAAAGGTGGAGCGATCTCTTGATGGTCAAATAACTGAAATCATCTATAAGGGTGCTCATAATCATGCAAAACCTGATCCCACTCGTCGAGCCATGGTTGGATCTGTGCCAATTTCAGGGGATACTCCAGAAATTGGTGAAGGTGGTGGAAACCATTTCAAACTTGAAGCTGGGCTGACGTGGAGGAACACTCAATATGGGGTGAAGGATATCAAACCTATCTCAAATTGTAGTGTTGATGGTCTAGAGAGGACGTCCTCTGTGTCAGTTTTGACTGAACTTTCTGATCCATTGTTAAATCCCCAAGAGAAAACTGTTGGGGTTCTTGAACCGGTAGGAACACCTGAGCTTTCATCCACACTTGCCAGTCATGATGATGATAGTGGTGGTGGTGGTGACGATGATCTTACAACTCAGGGAAGCATTTCAGTTTGCACAGAAGCTGATGATGCCGAACCTGAATTGAAAAGAAGGCGAAAAGAGGAGAGTTCAATTGAGACAAACTTGGCTTCAAGATCTGTACGTGAACCAAGAGTTGTTGTCCAAATTGAAACTGAAGTTGACATACTTGAAGATGGGTATCGGTGGCGGAAGTATGGTCAAAAAGTTGTCAAAGGAAATCCAAATCCAAGGAGCTACTACAAATGCACGAGTGCAGGTTGCTTAGTAAGGAAACATGTTGAGAGAGCATCACATGATCTGAAATGTGTCATTACAACGTACGAAGGGAAACACAATCATGAAGTTCCAGCAGCAAGAAATAGCAGTCAAGTAAACTTGAGTAATGGCAATGCCCAACCACCTGCGTCTCATGTGCAACCGAACATGGGTTTGTCTAGAAACTCAAACGTCCCAAAGTCCGAAACAGAAATTCAAGATCTTGCCACCCATTTTTATCCAAAACCAGAATTTAACCATGACTATCAAAGGTCTGGTTGTTTTGACACTTTTACAAACGATATGAAACTTGGAGCTCCTCCATTCTGTCAAATGAAGTTCCCTCCACTCCGGAACACCTTGCCATACAGTTCCTTTGGATTAAGTTCAAAGCATACTGCAACAGGAATCTCTGGGTCTTTGGCATCTGTGGTCTCGGACTTACCGATTTCTTTGCCACTGAACCAAAAACTTTCGGCTGCTGGTTACGATTATACCAATGGAAGACCAATACTACCATTTCAGGTTTTCTTGGCTGGTCAGCAGCTGAGGGAGACAGATAGATTCCTCACACCCAAACAGGAGCACGACGATGATAACATCTGTGCTTCATTCCAGCCAGTTGTTGATAGTTCGAGTGGATCTTCATCGTCATCGATTTCATCAGTTTATCAACAAATCATGGGAAATTTTACATAG

>CmWRKY23

ATGACGGAGCCGGAAAGCTTTGGAACTGACCAACTCGGAAGCTCCAAGGCGGCTATTGAAGGGCAGGAAGATGATGAAGAAGAAATGGAAGATTCGGATGATGAACCGGAGTTGGAAGGAGAAGGAGGAGGAAGAGTGAGTGAGTTGAAGCCGACTGAGTTGAGAACTGGTCCGTCGGTTTGTGAAGCTGTGGTGATGGGTTCACTTTCTGAAACCCTAACGGTGGCTTTTGTGAATCAGTCCTCTGAAAACGGTCGGTCTGATGGTTTGCCTGTCAATTCTTCTGCTCAGTCTGTCGAAGGAGCTGAATTGAAGCAAGCTCCATCTTCCCACAGTGAGCCTTTGGCGGTTGAATCAACCCAGACAGATAAGGTGCAAGAACAAAACCATCTTCAGTTGACAGTATTTAAAGGGCCTGATTCGGAACAATCACCAACTTCAGTTACCCAGTCTATCTCATCCTCTGCAAGTCCAAATTTATCTGAACATAAGCTGTCACCTAAGGTCCAGAAAGTATGTAAGCCAGAGCCAAACCAGAAAAATTTCTTCAACCATAAAACTCCATCATCTGTTCCCAATGCAAGGACACCTGCTTCTGATGGTTACAATTGGAGAAAATACGGTCAGAAGCAAGTGAAGAGTCCTAAGGGTTCACGTAGCTATTACAAGTGTACATATTCTGAATGCTTTGCTAAGAAGATTGAATGTTGTGATGACTCAGGGCAAACAACAGAGATTGTTTACAAAAGTCAACATAGCCATGATCCACCTAGGAAGATTAGTATACCGAAGGAAAGTAAGCTTGTGCCCTATGTTGAGCCTGTGGTTAAAAAAATCATTGCTGAGCATTCCAGAAGAGTAATTAATGATTCAGATCCTCCCACGTCTTCAAAAGAACCGTTACGAGAAACAGCTATAGTTGTCTTTGAAAGGAAACGACAGTACTCTAACGACTCTAACGGAAATGATGAATTTAAAATCAAAGATGAGAATGACTATGAAACTGAGGCAAAACTAAAAGTTAAGAAAGGCAGTGCGGGAAACTCAGGCACTTCCTTAAAACCTGGAAAGAAACCCAAGTTTGTGGTGCATGCTGCAGGTGACGTTGGAATCTCTGGTGACGGATACAGATGGCGCAAGTATGGTCAGAAAATGGTGAAAGGAAATCCTCATCCTAGGAACTATTACCGATGTACTTCTGCTGGTTGTCCAGTCCGTAAGCACATCGAATCAGCAGTGGAAAATCCTAATGCAGTAATAATAACATACAAGGGAGTTCACGATCACGACACGCCTGTACCAAAGAAACGACACGGTCCACCAAGTGCTCTTCTTGTAGCTGCTGCAGCACCAGCCTCCATGAGCAGCAATACACAACCGAAGAAAACTGATGTGGTCGAGTCCCAAATATCTTCAACACAATGGTCTGTGGATGCTGAAGGAGAGTTGACTGGTGAGGCCTTGGAGCTTGGGGGTGAGAAGGCAATGGAATCTGCTCGAACACTTTTGAGCATTGGATTTGAAATCAAGCCATGCTGA

>CmWRKY24

ATGGAACCCACAACTACCACCATCAATACTTCTCTTGACCTCAATTTGAATCCTCCGCCCTACACCGACGACCAATCTCATCCACCCACTCCTAATTCTCCACTCAAACAACAGGCTCCAACAACAGGCATTCTTGCTGAAAAATTGAATCGGATTAGTTCGGAGAATAAGAAGCTGAATCAGATGCTTGGGGTAGTGGTTGAGAATTACAGCGTTTTGAAAAATCAAGTTATCGATTTACTCATGAAATCCAGAAAACGAAAAGCAGCTCCAGGATGTGATAATTGTTGTAATTTCAATCGGAGCGCTTCCGATCAGTATTGCGGTTGTTGTAGCGATGATAATGATTCCTGTTATAATAATAAGAGGCCTAGAGAAAATAATAGTAAACCCAAGGTTATGAGAGTCCTCGTTCCCACCCCAGTTTCTGATTCCACTTTGATTGTGAAGGATGGATATCAATGGAGGAAATACGGTCAAAAGGTGACTAAAGACAATCCGTCACCAAGAGCTTACTATAAATGCTCATTTGCCCCTACCTGTCCGGTGAAGAGAAAGGTACAAAGAAGTGTTGAAGATCCATGTTATTTAGTAGCAACATATGAAGGACAGCACAATCATCCAAAACCCAATTCAGGAATTGAGTATCAACTAGTTGGACCAATTAATTTAGGTTCAAATACAAAGCTTGATTCTTCTAATAATGTTTCGTCATCACCTTCTTCTTCTATCAAATCTCCATCATCATCATCATCATTAATACCTTCTATTTCTCTTGATTACTTAACTAAATCTCAACCTCAAATACCATCACCTTCATCATCTAACTCTTCTTCTTCAACACAAAAGCTTCTTGTTCAACAAATGGCCACTCTTTTGACCAGAGACCCTAATTTCACTAGAGCTCTTGCCACTGCCATTACCGGAAACATGGTGGACAATGAAATTTGGAGATGA

>CmWRKY25

ATGGCCGATTCTCCTCCTCCTCCTCCTCCTCCTCCTTTCTCCACCGCCGATTCCTCCTCTACTTCCTCCAACGCTCTTTTCTTCTTCCCTCCCGATTCCGATTCCACCATCCTCACCCAATTCGGTTGGAATTTCCACTCTCTTCAACCTCAACCTTCTCGATTCCATGACTCTCACCCGATCCACTCCGATTTCCCTGCAACCTCCACTACTACTACTACTACTACTACTACTACTACTACTATTACTACTACTCCTCCACTCATCGAAGACTCCGCTGGATTATCAGACGCTCCTCTTCCTCTCCCTTCTAATCCTTCTCTCTCTTCCAGCTCCAGCGGTGATCCCCCGGACAAACCGCCTGAGATCATTACTCCGCGGAAGGTGAAAAAGAAGGGGCAAAAGCGAATTCGGCAGCCAAGATTTGCGTTCATGACCAAAAGTGAAGTTGATCATCTTGAAGATGGTTACCGGTGGAGGAAATATGGACAAAAGGCTGTCAAAAACAGTCCTTTCCCTAGGAGCTACTACAGGTGCACAAACAGCAAATGCACAGTAAAAAAAAGGGTAGAACGGTCGTGTGAAGATCCAAGCGTTGTAATAACAACATATGAAGGCCAACATTGCCATCACACCGTTGGATTCCCTCGAGGTGGATTAACCATAGCTCATGAAACTTCTTTTGGTTCTCAATTTTCACCTCAAATCCCACATTTTTTTTACCCTGATCCATCACCACCACCACCTCCACCCCCAACAACAACTAATAACCATAACCCTCCCACACCTCCCATTGACCAACATTTCCCCAGTACTTCTAGTTCGACTGAACAACAAGAACCACCAAATTCACATCTCCAACAACTTCCTTCCAACGAAGGCTTGCTCGGTGCTATCGTCCCCCCTGCCATGATGCGTAGAACAACATGA

>CmWRKY26

ATGGGTGAAGATGATGAACAGCCGCCGCCGCCGCCGCCGCCGTCGAAATCTAAGCCTTTTGCGTTGAGACCCACTATTAATCTGCCACCACGGACTTCTATGGAGTCCCTCTTCAGCGGTGGCCCGGGTTTGGGATTTGGGTTCAGCCCAGGTCCGATGACTCTTGTCTCGAGCTTTTTCTCTGATTCCGACGACTGCAAGTCTTTTTCTCAGCTCCTCGCCGGAGCTATGGCGTCGCCGGTGGCTGCCGTTCCTCCGTCGACGTCTGAGTTTAAGTCTTCTCCCGGTCTGCTTGATTCTCCGGGGCTTTTTTCGCCTGGTCAAGGACCTTTCGGAATGACGCATCAGCAGGCTTTGGCTCAGGTTACTATGCAGGCTGTAGAAGCCTATTCTCATAATCAAATGCAAGCCGCGTCCTTTTCTTCATCTGTAGCACCTTCTGCATCCTCCTTGCAGCTTTTGACATCCTTACCGGGAGAGAAGACTAAAGATCAGCTGATGCAACTTCCATTCCATAACTCTTCAGTTGCATCCAAGGAGCCATCTGATAACTCTCAATCAGAGCAGAGATTGCAATTGTCTTCGTGCAATGTTGATAAACCTGCTGATGACGGATACAATTGGAGAAAATATGGACAGAAACAAGTGAAGGGCAGTGAATTTCCTCGAAGCTACTATAAGTGCACGCACCCGAATTGTCCTGTCAAGAAAAAGGTTGAACGATCTTTGGAAGGACAAGTTACTGAAATTATCTACAAGGGCGAGCACAACCATAAACGGCCTCAACCAAATAAGCGATCAAAGGATGTCGGAAATTCTAATGGATATTCGATTGTTCATGGAAATCTTGAGCTATCTTCTCAAGTTCGGAGTGGATATTTGAATAAATTGGACGAGGAGACATCTATCTCTTCCATCAGAAAGAAGGATCAAGAATCAAGTCGTGTCACAAACGATCAGTTCTCAGGCAATAGTGATGGTGAGGGAGGGAGTGAGATAGAAACAGGAGTGAACCGAAAAGATGAAGACGAACCTGATGCCAAGAGACGGAATACTGAGGTTAGAAATTCTGAGCCAGCATCTTCTCATAGAACTCTAACAGAATCCAGAATCATTGTGCAGACTACAAGTGAAGTTGATCTATTGGACGATGGTTACAGGTGGCGTAAGTATGGGCAGAAAATTGTTAAAGGAAACCCTTACCCTAGGAGCTATTATAAATGCACAACTCCAGGATGCAATGTTCGTAAACATGTTGAGAGAGCTTCAACTGACCCGAAAGCTGTCATCACGACATACGAAGGTAAACACAATCATGATGTTCCACTTGGTAAAACCAGCAGCCACAGTTCAGTCAGCGGTAACATTTCTCAGCTAAAATCACAAAATATAGTTACTGAGAAAAAAAATTCAAGTAACAACACTGATCGTGGGAACTCCCATCAACAGCCCACCGGACTTTTACGGTTAAAGGAAGAACAAATAACATAG

>CmWRKY27

ATGTCAAATGATGAAGGGAAAAATGTGTACCAGCAGTATGATCCATTTCAATACAACCAATTGGATATGAACCGTTCGATCTTCCATCAACAAGCTGCAGCGGCGGCGTTGGATCCTGGCTTGATGAGTTTCACTAATTTCTTCGATACCAGCTCTTTGGATTATAATAGCCTGTCGAAGGCGTTCGATGTGTCGTGCTGTTCATCTCAAGTCATTTCTGCAGTGGACGACATGTCGAAAAAGAAGGCTTCAACGACAACCCCAAATTCTTCAGTGTCGTCTTCATCTAATGAAGCTGTAGTTGAAGAGGATTCAGTGAAGAGCAACAAATTAGAAGACATAAAAGGGCGGTGCGAGAATAAAGATGAAGAAAAGTCTAAGAAACAGAACAGCAATTTATCAAAAAAGAAAGAGAAACGGCCGAGGGAGCCCCGTTTTGCTTTCTTGACTAAGAGTGAGATTGATCATCTTGAAGATGGATATAGATGGAGAAAATATGGTCAGAAAGCAGTTAAAAATAGTCCTTACCCCAGAAGCTACTATAGATGCACCAGCCAAAAATGTGTAGTGAAAAAACGAGTTGAAAGATCATATCAAGATCCATCTGTGGTGATTACTACATATGAAGGCCAACACAACCACCATTGCCCAGCTACACTCCGAGGCCATTCTGCAGGAATCATGTCGTCTCCATTCTACGCATCGGCATCAACATCAGTAACAGCAGCCTCATCCGGGCCTACGCTTCCCCAAGAACTCTTCTCACATTTGTTACCAACCAATAACTGCCAAAACGACCCGGCCTCAATGATGTACCAAAATCTAAGCCTTCAACAACACCTTCAAATGCCTGATCATTACGGTTTGTTGCAAGATTTATTCACTCAAAAATAG

>CmWRKY28

ATGATTTTGTTTTACATTCTTTTCCCTTTAGGATTCGTCGTTCGGGCTCGGAATCGAGTAGTAATGGGGAGAACTGATGATAATGTTGCTATTATTGGGGATTGGGTACCTCCAAGTCCTAGCCCAAGAACCTTTTTCTCAGCAATGCAAATGCTAGGGGAGGATATTGGTTCTTCTAAACCTTCAATGGACACTACTACCAGCAGTGATCATAAAACCGAAGAACTCTTTCTCAGGCCTCGAGAACAAACCGTGTCCGAAAACGCCATTGCAAGAGGCGGGATCCCGGGTGTCAATTCAGGGGACCGGGGAATGGAGTTCGGTACGTTCTCAGAGCAGAAATTCCGTGGGGGACTCGTGGAAAGGATTGCAGCTAGAGCTGGCTTTAATGCTCCAAGATTGAATACAGAGAGCATTAGATCAACAGATCATTCTTTGAATTCAGAAGTAAAGTCTCCTTATTTGACAATACCACCTGGTCTCAGTCCAACTACATTGCTAGATTCTCCAGTTTTTCTTTCAAATTCACTGGCTCAACAATCTCCCACAACTGGGAAGTTCCCATTTTTACCAAATGTAAGTAGCACTCGGAGCTCGACGATGATGCCAGAGGCCAACAACAAAGGCAACAACAATCCGTTCCACGATAACAATACATCATTTGCTTTCAGACCCAGTGTGGAATCTGGATCCTCCTTTTTTCTCAATGCAGCAAGCAAAACAGCTTCTGCTACTGTTCTTCCACAATCTTGTCCAAGAATTGAGCTTCCAGTTCCACGTTCAGAAAATTCTTTTCAATCTCATCTTGCTGAACCATCTCTGTCTTTACCCCAAAATCGAATCGGTCACCATCCTCAAGTGGGACTCTCTACAACGTACGTTGAGAAGGACGATGGGAGTAAGGCTGTGTCAGAAGAGCAAAGACCCTTTGATTCTCTTGGTGGTGGTAGTGGTGAACATTCTTCGCCTCTCGACGAGCAACTGGATGAAGGGGAGCAAAGAGGCAGTGGGGATTCTATGGCTGGTGGTGCTTGCGGTGCTCCCTCGGAGGATGGGTATAATTGGAGAAAATACGGACAAAAACAGGTCAAAGGAAGTGAGTATCCTCGGAGTTATTACAAGTGCACACATCCTAACTGTCAGGTCAAGAAGAAGGTCGAGCGATCTCACGAGGGCCATATAACAGAGATCATCTATAAAGGAGCACATAACCACCCAAAGCCTTCACCAACCCGACGAGGAGCAATCGGATCTTCCGATTCCCATATGAATATGCAACTAGACATCCCTGCACAAGCAGGTCAACAAAGTGCTGATGTTCCTCTTTGGGAAGATTCACAAAAAGGGGTTCCAAGTGGAGCTCCTGATTGGATGCATGAGAACCTTGAGGTGACTTCTTCAGCATCTTTGGGTCCTGAATATGGCAATCAGCCTAACACTTTACAAGCTCAAAATGGGAGTCATATTGAAACAGTTGAGGCCATTGATGCCTCATCCACATTCTCTAATGATGAAGATGAAGATGATAGGGGAACACATGGCAGTATAACATTGGGATATGAAGGGGAAGGAGATGAATCTGAGTCTAAGAAAAGGAAACTCGACGCCTATGTAACAGAGATGAGCGGGGCCACTAGAGCTATCCGTGAGCCTAGAGTTGTTGTCCAGACTACCAGTGAAGTTGATATTCTGGATGACGGTTATCGTTGGCGCAAATACGGACAGAAGGTTGTTAAAGGAAATCCAAATCCTAGGAGTTACTACAAGTGCACAAATCCTGGCTGCACAGTGAGGAAGCATGTCGAGCGGGCATCACATGACCTGAAATCCGTGATAACCACATATGAAGGAAAACACAATCATGATGTTCCTGCAGCTCGCAACAGTAGTCACATCAGTTCAGGTACATCCAGTCCTGTAACTGGTCAAAACTCGACCGCGGCAATTCAAACTCATGCTCACAGGCCAGGGCCACCCCAGCCTCAAAATACCATTCCAAGATTTGAAAGGCCAGCTTTTGGGTTTGCTGGAAGACAGCAAATGGGGACTGCCCATGGCTTTGCTTTTGGAATGAACCAACCTGGATTGGGAAATCTGACCATGGCAGCAGTTGGGCAACCCAAGCTTCCCGTCTTGCCAATGCATCCTTACTTAGGACAAGCACACCATGTTAATGAAATGGGTTTCTTGTTGCCTAAAGGAGAGCCCAATGTAGAACCTACATCTGATCTTGGCTTGAACTTTTCCAATGGTTCAACTGTGTATCAGCAAATTATGAGTAGGCTTCCACTTGGACCTGAGATGTGA

>CmWRKY29

ATGGACCCCACTGATTCCGACCTCCCCGATCCATCTAATGCCTCCTCCGGTGCTAAGTACAAGCTCTTGTCTCCGGCCAAGCTTCCGATCTCCAGGTCCCCTTGCATTACCATCCCTCCTGGCCTCAGTCCCACTTCATTTCTCGACTCTCCTGTTCTTCTTACCAACTTGAAGGTAGAGCCTTCTCCGACTACTGGGTCCTTTACCAAGCTTCCAATGGCACACGACTCTTCTAGCTCAGCGATTTATCCAATGACCAGCATGGCTTTCTCAAATACTAATGCCTCAGATGAAGGAAGATCCAACTACTTTGAGTTCAAACCATATGTTGGACCAAATATGGTTCCTGCAGATTTGAGTCATAGGAAAGGTGAACAATCTTCTGAAGTTCAAGGTCAACCCCAACCTTTTACTGCTCCACCCATGACTAAATTCGAGATCAGTGTCATGTCAAATGATTTGAGTCGATCAACCCAGATGGACACACATACAGTTACTTCAGGGGCTAGTGTTCCTGAAGCCGATGGAGACGATATCAACCATAGTTTGAACACAAATAGCAGGGTTCAGGCTCCACAATCTGATCCAAAAGGCAGTGGCATTCCAGTAGTTTCAGATAGGTTATCTGATGATGGATATAACTGGCGGAAGTATGGACAGAAGCATGTTAAAGGGAGTGAATTTCCGCGTAGCTATTATAAATGTACCCATCCTAACTGTGAAGTGAAAAAGCTCTTTGAACGCTCCCATGATGGGCAAATAACTGATATTATCTATAAAGGTACCCATGATCATCCTAAGCCTCAGCCAAGCCGGCGATATTCTGCCAGTGCTTCTATGAATGTTCAAGAAGATGGGACTGATAAGCCTTCATCTTTATCTGGCCAAGATGACAGGTCTTGCAGCATGTATGCTCAGTCTATGCATACCATTGAACCAAATGGAACTACAGATCCATCCCTGCCTGCAAATGATAGCATCACCGAAGGTGCAGGAACAACTCTGTCATGCAAGAATCACGATGAGGTTGATGACGATGACATATACTTGAAACGGAGGAAAATGGAACTCGGTGGTTTTGATGTCTGCCCAATGGTTAAACCAATCAGGGAACCACGTGTTGTGGTTCAGACTCTAAGTGAGGTTGATATACTGGATGATGGGTATCGCTGGCGGAAATATGGTCAGAAGGTAGTGAGAGGAAATCCCAACCCGAGGAGTTATTACAAATGCACGAATGTTGGATGCCCTGTTCGAAAGCATGTTGAAAGAGCATCCCATGACCCAAAAGCTGTTATTACTACATATGAAGGGAAACATAATCACGATGTTCCCACTGCAAAAACTAGTAGCCATGATGTCACTGGCCCATCAACAATTCCATCATCAAGGTATAGACTCGAGGAGAGTGACACCATAAGCCTTGATCTCGGCGTTGGCATTGGCACAGGTGGTGAGAATAGACCAAATGAATACAGGCAGGCATTGCATTCACAACTTGTTGAAAACCGAGCTCCAAATGGCAATTTCAACTTCGAAGTAGTTCAAGAGAACTCGGCGCCCACCTATTTCGGTGTGCTGAATAGAGGTGTCATCAATTAG

>CmWRKY30

ATGGAAGAGGTTGAAGAAGCTACCAAATCAGCCATTGAGAGCTGCCATGGAGTTTTGAATCTTTTACTTCAACCTCCTCCTTCTCCTCCTCATCAACACCATTTCAAAAATTTAATGGTTGAAACTAAAGAAGCTGTTTTCAAGTTCAAGAAAGTGATTTCTCTTTTGAATTCTGATTTTAGTCATCCAAGATTTAGAAATTTCAACAAAATCCCTCTCCCTTTACCTCAAAATTCCCTATTGGATTCCCCTAATTACACTCTACATCCTCCAAACAAAAATCTTTTCAATTTCCCCCCTGGTTCTAATAGTAAAGTTTCAATTTTTTTGGGAAACCCAGATTTAGAATTGAGTCAAAATGATAAGAATACCCTTCATATCCCCAAACAATCTCCTTCTTTAAACTTTAGCTTCCCCCATCATCATCATCATCAACAACAACAACAACAACAGAGCGTTCTAGCTCATCAGAAACAAATGAAACAGCAAGCTGAAATGACGTTTCTTAGGAATAACAATGGGATGAATCTGAATTTTGATACATCTAACTGTACATTGACAATGTCATCAGCTAGATCTTTCATTTCTTCATTGAGTATGGATGGCAGCGTCATTGGCGACAGAAGCTCGTTTCATTTGATTGGACCGTCGACCACGACCACGACAACGTCGGGCAATAGCAAGAGGAAGTTTTCTGCTAGAGGAGAAGAAGGGAGCTTGAAATGTGGAAGCACTAGTAAATGCCATTGTTCAAAGAAGAGGAAACATAGAGTGAAGAGATCAATAAAAGTACCTGCCATAAGTAACAAACTTGCAGATATTCCTTCCGATGATTATTCATGGAGAAAATATGGGCAGAAGCCAATTAAGGGTTCTCCTCATCCAAGGGGTTACTACAAATGCAGCAGCATAAGAGGTTGTCCGGCAAGAAAGCATGTCGAGCGGTGCTTAGAAGACCCGTCGATGCTTATTGTAACGTACGAAGGAGAACATAGTCACCCGAAAATGTTGACGCAATCTGCACACACTTAG

>CmWRKY31

ATGGAGAATTATCAGATGTTTTTTCCTTGCTCCGATGGCGGCGGAGGACTGTCAGCCTATCATCATGCGGACATGTCATCCGGCGGTGCTTCTGATATGTTCGGTAATTTTCAGGGTGGCGATATGGAGGCGGTAAGTGGGTTTTTGGGGATGAAAAGAGAGGTGGATGGGGCGACGGTGGAAGCAGAGGGCGGTGGGAGAAAGAAGGGAGAGAAGAAGGTGAGGAAACCGAGATATGCTTTCCAGACAAGAAGCCAAGTTGACATTCTTGACGATGGGTATCGTTGGAGAAAATATGGTCAAAAAGCTGTAAAAAACAACAAGTTTCCTAGGAGCTATTATAGATGCACACATCAAGGGTGCAATGTGAAGAAACAAGTGCAAAGGCTAACAAGAGATGAAGGGGTGGTGGTGACAACATATGAAGGAATGCACACACACTCCATTGATAAGCCAACAGATAACTTTGAACAAATATTGAGTAGAATGCAGATTTATAGTACTCCTTTTTGA

>CmWRKY32

ATGGACCTTCTCCAGTGCAGGAATTTGCATATGGGTCCTCTTTGTTTGGGGAACCCATCTTTGGAATTGAACACAAATGCCAAAAGTTGTTCAATCCAACAAATACAATCTCAATCTGCTGCTCTTTATCATCATCACTTGCTTCAGAACAGGGTGGTGTTGAATAATAATCCTAATCCTCCGCAGCATGAAATGGTGTATCTTAGGAGCAATAATGGTGTTAACTTGAATTTTGATAGCTCTAGCTGCACACAACACACAATGTCATCCACTAGATCGTTTATTTCTTCATTGAGCATTGATGGGAGTGTGGCTAACTTGGATGGGAGTGCCTTCCATTTGATTGGTGCGCCCCGGTCTTCGGATCAGAATTCGTATCATAAGAGGAAGTGCAATGGGAGAGGGGAAGATGGGAGTGTTAAATGTGGAAGCAATGGGAGATGTCACTGCTCAAAGAAGAGGAAACATAGAGTGAAGCGGTCGATCAAGGTGCCCGCGATTAGTAACAAGCTTGCAGATATCCCTCCTGATGATTATTCATGGAGGAAATATGGCCAAAAGCCAATCAAAGGTTCTCCACACCCAAGAGGTTACTATAAGTGCAGCAGCATGAGAGGTTGTCCAGCAAGAAAGCATGTTGAGCGGTGTCTCGAAGAACCTTCAATGCTCATTGTTACATATGAAGGTGAACACAACCATCCTAGAATACCTTCTCAACCTGCAAATACTTGA

>CmWRKY33

ATGGAGGAGGTGGTGGCAGCCGATTCGCTACGGTACCCATTTCTCGATGGCAACGAGAGCAAGAGTTGTCTAGGGTTCATGGAGCTTCTCGAAGTCGATCGAGATTTTTCATCCCAATTTGATGTGTTTGAAACATCATCACCGTCTTTATCGTCTTCTTTGATTTCAAATCCTGAGAACTTGGAGATTTGGAATCAATGGCCTACCACGCCAAATTATTCGTCGTCGATCTCCTCAACTTCGAGCGAGATCGTTAATGGTGAGCTGACGGAGCCGAATCTAGAGGGAGGAGAAGAGAAACAAGATCGGCAACCAACTGTAAAAACTGACAAGCAGTTAAAAACAAAGAAGAGGAGTCCAAAGAAGAAAGGTGTGGAACCACGATTCGCATTTATGACAAAGAGTGAAGTGGATCATTTGGAAGATGGATATAGATGGAGAAAGTATGGTCAAAAAGCTGTGAAAAACAGCCCTCATCCTAGGAGTTACTATCGTTGTACTAGTGTAGCATGTCAGGTAAAAAAACGAGTGGAGAGATGTTTCCAAGATCCAAGCATTGTCGTTACAACCTACGAAGGTCAACACACTCATCCTAGCCCCATTATGGCACGGCCAACCTTCTTTCCTCCTCCCATCTCAGTCACCCTCTACGATGATTATTTAATTCAAAATAGTCATAATTCAAATGTTATGAGTCACTCAATTGCTTGGTGCCACCATTAA

>CmWRKY34

ATGGAAAGTGGGTGGAGCTGGGATCAAAAGTCACTCATTGGTGAGCTAATTCAGGGGATGGAGCTTACCAAGCAATTGAGAACAGAGTTGAGTTCAGCATCTGGAGAAGAAAGCAGAGGATCATTAGTACAAGGAATTTTATCTTCATATGAGAAAGCTCTTTTGATACTGAAATGGAACGGACCAACGAATCAGCTTCAGATGGTTGAAGCGACCCCTGGTTTGCCAGGCTCTCCAATTTCGACACAGCCCAGATGGACAGAACAAGTGAAAGTGAACTCTGAGACGGGATTCGAAGGACCCCACGAGGATGGTTATAGCTGGAGAAAGTATGGCCAAAAGGACATACTTGGTGCAACATATCCCAGAAGCTACTATAGATGCACTTTTCGCAATACTCAGAATTGTTGGGCAGTAAAGCAAGTGCAGAGATCAGATGAAGACCCTTCAGTATTTGAGATTACATACCGAGGAAAGCACACATGTTCCCAAGGGAACTATTTAGCCCAAACATGTCATTCACCAGATAAGCAAGAACAGAAGGAAACTGACCCCGATCCTCATGAGCTGCAGCCTTTGCAAGAAAACCTATTCGGTAATCAAACCATTCAGAACATCGAAAAGCTTGAAAATAAGGCATCTACCTTCTGCTTTGGCTCAAGCTCAACTTCTGTTGGATGTAAGGACATTGTAAATGCTGGCTTTTCAAATTTAGCCATCGACACTCACTCTGCTTTGGGAAGCTTTACTCGGTCATTTACCTCTCCAACCTCACCCGACAAAAACTACTTCACTCCATCCCCATGCCAAAGGAGCAACGTAGGAGGGACTCATAGTGTGCAAAATCTAGATCCTGATGTCCATGAGATTTTCTCAGCCAACACTTCAGCTACCAATTCCCCTATCCTGGATTGGGATTTTCCATTTGATTCAGAGCAGATCAATCCAAATTTCCCATTTAATTCCCAAGGTTTTTTCTACTAA

>CmWRKY35

ATGGGAGATAGTAATTATGATAATAATAATAATGTTGTTATTGGAGGAGTAAAAGAGGGTGATATTGTTATTAGTGGAGGGAATAATCATAATAACAACAATAATATTAAGTATAAAGGGAGAATGGTGATGGGTAAAAGAAGAAGTGCAATGGCTTCTCCAAGAATTGCTTTTCAAACAAGAAGTGTTGAGGATGTTCTTGATGATGGCTATAGATGGAGGAAATATGGTCAGAAGGCTGTCAAACATAGCAACCATCCTAGGAGCTATTACAGGTGTACACATCACACATGCAATGTTAAGAAACAAATTCAAAGGCATTCAAAGGATCCAACAATAGTTGTTACAACATATGAAGGGATTCACAATCATCCATCTGAGAAATTAATGGAAACTCTAACCCCTCTTCTCAAGCAATTGCAATTCCTTTCAGGCATTTAG

>CmWRKY36

ATGGAGGATTGGGGTTTGCAAGCAATTGTAAAAGGGTGCAACGGAATACCAATTGGTTCTTCAACAACAGCAGCAACAACAAGGCTTATGGAAGATACAAATAATTTGTATTCTTTTTTGAGATCAGATCAAGAAGAAGATGGAGGATTTTTTTCTTCTTGTGTTTATAATAATTATTATAATCCTCAAATTTCAAGTTCTTCAATATTTCATGATGAGTTTGAAGGATTATTTGGAAGGAATTCATCCAATAATTCAGCAGCTGCCTCCATTTCTCATCTTCTTAGAGATTTTAAGGAACCTGCTGATCAAAAGCTTCATCACAAGAACCAAATTATCCAACCAACTAAACAGAAACAAAGCAAGAAAAGTAGACAAAATAGGGTAGTGAAAGAGGTGAAAGCAGATAAAGTATGTTCAGATTCATGGGGATGGAGAAAATATGGACAAAAACCAATCAAAGGCTCTCCATACCCAAGAAGCTATTATAGATGCAGCTCCTCGAAAGGATGTTCTGCTCGAAAGCAAGTCGAACGTAGCCTCTCCGATCCCGAAGTCTTTGTCGTAACCTACACAGCCGAGCACAACCATGCCGAGCCTACTCGCCGTAACGCCCTTGCAGGGACCACCCGGAAGAAGTTCCCGGCGCTTGAGAACCCGAATTTGGACATGATTCTCTCACCTAATAATTCGACTTCAGTGGCATCCATTGAAGAAGATCAACATCATCCAATGGAAGGTGTGGCAGATGGGGAAGTTTTGATGGATATGCCATTTGAATTCTTCACTGGTTTGGAGGATTTACTTTTTGGATGA

>CmWRKY37

ATGCTCCTTCTTAACAATAACATGGCTGTGGAGCTCATGGTGGGATTTGGGGATGCTACAACTTCTAATAATTTCACCCCTAAGATGGAAGAAAATGCCGCTGTTTCTGCTGTTCAAGAAGCCGCTTCTGCTGGGATTCAAAGCGTCCAGAATTTCCTCCGATTGATGTCCCATACAACTAATCAGCAACACTCCCAAGACGATTCTTCTACTTCCACTACTCCTAATAATGGATATGAAGCCGTCGCCGATTCCGTCGTTAATAAATTCAAGAAGGTTATTTCTTTGCTCGACCGGAACAGAACCGGCCATGCTCGTTTCAGAAGGGCTCCGGTTCTTAGTACTACTACTACTACTACTCCGCCGCCTCCTCCTCCGCCAAAGGTCAAGCTGCAGCATCAAGATCCGAGTTCGTCGTCTCCGATTTCAGTACCTCCGATTCAAGTAAAGAAACAAGAATCAGTTTCCGCTTTTAAGGTTTATTGTCCAACGCCTTCATCTGTTGTGCGTTTGCCTCCTCTGCCTCACAACAACCCTCATCAGCCGTCTCATCCACCCAATACCTTTCAAGCCCAGCAAAATACTTCGTCGGTGGTACTCAAAAATGGGTCTGTAGATAGAAAAGATGCGACTACCACCATCAATTTTGCACCCTCGCCCCCAATTTCCGCTGCAAATTCGTATATTTCATCGTTAACTGGAGACACCGAAAGTTTACAACCATCTCTGTCATCTGGGTTCCAGTTCACCCACATGTCCCAGGTCTCTTCTGCCGGAAAGCCCCCTCTTTCATCCTCTTCGTTAAAGAGAAAATGCAATTCCATGGAGGACTCCGCCATGAAGTGTGGCTCATCATCCGGTCGGTGTCACTGTTCCAAGAAGAGCAGGAAAAACAGGATAAAAAGAGTCATCAGAGTTCCGGCCGTTAGCTCAAAACTTGCGGATATCCCACCAGACGATTACTCCTGGAGAAAATATGGCCAAAAACCCATCAAAGGATCTCCTCATCCAAGGGGATATTACAAATGTAGCAGTCTGAGAGGATGCCCAGCACGGAAACACGTAGAACGTGCCTTAGACGATCCAACAATGCTGATTGTAACTTACGAAAACGATCACAATCACGCCCACTCCACCGAAACACCTGCACCGCTTGTTCTCGAATCATCATAA

>CmWRKY38

ATGATGGATATTTTTTTGGACCTTAATGTGGATCCTAATTCTTCTTATGCTAATTCTACTATGGATGAGGCTCATCATTCTTCACAGAAAAGAGATCAATTTGATGGAGAAATCTATGGAGTTCAAGAAAAACTCTCACTGAGCTTGTCAAATAAGGGAAGCGACTCGAGCCCGACATTAGAACAGGAGTTGGATAGAAAAATCCAAGAGAACGGGAAGCTAAGTCAGATGTTAAGAATAATGTATGAGAAATACATTAATCTTCAGAAACAAGTGATGTATTTGTTAAGCAACCAAAAGCAAAACACAGAAATGGAAGGGGTTTGTTCAAGGAAGAGAAAGGCAGAAGGAGACCAAGAAGATTATGAGAATTTAGAAGGAATTTGCAGCACAAGAGATGAAGATTTTAACAGGTGGCTTAAGAGGCCAAGATTAAATGGAAATTCAAAGGTTTCTAAAGTTTTTGTGCAAAAAGATGCATCAGATCCAAGCTTGGTTGTGAAAGATGGGTATCAATGGAGGAAGTATGGGCAAAAGGTTACAAGAGACAATCCTTCTCCAAGAGCTTACTTCAAATGCTCCTCTGCACCAAATTGTCCTGTCAAAAAGAAGGTGCAAAGAAGTTTGGAAGATCCAACAATTTTGGTGGCAACTTACGAAGGAGAACACAGCCATGCTAGCCATTTCCAAACTGAGCTTTCTTTAAGGTCAATCAATGGCGGCAAAGGCAGCGCAGTCCCGGTCTTAGCGACGATCAAGCCGTCATGTGCCACCGTGACCCTCGATTTGATCCACGAAGACGGGCTGTTTAAGAGTCCGAAAGATTACGCGTCATCGGAGTCGGCCGAGGCAGCGGTTTGGCAGGAGTTTTTGGTACAACAAATGGCCTCTTCTTTAAAGAAGGATCCTGAATTTGCTGGCATTGTTGCTGGTGCCATTTCAGGCAAAGTTTTGGGAAACCAAACAAACAGAGAATAA

>CmWRKY39

ATGGAGGCTCAACAAGCTTTGGCAACCATTGATGATGATAGAGTTCAATCAACTGGAGAAGGAAATGACGAAGCTTCTCCCAACTCTAAACAACGTATTCATCTTAAGGTCGTCACTTCTATGGGTGACTTGGAAAAGCCCTTCATGGAAACTCTTTCAGTACCATCAGCTTCTAATTCAACTTGGAAGGAAGAGGATGATGAGCAACATCATCAACATCATGAAGAAAGAATGAAAAGGACGAAAGTTGAAATGAGGGAAGTAAAGGAAGAAAACGAGAGGTTAAAGAAATATTTGGATGAGATAATGAAAGATTATGAGACATTGAAAAGGAAATTCCATGAGATTAAGTATAATCATGATCATGATCAAATTAGAGAAGATCATCAAGGCAAAAGATCAACACAAACAAGTGGAAGTACAATAAATAATATTAATAACAATGATGATGATGATCATCATCATCAAGTTGAAGCTGAAGTTGATGACATGGTTTCACTCACATTAGGATCATCAAGATTCTCAACCCATCATCAAAACAAGAACACTTCTTCTTCTTCTTCTTCCTTCTCTCTAACTAATAAGATCTTAGATCTCAAACAAGATTATGTTATTCAAACCCCATCAATTATTGATCACAATATTCATAGTCCAACCCATAGTGAACCTAAAGATCAGGAAGAAGCCGGACAGACGACTTGGCCCCCGAGTAAAATGTCGAAACCTGGCGGTTTGCCCTCACCTGCTATCGGAGAAGATGAAGTTTCTCCACAGAATCCTCCTAAGAAAGCTAGGGTTTGTGTTAGAGCTCGATGTGATACTCCAACGATGAACGATGGATGTCAATGGAGAAAATATGGACAAAAGATAGCAAAAGGAAATCCATGCCCTAGAGCTTATTATCGATGCACCGGTGCACCCACTTGTCCCGTTAGGAAACAGGTACAAAGGAGTGTTGATGATATTTCCATACTAATAACAACCTATGAAGGAACTCACAACCATCCTCTACCTGTTTCAGCCATGGCCATGGCTTCCACCACCTCCGCTGCCGCTTCGATGCTCCTCTCGGGCCCTTCCTCCTCCTCCTCTACCTCCTCCCAACCTGGCCTAAACCATTCATGTACCGCTGCTGCGACCGCCGTGAATCTTCATGGAATGAACATGTATCTCTCTAACAACACCAGTTCAAAGCAATTCTATTTGCCAAACTCTTCAATGCTATCTTCTTCTCTAAACCATCCCACAATCACTTTAGATCTCACTTCAAATCCTCCTTCAACTTCCTCCTCTTCTCCTTTCCACAAAATTCCTTTAGTTAATAATAATAATAATTATAATAATTATCCCCTTCAAAATATCCCTTCACAAATCTTGATTTTGCCTCTTCACAACCCAATTTCATATTTTGCTAATAAACAACTTCCTCTTCATACTAACATCTACCAAGCTTATCTTCAACAAATTTCTAAATCTTCAATGACTCCGCCGCAACCGGCTCTACCGTCAGATACGATAGCAGCAGCAACTAAGGCAATTACATCGGACCCGAGTTTCCAATCGGCATTGGCGGCTGCGTTGAGCTCGATTATCGGAGGCGGGGAGACGGGTCCGAGTGTTTCTAGCTTAGTTGTTGGAGGAGGAGGAGGAGGACAAGGAAGTATGGGGTTTGAGGCTGCAGCTAAGTCATTAACATGCTCTACTTCTAAGAGTACTCCTTCTTCATCTCCTGGGGACAGTAGAGATAATGGGAAGTGA

>CmWRKY40

ATGGCGGTCGATCTCGCAGCTTTTCCGACCATCTTCGACGATCAAACCGCCATTGAAGAAGCCGCCACCGCGGGCTTACAAAGCATGAACCATCTAATCCATCTCTTGTCCAAACAACAACAACAACAACAACAATACCACTCCGAATCTCCTAATAACATCGATCTCAATTCTTCTCTTCTCACCGATTTCACCGTCTCCAAATTCAAACGATTGATTTCCCTTTTAAACCGAACCGGCCACGCTCGGTTCCGCCGTGGACCTTCCGATTCTCCTAATCCCGTTCTGAATTCCCTCGATCCACCTCAGAAAACCCATTTCTCTAAACTTAATTTCTCCCCTGTTTCGAAAATCCCTGAGTCTAGAGATTCCACTACTACCTCTTCTTTTGTGTCTACGGTCACCGGCGACGGTAGTGTTTCTAACGGTAAGCTTGATCTATCGGTCTATACTACGCCGCCTGCCAACGGCGGTAAACCGCCGTTGGCGATGAAGAGGAAGTGTAATGATGTATCTGGATTTGGTTGTAAGGTTCCTAATTCGAAGCTATGTCACTGTGCAAAGCGAAGGAAATCTGGAATGAAGAAAACTGTAAAGGTCCCGGCGATAAGTTCTAAGATCGCTGATATACCCTCCGATGAATACTCTTGGAGAAAGTACGGCCAAAAACCCATCAAAGGCTCACCTTATCCTAGAGGGTATTATAGATGTAGCACCGTGAAAGGATGTCCGGCGAGAAAGAAGGTAGAAAGAGCTCGAGACGATCCGTCGATGCTCCTCGTAACCTACGAGGGCGATCACCGTCACCCGCACCCTATGGTCGCCGACGCCAGCGTGGGTGTCGTTTCCTAA

>CmWRKY41

ATGATGGGGGAGAGGGGAGGGGGTGATTGTGATAAGAAAAAGAAGATGAGAAGTAGGAGATTTGCTTTTCAAACAAGAAGTCAAGTTGATATTCTTGATGATGGCTATAGATGGAGGAAGTATGGACAAAAGGCTGTGAAGAACAACAAATTCCCAAGAAGCTACTATAGATGTACACACCAAGGTTGCAAAGTGAAGAAGCAAGTTCAGAGGCTAACAAGAGATGAAGGGGTGGTGGTGACGACTTATGAAGGCATTCATTCTCATCCCATTGAGAAATCCACTGATAATTTTGAGCATATTTTGTCTCAAATGCAAATCTACACTTCTTATTAA

>CmWRKY42

ATGGAGGTTTCTTCTCATCATTCTTTCAAACCAAACCCAAATGATGAACATCATCTTCTTTCAACTCAACCCACACCAGAAGAAGAACAACCCCTTTCCAAAAAAAGAAAGGTTGTTCAAAAGACTGTGGTTACTGTAAAGATTGGGTCTAAAAAAGCTGCAATAGGGATAGGGAAGATGAAAAATGAAGGTCCACCTCCTGATTTTTGGTCTTGGAGAAAATATGGCCAAAAACCCATCAAAGGATCTCCATATCCAAGGGGTTATTATAGATGTAGTACAACAAAGGGTTGTTCAGCAAAAAAACAAGTTGAGAGATGCAAAACAGATGGTTCAATGTTCATCATAACATACACTTCAAGCCATAACCATCCAGGTCCCAACATCTCTACTCTCAATTTGGATCAAAATCAACAAGAGATTGATCAACCACAGCCGTTGGATCAAGATGATGATGAAGATGATGATCTTGTTCCAAATCAAGAACAAGATCATAATAACAACAATAATGATCATGAGAAAAACAGCATTATTGTTAGTCAGTGTGAAGAAGAAGTAGAAGTAGAAGTAGAAGAAGGAGAAGAAGATGATGATGATGAATTATTATTATTAGAAGATGAAGAGAAAAAAGGAATGGAAAAAATAAAGGATGAGTGTTTGGATCAAGAACCAATAATTTCTTCTTCTTCTTCTTCTTCATGTTGTGATGAGGTGATGATCATAAAAACAAAAAAATCAGAAATAGAAAATCATGATAATTTCTTTGATGAGCTTGAAGAATTGCCCATTCCTCCACCTTTTTCAAGCACCTTGATGAGATCAAGCTATTCTTTTGATGAAATTAGGATTTCTGCTGCCCCTTCTTGATGTTAATGCTGTTTTTCTAGTGCTTAATTCCCTTTCTGTTCTTAG

>CmWRKY43

ATGGACCGCCGTGTTCGTACAAACCCATTCCTCTCCGAACAGGAAGATCCCGAACCCACGTCCGACGACGGCTTACCCGAATCACCCTCCGACGCCAATGACTCAAAACCCACCGCTGCTCCACCTCCTAAAAAGAGTAGAAGGGGAGTGCAGAAGAGAGTGGTGTCCGTACCGATCGCTGACGTGGAAGGATCTAGGAGCAAAGGGGAAGCATATCCACCGTCCGATTCATGGGCATGGAGGAAATACGGCCAAAAGCCAATCAAAGGCTCTCCTTATCCGAGGGGATATTATCGATGTAGTAGTTCCAAGGGATGCCCAGCAAGAAAGCAAGTAGAGAGAAGCCGTGTGGATCCCACCAAGCTCGTTATTACCTACGCCTTCGACCACAACCACCAACTCCCGGTCACCAAATCTCACCACCACCACCACAATTCCTCCCCGTCCTCCGCCGTGGTCGCCGCGGCATCTGCCGGAACGGACTCTCCCTCGCCAGGGAGCACTACGACGTCGTCCTCCACTTCCTCCGGCGACAACACCAACGCGGCTCCGTCGTCACCGGCGGCGAAGTTTGAGGAGGTGGCTGCGGTTTTCGCGAGTCAGCCGGAGCTGGAACTCGGGGGCGACTCGCTGATGATAAAACCATGCATCGGAGATTTCGGGTGGTTGGGTGAGGTGGCATACGACAGAATCCTGGAGGGCCCGATTTGCGGGGGAGGCGACATATTCGACGACGCCGATGTAATGGTTTTGTCCACAAGAGGAGATGACGAGGAGGAGTCGTTATTTGCGGATCTTGGGGAATTACCCGAAGGATCGGTGGTTTTCGGCCGACGGCGAACGGTCCAACCGGACGGACCGAATCGGACATGTGGCACAGTTTTAGACTGCTAG

>CmWRKY44

ATGGCACCCCCACAAAACAGAAAAGCTAGGGTTTCTGTAAGAGCAAGATGTGAATCTGCCACCATGAATGATGGTTGTCAATGGAGGAAATATGGTCAAAAAATTGCGAAGGGAAATCCATGCCCTCGAGCATATTATCGCTGCACTGTTGCACCGGGATGTCCAGTTAGAAAACAGGTACAAAGATGCTTAGAAGATATGTCAATTCTAATAACAACCTACGAAGGAACACATAATCATCCTCTACCTGTTGGAGCAACAGCCATGGCTTCCACAGCTTCAGCAGCTTCTGCTTCCTTTATGCTCTTAGACTCATCTAATAATAATAATACCAATCTTTCAAATTCTCTTCATCAAAACCCTAATATTCTTAATTCTTCTTCTCCTTCTTTCCTCCAAACTCAAAACCCTAATAATCATCTTTTCACCCCATTATTCCCCACCTCCTCAACCTCCCACTTCCCCCATTCCTTTTACCATTCCAACTTTCAACCTAATCATCTTGTCAGTCCTCTTGACCGTCGCACCTGGAAACCGGTCGATGATAATAAGCCACCACCGTTGACGCCCGATGCCGTGTCTGCCATTGCTTCTGACCCTAAGTTTCGAGTTGCCGTTGCGGCCGCTATTTCTTCGCTCATTAACAAAGAGAATGAACACGTGACTACAACGGGAGAGACGGCCACGGATGGTAAAGGTGGTGGTGGCAGCGATAGTGATAGTGGAAGCAAGAAATGGGTTGTTGAATCCCTCTCATCAAAATCAAATGGTAATTGA

>CmWRKY45

ATGTCAAATGAAGGTCATGATCATGAGAAGGAAGAGGATGATCCTTATTACCCACAATTTGATCCTTTCACTCACAACTTTCATCATCAAAAACCCTTTGAAGTTCCTCCTCCTTTGACAAACCCTTATTCTGAAGCTTTTGATATTGCTCCTTATAATGTGGGGTTTTATTCTGATTTCTCACATGCCTCATCTCAATATGACTACCACAACATCTTCTCTACTCCCTTTGAGATGTCGTGCTCGTCGTCCGAAGTTATTTCTTCTGTCGACGATGCTTTGAAGAAGTCGTCAAGTCTCGGTCGAGATCTGTCATCGGTGGTGACTGGTGAGCACCCATTGACACCGAATTGTTCCTCTACCACGTGCTCCTCCGATGAGGTTGTCGCGGGTGGTGGTGATTCTTCCAAGAGCGGAGAGGTGAAAGGGTTTGATGATAAAAAGGGAGAAAATAGCAAGAAAGTGGATGAAGGAAAAAAGAAGGAGAAAAGAGAAAAAGGGCCCCGATTCGCCTTCTTGACCAAGACCGAAATCGATAATCTTGAAGATGGATATAGGTGGAGAAAATATGGTCAAAAAGCAGTCAAAAACAGCCCTTTTCCAAGAAGTTATTATAAATGCACAAGTCAAAATTGCAGTGTGAAAAAAAGAGTAGAAAGATCATCGGAAGATCCAGGTTTTGTTATAACAACATACGAAGGCAAACACAACCATTATTGTCCAATTACACTTCGTGGCCATAATCCCACCGGAGTCTTGCCGCCCTCCGTCACTCCGCCCTTGTTTCCCCCGTCGCCCAACTTCTTCTCCGCCGAGTCCTTCTGTGAAAATCTGCACCAACAATATCAGTATGGCCTCTTCCAAGATTTTATCAATAATCCATCATTTAATAATTCAAACCAACACCCATCAAATTAA

>CmWRKY46

ATGGAGGCGCCGGAGTTTTCTCACGGCCGTATTTTGCCGGCAGAGCTAAGGAGGAAGATCACGGCGAAGCTTCTCCCTGGCCAAGACTCAGCAGCTCACCTTCAGACTCTTCTACAGTCAGCCGCCGCCACGGAGCAGGACAAGCGAGCTCTTGCCACCAAGATCTTGACTTCCATCACCGAAGCGATCTCCATCCTGGAGTCCGCCGCTGGTGAGGAGTTGAGCTGTCCTGATAATTCCCTTTGCTCCGATCTAGATTCCGACGACTCACGGCGGAGCACGGGGGTTAAGAATAATCCATCACGTGCCAACAAAAGAAGAAGATCGATGAACACGAGATTTGTTAGGACGTCGAGGACGACGGAAGACGAGTATGGTTGGAGAAAGTACGGCCAAAAAGTTATCCACAACACAACTTATCCAAGGAGCTATTATAGATGCACTCACAAGTATGATCAAGGTTGTCAAGCCACGAAGCAAGTACAGAGAATGGAAGGTTCTGATTCAGAAGCAATGTACAAGATCACCTACATATCTGACCACACATGTCGCCGCCCTGCCTCTCCGATCGACGCCTCCGCCATTGCCACCCCCTCCGATTCTTCAAACCTCATATCTTTCTCCAACGATTGTAACGCTCCATTGACCGAGGGTACTGGCTATAGTTTGATCTCATGGCCGAGTGATCATGACGATGCAATCAAGGTGGGAGAGACAGCCACGACGAGTGGTTCCACAGGTCATGAGATTGATTTGTGGTCGGATTTGAAGGAGTTTGGGAGCTTCCAAACAACGGCTATGACAACCACGACGGCGAATCCATACTATTTCTCAACCGCAGATGACGATGCAGATTCTCTCATGTTTTGGGAATTTTGA

>CmWRKY47

ATGGCCTCCTCTTCCGGGAGCTTAGACACCTCTGCTAATTCTCATCCCTCCTTTACTTTCTCTACTCATCCTTTTATGACTTCTTCTTACTCTGACCTTCTTGCCTCTGCCAACAACGATCCTCCTTCCTCCGCTCCTCTCCGCGGTTCCGGTACCGGAGTTCCTAAATTCAAATCCCTCCCTCCCCCTTCTCTCCCTCTCTCTCCTCCCCCCATGTCTCCTTCTTCCTTTTTCGCCATTCCTCCTGGCTTGAGTCCCGCCGAGCTTCTTGATTCCCCTGTTCTTCTCAATGCTTCTCATGTTCTGCCGTCGCCGACCACTGGGACTTTCCCGTCTCACTCGTTGAATTGGAAGAGCAATTTTGGATATAATCAGCAGAACATTAAGGAAGAAAACAAATATTCGTCTAATTTCTCATTTCAAACTCAATCTTCAAAGCTCCCGCCGACGTCTTTTCAGCCTTCATCCACCACAGCTCCCACGACTCAGGGATGGAGTTTTCAAGAACAGCGCAAGAAGGAGGATGGTTTTTCGTCCGAGAAGAATATGGTGAAGCCGGAGTTTGGATCGATGCGGAGCTTCTCACCGGAATATGGGGTTGTTCAAAACCAGAGCCAAAACAACAGCAGCGGGGAGTTGCAGTCTGATTATGGCAACAATTACCCTCAACAATCTCAGACGGTGAATCGAAGGTCTGACGACGGCTACAACTGGAGAAAATACGGCCAAAAACAGGTTAAAGGAAGTGAAAATCCGAGAAGCTATTACAAGTGCACTTTCCCCAATTGCCCAACAAAGAAAAAAGTTGAAAGATCCTTAGATGGACAGATCACGGAGATCGTTTACAAAGGCAGCCATAACCATCCCAAGCCCCAATCCACGAGGAGGTCGTCGCTGTCGTCGGTCGGTTCTTCTCAAGCGATAGTGGCTTTGAATCAGGCCGCTAACGAGATGGCGGACCAGTCATTTACAACCCAAGGCAGTGGCCAATTTGACGGCGTTGCAACACCGGAGAATTCCTCGATTTCAATCGGCGACGACGACTTCGATCGGAGCTCTCAAAAGAGCAAATCCGGAGGGGACGATTTTGATGAGGAAGAACCAGAGGCTAAGAGATGGCGAAGGGAAGGTGACAACAATGAATGTATTTCGGCTGCCGGCAGCCGAACGGTGAGAGAGCCGAGAGTCGTCGTCCAAACTACCAGCGACATCGACATTCTCGACGACGGTTACCGGTGGAGGAAGTACGGCCAGAAAGTTGTGAAGGGAAACCCAAATCCAAGGAGTTACTACAAATGCACAAATCCAGGATGTCCAGTAAGAAAGCATGTAGAGAGAGCTTCACATGATCTAAGGGCAGTGATAACAACTTACGAAGGGAAGCACAACCACGATGTTCCACCGGCACGTGGCAGTGGAAGCCATTCCCTCAGCCGTCCATTCCCCAACAACGATCCTCCGGCCGCGATTCGCCCATTATCGGTAGTGACCCATCAGTCAAACAACGGGGGGCATCCGCAGGGTCTGCGGCTGCAGCAGCGATCTTCGGATTCCCAATCAGCGTTCACAGTGGAAATGGTACAAAATGGGAATGGATTTTCATTCCCAGAATTTGGAAACTCAATGGGAATGGGATCCTACATGAACCAAACACAGCCCAGTGACAATTTGTTCACAAGAGCCAAAGAAGAGCCAAGAGATCATGATATGTTCATCCAATCTCTCCTATGTTAA

>CmWRKY48

ATGGCCAGCTTCCCTTATGAAGATTCAAACCCTAACCCTAATAATCTTAACCCTAACCCTAATCATTATTATACTCCTTTTCCTCCGATTCTCGATCCCGCTTCTTTCTTCGATTTTGAGCTCTCCGATTTCCTTTTGTTTGACGATAACAACAACAACAATAATAATAATATCGTCGACCAAGCTGCATCGTCCTCGCCAAGTATGACTTCGTCGGAGAAGATCACTGGTGGAGGAGGAGTCGATAGTAGTGGCAGCTCTACTGTAATCGATACCGGATCTAGCATAGTAGTTTCATCGGGAGCGTCAACGACATCTATAAGAAGCAAAAATGGGGAGAAGAAAAGGAAAGGGGAAATGGGATGTAGAGTTGCATTTAGAACCAAATCAGAGCAAGAAATTATGGATGATGGCTACAAGTGGAGGAAGTATGGGAAAAAATCTGTGAAGAATAGTCCTAATCCCAGAAATTACTATAAATGTTCAAGTGAAGGATGCAATGTGAAGAAGAAGGTGGAAAGAGACAGAGAGGATGCAAACTATGTGATTACTACTTATGAGGGAATTCACAACCATGAAAGCCCCTTTGTTGTGTATTACAATCAATTGCCATCCTTTACAACCTCTACCCCCACCTAG

>CmWRKY49

ATGGGTTCAAAATCCCAAGTTCTATTGAACCCACAAGCTTTGTTGGAAGATCATCAAGAAGTGACACCAAATTCTCAAATGGGTTTCTTCAATTTCCCTTCAAATTTAACCTTTTTTCAACTTCCTTCAATCCCTCAAACCCATTCTCCTTCACCTTCATTTGATCCTCCAAATTTCTCCACTTCCAATAACAATACCAATAATAATAATAATAACAATTCCAATAATCTTTCTGAAACCCTACTTTCTTCTTCTATTTTGCCTCTCAAATCCTCCATTTCCTATGAAATTGCACCCCAACATCTTCTTTCCTTGCAAACATCCACTCCAAATCTATGGCCATGGGGAGAAATTGGAGAAAGACTATTGATGAATGGGAAGAGATCAAATAATAATGAGAATAATAATAATCAATTGGGAGTATCAAAAATGAAGATGAAGAAAATGAAAGGAAGAAGAAAAGTGAGAGAGCCAAGATTCTCATTCAAGACCATGAGCGATGTTGATGTTCTTGATGATGGTTACAAATGGAGAAAATATGGACAGAAAGTGGTAAAAAACACACAACATCCTAGAAGCTATTATCGTTGTACACAAGATCATTGTAGGGTTAAGAAAAGAGTAGAGAGATTAGCTGAGGATCCAAGAATGGTGATTACAACTTATGAAGGCAGACATGTTCATTCTCCTTCCCATGATTCTGAAGATTCTGAAGCTCAAACCCATCTCAATAATTTCTTTTGGTAA

>CmWRKY50

ATGGCTAAGAAGGATGATTCTGCAGCAAGGCCTCCACTTCAACGCCCCACTATTACTCTCCCTCCTCGTCCTTCTATGGAGGCTTTTTTCACCGGCGGTCCCACTGGGGTTAGCCCTGGTCCTATGACTCTTCTTTCAAGTTACTTCGCCGACGGTGCCGTTGACTCTCCTTCCTTCTCCCAGCTTCTCGCTGGAGCTATGGCTTCTCCGATGGCTATGGGGTTTTTCGGAACTGGGTCTACTCCCAATTATTATGCCAAGGATGGACCCGGCTCGGAATTGGAATTTGGGATGAAACAATCGAAGCCGGTGAATTTAGTGGTAGCTCGTTCTCCTTTGTTCTCGGTTCCTCCGGGGCTTAGTCCTTCGGGGTTGCTTAACTCGCCGGGGTTTTATCCTCCTCAGAGTCCATTTGGAATGTCGCATCAGCAGGCATTGGCTCAAGTTACTGCCCAAGCAGCATTGGCAAATTCTCATATGCATATGCAGCAAGCGGAATATCAACATTCTTCGGTACCAGCTCCTACAGAACCGTTAGCTCGTGATCCATCATTTTCTCTTGATGAAGCATCTCAACTGGCCATAATACCCTCCACATCAGACACTAAAAGTCTGATTGCAGAATCGACAGAAGTTTCTCATTCTGATAGAAAATATCAACCTCCTCCTCCCCCCCATGGCTCCGATAAACCTGCAGATGATGGCTATAACTGGCGTAAATATGGACAGAAGTTGGTTAAGGGCAGTGAATTTCCACGAAGCTATTACAAATGCACTCATTTGAATTGTCCTGTAAAAAAGAAGATTGAGCGCTCACCTGATGGTCAGATTACTGAAATTATTTACAAAGGCCAGCACAACCACGACCCTCCTCCAGCCAACAAACGTGCAAGAGATAATGTTGAGCCAGCTGGATGTACAAATTCATTGATTAAGCCTGAATGTGGCTTGCAAAACCAAGCTGGAATTTTGAACAAGTCAAGTGAAAATGTGCAATTAGGGTCTAGTGACAGTGAAGGACGAGCTGATACAGAGATAACGGATGACAGAGATGAAGATGAGCCAAATCCAAAGCGGCAGAACATTGACGCAGGGACGTCAAGTGTTGCCTTGTCACATAAAACACTCACAGAACCAAAAATCATTGTCCAAACAAGAAGTGAGGTTGACCTGTTAGATGACGGTTATAGGTGGCGCAAGTATGGACAGAAAGTGGTTAAAGGAAATCCTAACCCTAGGAGCTACTACAAATGCACTAGTGCTGGGTGCAACGTCCGAAAGCATGTTGAGAGGTCTTCAACAGACTCGAAAGCTGTTGTAACTACATACGAAGGGAAACATAACCATGATGTTCCTGCAGCCAGAAACAGCAGCCACCACACTGTCAACAACACTGTCCACCATATCAAACCACTCAAAGTTGTAGCTCAGAAACATCCATTACTTAAAGAGATGGAATTTGGAACTAATGACCAGAGACCTGCCGTTTTGCAGCTTAAAGAAGAACAAATCACTGTGTAA

>CmWRKY51

ATGGAGTTTCTCTCTCTAATTCAAGAGCTTAATCAAGGCAAACAACTCGCTAACCAACTCCGTAACCATCTTCATCCTTCTTCTTCCTCCCATGGCATTCTCTTAGTCGATAAGATTCTTCGTTCCTACGAAAACGCGCTTTTGGCTCTCTCCGCCGGTGGTGGTGCTTCCGTCAATTCCGCCATTGCTCCGGTCAATGCGGCTGTCAAAGATGGAGATGTATCCAAAAAGAGAAAGTTAATGGCGAAATGGAGTGAACAGGTTAAGGTTTCCTCTGGCTCTGCTGTTGAAGGTCCGTGTTGTGATGGATTTAGTTGGAGAAAGTATGGTCAGAAGGATATTCTTGGATCCCAATTTCCCAGAGGCTATTTTAGATGCTCACATCGCTTTACACAAGGATGTTTAGCGACAAAACAAGTTCAAAAGTCAGACAATGATCCAACCATTTATGATGTAACTTATAGAGGAAGACACACTTGCAACAAAGCCCTTCATTCAACAAACACACCACAAGAACACCAAAACTCATTACTTCAACACCCAATCCCACCAAAACAAGAAGAAAAGCCATTGCAGCAGTTACATGATCCTTTATGTTTTATGTTCAGCTCCGACCCCATTCGAGTAAAATCCGAGAACTTAGACAATGCCAATGGTGGCCTCCTTCAACCATTTTGTCCCCCATCCCCGATGTTCGGTTCTGAAGTTCAAGATGATCAAAGCCCTTTTAGGGAAAGTGAGTGTTCTCTGACGTTTGAATCGAATGATACGTTTGGATTGTGCTGTGATTTCGAAACTGGGTTTGTGTCGATTCCTAGTTCAATGACTAATATTTCGATTGGAGACTTGGAGGAGTATTGTAGTTTTGATAACTTGGAGATGTTCTGTTGA

>CmWRKY52

ATGGTTTCTACAGGGGATCAGTTAGAGAATGGGGTTGATTCCGATCAATTGGATCATGAAAACAGTTCAGATAGTCAACCGCAAGCGTCTCAGGACGATCCTGGTGGAACTAATGCTTCGAAATCAGATCACAAATGCACAGGGGCTTCCAGTAATACACTTGAAGAAGCTGTCAAGCAGCCTGAAGTTACAATAGCACTAGTGGATCGAGGAGACATCTCTAATATAGTGACTGAGAAAGTGACCCATAAGCCAATTACTGCAGAACAGAATCCCCTATCTGTTTTGAAAGTATGTATTACCTCCAGTATACGAGAGAAAGTATCAGAGGATGGATATAACTGGCGGAAATATGGTCAGAAACTGGTTAAGGGCAATGTCTTTGTTAGAAGCTATTACAGATGTACGCATCCTACTTGCATGGTGAAAAAACAACTGGAGCGTACTCATGATGGGAAAATCACAGACACCGTTTACTTTGGTCAGCATGATCACCCTAAACCTCAACCTCATATTCCAATTCCTGTTGGAGTCGTTACCATGGTTGAAGAAAAATTAGGTGAACATGCTTCTGGAAATTCTCAAGATAAAACCTCCACTACGCTTAGCCCAACTCCTCAACAAACGGAGCTTACTGATATGCGTCAACCGCCATCAGTTATAGCCAGTGATAATGTAAAAGATGAAGCTTCGAAAAGGTCTCGGACTAATGATGAAGCTTCGAAAAGGTCTAGGACTATTGATGAGATTGATAGTGATGATACACCAGACCTGAAACGAGATTTCAAAAGGAAGAAAAGATGTAATATTGACGTGACGACTGTAGCAGACAAGTCAATTGTTGAATCTCGGGTTGTTGTTCAAACTCCTAGTGAGGTTGACATCGTCAATGATGGATACCGCTGGCGGAAGTATGGACAGAAATTTGTGAAAGGAAATCCAAATCCTAGGAGTTACTACAGATGCTCAAGTCCTGGATGTCCAGTTAAGAAACATGTAGAGAGGGCGTCTCATGATCCCAAAGTGGTGCTTACCACATATGAGGGCCAGCATGACCATGTTCTGCCACCTATTAGAACAGTGACGTTGAACTCAGTTGGATCCACAACAGCCCATAGTGACGAAACAAAACCAAAACCAGTTAGTACGGTGGTTCATGCTAGTAAGGATCCGCAAAGCGATTCAAGTTCTGAAGGCAAATTGATTGAAGAGAATGGGAAGTTGAATGCAACAGAAACAAGTGATGACATTATACTCGATGGGGTGGTGGTAAATCCTAGCCCAGGAGTTGCGAGTGAACAAAATAAGCAGCTGAAAGTAGCAATCGAAAGCTAG

>CmWRKY53

ATGGCGGTGGAGCTCCTCACCGGCTTCACCAACCCTCCTCAACTCTCTTCTCGATCCCCCATGGACCAAGACTCCGCCGTTCAAGAAGCCGCCTCCGGCCTCGACACCCTCAAAAAGCTCATCACATTACTCTCTCATTCTCCCCCTTCTAATCTCGACTCCGATTGCCAAGCCGTCGCCAACGCCGCCGTCTCCCACTTCCGCAAGGCCATATCTCTCCTCGGCCGCTCCTCTAGAACTGGCCATGCCCGATTCCGCCGTGCCCCTTTAGATTCCTCTAAAATCTATAACGCTACCCCCATTCAACAAATCCCTCCGCCCTCCCTCGACCGCCTCGATTCTGCCACCACCATCAATTTTTCTTACTCCGCTGCTCCTACTTCCTCCTTCTTAACCTCCCTTCCTGCTTCCGATTCAGAAATTAAGCTCCAACATCAACCCTCTTCCTCTTCTTTTCAGATTACTGATCTTTCCAGAGTTTCGTCTGTTGTCTCTAAGCCTTCTTCTGGTCTCAAGAGAAAGTGCGGTTCTGAGAATTTGGGCTCTGGGAAATGCGCTGGATCTTCTGGTGGTCGTTGCCACTGCTCTAAGAAAAGCAGAAAATTGAGGTTGAAAAGGGTGGTGAGAGTACCAGCAATAAGTTCTAAGAACGCTGATATTCCTCCGGATGATTATTCGTGGAGGAAGTATGGGCAAAAGCCGATAAAGGGTTCTCCATATCCGAGAGGTTACTATAAGTGTAGCAGTTTAAGAGGATGTCCGGCAAGAAAACACGTCGAGAGAGCGTCAGATGATCCATCTATGTTAATAGTAACGTACGAAGGCGATCATAATCACTCTCAATCAGTTGCAGAAGCCTCAAGTCTCATCCTTGAATCTTGGTGA

>CmWRKY54

ATGGAATTTTTTTTTTTCTCTGCTACATTAGGTGATCCTAGTGGCGGCGGCCGGCGGAGACAGCCGGACCTTGCGGTGGTGGGCAAGGGTAATCCGCCGTTGTCTCCACCACTTTACTCTCCTTCTTCCTTCTTCACTATTCCTCCTGGTATCAGCCCCACCCAATTACTTGATTCCCCTCTTCTTCTCAACTCTTCTCCTATTCTGCTATCGCCGTCCACCAGAGATTTGAGAAAATCAACGACTAACAATTCCGGCCATCACCAACAGAACATCAAACAAGAACACAACATTACTGAATTCTCATTTCCTCGTAATCACACCACAAAATCATCATCATCATCAATGTTTCAATCCTCTTCAACAGTGCAAACTGAGGCATGGGGTTTGGAATCGGAGAGCGACCGGGGAGAATGGGAGATGAGAAACAGATCAGGATCTGAGGATGGGTTTAATTGGAGAAAATATGGTCAAAAAGTGAAGAAGGTCATCTTCCTCTTTTTCTTTTCTTCTTCTTCTTCTTCTTCTTCTTCTTCCTCCTCTTTTGAACCTGTAACAATGGCCGCACAGCTAAAGATCAATTCAATTGCCTCTCACTCTCTAACCACGCCGGAGAACTCTTCAATTACCATTGGGGATGACCATTCCGACCAAGCTCATCCAAAAAGATGGAAGAGTGAGAGTGAGAAGGAAATTATGACAACAGGAGGAGGGGGGAAAACAATGAGAGAAGAAAGGATTGTAGTTCAAACAATTAGCAATGTGGATAAATTAGATGATGGTTACTGGTGGAGAAAATATGGACAGAAAGTGGTAAAGGGAAATCCAAATCCAAGGAGCTATTACAAGTGCACATATCCAGGTTGTGGAGTGAGGAAGCATATAGAGAGAGCATCCCATGATCTTAGGGCAGTTGTCACTACCTATGAAGGGAAGCACAACCACGATATTCCGGCAGCCCGTGGCGGCAAACCCATACCTTAG

>CmWRKY55

ATGGCTGCTTTCTCCTCTTGGTTGTTGGATTCTGTTGATACTAAATTAGATCTCAATTCTATGCCTCTCAGATTCTCCGGTGAACCTCCGAAGGAGAGGAATTATATGGATGTCGAGAGGAAGGTTACTGTCAAAGAAGAGACTGGAGCTTTAATGGAGGAGTTGAAGAGGGTTAGTGCAGAGAACAAGAAATTAACTGAAATGTTGACTGTTGTTTGCGAAAATTACAACACTTTGAGAGGACATTTGATGGAACAAATGAACAAAAATGGAGAAAAAGAGATTTCTTCTTCAAAGAAGAGGAAATCAGAGAGTAGCAATAACAATAACAATATGGTTGGAATGAATGGGAATTCTGAAAGTAGTTCAACTGATGAAGAATCTTACAAGAAACCAAAAGAAGAAACCATTAACAAATCAGCTAAAATCACTAGAGTTCAAGTCAAAATTGGAGCTTCTGATTCAAATCTGGTTGTTAAAGATGGATATCAATGGAGGAAATATGGTCAAAAAGTCACTAGAGATAATCCTTGTCCTAGAGCTTATTTCAAATGCTCTTTTGCTCCTAGTTGCCCTGTTAAAAAGAAGGTTCAAAGAAGTGTTGAAGATCAATCAGTTCTTGTAGCCACATATGAAGGTGAACACAATCATCCACACCCATCTCAGATTGAGGCCACTTCCGGTGGTGCCGCAGCCCGTAGTGTTAACATAGCTCCGGCGGTGGTTACGGCAGCGCCGGGATCCTCGTCGGCGCAGGCCGTTTCCCTTGACTTGGTGAAGGCGAAACCGATTATGGAAGCTAAAACGATCGCCAACCCAAGATTTGACTCACCAGAACTGCAACAGTTTCTCGTCGAACAGATGGCCTCTTCATTAACGAAAGATCCCAATTTCACGGCGGCGCTTGCGGCAGCGATTTCGGGAAAGATTTTCCCACATTGA

>CmWRKY56

ATGGAGAATTTTGGGGAATGGGATCAAAACAAGCTCAAAAATGAGTTACTTAAAGGGATGGAATTAGCAAAGCAACTCCAAATTCAACTCAATGTGAGATCAACACCATCATCATCCATGGCTGCTTCTTCTTCTTCTTCTTCTTCTTCTTCTTCTTCTTCTTCTTCTTCTTCGAATGATGGTTGTGAACTATTAGTTCAGAAGATTTTATGTTCATATGAAAAGGCACTGTCTTTGCTTAACTCATATGGAGCTCAAATAAATATATATGAATCTCCTTCCTCTTTCAATGGTGGAAGTCCAAGGAGTGAAGATTCGGACCGTGAATTTAAAGACCCATTTGATATAACCAATGCTAATTCTTTTCGCAAAAGGAACATTCTTCCAACATGGACACAGAAATTCCAAGTTAGTCCTGGGATGGCCATTGAAGGGTCTCTTGATGATGGATTTGCCTGGAGGAAATATGGTCAAAAAGGCATTCTTGGTGCCAAACATCCAAGAGGGTATTACAGATGCACACACAGGAACCTCCAAGGTTGTCAAGCAACCAAACAAGTTCAACGGTCGGACGACGACCCGACCATCTTCGAAATAACGTACAGAGGGAAGCATAGCTGCAGCCAAGTTTCAAACCTCAGTACTCCATGTACAACAACATCAGAGTTTCAACAACAAAATCAGGGTGTAGTAGTAGAATTACCCGACCAAAAAAAGGCCCAAAATCAACAAACATCACCTGATGCTCTCTTGGACTCATGGTCATCCTTAAGAGTCATAACTCAAAACCTCGACACAACTCATGAACCAACATTACAATTCCATCCTTTATGCTACGACCGTGTCGAGTTTGCTTCAACGTCGACTGTGGACGTGAACTTCACCGAGTTTTCTTCATTTTTGTCCCCAACAACATCTGGTTCCGGGTTGAGCTATTTCTCTGCCTCATCAAGTGGGTTGAGTGAAGGGTTTGTTGTTGGGAATCAGAACTTGAATAATTTGCAGCCAAACAATTGTGAGATATTTTCCTCACCAACTTCAGCTTTAAACACTCAAACTACTACTGCCTTAGATTTCTCTTTTGGTGAACTTCAAATGGAGCCAACTTTCTCATTTGACAACACAGACTTCTTCTCCTAA

>CsWRKY1

ATGGAAGTCGATTGGGATCTTCATGCCGTCGTCAGAGGTTACTCCGCCGCGCCCTCTGCTGCTACCATTGTTCCCGCTTCCTCTTCTTCTTCTTCTTCTTCTTCCAATAACCCTGTCGTCCCTTTTTCTTTTGGTAGAGACCTAACCAACAATCAAATGAAAAACCATTTCTTCTCTCTTCAAGATCCATTTCAACCCTCTAATTGTAATTCCACTCAAGAATTGCATGAGCTTTTCAAACCCTTTTTCCCTAAATCTCAACCATCTCCTTCTCCTCCGCCGCCTCCTCCTGCACCGCCTGCTCCGTCGCTTCTTTCTTCTCCTCCGGCTCCTAAAATTTTGACTCATCAGAAACAGAGCACCCATCTCCCTAAACAACTCCATTCAACCTCTGCTTCTGCTCCGAGATCCAAACGCCGGAAGAATCAGCTGAAGAAGGTTTGTCAAGTTCCGGCGGAGTCTCTGTCTTCGGATATTTGGGCTTGGCGAAAATATGGACAAAAACCCATTAAAGGATCTCCATATCCAAGGGGATATTATAGATGTAGCAGTTCGAAGGGTTGCATGGCCCGAAAACAAGTGGAGAGGAACAGATCCGACCCGGGAATGTTCATAGTCACTTACACGGCGGAGCACAACCACCCGGCGCCTACTCACCGCAACTCTCTCGCCGGCTCCACCCGTCAGAAACCCATCACACCGTCCACCACCGCGTCCGGATCTGAAAAACTCGACCCGAAACAACCGGTCTGCTCGTCGGAAGAACAAAGTACGATCACGGAGAGCAAAGAAGAGAAAGAGGAATTATTAATGGCCGAAGATGAAGAAGACGACGATCTGGGAATCTCCGATTTGATCGTGAACGACGATTTCTATGTGGGTTTTGAAGAACTTGACAGCCCAATAACCGACGACTGCTTTTCCGATCCATTCCCGGCCAACTTCGACCTTCCATGGTTGTTTAACGGAAATCCAGACGGCGAAATTGTAAAACTCCCTTGTTAA

>CsWRKY2

ATGTCGGATGAAATGTTTAAAGATTTATTTTATAGTGGGATGGATGAGTATGAGTCTATAGTGAGAGCTTTTGGAATAACATCGGATTATTCGAATATCAATAATGAAATTTCGGGGACGACTGCGATGAATTCTTCTTGTTCGTTGTCATCTTCGGATGCTGGAGGAGGCGAGGAGGATGATTCTGTGAAAGAGAAGGAGAAACAGATCAGTAAAGATGTTGTGGAAGATAACGGAGGGGAGAGTTCCAAGGCAGCTGGATCGGGTAAATCGAAGAAGAAAGGAGAGAAAAAAGAGAGAGAAGCGAGAGTCGCTTTCATGACCAAGAGCGAGGTTGATCATCTTGAAGATGGATATAGATGGAGAAAATATGGACAGAAGGCTGTCAAGAACAGTGCTTATCCCAGAAGCTATTACAGATGCACAACTCAAAAATGTGGAGTGAAAAAACGAGTAGAGAGATCCTACGAAGATCCGTCCATTGTAATTACAACCTATGAAGGTCAACACAACCACCTAATTCCGGCGACTTTAAGGGGGAACCTGTCGGCGGCGAGCGGGACATTTTCGCCGTCCATGCTGACGCCAATGCCAGTGGTCGGTGGCGTCGGGTTTCTGCCTGCAGAACTATTGAGCAATGCAGGCAACAACCAAGCCGTCGGTGGCGGCGCCACGGTTTATTCACACAACAACTTCGACTATACTTACAACGGTCGGCAACCGGAATATGGGCTTCTGCAGGATATTTTTCCAGCTCCATCGTCGTTTTTCAACCGACAGCCGTGA

>CsWRKY3

ATGGCGGTGGAGCTCCTCTCCGGCTTCACCAACGCTCCTCAACTCTCCTCCCGATCCCCTATGGACCAAGACTCCGCCGTTCAAGAAGCCGCCTCCGGTCTCGACACCCTCAAAAAGCTCGTCACATTACTCTCTCATTCTCCCCCTTCTAATCTCGACTCCGATTGCCAAGCCGTCGCCAACGCCGCTGTCTCCCACTTCCGCAAGGCCATATCTCTCCTCGGCCGCTCCTCTAGGACCGGCCATGCCCGATTCCGCCGTGCCCCTTTAGATTCCTCTAAAATCTATAACGCTACCCCCATTCAACAAATCCCTCCCCCCTCCCTTGACCGCCTCGACTCCGCCACCACCATCAATTTCTCCTACTCCACTGCTCCTACTTCCTCCTTCTTAACCTCCCTTCCTGCTTCCGATTCAGAAATTAAGCTCCAGCATCAACCCTCTTCCTCTTCTTTTCAGATTACTGATCTTTCCAGAGTTTCCTCTGTTGTCTCTAAGCCTTCTTCTGGTCTCAAGAGAAAGTGCGGTTCTGAGAATTTGGGCTCTGGGAAATGCGCCGGATCTTCTGGTGGTCGTTGCCACTGCTCTAAGAAAAGAAAATTGAGGTTGAAAAGGGTGGTAAGAGTACCAGCAATAAGTTCAAAGAACGCTGATATTCCTCCAGATGATTATTCGTGGAGGAAGTACGGGCAAAAGCCGATTAAGGGTTCTCCATATCCAAGAGGTTACTATAAGTGTAGCAGTTTACGAGGATGTCCGGCAAGAAAACACGTTGAGAGAGCGTCAGACGATCCATCTATGTTAATAGTAACGTACGAAGGCGATCATAATCACTCTCAATCAGTTGCAGAAGCTTCAAGTCTCATCCTTGAATCTTGGTGA

>CsWRKY4

ATGGACAATAACAATTATCAGCAAGCTGGTGATTTGACTGATGTGATCCGACTGAGTTCCGCCCCTGTCGCCGGTCATTTCTCCTCCGAATTCTCTCTCGACCCGTTTTCCGGTGACAGACAACTCTGGTCGCACCTTCCTGCTGACAATTCTTCCATGAATTTTGGAGATCCGTTGTCGTTTCCAACTCGAGATCCATTTCTTCTTCCCCATTTTTCTCCCTTCAACTTTGCTTCGGATGGCGGCGGTGAGGGTTTGGCGGCGGACGACCAGCCGTCTAATTTACTTTCCCATATGTTGCAGATCTCTCCGAGTTCTGGTGATGGTGTAATTAGTACTCCTCCATGTGAGTCGCTCGCCACAGCAGTGGGGAATTCTCCGAGGTCGACTTCGGTTCGTGGTGGCGGTGGTCTTGTGCTTCCGACCGGTGGCTCGAACTCTCTTTGTTTAATGGAGAATTCCGGGATTCAGATCTCTTCTCCGCGAAATTCCGCCAATAAAAGAAGGAAGAGCCAAGTGAAGAAGGTGGTGTGTATTCCGGCACCGGCACCGGCGAACAGTCGATCAAGCAGCGGCGAAGTGGTTCCTTCCGATCTATGGGCATGGAGGAAGTACGGACAAAAGCCCATCAAAGGATCCCCATATCCAAGGGGATATTATAGGTGCAGTAGTTCCAAGGGCTGCTCCGCTCGAAAGCAGGTCGAGCGAAGTCGTACTAACCCCAACATGCTTGTCATCACCTACACTTCGGAGCACAACCACCCATGGCCCACTCAACGCAATGCCCTTGCAGGTTACCGCTAA

>CsWRKY5

ATGGAGGAGGTGGTGGCAGCCGATTCGCTACGCTATCCATTTCTCGATGCCAGCGACAGCAAGAGTTGTCTAGGGACGTTCATGGAGCTTCTCGAAGTCGATCAACATTTTTCATCGCAATTTGATGTGTTTGAAACATCATCACCCTCTTTATCGTCTTCCTTGATTTCAAATCCTGAGAATTTGGAGATTTGGAACCAATGGCCTACCACCCCGAATTATTCGTCCTCGATCTCATCAACTTCGAGCGAGATCGTTAACGGTGAGCTGACGACGGAGCCGAATCTGGAGGGGGGAGAACAGAAACAAGATCAGCAACCAACTGTAAAAGCTGACAAGCAGTTAAAAACAAAGAAGAGGAGTCCAAAGAAGAAAGGTGCAGAACCACGATTTGCATTTATGACAAAGAGTGAAGTGGATCATTTGGAGGATGGATATAGATGGAGAAAGTATGGTCAAAAAGCTGTGAAAAACAGCCCTCATCCTAGGAGTTACTATCGTTGTACTAGTGTAGCATGCAATGTAAAAAAACGGGTGGAAAGATGTTTGCAAGATCCAAGCATTGTCGTTACAACCTACGAAGGTCAACACACTCATCCTAGCCCCATTATGGCACGGTCAACCTTCTTTCCTCCTCCCATCTCGGCCACTCTCTACAATGATTATTCAATTCAAAATAGTCACAATTCAAATGTTATGAGCCACTCAATTGCTTGGTGCCACCATTAA

>CsWRKY6

ATGGCGGTCGATCTGATGAGTTTCCCGAAGATGGATGATCAAATCGCTATACAAGAGGCAGCATCGCAAGGTTTGAAGAGTATGGAGCATTTGATTCGTCTTCTTTCTCACAAGCAATCTTCAAACCATGTCGATTGTTCTGACCTAACTGATGCAACCGTTTCCAAGTTCAAGAAAGTCATTTCTCTCCTCAATCGAACCGGTCACGCCAGATTCCGGCGAGGTCCGGTTTCTTCGACTTCCTCATCGTCTTCCGGTTCATCCGCTCATCTCTCTCAAAATCAAGCCATGACTCTCACTCCTACTCCTATGACTCTTGATTTCACTAGACCGAATATTCTGAACTCTAACCCTAAGGGAGCCGATTTGGAATTCTCTAAAGAGACCTTCAGTGTCTCTTCGAGCTCATCGTTTATGTCCTCTGCTATCACCGGAGATGGAAGTGTATCTAACGGAAAATTAGGAACGTCGATCTTTTTAGCACCAGCGCCAACTGCTTCCGGCGGTAAGCCTCCGCTCTCGGCGGCACCTTACAAGAAGAGGTGTCACGAACACGATCATTCCGAAGATTTATCCGGGAAATTCTCCGGATCAACATCAATCTCCGGAAAGTGCCATTGCTCGAAGAGAAGAAAAAATCGGATGAAGAAGACCATCCGAGTCCCGGCTATCAGTTCAAAAATCGCCGATATTCCACCGGACGAGTACTCATGGAGGAAGTACGGTCAGAAGCCGATCAAAGGATCTCCATACCCACGGGGGTATTATAAGTGTAGCACGATGAGGGGATGTCCGGCGAGGAAACACGTGGAGAGAGATCCGAACGATCCAGCGATGTTGATTGTAACGTACGAAGGGGAGCATCGCCATACACAGAGCTCATTACCGGAAAATATGGCGGCGGCTGGAGGAGTAGCTTTAGTTTTTGAGTCAAGTTGA

>CsWRKY7

ATGGAAAATTTCCCTAGTTTTCTCTCAACTTCATCCTCTTCATCCGCCCTTTCTCTCCAACAAATCTTGTTTTCTCGAGCTTCCAACCACGGGGGAGACCATTTTCGACAGCCACCCTTCGTCTCTGATGGCTTTCCGATCTTGTTTCGTGACGTTTCAACAGACGACATGTCGTTTGATGCAACGAGTAGTGTGAAGGATGATGATCGTAATAATGTTGCTGTTTCGGTTAGTACTGAAAGGTTTCGTGTTGATGGGTCGTCGGGTAGAAGTGGAGTAGTGGAATATGGGTTGAAGAAGGAGGAGGGGGAGGGGAGGGGACGAGGGGAGGATTGTGATAAGAAGAAGAAGATGAGAAATAGAAGATTTGCTTTTCAAACAAGAAGTCAAGTTGATATTCTTGATGATGGTTATAGATGGAGGAAGTATGGACAGAAGGCTGTGAAGAACAACAAATTCCCAAGAAGCTACTATAGATGTACACACCAAGGATGCAAAGTGAAGAAGCAAGTTCAAAGGCTAACAAGAGATGAAGGAGTGGTGGTGACAACTTATGAAGGCATTCATTCTCATCCCATTGAGAAATCCACTGATAATTTTGAGCATATTTTGTCTCAAATGCAAATTTACACTACTTCTTATTGA

>CsWRKY8

ATGGAGGTTTCTTCTCATCATTCTTTCAAACCAAACCCAAATCATGAACATCATCTTCTTTCAACTCAACCCACACCAGAAGAAGAACAACCCCTTTCCAAAAAAAGAAAGGTTGTTCAAAAGACTGTGGTTACTGTAAAGATTGGGTCTAAAAAAGCTGCAATAGGGATAGGGAAGATGAAAAATGAAGGTCCACCTCCTGATTTTTGGTCTTGGAGAAAATATGGCCAAAAACCCATCAAAGGATCTCCATATCCAAGGGGTTATTATAGATGTAGTACAACAAAGGGTTGTTCAGCAAAAAAACAAGTTGAGAGATGCAAAACAGATGGTTCAATGTTCATTATAACATACACTTCAAGCCATAACCATCCAGGTCCCAACATCTCTACTCTTAATTTGGATCAAAATTATCAACAAGAGATCGATCCACCACAGCCGTTGGATCGAGATGATGATGAAGATCATCATGATCTTGTTCCAAATCAAGCACAAGATCATGATAACAACAGTAATAATGATGATAAAAACAGCATTATTATTAGTCAGAGTACTGAAGAAGAAGAGGTAGAAGAAGTAGAAGAAGAAGAAGAAGATGAATTATTATTAGTAGAAGATGAAGAGAAAAAAGGAATTGAAAAAATAAAAGATGAGTGTTTGGATCAAGAACCAATAATTATTTCTTCTTCTTCTAATTCTTCTTCATGTTGTGATGAGCTGATGATCATAAAAACAAAAAAATCAGAAATAGAAAATCATGATCATTTCTTTGATGAGCTTGAAGAATTGCCCATTCCTCCACCTTTTTCAAGCACCTTGATGAGATCAAGCTATTCTTTTGATGAAATTAGGATTTCTGCTGCCCCTTCTTGA

>CsWRKY9

ATGGACCGCCGTGTTCGTACAAACCCATTCCTCTCCGAACAGGAAGATCCCGAAGCCACCTCCGACGACGGCTTACCCGAATCACCCTCCGACTGCAATGACTCAAAACCCACCGCTGCTCCACCTCCTAAGAAGAGTAGAAGGGGAGTGCAGAAGAGAGTGGTGTCCGTACCGATCACTGACGTGGAAGGATCTAAAAGCAAAGGGGAGGCATATCCACCGTCCGATTCATGGGCATGGAGGAAATACGGCCAAAAGCCAATCAAAGGTTCTCCTTATCCCAGGGGATATTATCGATGTAGTAGTTCCAAGGGATGCCCAGCAAGAAAGCAAGTAGAGAGAAGCCGTGTGGATCCCACCAAGCTCGTTATTACCTACGCCTTCGACCACAACCACCAACTCCCGGTCACCAAATCTCACCACCACCACCATCATAATTCCTCCCCGTCCTCTGCCGTGATCGCCGCCGTATCTGCCGCAACCGACTTTCCTTCTCCAGGGAGCACTACGACGTCGTCCTCCACTTCATCCGGCGACAACACCAACGCCGCCCCGTCGTCTCCGGCGGCGAAGTTTGAGGAGGCGGCTGCGGTTTTCGCGAGTCAGCCGGAACTGGAACTCGGAGGCGACTCGCTGATGATAAAACCATGCATCGGAGATTTCGGGTGGTTGGGTGAGGTGGCGTATGACAGAATCCTTGAGGGCCCGATTTGCGGAGGTGGCGACATATTCGACGACGCCGATGTAATGGTTTTGTCCACTAGAGGAGATGACGAGGAGGAGTCGTTATTTGCGGATCTTGGGGAATTACCCGAAGGATCGGTGGTGTTCGGCCGACGGCGAACGGTCCAACCGAACGGACCGAACCGGACATGTGGCACAGTTTTAAACTGCTAG

>CsWRKY10

ATGGACGCTGCTGCTGCTTTTGGCCGTCCAAGGCCTGTCGTCAAGACCGAGAAACCTCCCCTTCGCGACCTTAGTGACAACGATCGGAGTTCTCCCTCTAAACAACAACAACAACTTCTCCTCAGAGGGAACAACCATGCAAAACAAGAACATGATACTACTGAAGATAAAACAAGTTGTTCATCTGATCAAAAGGATTTGAGCTGCATCAAACTGCAGGAAGATCAATTGGAATCTGCTCGAGCAGAAATGGGGGAAGTAAGAGAAGAAAACCAAAGACTAAAGCAGAGCTTAACCCAAATCATGAAGGATTATGAAGCTTTGAAAATGCAATTCCTGGGGATTGTTGGACGAGACTGTAAGAAAGTACAAGACGAAGACAACGACGTGAATAAGGAACAACAACAACAACAACACGATGACGATCAAATCGAGCTGGTTTCACTTTCTCTGGGGAGATTTCCGGTGTCGGAGAAGAAAAAAATAGTCGATGAGAAAAGCTGTATGAATATCATCGGTGGTGACCACAACGAAGAAGCCGCCTGTAAAGAGGCTTTATCTCTCGGTTTGAACTGCAAATTTGAACGGGAAGAATCAATGATGGCCGTCGCTAAAGAAGTCGATTCTCCAAATAGTTTTGATCATGAGTCGACGAAGGAAGAAGCTGGAGAGACAAATTGGCCATCTAAAGGTGGGAAGACAATGAGAAGTGTTGAAGATGATGTTACACCGCAGAATCCACCCAAACGTGCTAGAGTTTGCGTTAGAGCCCGATGTGAAACCGCCACGATGAACGATGGTTGTCAATGGAGAAAATATGGGCAAAAGATAGCAAAAGGAAATCCATGTCCACGAGCATATTATCGTTGCACAGGTTCGCCAACATGTCCTGTAAGAAAACAAGTCCAAAGATGCGCTGACGACATGTCGATTTTGATTACCACCTATGAAGGTAATCATAACCATCCATTACCTGCCTCCGCCAACGCCATGGCCTCCACCACCTCTGCTGCTGCCTCCATGCTCCTCTCTGGCTCCACCACCTCCGCAACGGCGGCCTCCTCCTCGTCCACCGCCTCGAATAGTCTCCATGGCCTAAACTTTTACCCAAATAATTCTAAACCCAATTTTTACTTACCCAACAGTAACTCCTCTATAATTTCATCCACTTCCCCAACTCACCCCACAATCACTTTAGACCTCACTTCAAATCCTTCTTCCTCTCCTTCAAGTTCCTCAACCCATTTTGGTAAATTCACATCTAATTTTCCCAATTCTCGTTACCCTTTTACTGGACAACTCGATTTTGGATCTTCTAGAAACAACGTGTTATCATGGAATAACGGTCTTCTTAGTTACAACAGAAATAACCACCCTACTACTACTACTACAACGGCCAACAATATCTACCAAAACTACATCCAACAACAACAACGAAACCCCACCACATCACTACAACATCAACAACCACCTTTACCTGACACCATTGCTGCCGCTACCAAGGCCATCACGGCCGATCCGAGCTTTCAATCTGCTCTCGCTGCCGCTCTTACATCGATCATTGGCACCGGGGGTGCTAGTGCAAGTGCTGGACTCACCAAGTCATTGTCAGGAAGAGGCGAACAATCATTGTTTCAATTGATGACGACGGCAGCTACTACAAATAAAGGAAATGGATGTGGGACAAGCTTTTTGAACAACATTACGACAACGACGACCACCACAAGTAATTCACCACCAACGGGGAATATGGTGTTTGTTCCAACAAATTCGTTGCCGTTTTCGAATTCGAAAAGTGCTTCGGCTTCTCCAGGTGATCATATTGATCTTACCAATTAA

>CsWRKY11

ATGGCCTCCTCTTCCGGGAGCTTAGACACCTCTGCTAATTCTCACCCCTCCTTCACTTTCTCTACTCATCCTTTTATGACTTCTTCTTACTCTGACCTTCTTGCCTCTGCCAACATCGATCCTCCTTCCTCCGCTCCTCTCCGCGCTTCCACTACCGGAGTTCCTAAATTCAAATCCCTCCCTCCCCCTTCTCTCCCTCTCTCTCCGCCCCCCATGTCTCCTTCTTCCTTTTTCGCCATTCCTCCTGGTTTGAGTCCCGCCGAGCTTCTTGATTCCCCTGTTCTTCTTAGTGCTTCTCATGTTCTGCCGTCGCCGACCACCGGGACTTTCCCGTCTCACTCGTTGAATTGGAAGAGCAATTTTGGATATAATCAGCAGAACATTAAGGAAGAAAACAAATATTCGTCTAATTTCTCATTTCAAACTCAATCCTCAAAGCTCCCGCCGACGTCTTTTCAGCCTTCATCCACCATAGCTCCCACCACTCAGGGATGGAGTTTTCAAGAACAGCGCAAGAAGGAGGATAGTTTTTCGTCCGAGAAGAATATGGTAAAGCCGGAGTTTGGATCGATGCGGAGCTTCTCACCGGAATATGGGGTTGTTCAAAACCAGAGCCAAAACAACGGCAGCGGGGAATTGCGGTCTGACTATGGCAACAATTATCCTCAACAATCTCAGACAGTGAATCGAAGGTCGGACGACGGCTACAACTGGAGAAAATACGGCCAAAAACAGGTTAAAGGAAGTGAAAATCCGAGAAGCTATTACAAGTGCACTTTCCCCAATTGCCCAACAAAGAAAAAGGTCGAAAGATCCTTAGATGGACAGATCACGGAGATCGTTTACAAAGGCAGCCATAACCATCCCAAGCCCCAATCCACGAGGAGGTCGTCGCTGTCATCGGCCGGTTCTTCTCAAGCCATAGTGGCTTTGAATCAGGCCGCTAACGAGATGGCGGACCAGTCATTTACAACCCAAGGCAGTGGCCAATTTGACGGCGTTGCAACACCGGAGAATTCCTCGATTTCAATCGGCGACGAAGACTTCGATCGGAGCTCTCAGAAGAGCAAATCCGGAGGGGACGATTTTGATGAGGAAGAACCAGAGGCTAAGAGATGGCGAAGGGAAGGTGACAACAATGAAGGTATTTCGGCTGCCGGCAGCCGGACGGTGAGAGAGCCGAGAGTCGTCGTCCAAACTACCAGCGACATCGACATTCTCGACGATGGTTACCGGTGGAGGAAGTACGGCCAGAAAGTTGTGAAGGGAAACCCAAATCCAAGGAGTTACTACAAATGCACAAATCCAGGATGTCCAGTAAGAAAGCATGTGGAGAGAGCTTCACATGATCTAAGGGCAGTGATAACAACCTATGAAGGGAAGCACAACCACGATGTTCCACCGGCACGTGGCAGTGGAAGCCATTCCCTCAGCCGTCCATTCCCTAACAACGAGCCTCCTGCCGCGATTCGCCCATTATCGGTAGTGACCCATCACTCAAACAACGGAGGGCATCCGCAGGGTCTACGGCTGCAGCGATCTTCGGATTCCCAAGCAGCGTTCACAGTGGAAATGGTGCAAAATGGGAATGGATTTTCATTCCCAGAATTTGGAAACTCAATGGGAATGGGATCCTACATGAACCAAACACAGCCCAATGACAATTTGTTCACAAGAGCCAAAGAAGAGCCAAGAGATCATGATATGTTCATCCAATCTCTCCTATGTTAA

>CsWRKY12

ATGGCTGCTTTCTCCTCTTGGTTGTTGGATTCTGTTGATACTAAATTAGATCTCAATTCTGTGCCTCTCAGATTCTCTGGTGAACCTCCGAAGGAGAGGAATTATATGGATGTCGAGAGGAAGGCTACTGTGAAAGAAGAGACTGGAGCTTTAATGGAGGAATTGAAGAGGGTTAGTGCAGAGAACAAGAAATTGACTGAAATGTTGACTGTTGTTTGTGAAAATTACAACACTTTGAGAGGACATTTGATGGAACAAATGAACAAAAATGGAGAAAAAGAGATTTCTTCTTCAAAGAAGAGGAAATCAGAGAGTAGCAATAACAATAACAATATGGCTGGAATGAATGGGAATTCTGAAAGTAGTTCAACTGATGAAGAATCTTACAAGAAACCAAAAGAAGAAACCATTAGCAAATCAGCTAAAATCACTAGAGTTCAAGTCAAAATTGGAGCTTCTGATTCAAATCTGGTTGTTAAAGATGGATATCAATGGAGGAAATATGGTCAAAAAGTCACTAGAGATAATCCTTGTCCTAGAGCTTATTTCAAATGCTCTTTTGCTCCTAGCTGCCCTGTTAAAAAGAAGGTTCAAAGAAGTGTTGAAGATCAATCAGTTCTTGTAGCCACATATGAAGGTGAACACAATCATCCACACCCATCTCAGATTGAGGCCACTTCCGGTGGCGCCGCCGCCCGTAGTGTCAACATAGCTCCGGCAGTGGTTTCGGCAGCGCCGGGATCCTCGTCGGCGCAGGCAGTTTCTCTGGACTTGGTGAAGGCGAAACCGATCGCGATTATGGAAGCTAAAACTTTCGCGAACCCCAAATTTGACTCACCAGAGTTGCAACAGTTTCTCGTCGAGCAGATGGCCTCTTCATTAACGAAAGATCCCAATTTCACGGCGGCGCTTGCGGCGGCGATTTCGGGAAAGATTTTCCCACATTGA

>CsWRKY13

ATGGATTGCTCCTGGCCTGACACCACCCCCTTTGATCGAAGAAAAGCAGCCGATGAATTGCTTCGTGGCCGTGAACTTGCACAACAACTACGAGCGTATCTACAGATAAGTTCCACTCCGGCCTCCCAAGATCTACTCACCAGAATCCTATCCTCTTTCTCCAAAACACTTTCCATCTTGAATCATCGCTGCGACTCCGATGACATAAATGGCTCTATTGTGGACTCACCCGAGGATCATGGTAGTAGAAAATCCGAGGAATCTGGAGACAGTTGCAAGAGCTCCACCCCTAATAATGATCGCAGGGGTTGCTATAAGAGAAGGAAGAGTTGCCAGAGTTGGGCAAGAGAAAGCTGCGACCTAGTGGACGATGGGCACGCGTGGAGGAAATACGGGCAGAAGACGATTCTAAATGCCAAATACCCGAGAAACTACTACAGATGCACCCATAAATACGACCAGACCTGCCAAGCCACAAAACAAGTCCAGCGACTGCAAGACAATCCCCCCAAGTTCCGTACTACTTATTATGGCAATCACACTTGCTCCAATTTCCTCAAGGCTTCCGACATAGTGCTCGGCTCCTCCAATTTCGACGATTCCTGCAGCGGAGTGCTCCTCAGTTTTGACACCACTGCAGCACCCAACTTTTTTCTCCCACACGATCCTACGTTGGTTAAGAAGGAAGAAGTTGTAACCCCCGATGCTGGCAGTGGCAGGGATGACGAGGCAGTCTGTTCCCCATCCGATTACATGAGTACGGCCGACGACCATCTCTCCGAGGTTTTCATGGGTTCCGTCGTCGATTTTGAGGATGATGACTTACCACCATTCCACTTTTGA

>CsWRKY14

ATGAGTAACATAAACCAAACAATAAATACACTCGCTGGGGGATCTTCTGACAACAGAACCAATAATTTCGCCATGGAAGTTCCTAAATTCAAGTCTTTTCAACCTCCTCCATTCCCCATGTCTCCTTCTTCTTACCTTTCCTCTTTCTCTTCTGGTTTAAGCCCCACTGAGATTCTAAATTCCCCTCTTCTCTTCTCCTTTGGTGTTTTTCCATCTCCTACTACCGGTGCTTTAAACTTGAGAAATGATTATGAGGAGGTTGACCAACAAGAAATGAAGGGAGATGTTAAAAATTACTCTGTTTCTGCGTATAACCCACAAACAGGATCCTCTGTTTCGTCTTATTTTCAGTCCTCTTCTTCCAACTTGACTCTACTGAATCCAAGCGGTTTGTCGTGTGATGAAAGTGGAGCTAAATCAGAATTTGTTAACACAGAAATGGCGGCAGCTGAATCAAAGCAAAATTCTCAGCTAGCAATTTACAACAGAGAACAACAAAAATCAGAGAACGATGGATATAATTGGAGAAAATATGGGCAAAAACAAGTGAAAGGAAGCGAAAATCCACGAAGCTATTACAAGTGCACGTTTCCAAGTTGCCCAACAAAGAAAAAAGTAGAAAGATCATTAGATGGGCAAATTACTGAGATTGTTTATAAGGGAACCCATAATCACGCCAAGCCTCAGCCGACAAGACGCTCCAGTAACTCCGGCGTTTACGATCCATCAGCGGCGGAAACCGGGGTGTTGCAGGAGGATTGTTCGGTGTCGGTGGGGGAGGAGGAATTCGAACCCAATTCGCCGTTTAGCAACTCGATTGAGGACAATGAAAACGAACCGGAAGCTAAGAGATGGAAAGGAGAAAATGAGAATGAGGGATATTGTGGGGGAGGAAGCAGAACAGTGAAAGAGCCAAGAATTGTTGTTCAAACAACAAGTGAAATTGACATACTGCCTGATGGTTACAGATGGAGGAAATACGGACAAAAAGTCGTCAAAGGAAATCCAAATCCCAGGAGCTACTACAAATGCACATCTTTAGGTTGCCCGGTGAGGAAACACATCGAGAGAGCGGCCAACGACATGAGGGCTGTGATCACTACCTACGAAGGCAAACACAACCATGAAGTTCCAGCAGCACGTGGCAGCGGTGGCGGTGGCTACAACACCATCAACAGACCTATACCAACCAACATTCCAATGGCATTGAGGCCATTATCCAGTGTCACCAGCCATTCTTTCCCAGCAAATTTTCCAGCTGCATTCCGGCCAGGAAATTTAGGCATGTCAGAAACAGGAACACAAGCATCATCGTTCCCATTTCAAACATCACATGGAGTGCTTCCAAGTTTCCAAGTTTCGGGATTTGGATCAGCAGCCAAGGAAGAAGTAAGAGACGACACATACTTCATCAACTCATTTCTATCATAG

>CsWRKY15

ATGGACAATAAAGCAACAGAGAGAGTTGTTATTGCCAGACCAGTAGCTTCAAGGCCAACATGTTCCAGTTTCAAGTCGTTCTCTGATATTCTTACGTGTGCATTCGATACTTCTCCACCGAATATGTCATCCGAAACCAGGGTTGCTGCCATTAGACCAAAGACAGTGAGGTTTAAGGGAAAAATATCAGAAACGATCCCTGGAACGAACTCTCATAGTTCATCTGATACTCTCGCTGTATCAGAGATCAAAACCACTGTTTTATTCAAACCTTTGGCGAAGCATGTATCAAAAAGGACTGTCTCTCAGCTGTCACTCATGGCAAGTGGCTTCATAATTAGAACTGTTTATACATGTACTTCTATTCCGTTGTACGGGAACACCAATTTGCAAAACTGTTTACCACCTCCACCAGTTGAGGTCTGTATTCAATGTCCGAATCAAGATGATGGCAATTTCCAATCTGCACTGACCTCAAATCTCTGTATTCAATGTCCAAATCAAGATAATGACAACTTTCAATCTGCGCCGACATCAGATCTTCCTCAAAACATCACATCCACTGTTGAAAACAGTCAATCTATTGGAAGCTCAAGGGTGACTTTAAGTTATAGCAAAAAAGATCCAACATTGCTTCGTCCTCAAATTAGTGGTGCTCAGCCTTCTTATGACGGATATAATTGGCGGAAATATGGGCAAAAGCAAGTAAAAGGAAGTGAGTATCCACGGAGTTATTACAAGTGCACACATCCAAGTTGTCCTGTCAAAAAGAAGGTTGAGAGATCATTGGATGGGAAGGTTGCTGAAATTGTTTACAAAGGTGAGCACAACCACCCAAAGCCTCAACCTCTAAAGCAAAACTCATCCGGGACACAAAGGGAAGGGTCAATATCAAATGGAACTACACAAGATACAAATCCGGAGCTATGGTTCAATTACCTCAATGGGCGGATAGAAGGTTGTGAAAGTAGGATTGAGAATCACATCGAAAAAACTTGTCAAGACAGAGTTACAATACCCTTTGACCCGTTTTCAAATCAAGAAGTCAATGCTAGATGTGGAATCTCCGATAATAATTCATGCGGTCTAAGTGTAGAATGTGAAGAAGGAAGCAAAGGACTCCAGTCCATGGATGATAAATTGCGAAGTAAAAGAAGGGGTGGAAAAAATCCAACAAATGAAGGTGAGACATTAATTGAAGGTGTTAATGAGCACCATGCAATGGCTCAAGATTCCACTGGTATCGAGATTTCTGGAAAAGGCGTTCGCTGGAGAAAATATGGGCAGAAAGTTGTGAAGGGTAATCTATACCCACGAAGTTACTATAGATGTACTGGCCTCAAATGCAAAGCACGCAAATATGTCGAACGAGCATCTGAGGATCCGGATTCCTTCATCACAACTTATGAAGGAAAACATAATCATGGTATTTCACTTGGAACTTCAATTTCTGTAGCTCCTGAAATGGAATAA

>CsWRKY16

ATGGAGGCTCAACAAGCTTTGGCAACCATTGATGATGACAGAGTTCAATCAACTGGAGAAGGAGATGATAAACAAGCTTCTCCTAATCCTAAACAACATATTCATCTTAAGGTCCCTTCTATGGGTGACTTGGAAAAGCCTTCCATGGAAAATCTTTCAGTCCCATCAGCTTCTAATTCAATAACTTGGAAGGAAGAGGATGATGAGCAACGTCATGAAGAAAGAATGAAAAGGACGAAAGTTGAAATGAGGGAAGTAAAGGAAGAAAACGAGAGGTTAAAGAAATATTTGGATGAAATAATGAAAGATTATGAGACATTGAAAAGGAAATTCCATGAGATTAAGAATAATCATGATGATCAAATTAGAGAAGAAGGCAAAAAATCAACACAAACAAGTGGAAGTACAATAAATAATAATAATAATGATAATGATGACGATCATCAAGTTGAAGCTGAAGTTGATGACATGGTTTCACTCAGATTAGGATCAAGATTCTCAACCCATCATCAAAACAAGAACACTTCTTCTTCTTCCTTATCTCTAACTAATAAGATCTTAGATCTCAAACAAGATTATGTTATTCAAACCCCATCAACTATTGATCACAGTATTCATAGTCCAACCCACAGCGAACCTAAAGATCAGGAAGAAGCCGGACAGACGACTTGGCCGCCGAGTAAAATGCCGAAGCCTGGCGGTTTGCCCTCACCTGCTACCGGAGAAGATGAAGTTTCTCAACAGAATCCTCCTAAGAAAGCTAGGGTTTGTGTTAGAGCTCGATGTGATACTCCAACGATGAACGATGGATGTCAATGGAGAAAATATGGACAAAAGATAGCAAAAGGAAATCCATGCCCTAGAGCTTATTATCGATGCACGGGTGCACCCACTTGTCCCGTTAGAAAACAAGTACAAAGGAGTGTTGATGATATTTCCATACTAATAACAACCTATGAAGGAACTCACAACCATCCTCTACCTGTTTCAGCCATGGCCATGGCTTCCACCACCTCCGCTGCCGCTTCCATGCTACTCTCGGGCCCTTCCTCCTCCACCTCCTCCCAACCTGGCCTAAACCATTCATTTACCGCTCCTGCGACTGCTGCGAATCTTCATGGAATGAACATGTATCTCTCTAACAACACCAATTCAAAGCAATTCTATTTGCCAAACTCTTCAATGCTATCTTCCTCTCTAAACCATCCCACAATCACTCTAGATCTCACTTCAAATCCTCCTTCAACTTCCTCCTCTTCTCCTTTCCATAAAATCCCTTTAATTAATAACAATAATTATCCCCCAAAATATCCCTTCACAAATCTTGACTTTGCCTCTTCACAGCCCAATTTCATGTCTTGGAATAATAACAATAATGCTTATAGTAATATTACCAAAAACAATGCAATAATAGGAATGGGATCGGATTTTGCTAAACAACTTCCTCTTCATACTAACATCTACCAAGCTTGTCTTCAACAACTTTCTAAACCTTCAACGACTCCCCAGCCACCAGCTCTACCAGATACGATAGCAGCGGCAACTAAGGCAATTACATCAGACCCGAGTTTCCAATCGGCATTGGCGGCTGCGTTGAGTTCGATTATCGGAGGCGGGGAGACGGGTCCGAGTGTTTCTAGCTTAGTTGTTGGAGGAGGAGGAGGAGGAGGACAAGGAAGTATGGGGTTTGAGGCAGCTAAGTCGTTAACATGCTCTACTTCTAAGAGTACTCCTTCTTCATCTCCTGGGGATAGTAGAGATAATGGGAAGTGA

>CsWRKY17

ATGGACGAGGAGGAGGATGATGAGTTGGAAGAAGAAAGAAGTTTGAAAAAAGTGAAAAGTGAAGAGTCTGGAGGGGAATTGAAGAAGAAGAAGAAGATAAGAAAAAGGAGATTTGCATTTGAAACGAGGAGCCAAGTTGATGTTCTTGATGATGGCTATCGTTGGAGAAAATATGGTCAAAAGGCTGTTAAGAACAACAAGTTTCCCAGGAGCTATTACAAGTGTTCGAATGAAGGCTGCAAAGTGAAGAAGCAAATTCAAAGGCTAACAAATGATGAAGGAGTTGTATTGACAACGTATGAAGGAGTTCATTCCCATCCTATTGAAAAACCTCATGATTCCTTTCAAAATATCTTGACCCATATGCATATTTACCCTTCTTCTTCTTCTTCCTTTTAA

>CsWRKY18

ATGCTGAAAGTTGTGAGCATCAAGAACTTGGTATCTCAAGTGGATGTTTGTTCAAGAGATCATCGTCATGATCAGAATGACGAGGGTTCAAGATCAGAGAGAGCCAATCTCAAAGTTAGTCCACCCGCTCTTGCTCTTGCTCTCGAAACTTCGTCCACCACCCAAGCATATGCTAGAACTACCTTCAAAGACCAAGCTTTGATGGTGAAGGATGGATATAAATGGAGGAAATATGGGCAAAAGATTACCAAGGATAATCAATCTCCTCGTGCTTATTTCAAGTGTTCTTCCCCAGGATGCCCTGTCAAAAAAAAGGTACAAAGAAGCCTGGAAAATAAGTCAATGGTGATAGTCACATACGATGGCCACCACAACCACAACCACAACCACGAAAATGCCTCTCCTCCACCGCTTTCAAGCTCTCAACGAGGGTCGTCTTCGTCACCACCGCTTCCGGTGGAGACCAACCGAGTTGCTCTACCAATGTCTCTCAATCTTGATCTCACTCTTTCCAGACATGCAGATGATCATAAATTATAA

>CsWRKY19

ATGCTTGGGGTAGTGGTTGAGAATTACAGCGTTTTGAAAAATCAAGTTATCGATTTAATCATGAAAACCAGAAAACGAAAAGCAGCTCCAGGATGTGATAATTGTTGTAATTTCAATAGGAGCGCTTCGTCCGATCAATATTGCGGTTGTTGTAGCGATGATAATGATTCCTGTTATAATAAGAGGCCTAGAGAAAATAATAGTAAACCCAAGGTTATGAGAGTCCTCGTTCCCACCCCAGTTTCCGATTCCACTTTGATCGTGAAGGATGGATATCAATGGAGGAAATATGGTCAAAAGGTGACTAAAGACAATCCATCACCAAGAGCGTACTATAAATGCTCATTTGCCCCTACCTGTCCGGTGAAGAGAAAGGTACAAAGAAGTGTTGAAGAGCCATGTTATTTAGTAGCAACATACGAAGGACAACACAATCATCCAAAACCCAATTCAGGAATTGAGTATCAATTAATTGGACCAATTAATTTAGGTTCAAATACAAAGCTTGATTCTTCTAATGTTACATCATCACCTTCTTCCTCTATCAAATCTCCATCATCATCATCGTTAATGCCTTCTATGTCTTTTGATCACTTAACTAAATCTCAACCTCAAATACGATCACCTTCATCGTCTAATTCTTCTTCTTCAACTCAAAAGCTTCTTGTTCAACAAATGGCTACTCTTTTGACCAGAGACCCTAATTTCACTAGAGCTCTTGCCACTGCCATTACCGGAAACATGGTGGATAGTGAAATTTGGGGATGA

>CsWRKY20

ATGGACCACTTTGGCCACCTCACAGATGATGGTGTTCTCGTTGATGCCAATGCTGGAAGCTTTGCCCAATCACGACCTTCCAAGAGACGGAGAAATGGGTGGATCCCTACAAAGGATTCCCATTCCCCCATTCTTTCTTCTCCCTCCCTCACCATTCCTCCTGGAATCAACCCAACTCTGTTGCTTGATTCCCCTGTTATGCTTCTCAATACACAGGATTTGCCATCTCCAACCACTGGTACATTTCCTCCCATACATCAAATAAAGGATGAACAATCATTGTTGAATCCTGTAATGCCAGAAGATGGAATTAGCCACGGTAGTGAGGATTCCTTCTTCAGGTTCGCACCTCAGGGGGAGCTTTGCACTCTTCAAAGTCTTCTGAGGATTGAAAATCAGAATTTATTACGTTTCATAATGGAAGCTGAAATTGATCATCAAGCCCTTGAATCGGAGAAAACGCTGATGGATTTTGAATTCGTACCTGATATTCCAAAAGAGGCTGCTGTATTGAAATATGAAATTGCACCATCCACTGACAATAGTTATTTTGATGGCAAGATTGTTAATGGCAACTGTGAGAATATGGAAAGTTGTCTTTCTTCTATAACTACTAATCAACCATGTATACATGAAGAATCAACACAAGGAGATGACATTGACACGCAGCATCCTTTAGAAGATGAACAAAAAGGGTCTTACATTCCTATGGGAATGTTAAGGACATCAGAAGATGGATACAACTGGAGGAAATATGGACAGAAACAGGTCAAAGGTAGCGAATATCCAAGAAGCTACTATAAATGTACGCATCCAAATTGTCTAGTAAAGAAAAAGGTGGAGCGATCTCTTGATGGTCAAATAACTGAAATCATCTATAAGGGTGCTCATAATCATGCAAAACCTGATCCCAATCGTCGAGCCATGGCTGGATCTGTGCCAATTTCAGGGGATAATCCAGAAATTGGTGAAGGTGGTGGAAACCATTCCAAACTTGAAGCTGGGCTGACGTGGAGAAACTCTCAATATGGGGTGAAGGATATCAAACCTATCTCAAATTGTAGTGTTGATGGTCTAGAGAGGACGCCCTCCGTGTCAGTTTTGAGTGAACTTTCTGATCCATTGTTAAATCCCCAAGAGAAAACTGTTGGGGTTCTTGAACCGGTAGGAACACCTGAGCTTTCATCCACACTTGCCAGTCATGATGATGATAATGGTGGTGGTGGTGACGATGATCTTACAACTCAGGGAAGCATTTCAGTTTGCACAGAAGCTGATGATGCCGAACCTGAATTGAAAAGAAGGCGGAAAGAGGATAGTTCAATTGAGACAAACTTGGCTTCAAGATCTGTACGTGAACCAAGAGTAGTTGTCCAAATTGAAACTGAAGTTGACATACTTGAAGATGGGTATCGGTGGCGGAAATATGGTCAAAAAGTTGTCAAAGGAAATCCAAATCCAAGGAGCTACTACAAATGCACGAGTGCAGGCTGCTTAGTAAGGAAACATGTTGAGAGAGCATCACATGATCTGAAATGTGTCATTACAACATACGAAGGGAAACACAATCATGAAGTGCCAGCAGCAAGAAACAGCAGTCAAGTAAACTCGGGTAATGGCAATGCCCAACCGCCTGCTTCTCATGTGCAACCAAACATGGGTTTGTCCAGAAACTCAAACGTCCCAAAGTCCGAAACAGAAATTCAAGATCTTGCCACCCATTTTTATCCAAAACCGGAATTTAACAATGACTATCAAAGGTCTGGTTTTGACACTTTTACAAACGATATGAAACTTGGAGCTCCTCCATTCTGTCAAATGAAGTTCCCTCCACTCCGGAACACCTTGCCATACAGTACCTTTGGATTAAGTTCAAAGCATACTGCAACAGGTATCTCTGGGTCTTTGGCATCTGTGGTCTCGGACTTTCCGATTTCTTTGCCATTGAACCAAAAACTCTCGGCCGCTGGTTACGATTATACCAATGGAAGACCAATACTACCATTTCAGGTTTTCTTGGCTGGTCAGCAGCTGAGGGAGACAGATAGATTCCTCACACCCAAACAGGAGCACGACGACGATAACATCTGTGCTTCATTCCAACCAGTTGTCGATAGTTCGAGTGGATCTTCATCGTCATCGATCTCATCAGTTTATCAACAAATCATGGGAAATTTTACATAG

>CsWRKY21

ATGGAGTTTCTCTCTCTAATTCAAGAGCTTAATCAAGGCAAACAACTCGCTAACCAACTCCGTACCCATCTCCATCCTTCCTCTTCCTCCCATGGCATTCTCTTAATCGATAAGATTCTTCGTTCTTACGAAAACGCACTTTTGGCTCTCTCCGGCGGTGCTGCTGCTTCCGTCAATTCCGCCATTGCTCCGGTCAATGCGGCTGTCCAAGATGGAGATGTGGTAATGGCGAAATGGAGTGAACAGGTTAAGGTTTCCTCTGCCTCTGCTGTTGAAGGTCCTGGTTGTGATGGATTTAGTTGGAGAAAGTATGGTCAGAAGGATATTCTTGGATCCAAATTTCCCAGAAGCTATTTTAGATGCTCACATCGCTTTACACAAGGATGTTTAGCAACAAAACAAGTTCAAAAGTCAGACAATGATCCAACAATTTATGAAGTAACTTACAAAGGAAGACACACTTGCAACAAAGCCCTTCATTCAACAAACACACCACAAGAACACCAAAACACATTCCTTCAACACCCAATCCCACCAAAACAAGAAGATAAGCCATTGCAGCAGTTACATGATCCTTTATGCTTCATGTTCAGCTCCGACCCCATTCGAGTAAAATCCGAGGACTTAGAATATGCTAATGGTGGCCTCTTTCAACCACTTCGTACCCCGTCCCCGATGTTTGGTTCTGAAGTTCAAGATGACTTAAGCCCTTTTAGGGAAAGTGAGTGTTCTCCGACGTTTGAATCGAATGATATGTTTGGATTGTGGTGTGATTTCGAAACTGAGTTTGTGTCGATTCCTAGTTCAATGACTAATATTTCGATTGGAGATTTGGAGGAGTGTTTTAGTTTTGATAACTTGGAGATGTTCTGTTGA

>CsWRKY22

ATGGATCGAATCAAAGAAGAGAATAAGGCGTTGAGAAAAGCTGTGGAACAGACAATGAAAGATTATTATGATCTTGAAATGAAAATTGGTTTCTTTCAACAAAACAATAACCTCAACAACAAGCTGGAGTGTGATCATAACTTCCTATCATTCCATGGAAATGAGAACAAAAGGCACGAAGAACTAACAAAACACGACCTCGAACTCGGAGAAATGGCAAAGAAGAAGAGACGAGTTGGGTCGGCATCGAAGGAAGACGAAATGAGGGAGAGTGAACTGGGGTTATCATTAGGGCTCCATACAAAAAACAGTAATGATGATTTGGAACAAGAAGATAATGATAGGGAATTATTAATAGAAGAAGAAAGAAGAGAAATTAAGAACAAGGAAAATTCAATAATAATGTCAAATTTCAATTCAATCCAAAACAAACCACAAAGGCCTGAATTGCAAGCAATGGCACCCCCACAAAACAGAAAAGCTAGGGTTTCTGTAAGAGCAAGATGTGAATCTGCTACTATGAATGATGGTTGCCAATGGAGAAAATATGGTCAAAAAATTGCAAAGGGAAATCCATGCCCTCGAGCATATTATCGTTGCACTGTTGCACCGGGTTGCCCAGTTAGAAAACAGGTACAAAGATGCTTAGAAGACATGTCAATTCTAATAACAACATACGAAGGAACACATAATCATCCTCTCCCTGTTGGAGCAACAGCCATGGCTTCCACAGCTTCAGCAGCTTCTGCTTCCTTTATGCTCTTAGACTCATCTAATACTAATAATACCAATCTTTCTAATTCTCTTCATCTAAACCCTAATATTCTTAACTCTTCTTCTCCTTCTTTCCTCCAAACTCAAAACCCTACTAACCATCTTTTCACCCCATTATTCCCCACCTCCTCAACCTCCCACTTCCCCCATTCCTTTTACCATTCCAACTTTCAACCTAATCATCTTGTTGGTCCTCTCGACCGTCGCACGTGGAAACCGACCGATGATAATAAGCCACCACCGTTCACGCCCGATGCCGTGTCTGCCATTGCTTCTGACCCTAAGTTTCGAGTTGCCGTTGCGGCCGCTATTTCTTCGCTCATTAACAAAGAGAATGAACACATGACGACATCGATGACGGGAGAGACGGTCACAGATGGTAAAGGCGGTGGTGGCAGCGATAGTGATAGTGGAAACAAGAAATGGGTTGTTGAATCCCTCTCCTCAAAATCAAATGGTAATTGA

>CsWRKY23

ATGGCTGTCGAGCTCCTCGTCGGATTTGGTGATGCTACACCTTCTAATCATTTCACCCCCAACATGGAAGAAAATGCTGCTGTTTCTGCTGTTCAAGAAGCCGCTTCTGCTGGGATTCAAAGCGTCCAGAATTTCCTCCGATTGATGTCTCATACAACTAATCACCAACACTCCCAACATGATTCTTCTACTTCCTCTACTCCCAATAATGGATATCAAGCCGTCGCAGATTCCGTCGTTAATAAATTCAAGAAGGTTATTTCTTTGCTTGACCGCAACAGAACCGGCCATGCTCGTTTCAGAAGGGCTCCGGTTCTTACAACTACTACTACTACTACTACTCCGCCTCCTCCTCCTCCGCCAAAGGTCAAGCCCCAGCATCAAGATCCGAGTTCGTCGTCTCCGATTTCGGTACCTCCGGTTCAAGTAAAGAAACAAGAATCAGTTTCTGCTTTTAAGGTTTATTGTCCAACGCCTTCCTCCGTTGTGCGTTTGCCTCCTCTGCCTCACAACAACCCTCATCAGCCTTCTCATCCATCCAATACCTTTCAAGCCCAGCAAAATACTTCGTCGGTGGTACTCAAAAATGGGTCTGTAGATAGAAAAGATGCGACTACCACCATCAATTTTGCAGCCTCGCCCCCAATCTCCGCTGCAAATTCGTATATTTCATCGTTAACTGGAGACACCGAAAGTTTACAGCCATCTCTGTCATCTGGGTTCCAGTTCACCCACATGTCCCAGGTCTCTTCTGCCGGAAAGCCCCCTCTTTCATCCTCTTCGTTAAAGAGAAAATGTAATTCCATGGAGGATTCCGCCATGAAGTGTGGCTCATCATCCGGTCGGTGTCACTGTTCCAAGAAGAGGAAAAACAGGATAAAAAGAGTCATCAGAGTTCCGGCCGTTAGCTCAAAACTTGCGGATATCCCACCAGATGATTACTCCTGGAGAAAGTATGGGCAAAAACCCATCAAAGGATCTCCTCATCCAAGGGGATATTACAAATGTAGCAGTCTGAGAGGATGCCCAGCACGGAAACACGTAGAACGTGCCTTAGACGATCCAACAATGCTGATTGTAACTTACGAGAACGATCACAATCACGCCCACTCCACCGAAACACCTGCACCGCTTGTTCTCGAATCATCATAA

>CsWRKY24

ATGGCTAAGAAGGACGATTCTGCAACAAGACCTCCACTTCAACGCCCCACTATTACTCTCCCGCCTCGTCCTTCTATGGAGGCTTTTTTCACCGGCGGTCCTACTGGGGTTAGCCCTGGTCCTATGACTCTTCTTTCAAGTTACTTCGCCGACGGTGCCGTTGACTCTCCTTCCTTCTCCCAGCTTCTCGCTGGAGCTATGGCTTCTCCGATGGCTATGGGGTTTTTCGGAACTGGGTCTACTCCCAATTATTACGCCAAGGATGGACCCGCCTCGGAATTGGAATTTGGGATGAAACAATCGAAGCCGGTGAATTTAGTGGTAGCTCGTTCTCCTCTGTTCTCGGTTCCTCCGGGGCTTAGTCCTTCTGGGTTGCTTAATTCGCCGGGGTTTTATCCTCCTCAGAGTCCATTTGGAATGTCGCATCAGCAGGCATTGGCTCAAGTTACTGCTCAAGCAGCATTGGCAAATTCCCATATGCATATGCAGCAAGCTGAATACCAACATTCTTCAGTACCAGCTCCTACAGAACCGCTAGTGCGTGATCCATCATTTTCTCTCGATGATGCATCTCAACTGGCCATAATACCCTCCACATCAGACACAAAAAGTCTGATTGCAGAATCGACAGAAGTTTCTCATTCTGATAGAAAATATCAACCTCCTCCTCCCCCCCATGGCTCTGATAAACCTGCAGATGATGGCTATAACTGGCGTAAATATGGGCAGAAGTTGGTTAAGGGCAGTGAATTTCCACGAAGCTATTACAAATGCACTCATTTGAATTGTCCCGTAAAAAAGAAGATTGAGCGCTCACCTGATGGTCAAATTACTGAAATTATTTACAAAGGCCAGCACAACCATGAACCTCCTCCAGCCAACAAACGTGCAAGAGATAATATTGAGCCGGCTGGATGTACAAATTCATTGATTAAGCCTGAATGTGGCTTGCAAAACCAAGCTGGAATTTTGAACAAGTCAAGTGAAAATGTGCAATTAGGGTCTAGTGACAGTGAAGGACGAGCTGATACAGAGATAACGGATGACAGAGATGAAGATGAGCCAAATCCAAAGCGGCAGAACATTGACGCAGGGACATCTGGAGTTGCCTTGTCACATAAAACACTCACAGAACCAAAAATCATTGTCCAAACAAGAAGTGAGGTTGACCTGTTAGATGACGGTTATAGGTGGCGCAAGTATGGGCAGAAAGTGGTTAAAGGAAATCCTAACCCTAGGAGCTACTACAAATGCACTAGTGCTGGGTGCAATGTCCGAAAGCATGTTGAGAGGTCTTCAACAGACTCGAAAGCTGTTGTAACTACGTACGAAGGGAAACATAACCATGATGTTCCTGCAGCCAGAAACAGCAGCCACCACACTGTCAACAACACTGTCCACCATATCAAACCACTCAAAGTTGTAGCTCAGAAACATCCATTACTTAAAGAGATGGAATTTGGAACTAATGACCAGAGACCTGCCGTTTTGCAGCTTAAAGAAGAACAAATCACTGTGTAA

>CsWRKY25

ATGGCCGTGGACCTCATCTCCCACCTTTCCCCACTCCCCACTATGGAACCCAACGCCGTCCAAGAAGCCACTTCCGGCCTCGAAAGCGTCCACAAACTCATCCGATTACTCTCCATTCCCAATCCCCATTCCTTACCTTCCTCCACTCAATCCCCAATCGATTTCCCTACCGACTGCCGTGCCGCCGCCGACGCTGCCGTCTCCAAGTTTAAAAAGGTCATTTCCCTCCTCGGCCGTAGTCGTCTCGGCCACGCTCGCTTCCGCAGAGCTCCTCTGCCTCAACAACCTCATTACGTTACTCCCATCCAGCAGATCCCACCCCATCCCCATCTTAATAACAACAACAACATCAACGACGAATCTCTTAATTTCTCCGCTCATAATTCCTTCATTTCTTCGTTGACTGGCGACGCCGATACCAAACATCCTTCTTCCTCTTCCTCGCCTTTTCTCATTTCCAACCTGTCCCAGGTCTCCTCTGCTGGGAAGCCCCCTCTTTCTAGTTCTTCCCTTAAGAGGAAGTGCAGCTCCGAAAACTTGAGATCCGGCAAGTGCGCGGCTGCTTCTTCCTCTTCTCGATGTCACTGTTCCAAGAAGAGAAAATTGAGGGTGAAGAGAGTGGTGAGAGTTCCGGCAATAAGCTTGAAAATGGCGGACATCCCACCGGACGATTATTCATGGAGGAAATATGGTCAAAAGCCCATTAAAGGCTCTCCACATCCCAGGGGTTATTACAAGTGTAGCAGTGTAAGAGGTTGTCCAGCCCGGAAACACGTAGAACGGGCCGTAGACGATCCGGCGATGCTAGTGGTGACTTACGAAGGAGAACACAATCACACCCTCTCTCTCCCCGAAACCTCTACTCTCATCCTCGAGTCCTCATAG

>CsWRKY26

ATGATGCCATCGCCCCAGCGTGATGACACGAGAGTGAAGGAACCTTCTTGGCAGGCAGCAGGAATTGAAACGAAAGGTGTGAAGAGAAAAAAACCTAGAGAAAATGGACGCACTAATAGAGTTGCATTTATAACAAAGTCGGAATTGGAAATCTTGGATGATGGCTTCAAATGGAGAAAGTACGGCAAAAAATCTGTCAAGAATAGCCCTCATCCGAGGAATTACTACAAATGCTCGAGTGGAGAATGTGGAGTGAAAAAGAGAGTAGAAAGAGACAGAGATGATTCAAGCTATGTTATAACAACATATGAAGGTGTTCACAACCACGAGAGCCCTTTCCTGATGTATTGCAATGGTTCAAAACTATTTCATCCTCATCCCATTTGCCCTAATTCCTCTTCTCCTCCCTATTCTTCTACCACTACCCTTTGA

>CsWRKY27

ATGGGGTCATCGGCGTCGGAAGAAGAGATGATGGTGAGACTATTAAAAGGAGGAGATCAAGTGGCGGCCGCCGTGGACGGCGCGATCAAGTGGCGGCCGCCGGATGAGAGGGGGGTGAGCTTTATGAGAGGGAGAAATGCGATTGGAAATTATGGCGGCGAGGATGATCACAACAACGAAAATGACGGGAAGCCTCGGTTGAGGGTGTCAACGATGAAGATGAAAAGGATAAAAGGTAGGAAGAAAGTGAGAGAGCCAAGGTTCAGCTTTAAAACGATGACGGACGTGGATGTTCTTGACGATGGTTACAAATGGAGAAAGTATGGCCAAAAGGTTGTTAAGAACACCCTCCATCCAAGGAGTTACTATCGTTGCACGGAAGAGAATTGTAAGGTAAAAAAGAGAGTAGAAAGGTTAGCGGATGATCCAAGAATGGTGATAACAACATATGAAGGAAGACATGCTCATTCGCCTTCGGATCATAATTTAGAAGATTCTTTCATGGGTCACTTACCTTCTTCCCACCTCACCAATTTCTTCTGCTAG

>CsWRKY28

ATGGATTCTCTAGCAGCCATTCCCTGGTCAGATACTCACACCACTGTGTCCGATGACCACCTTTTCACCCTCACTAACCTTCTCCACGACGACGAGAACCACGCCTCCTCTCCACTCTTCCTCCTCCCCCAAGATGCTGACGACAATAATCGAGCCATCAGGGTCCCCGTCCCCGGTGGTGCAACCTACTTTGGGCCGACAATTGAGGACATTGAGAATGCACTTTCCATTGGTACTCCTAGATCAAAAGACCTCCAATCCCACACTCAAATTTCTCATACCGGATTTTCAATTGTGGAAAGAGCGAACTTGAATAAGGTTGAGCATAAGTATAGCCTTAGAATCAAGAGCTGTGGAGGGAATATGGTGGCTGATGATGGATATAAGTGGAGGAAATATGGTCAGAAGTCCATCAAAAATAGCCCCAATCCTAGGAGCTACTATAGGTGTTCAAATCCAAGATGCAGTGCAAAGAAGCAAGTAGAAAGGTCGATAGAAGATCCAGACATCTTCATCATAACATACGAAGGGCTTCACCTCCACTTTGCCTATCCATTCTTTCTAATGGGCCAAAGCCCACAAGCCCAATCTCCAACAAAAAAGCCGAAGACAATCGACCCAGAGCAGCCCGAGGCCCATGAAAAGCCAAGTTTCCTCGACCCAATAGAATCAAGTGGCTCACAAGGTTTGCTGGAGGACATGGTCCCATGGTTGATTCGAAACCCATCTACCCATCACAACGCTCTATCAAATTCTTCCTCTTGCTTATCCCACCGTTCACCTCCACCCACCCCTCCATCACCCTCCACCTCTCCTACCTTCATAACTTCCTGTTTTTGA

>CsWRKY29

ATGGATTGCATTTTGGATTTTCAAGATTTTGCTTTTACTTTTATAAATGTAGGTGAAATATTTTGTAGGCGGAAGGTGAAAAAGAAGGGGCAAAAGCGAATTCGGCAGCCAAGATTTGCGTTCATGACCAAAAGTGAAGTTGATCATCTTGAAGATGGTTACCGGTGGAGGAAATATGGACAAAAGGCTGTCAAAAACAGTCCTTTCCCTAGGAGTTACTACAGGTGCACAAACAGCAAATGCACAGTAAAAAAAAGGGTAGAACGGTCGTGTGAAGATTCAAGCGTTGTAATAACAACATATGAAGGCCAACATTGCCATCACACCGTTGGATTCCCTCGAGGTGGATTAACCATAGCTCATGAAACTTCTTTTGGGTCTCAATTTTCACCTCAAATCCCACATTTTTTTTACCCTGATCCACCACCACCACCAACAACAACTAATAACCATAACCCTCCCACACCTCCCATTGACCAACCACTACACCATTTCCCCAGCACTCCTAGTTCGACTGAACAACAAGAACCACCAAACTCAAATCTCCAACAACTTCCTTCCAACGAAGGCTTGCTCGGTGCCATTGTCCCCCATGCCATGATGCGTAGAACAACATGA

>CsWRKY30

ATGGACAATAGAGGTGGCGCAGTGATAGAAGCCCACGTGGTAGAATACGTGAGCAAGATGACTAACCAGCACCTCACTACGTCGGAGAGTGATCTTTCCGAGCAGCCCGGGTTCGAGTTTACGGATTGGATGTTTGATGGGTGGCTGAATGAAAACTCTTCGTCTCTGACTGACTCGGTGATGTACCCAGTTTATCAAGAGGGGGAGGTTGATGAGTTTGTTGGGAACACCATTCAGCAAGGAGAGCCTAGCAGCAGAGACTATGGGAGAGAGAGGGAAATTAGAGAAAGATTTGCATTCAAGACAAAATCAGAAGTTGAGATTTTGGATGATGGTTTCAAGTGGAGGAAATATGGGAAGAAGATGGTGAAGAACAGCCCAAATCCAAGGAACTACTACAAATGCTCAGTCGAAGGCTGCCCAGTGAAGAAGAGAGTCGAAAGAGATCGAGAGGATCCAAAATACGTGATAACGACGTATGAGGGTGTTCATACTCATGAAAGCTCTTGA

>CsWRKY31

ATGAACTTCTTCCCCTCCGATGATAAATCCCGAGTCCTCTCTGCTTCTCACTCCAATCTTACTCCCACCAAACTCCCCTTCAATGTTAATACCGGGTTGAATCTCTTGACGACCAACTCCTGTAGCGATCAATCCATGGTGGATGATGGGGTTTCACCAAACCCAGAAGAAAAAAGAGTAAAAAACGAGAGAGCAGTCCTTCAAGCTGAATTGGAGAGGATTAATTCAGAGAATCTAAGATTAAAAGACATGTTAAATCAAGTGACGAGCAATTACCAAACTCTACAGATGCAATTCAATACACTAATACAAACCCAGAAAACAGAAGACGTCGGTGACCCGATTGAGGAAAACCCCGACGGCAGTGGTGGCGGAGGGAATAACAATAACAACAACAACACAAATATTAGCAATAAACTGGTGCCGAGACAATTTATGGATCTTGGATTAGCTACCAATACGGAGAATGATGAGGCATCGATGTCATCGTCAGAAGGAAGAAGTGGGGAGCGCTCCCGGTCGCCGGGAAACACGGGAGAAGTAGCATCGTCGAAACGGCAAAGCCCAGATCAATCTTCCAATTGGGGTTCCAATAATAACAATAATAATAATAAAGTTCCAAAATTCAGTTCTTCTTCGGGTAAAGAAGTGGATCAAACTGAAGCTACTATGAGAAAGGCTAGAGTCTCTGTTCGAGCCAGATCAGAAGCACCCATGATAACAGATGGATGCCAATGGAGAAAATATGGACAAAAAATGGCAAAGGGAAACCCTTGTCCACGAGCTTACTATCGTTGCACGATGGCTCTCGGCTGCCCCGTTAGAAAACAAGTACAAAGATGTGCAGAAGACAAAACAATATTGATAACAACCTACGAAGGAAACCACAACCACCCATTGCCGCCGGCTGCCATGGCCATGGCTTCCACCACATCATCGGCGGCAAGAATGCTTCTATCAGGATCCATGTCAAGTGCTGATGGTTTAATGAACTCCAATTTTTTAGCAAGAACCTTATTACCATGTTCTTCAAGCATGGCTACAATCTCAGCCTCAGCTCCATTTCCCACCGTCACATTAGACCTAACTCAAACTCCTAATCCCTTATTCCAACGCCCCGCTACAGGTCACTTCCCCATCCCGTTCGCAGCCGCCGCTCCCCCTCAAACCTTCCCACAGATCTTTGGACATGCATTATACAATCAATCAAAATTCTCGGGCCTCCAAATGTCCAAGGATATGGAAGCACCACAGCCTCCTCCACCTCCGCAAAATCCATTCACCGACACGTTGAGTGCGGCGGGTGCAGCCATCGCGTCTGACCCCAACTTCATTGCGGCGTTGGCAACAGCAATGACGTCGCTGATCGGGGGATCGCATCATCAGAAGGAGAATGGTAATGGCAATAGTAATGTTGATAATAAAACAAGTAGCAACTCTCAACAGTAA

>CsWRKY32

ATGGTTTCTACAGGGGACGGGGACCAGTTAGAGAATGAAGTTGATTCCGATCAATTGGATCATGAAAACAGTTCAGATAGTCAACCGCAAGCGTCTCAGGACGATCCTGGTGGAACTAATGCATCGAAATCAGATCACAAATGCACAGGGGCTTCCAGTAATACACTTGAAGAAGCTGTCAAGCAGCCTGAGGTTACAATAGCACTAGTGGATCGAGGAGAAATCTCTAGTATAGTGACTGAGAAAGTGACCCATAAGCCAATTACTGCAGAACAGAATCCCCTATCTGTTTTGAAAGTATGTATTACCTCCAGTATACGAGAGAAAGTATCAGAGGATGGATTTAACTGGCGGAAATATGGTCAGAAACTGGTTAAGGGCAATGTCTTTGTTAGAAGCTATTACAGATGTACGCATCCTACTTGCATGGTGAAAAAACAACTGGAGCGTACTCATGATGGGAAAATCACAGACACTGTTTACTTTGGTCAGCACGATCATCCTAAACCTCAACCTCATATTCCAGTTCCTGTTGGAGTCGTTACCATGGTTGAAGAAAAATTAGGTGAACATGCTTCTGGAAATTCTCAAGATAAAACCTCCATTGCACTTAGCCAGACACCTCAACAAACTGAGCTTGCTGATATGCGTCAACCACCATCAGTTATAGCCAGTGATAATGTAAAAGATGAAGTTTCGAAAAGGTCTAGGACTAATGATGAGGTTGATAGTGACGATACACCAGACCTGAAACGAGAGAAGAAAAGATGTAATATTGACGTGACGACAGTAGCAGACAAGTCAACTGTTGAATCTCGGGTTGTTGTTCAAACTCCTAGTGAGGTTGACATCGTCAATGATGGATACCGCTGGCGGAAGTATGGACAGAAATTTGTGAAAGGCAATCCAAATCCTAGGAGTTACTACAGATGCTCAAGTCCTGGATGTCCAGTTAAGAAACATGTAGAGAGGGCGTCTCATGATCCCAAAATAGTGCTTACCACATATGAGGGCCAGCATGACCATGTTGTGCCTCCTATTAGAACAGTGACGTTGAACTCAGTTGGATCCACAACAGCCCAGAGTGACGAAACAAAACCAAAACCGGTTAGTACGGTGGTTCATGCTAGTAAGGATCCGCGAAGTGATTCAAGTTCTGAAGGCAAATTGATTGAAGAGAATGGCAAGTTGAATGCAACAGAAACAAGTGATGACATTATACTCGATGGGGTGGTGGTAAATCCTAGCCCAGGAGTTGCGAGTGAACAAAATAAGCAGCTGAAAGTAGCAATCGAAAGCTAA

>CsWRKY33

ATGGAAGAGGTTGAAGAAGCTAACAAATCAGCCATTGAGAGCTGCCATGGAGTTTTGAATCTTTTACTTCAACCTCCTCCTTCTCCTCATCATCATCAACACCATTTCAAAAATTTAATGCTTGAAACTAAAGAAGCTGTTTTCAAGTTCAAGAAAGTGATTTCTCTTTTGAACTCTGATTTTAGTCATCCAAGATTTAGAAATTTCAACAAAATCCCTCTCCCTTTACCTCAAAATTCCCTATTGGATTCCCCTAATTACACTCTACATCCTCCAAACAAAAATCTTTTCAATTCCCCCCCTGGTTTTAATAGCAAAGTTTCAATTCTTTTGGGAAACCCAGATTTAGAATTGAGTCAAAATGATAAGAATTCCCTTCATATCCCCAAACAATCTCCTTCTTTAAGCTTTAGCTTCCCCCATCATCATCATCCTCAACAACAACAACAACAACAACAACAGCAACAGAGCCTTCTAGCTCATCAGAAACAAATGAAACACCAAGCTGAAATGATGTTTCTTAGGAATAACAATGGGATGAATCTGAATTTTGATACATCTAACTGTACAATGACAATGTCATCAGCTAGATCTTTCATTTCTTCATTGAGTATGGATGGCAGCGTCATTGGCGACAGAAGCTCGTTTCATTTGATTGGACCGTCAACCACGACCACGACAACGTCAGGCAATAGCAAGAGGAAGTTTTCTGCTAGAGGAGAAGAAGGGAGCTTGAAATGTGGAAGCACTAGTAAATGCCATTGCTCAAAGAAGAGGAAACATAGAGTGAAGAGATCAATAAAAGTACCTGCCATAAGTAACAAACTTGCAGATATTCCTTCCGATGATTATTCATGGAGAAAATATGGGCAAAAGCCAATTAAGGGTTCTCCTCATCCAAGGGGTTACTACAAATGCAGCAGCATAAGAGGTTGTCCGGCAAGAAAGCATGTCGAGCGGTGCTTAGAAGACCCGTCGATGCTTATTGTAACATACGAAGGAGAACATAATCACCCGAAAATGTCGACGCAATCTGCACACACTTAG

>CsWRKY34

ATGGATATTTTTTTGGACCTTAATGTGGATCCTAATTCTTCTTATGCTAATTCAACTATGGATGAAGTAGCTCATCATTCTTCAAAAAGAGATCAATTTGATGGAGAAATCTATGGAGATAAAGAAAAACTCGCACTGAGCTTGTCAAATAAGGGAAGCGAATCGAGCCCGACATTAGAACAGGAGTTGGATAGAAAAATCCAAGAGAATGGGAAGCTAAGTCAAATGTTGAGAATAATGTATGAGAAATACATTAATCTTCAGAAACAAGTGATGTATTTGTTAAGCAACCAAAAGCAAAGCACAGAAATGGAAGGTGTTTGTTCAAGGAAGAGAAAGGCAGAAGGAGAGCAAGAAGATTATGAGAATTTAGAAGGAATTTGCAGCACAAGAGATGAAGATTTTAACAGGTGGCTTAAGAGGCCAAGATTAAATGGAAATTCAAAGGTTTCTAAAGTTTTTGTGCAAAAAGATGCATCAGATCCAAGCTTGGTTGTGAAAGATGGGTATCAATGGAGGAAGTATGGGCAAAAGGTTACAAGAGACAACCCTTCTCCAAGAGCTTACTTCAAATGCTCCTCTGCACCAAATTGTCCTGTCAAAAAGAAGGTGCAAAGAAGTTTGGAAGATCCAACAATTTTGGTGGCAACTTACGAAGGAGAACACAGCCATGCTAGCCATTTCCAAACTGAACTTTCTTTAAGGTCAATCAATGGCGGAAAAGGCAGTGCAGTCCCAGTCTTAGCGACGATCAAGCCGTCATGTGCTACGGTGACCCTCGATTTGATCCACGAAGATGGGCTGTTTAAGAGTCCTAAAGATTACGCGTCATCGGAGTCGGCCGAGGCAGCGGTTTGGCAGGAGTTTTTGGTACAACAAATGGCCTCTTCTTTAAAGAAGGATCCTGAATTTGCTGGCATTGTTGCTGGTGCCATTTCAGGCAAGGTTTTGGGAAACCAAACAAATAGAGAATAA

>CsWRKY35

ATGGCTGCCGGAAACGACGATTGGGATCTTTCCGCCGTCGTCCGGAGTTGCAATTCAGCTGGTTCCGCTACTGACCCAACATCCGCCGCGGCGGAAGAATCTGCGTTGTCTTGTCTAGCTTCTTTAACGTTCGACGACGATCCAAACGACGTCGCATTCTCCTTTTCCGATATCTTTCAACCAAAACAGCCAAACGGCTTCCATGAGTTACACCAAGCCTTCATCTCTTTTCTCCCTAACCCCTCCACCACCGCCACCACCGTCACAACCGTACCTGCTGCTGAGCCCGAAATCCCTTACTTAACAACCCCCCCGACGAATCGCCATTTCCGTCAGTTAGGTTTAAATTACAGAAAGAACCAACAGAAGAGAAGGGTATGCCATGTTACTGCAGATAACCTCTCGACGGACATGTGGGCTTGGCGTAAATATGGGCAAAAGCCCATTAAAGGATCTCCGTATCCAAGAAACTATTACCGTTGCAGCAGCTCAAAAGGGTGTGGAGCAAGAAAGCAAGTTGAACGCAGCAACGACGATCCCGAAACTTTCACCATCACATACACCGGCGATCACTCTCATCCCCGTCCAACTCACCGTAATTCCCTCGCCGGAAGTTCCCGCAACAGGTCCTCCTCCTCCTCCTCCTCCTCCCGACACCCCACCATGGGTGATTCCGATCCCCCGATGATGACCGCCTCCGTTCTCCTACCCTCCTCCTCTTCCCCTGCAGCTTCTCCCATCACACCGCTGAACGACTACGATTCCACCATCGGCGAGAAAGATGGAGAGATGTTTGAAGATATGCCGATTGATAGTGACGATGAAGACGATGATGACGATATTCTGATCCCGAACCTGACGGTGAGAGATGAAATCTATGTTGGGTTTGATGGGGTTGGGAGGGGACGGTCATCGTAA

>CsWRKY36

ATGGCCGTCGATCTAGCAGCTTTTCCGGCCTTCTTCGACGATCAAACAGCCATTGAAGAAGCCGCCACTGCGGGTTTACAAAGCATGAATCATCTAATCCATCTCTTATCCAAACAACACCACCACCACCACCATAACATCGATCTCAATTCTTCTCTTCTCACCGATTTCACCGTCTCCAAATTTAAACGGTTGATTTCCCTCTTAAACCGAACCGGCCACGCTCGTTTCCGCCGTGGACCTTCCGATTCTCCAAATCCCGTTCTGAATTCCCTCGATCCGCCTCAGAAAACCCATTTCTCTAAACTTAATTTCTCCCCTGTTTCGAAAGTCCCTGAGTCTAGAGATTCCACTACTACCTCTTCCTTTGTGTCTACGGTCACCGGCGACGGTAGTGTTTCTAACGGTAAGCTTGATCTGTCTGTCTATGCAACGCCGCCGGCCAACGCCGGTAAACCGCCGTTGGCGATGAAGAGCAAGTGTCACGATGTATCTGGATTTGGTTGTAAGGTTCCTAATTCGAAGCTCTGTCACTGTGCCAAGCGAAGGAAATCTGGAATGAAGAAAACGGTAAAAGTCCCGGCGATCAGTTCTAAGATCGCTGATATACCCTCCGATGAATACTCATGGAGAAAGTACGGCCAAAAACCCATCAAAGGCTCACCTTATCCTAGAGGGTATTACAGATGTAGCAGCGTGAAAGGATGTCCGGCGAGAAAAAAGGTAGAAAGAGCTCGTGACGATCCGGCGATGCTTCTCGTAACATACGAGGGGGATCACCGTCACCCGCACCCTACGGTCACCGACGGCGTTTCCCAAAAGAGTTGA

>CsWRKY37

ATGGATGCGCCGGAGTTTTCTCACGGCCGTATTTCGCCGGCAGAGCTAAGGAGGAAGATCACGGCGAAGCTTCTCCCTGGCCAAGACTCAGCAGCTCACCTTCGGATTCTTCTCCAGTCAGCGACCGCGACGGAGCAGGACAAGCGAGCTCTTGCTACCAAGATCTTGACTTCCATCACCGAAGCGATCTCCATTCTGGAGTCCGCTGGCGAGGAGTTGAGCTGTCCTGATCATTCCCTTTGCTCCGATCTAGATTCCGGCGAATCACGGCGGAGCAGGGCGGTTAAGGATAATCCATCACGTGCCAACAAAAGAAGGACGTCGAGGACGACGGAAGATAACTATGGTTGGAGGAAGTACGGCCAAAAAGCTATCCACAACACAACTTATCCAAGGAGCTATTATAGATGCACTCACAAGTTTGATCAAGGTTGTCAAGCCACAAAGCAAGTACAGAGAATGGAGGGTGATGATTCAGAAATAATGTACAATATCACCTACATATCTGACCACACATGTCGCCGCCCTGCCTCTCCTATCGATGCCTCCGCCATTACCACCCTCTCCGATTCTTCCAACCTCATATCTTTCAGCTCCATCGATTGTAACGGTCCATTCACCGAGGGTACTGGCTATAGTTTGATCTCCTGGCGGCCGAGTGATGACGATGTAGTGAAGATAGGAGAGACAGCCACGACCAGTGGTTCCACAGATGATCATGAGATTGATTTATGGTCGGATTTGAAGGATTTTTTGGAGCTTCCAAACAACGGCTATGACAACGACAACGAAGAATCCATACTATTTCTCAACCGAAGATGA

>CsWRKY38

ATGGAAGAAGCCATTATTTCTTTGATTCTTCGTGGGTGTTCTTTGGCAAGGCAATTGGAATTCAACGTTTTAAATTTGGGGAGTAATAATATCAATATTTCTGATCAATCCCATTTCATGGCTCGATCATGTGATGAGATTTTGGGTGTTTTTTCCGCCGCCAAGGACCGGCTGGGCAGCCACGAACCACCGCCTCTCACAGGGGTTCAGGGGGAGGTCGGGTTGGAGGAGTGGTTGAAATCTACTTGCTCTCAAGCTATGGAGTTGTCGCAAATGCAGATGATATCTCCGTCCCCGCCGTCTGCGATTGTGTCGGTTCCGGCGGCTGTGAAGGATTCTGGGAAGCTTATGGCAATGGGTCTTTCTTCTTCTTCCTCCTCCTCGTCGACAAAAGCACGAAGACGAAAAGATGATACGGAGAAGAGAACAGTGAGGGTGGGGGCGCCGCGCATAGGAAACACAGAGCTGCCGCCCGACGACGGCTTCACATGGCGGAAATATGGACAGAAGGAGATTCTTGGCTCTAGGTTTCCCAGAGGCTATTTCAGATGCACCCACCAGAAGCTCTACCACTGCCCAGCCAAGAAGCACGTCCAACGCCTCGATGACGATCCCCACACGTTTGAAGTCACCTACCGCGGCGAGCATACATGCCACATGTCGGCCACCGCCCCCTCCGCTCCCCCGCCGCCGCCTCTCCCCACCGACCAACCCATCTCCCACTTTCACCCAGCCGCCCCCTGGCTTTCGATGGATGTGGTCGGTTCAGGGTCGACATCCGGCGCTCCGTCCACTGTACGGTACGGAAAAGACGTGGGGGATCAGTTTGCGGTGGTGGACATGGCTGACGTGATGTTCAATTCCGGGACGAGCAGTAGTAATAGTATGGACTCTATCTTTGCCGCATACGTGGGGGAGGATCACAAGTGGGAGAAAGAGGACAAGAAGAAGTAG

>CsWRKY39

ATGGAGGATTGGGGTTTGCAAGCAATTGTAAAAGGGTGCAATGGAATACCAATTGGTTCTTCAACAACAGCAGCAACAACAAGGCTTATGGAAGATTCAAATTGGTATTCTTTTTTGAGATCAGATCAAGAAGAAGATGAATTTTTTTCTTCTTGTGTTTATAATAGTAGTTATAATAATCCTCAAATTTCAAGTTCTTCAATATTTCATGATGAGTTTGAAGGATTATTTGGAAGAAATTCATCTAATAATTCAGCAGCTGCCTCCATTTCTCATCTTCTTAGAGATTTTAAGGAACCTGCAGATCAAAAGCTTCATCACAAGAACCAAATTATCCAACCAACTAAACAGAAACAAAGCAAGAAAAGTAGACAAAATAGGGTAGTGAAAGAGGTGAAAGCAGATAAAGTATGTTCAGATTCATGGGGATGGAGAAAATACGGACAAAAACCAATCAAAGGCTCTCCATACCCAAGAAGCTATTATAGATGCAGCTCCTCGAAAGGATGTTCTGCTCGAAAGCAAGTCGAGCGTAGCCTCTCCGATCCCGAAGTCTTTATCGTAACCTACACGGCGGAGCACAACCATGCCGAGCCTACTCGCCGCAACGCCCTTGCTGGGACCACCCGGAAGAAGTTTCCGGCACTTGAGAACCCGAATTTAGACATGATTCTCTCACCTAATAATTCAACTTCAGTGGCATCCATTGAAGAAGATCAACATCCAATGGAAGGTGTGACAGATGGGGAAGTTTTGATTGATATGCCATTTGAATTCTTCACTGGTTTGGAGGATTTACTTTTTGGATGA

>CsWRKY40

ATGGTGATGGGTAAAAGAAGTACAATAGCTTCTCCAAGAATTGCTTTTCAAACAAAAAGTGTTGAGGATGTTCTTGATGATGGCTATAGATGGAGGAAATATGGTCAGAAGGCTGTCAAACATAGCAACCATCCTAGGAGCTATTACAGGTGTACACATCACACATGCAATGTTAAGAAACAAATTCAAAGGCATTCAAAGGATCCAACTATAGTGGTTACAACATATGAAGGGATTCACAATCATCCATCTGAGAAATTAATGGAAACTCTAACCCCTCTTCTCAAGCAATTGCAATTCCTTTCAGGCATTTAG

>CsWRKY41

ATGGCGGAACCCGAAAGCTTTGGAACTCACCAACTCGGAAGCTCCAACGCGGCTACTGAAGGCCAGGAAGATGATGAAGAAGAAATGGACGATTCGGATGATGAATCAGAGGTGGACTTGGGAGGAGAAGGAGGAGGAACAGGGAGTGAGTTCAAACCGACTGAGTTGACAACCGGTTCGTCGCTTAATGAAGCTTTGGTGACGGGTTCACTTTCTGAAACCCTAACGGTGGCTTCGTCCGCTGAAAACGGTCCGTCTGATGGTTTGCAGCCTGACGAAGGAGCTGAATTGAAGCAAGCTCCGTCTTCCCACAGTGAGCCTTTGGCTGTTGAAGCAACCCAGACAGATAAGGTGCAAGAACAAAACCGTCTTCAGTTGACGATATTTAAAGGGCCTGATTCAGACCAATCACCAACTTCAGTTACCCAGTCTATCTCATCCTATGCAAGTTCAAATTTATCTGAACATAAGCTGTCACCTAAGAGGGTCCAGAAAATATGTAAGCCAGAGCCAAGCCAGAAAAATTTCTTCAACCATAAAACTCCATCATCTGTTCCCAATGCAAGGACACCTGCTTCAGATGGTTACAATTGGAGAAAATACGGTCAGAAGCAAGTGAAGAGTCCTAAGGGTTCACGTAGCTATTACAAGTGTACATATTCTGAATGCTTTGCTAAGAAGATTGAATGTTGTGATGACTCAGGGCAAACAACAGAGATTGTTTACAAGAGTCAACATAGCCACGATCCACCTAGGAAGATTAGTACACCAAAGGAAAGTAAGCTTGTGCCTTATGTTGAGCCTGTGGTTAAAAAAATCATTGCTGAGCATTCCAGAAGAGTAATTAATGATTCAGATTCTCCCACACCTTCAAAAGAACCGTTACGAGAAGCAGCTATAGTCGTCTTTGAAAGGAAACGACAGCACTCAAATGATTCTAATGGAAATGATGAATATAAAATCAAAGATGAGAATGACGATGAACCTGGGACAAAACAAATAGTTAAAAAAAGCAGTGCGGGAAACTCAGGCACTCCTTTAAAACCTGGAAAGAAACCCAAATTTGTGGTGCATGCTGCAGGTGACGTTGGAATCTCGGGTGACGGATACAGATGGCGGAAGTATGGTCAGAAAATGGTGAAAGGAAATCCTCATCCTAGGAACTACTACCGATGTACTTCTGCTGGGTGTCCAGTCCGAAAGCACATTGAATCAGCGGTAGAAAATCCAAATGCAGTAATAATAACATACAAGGGAGTACATGACCACGACACGCCTGTACCAAAGAAACGACACGGTCCACCAAGTGCTCTTCTTGTAGCTGCTGCAGCACCAGCCTCCATGAGCAGCAATGCACAACCGAAGAAAACTGATGTGGTGGAGTCCCAAATATCTTCAACACAGTGGTCTGTGGATGCTGAAGGAGAGTTGACTGGTGAGGCCTTGGAGCTTGGGGGTGAGAAGGCAATGGAATCTGCTCGAACACTTTTGAGCATTGGATTTGAAATCAAGCCTTGCTGA

>CsWRKY42

ATGGAAGAGGTTGAAGAAGCTAATAGGGAAGCTGTTGAGAGTTGTCATAAAGTGTTGAATCTCTTGACTCTGCCTTCCTCATCTCAAGATCACTTGAAGCTTAGGAGTTGTTTAATGGCGGAAACTGGAGAGGCTGTGTTTAAGTTTAGGAAAGTTCTGTGTCTTCTTGATTCTTCTGGGTTGGGTCATGCAAGGGTCAGGAAAAAGAAGAAGGTCAACAACTTTTTGTTTAATTCCTCCTCCTCCTCCTCTTCTTCTTCCCCTTTTCCTCTTCCCCAATCTTTGTTCTTAGAAACTTATTCCCCTGATTGCAGAATGGACCACCTCCAGGGCAGGAATTTGCAGATGGGTCCTCTTTGTTTGGGGAACCCATCTTTGGAATTGAACACAAATGCCAAAACTTGTTCAATCCAACAAATACAATCTCAATCTGCTGCTCTTTATCATCATCATCATCATCACTTGCTTCAGAACAGGGTGGTCTTGAATAATAATCCTAATCCTCCGCAGCCTGAAGTAGTGTATCTTAGGAGCAGTAATGGTGTTAACTTGAATTTTGATAGCTCTAGCTGCACACAACACACAATGTCATCCACTAGGTCGTTTATTTCTTCATTGAGCATTGATGGGAGTGTGGCTAACTTGGATGGGAGTGCCTTCCATTTGATTGGGGCGCCCCGGTCTTCCGATCAAAATTCGTATCATAAGAGGAAATGCAATGGGAGAGGGGAAGATGGGAGTGTTAAATGTGGAAGCAATGGGAGATGTCACTGCTCAAAGAAGAGGAAACATAGAGTGAAACGGTCGATCAAGGTGCCCGCGATTAGTAACAAGCTTGCAGATATCCCTCCCGATGATTATTCCTGGAGGAAATATGGCCAAAAGCCAATCAAGGGTTCTCCACACCCAAGAGGATACTACAAGTGCAGCAGCATGAGAGGTTGTCCAGCAAGAAAGCACGTTGAGCGTTGTCTCGAAGAACCTTCAATGCTCATTGTTACATATGAAGGTGAACACAACCATCCTAGAATACCTTCTCAACCTGCAAATACTTGA

>CsWRKY43

ATGGATGATGTTTCCTTCAACCATAATAACCACCACCAATTCTATTCATCTGATCCATTCGAAGATTCCGACGAATTAAAACCCACCCCCGACTCTCCTCCTCCTACTTCCACCATCGCCGCTGCCGCCACCACCAAAAAGGGCAGAAGAGGAATGAAGAAGAAAATTGTATCAGTGAAAATTAACGGCGACAGCCCAAGAAATTCCTCCGGTTCCGCAACTCCACCTTCAGATTCATGGGCTTGGAGAAAGTATGGTCAAAAACCCATCAAAGGCTCCCCTTATCCGAGGGCGTATTATAGGTGTAGTAGTTCAAAGGGATGTCCAGCGAGGAAGCAAGTAGAGAGGAATCGGCTTGATCCAACCACGTTGGTCATTACATATTCGTGTGAGCATAATCATTCCGGTCCGGTTTCTAGAAATAACAACAACAACAATAATCAGAATAACCAAATCGTTGTGATGAAACCCGGTTCACCAGAGACGGTTGCGGTTCATCAAGAACCCGAAGTGGAAGAGAAATTTGTGGAGATTGGGGGGGGAGAAGAGTCGTTGATAACGGCGGATGAGTTTAGTTGGTTTGGGGAAATGGAAACGACGTCGTCTACGGTACTCGAAAGCTCCATTTTTTCCGGCAGAGCGTCCACTGGATTGGTGGATCATTCAATTTCTAGTGACGTGGCAATGCTTTTTCCAATGGGGGATGATGACGTGGATGAGTCACTCTTTGCGGATCTAGGGGAGTTGCCGGAGTGTTCACTCGTGTTCCGGCGCGGCGGAGGTCGTGGATTACCGGTGGATGAACAACCGGCGGCAGCCCAGCGGCGGATCACGCCTTGGTGTGGGACAACCACATGA

>CsWRKY44

ATGGAGTTGCTTGGTCTTAATTACAATTCTGAATTTACTGAGGCTTTTAATAATCCTCCAGCCACCCCCAATTGTTCGTCCTCCGTCTCCTCCGCCTCTAGCGACGCTCTTAACGACGACGAACCACCACCACCACCACCACAACAAAAAGAAGATAAAACTTTTGTTTTCATGATTGTTTTGATCAGATCCATCCTGAAAATGGGATTGAAAGGGATAAAAAAGAGGAAGGAGAAGGAGAAGGAGAAGAAAGCAAGATTCGCATTCATGACAAAGAGTGAAGTTGATCATCTAGAAGATGGTTATCGATGGAGAAAGTACGGCCAAAAAGCTGTCAAAAACAGCCCTTTTCCTAGAAGTTATTACCGTTGCACGAGTGCAGCATGTAATGTAAAGAAGAGAGTTGAACGATCTTTTGCTGATCCAACTGTAGTGGTGACCACCTATGAAGGCCAACACACTCACCCAAGCCCTATCCTTAGCCGGTCCGCCCTAGCCGTCGCCATTCCACCTCCCTCTTTCATCCCTGGGGCGGGCGGAGAGTGTGTTGGGGGCGTTGTAGCCATGCCATGGCTCAAACCTAGCAACAATGATGCTCATGATGGTAACACGGTTCCAGCAATGTCTCATCAATACTTTCAAAACTCGACCTACATCACTGCCCAAAATGTAGCCGCCAACTATAACCGAAACAACCACATCGGAGCAGCGAACGCGGCCGGAATCCTCCAGGAGAAACGGAAAAAAGAGATCGATTTTCCGTATGTTGAAACTTTGGTTACAAGTTCGATGGGTCTTCTAACTTAA

>CsWRKY45

ATGTTTTTTCCTTGCTCCGATGGGGGCGGAGGACTGTCAGCCTATCATCATGCCGACATGTCGTCCGGCGGTGCTTCTGATATGTTCGGTAATTTTCAGGGTGGCGATATGGAGGCGGTAAGTGGGTTTTTGGGGATGAAAAGAGAGGTGGATGGCGGGGCGGTGGAAGCAGAGGGCGGAGGGAAAAAGAAGGGAGAGAAGAAGGTGAGGAAACCGAGATATGCTTTTCAGACAAGAAGCCAAGTTGACATTCTTGACGATGGGTATCGTTGGAGAAAATATGGTCAAAAAGCCGTCAAAAACAACAAGTTCCCGAGGAGCTATTATAGATGCACACATCAAGGATGCAATGTGAAGAAACAAGTGCAGAGGCTAACGAGAGATGAAGGGGTGGTGGTGACAACATATGAAGGAATGCACACACACTCCATTGATAAGCCAACAGATAACTTTGAACAAATATTGAGTAGAATGCAGATTTATAGTACTCCTTTTTGA

>CsWRKY46

ATGGGGAGAACTGATGATAACATTGCTATTATTGGGGATTGGGTACCTCCAAGTCCTAGCCCAAGAACCTTTTTCTCAGCAATGCAAATGCTAGGGGAGGATATTGGTTCTAAACCTTCAATGGACACTACTACCATCAGTGATCATAAAACCGAAGAACTCTTTCTCAGGCCTCGAGAACAAACGGTGTCCGAAAACGCCTTTGCAAGAGGCGGGATCCCGGGTGTCAATTCTGGGGACCGAGGAATGGAGTTCAGTACGTTCTCGGAGCAAAAATTTCGTGGAGGACTCGTGGAAAGGATTGCAGCTAGAGCTGGATTTAATGCTCCGAGATTGAATACAGAGAGCATTAGATCAACAGATCATTCTTTGAATTCAGAAGTAAAGTCTCCTTATTTGACAATACCACCTGGTCTCAGTCCCACTACATTGCTAGATTCTCCAGTTTTTCTTTCAAATTCACTGGCTCAACAATCTCCCACAACTGGGAAGTTTCCATTTTTACCAAATGTATGTATTTCTCGGAGCTCAACGATGATGTCAGAGGCCAACAACAAAGGCAACAACAATCTGTTCGACGATAACAATACATCATTTGCTTTCAGACCCAGTGTGGAATCTGGATCCTCCTTTTTTCTCAATGCAGCAAGCAAAACAGCTTCTGCTACTATTCTTCCACAATCTTGTCCAAGAATTGAGGTTCCAGTTCCACGTTCAGAAAATTCTTTTCAATCTCATCGTGTTGAACCATCTCTGTCTTTGCCCCAAAATCGAATTGGTCACCATCCTCAAGTGGGACTCTCTACAACGTACGTCGAGAAGGACAATGGGGGTAAGGCTGTGTCAGAAGAGCAAAGACCCTTTGATTCTCTTGGTGGTGGTAGTGGTGGTAGTGGTGAACATTCTTCACCTCTCGACGAGCAACTGGATGAAGGGGAGCAAAGAGGCAGTGGGGATTCTATGGCTGGTGGTGCTTGCGGTACTCCCTCGGAGGATGGGTATAATTGGAGAAAATACGGACAAAAACAGGTTAAAGGAAGCGAGTATCCTCGGAGTTATTACAAGTGCACACATCCTAACTGTCAGGTCAAGAAGAAAGTCGAGCGATCTCACGAGGGCCATATAACAGAGATCATCTATAAAGGAACACATAACCACCCAAAGCCTTCACCAAACCGACGAGGAGCAATTGGATCTTCTGATTCCCATATGAATATGCAACTAGACATCCCTGCACAAGCAGGCCAACAAAGTGCTGATGTTCCTCTTTGGGAAGATTCACAAAAAAGGGTTCCAAGTGGAGCTCCTGATTGGATGCATGAGAACCTTGAGGTGACTTCTTCAGCATCTTTGGGTCCTGAATACGGCAATCAGCCTAACTCTTTACAAGCTCAAAATGGGAGTCATATTGAAACAGTTGAGGCCATTGATGCCTCATCCACATTCTCTAATGATGAAGATGAAGATGATAGGGGAACGCATGGCAGAAACTCGATGCCTATGTAA

>CsWRKY47

ATGAGTGGGGCCACTAGAGCTATCCGTGAGCCTAGGGTTGTTGTCCAGACTACCAGTGAAGTCGATATTCTGGATGACGGTTATCGTTGGCGCAAATACGGACAGAAGGTTGTGAAAGGAAATCCAAATCCTAGGAGTTACTACAAGTGCACAAATCCTGGCTGTACAGTGAGGAAGCATGTCGAGCGGGCGTCACATGACCTGAAGTCCGTAATAACCACATATGAAGGAAAACACAATCATGATGTTCCTGCAGCTCGAAACAGTAGCCATATCAGTTCGGGTACATCCAGTCCTGTAACTGGTCAAAACTCGACTGCGGCAATTCAAACTCATGTTCACAGGCCAGGGCCATCCCAGCCTCAAAACACCATTCCAAGATTTGAAAGGCCAGCTTTTGGTTTTGCTGGAAGACAACAAATGGGGCCTGCCCATGGCTTTGCTTTTGGAATGAACCAACCTGGATTGGGAAATCTGACCATGGCAGCAGTTGGGCAACCCAAGCTTCCCGTTTTGCCAATGCATGCTTACTTAGGACAAGCACACCATGTTAATGAAATGGGTTTCTTGTTGCCTAAAGGAGAGCCCAATGTAGAACCTACATCTGATCTTGGCTTTTCCAGTGGTTCAACTGTGTATCAGCAAATTATGAGTAGGCTTCCACTTGGACCTGAGATGTGA

>CsWRKY48

ATGGATGATGGCTACAAGTGGAGGAAGTATGGGAAAAAATCTGTGAAGAATAGTCCCAATCCCAGAAATTACTATAAATGTTCAAGTGAAGGATGCAATGTGAAGAAGAAGGTGGAAAGAGACAGAGAGGATGCAAACTATGTGATTACTACTTATGAGGGAATTCACAACCATGAAAGCCCCTTCGTTGTGTATTACAATCAATTGCCATCCTTTACCTCTGCCTCTACTCCCACCTAG

>CsWRKY49

ATGGGTTCAAAATCCCAAGTTATGTTGAACCCACAAGCTTTGTTGGAAGATCATCAAGAAGTGACACCAAATTCTCAAATGGGTTTCTTCAATTTCCCTTCAAATTTAACCTTTTTTCAACTTCCTTCAATCCCTCAAACCCATTCTCCTTCACCTTCATTTGATCCTCCAAATTTCTCCACTTCCAATAACAATACCAATAATAATAATAATTCCAATAATCTTTCAGAAACCCTACTTTCTTCTTCTATTTTGCCTCTCAAATCCTCCATTTCCTATGAACTTGCACCCCAACATCTTCTTTCCTTGCAAACATCCACTCCAAATCTATGGCCATGGGGAGAAATTGGAGAAAGGTTATTGATGAATGGGAAGAGATCAAATAATAATGAGAATTATAATAATCAATTGGGAGTATCAAAAATGAAGATGAAGAAAATGAAAGGAAGAAGAAAAGTGAGAGAACCAAGATTCTCATTCAAGACCATGAGCGATGTTGATGTTCTTGATGATGGTTACAAATGGAGAAAATATGGACAGAAAGTGGTAAAAAACACACAACATCCCAGAAGTTATTATCGTTGTACACAAGATCATTGTAGGGTTAAGAAAAGAGTAGAGAGATTAGCTGAGGATCCAAGAATGGTGATTACAACTTATGAAGGCAGACATGTTCATTCTCCTTCCCATGATTCTGAAGATTCTGAAGCTCAAACCCATCTCAATAATTTCTTTTGGTAA

>CsWRKY50

ATGACAAAAAGCGAAGTGGATCATTTAGACGACGGTTACAGATGGCGAAAATACGGTCAAAAAGCCGTTAAAAACAGCCCTTACCCAAGAAGTTATTACCGTTGCACCACCGCCGGCTGCGGCGTCAAAAAACGAGTCGAACGATCCTCCGACGACCCTTCCATCGTCGTCACCACTTACGAAGGCCAACACACTCATCAAAGTCCAATCATGCCACGTGGAGCTTTATCCTCCACCGCCTTCACCCCCTCACCACAACAACAACCCCCGCTTGTTTTCTCACAGCCCCAACAATTATACCGCAACCAATTTACGTACGCTCCGGCGCCGCCTGCGGACGTTGTCACCTGCGGCGGAGGGTTCGGTCACGTGTTTCACAGTTTTGGAGAAGAACGACGTCTCATCGATGGTAGAACCACCACCACTACTTCTACTACTACAACTGATTCTTTCCAAGACCATGGCCTTCTTCAAGATATGATCGTACCTTTCCCAGAAGAGAAAGAAAAAAAAGTTAATTAA

>CsWRKY51

ATGGAATTTTTTTTCTCTGCTACATTAGACGATCGTAGTGGCAACGGCCGCCGGACACAGCCGGACCTTGCGGTGGTGGCCATGGGTAATCCGCCGTTGTCTCCACCACTTTTCTCTCCTTCTTCCTTCTTCACTATTCCTCCTGGTATCAGCCCCACCCAATTACTTGATTCCCCTCTTCTTCTCAACTCTTCTCGTATTCTGCTATCGCCGTCCACCGGAGATTTGAGAAAATCAGCAAGTAACTGTTCCGGCCACCACCAACAGAATGTCAAACAAGAACACAACAACATTACTAAATTCTCATTTCCTCCTAATCACACCACAAAATCATCATCATCATCATCAATCTTTCAATCCTCTTCAACATCTGAGGTGCAAACTCAGGCATGGGGTTTGGAATGGGAGAATGACGACCGGGGAGATGGATGGGGGATGAGGAACAGATCAGGATCTGAGGATGGGTTTAATTGGAGAAAATATGGGCAAAAAGTGGTAAAAGGAAGTGAAAATCCTAGAAGCTATTACAAATGTACATTCCCAAATTGCCCTGTTAGAAAACAAGTTGAAAGATCGTTGAATAACAATGGTCAAATTACTGAAATTGTTTACAAAAGTAAACATAACCATCCAAAACCAGACTTCACTAGAAGGTCATCTTCCTCTTTTTCTTCTTCTTCTTCTTCACTCTGTAACCACGCCGGAGAACTCTTCAATCACCATTGGGGATGA

>CsWRKY52

ATGATAAGAGGAGGAGGAGGGGTGAAAACAATGAGGGAAGAAAGGATTGTAGTTCAAACAATTAGCAATGTGGACAAATTAGATGATGGTTACTGGTGGAGAAAATATGGACAGAAAGTGGTCAAGGGAAATCCAAATCCAAGGAGCTATTACAAGTGCACATATCCAGGATGTGGAGTGAGGAAGCATATAGAGAGGGCATCCCATGATTTTAGAGCAGTTGTCACTACCTACGAAGGGAAGCACAACCACGATATTCCGACGGCCCGTGCCGGAAAACCAATACTTAGTAATCAGCAGGGTAGAAATAATGAGGTTGTGTCGTCGAGCATTGGTGGTGATCCACGATCGAGTTCAAAGATTTGTAGTGGTACTAATGGATTATTGAGGTTGACAAATATGTTGTGGAATAACAAAGAAGAGGTCGTCGATAACATATACGAGTTGTTATAA

>CsWRKY53

ATGTATGAATCTCCTTCCTCTTTCAATGGTGGAAGTCCAAGGAGTGAAGATTCTGACCGTGAATTTAAAGACCCATTTGATCTAACCAATGCTAATTCTTTTCGCAAAAGGAACATTCTTCCAACATGGACACAGAAATTCCAAGTTAGTCCTGGGATGGCCATTGAAGGGTCTCTTGATGATGGATTTGCCTGGAGGAAATATGGTCAAAAAGGGATTCTTGGTGCCAAACATCCAAGAGGGTATTACAGATGCACACACAGGAACCTCCAAGGTTGTCTAGCAACCAAACAAGTTCAACGATCCGACGACGACCCGACCATCTTCGAAATAACGTACAGAGGGAAGCATAGCTGCAGCCAAGTTTCAAACCTCAGCACTCCATGTACAACAACACCAGAGTTTCAACAACAAAATCAGGGTGTAGTAGTAGAATTAACTGACCAAAAAAAGGCCCATAACCAACAAACAGCACCTGATGCTCTCTTGGACTCATGGTCATCCTTAAGAGTTATAACTCAAAACCTTGACACAACTCCTTTGAGCTATGACCGTGTCGAGTTTGCTTCAGCGTCGACTGTGGACGTGAACTTCGCGGAGTTTTCTTCATTCTTGTCCCCAACAACATCTGGTTCTGGGTTGAGCTATTTCTCTGCCTCATCAAGTGGGTTGAGTGAAGGGTTTGTTGGGAATCAGAATTTGAATAATTTGCAGCCAAACAATTGTGAGATTTTTTCCTCACCAACTTCAGCTTTAAACACTCAAACTACTACCGCCTTAGATTTCTCTTTTGGCGAACTTCAAATGGAACCAACTTTTTCATTTGACAACACAGACTTCTTCTCCTAA

>CsWRKY54

ATGGACCCCACTGATTCCGACCTCCCCGATCCATCTAATGCCTCCTCCGGTGCTAAGTACAAGCTCTTGTCTCCCGCCAAGCTTCCGATCTCCAGGTCCCCTTGCATTACCATCCCTCCTGGCCTCAGTCCCACTTCATTTCTCGACTCTCCTGTAGAACCTTCTCCGACTACTGGGTCCTTTACCAAGCTTCCAATGGCACATGACTCTTCTGGCTCAGCGATTTATCCAATGACCAGCATGGCTTTCTCAAATACTAACGCCTCGGATGAAGGAAGATCCAACTACTTTGAGTTCAAACCATATGTTGGACCAAATATGGTTCCTGCAGATTTGAGTCATAGGAAAGGTGAACAATTTTCTGAAGTTCAAGGTCAACCTCAACCTTTTACTGCTCCACCCATGACTAAAATGGAGATCAGTGTCATGTCAAATGATTTGAGTCGATCAACCCAGATGGACACACATACAGTTGCCTCAGGGGTTAGTGTTCCTGAAGCCAATGGAGATGAGATCAACCATAGTTTGAACACAAATAGCAGGGTTCAGGCTCCACAATCTGATCCAAAAGGCAGTGGTATTCCAGTAGTTTCAGATAGGTTATCTGATGATGGATATAACTGGCGGAAGTATGGACAGAAGCATGTTAAAGGGAGTGAATTTCCGCGTAGCTATTATAAATGTACCCATCCTAACTGTGAAGTGAAAAAGCTCTTTGAACGCTCCCATGATGGGCAAATAACTGATATTATCTATAAAGGTACCCATGATCATCCTAAGCCTCAGCCAAGCCGGCGATATTCTGCCAGTGCTTCTATGAATGTTCAAGAAGATGGGACTGATAAGCCTTCATCTTTACCTGGCCAAGATGACAGGTCTTGCAGCATGTATGCTCAGACAATGCATACCATTGAGCCAAATGGAACTACAGATCCATCCATGCCTGCAAATGATAGGATCACTGAAGGTGCAGGAACAACTCTGCCATGCAAGAATCACGATGAGGTTGATGATGATGACATATACTTGAAACGGAGGAAAATGGAACTTGGTGGTTTTGATGTCTGCCCAATGGTTAAACCAATCAGGGAACCGCGTGTTGTGGTTCAAACTCTAAGTGAGGTTGATATACTGGATGATGGGTATCGCTGGCGGAAATATGGTCAGAAGGTAGTGAGAGGAAATCCCAACCCGAGGAGTTATTACAAATGCACAAATGTTGGATGCCCTGTTCGAAAACATGTTGAAAGAGCATCCCATGACCCAAAAGCTGTTATAACTACATATGAAGGGAAACATAATCACGATGTTCCCACTGCAAAAACTAGTAGCCATGATGTCACAGGCCCATCGACAATTCCATCATCAAGGTATAGACTCGAGGAGAGTGACACCATAAGCCTTGATCTCGGCGTTGGCATTGGCACAGGTGGTGAGAATAGATCAAATGAATACAGGCAGGCATTGCATTCACAACTTGTTGAAAACCGAGCTCCAAGTGGGAATTTCAACTTTGAAGTAGTTCAAGAGAACTCGGCACCGACCTATTTCGGTGTGCTCAATAGAAGTGTTATAAATTAG

>CsWRKY55

ATGGAAAGCGGGTGGAGCTGGGATCAAAAGTCACTCATTGGTGAGCTAATTCAGGGGATGGAGCTTACCAAGCAATTGAGAGCAGAGTTGGGTTCAGCATCTGGAGAAGAAAGCAAAGGATCATTAGTACAAGGGATTTTATCTTCATATGAGAAAGCTCTTTTGATACTGAAATGGAACGGACCAATGAATCAGCTTCAGATGGTTGAAGCGACCCCTGGTTTGCCAAGCTCTCCAATTTCGGTTAATGGAAGTCCTTCTAGTGATGACTCTGGTAGAGTTCTCAAGGACCCTCAGGACTCCAGAAAAGAATCAAAGAAGAGAAAGACACAGCCCAGATGGACAGAACAAGTGAAAGTGAACTCTGAGACGGGATTCGAAGGACCCCATGAGGATGGTTATAGCTGGAGAAAGTATGGCCAAAAGGACATACTTGGTGCAACATATCCGAGAAGCTACTATAGATGCACTTTTCGCAATACTCAGAATTGTTGGGCAGTAAAGCAAGTGCAGAGATCAGATGAAGACCCTTCAGTGTTTGAGATTACATACCGAGGAAAGCACACATGTTCCCAAGGGAACTATTTAGCCCAAACATGTCATTCACCCGATAAGCAAGAACAGAAGGAAACTGACGGCGATCATCATGAGCTGCAGCCTTTGCAAGAAAACCTATTCGGTAATCAAACCATTCAGAACATCGAAAAGCTCGAAAATAAGGCATCTACCTTCTGCTTTGGCTCAAGCTCAACTTCTGTTGGATGTAAGGACATTGTAAATGCTGGCTTTTCAAGTTTAGCCATCGACACTCATTCTGCCTTGGGAACCTTTCCTCAGTCATTTACCTCCCCAACCTCACCTGACAAAAACTACTTCACTCCATCCCCATGCCAAAGAAGCAACGTAGGAGGGACTCATAGTGTGCAAAATCTAGATCCTGATGTCCATGAGATTTTCTCGGCCAACACTTCAGCTACCAATTCCCCTATCCTGGATTGGGATTTTCCATTTGATTCAGATCACATCAATCCAAATTTTCCATTTAATTCCCAAGGATTTTTCTACTAA

>CsWRKY56

ATGGAAAAGAAATTTGAAGAAGAATCAAGGATGGGGTTTGATGATTCCGCCGGGTTTTACTCTCCGGCGGTGTTTTCAGATGACTTTCCGTCTTCTAATTTTGATTCTTTTTCCAGTATTTTCGACATGCCATGTGATGCCCATAAAGCTTCTAATTTTGATTTTTATTATTACAATAACAATAATAGTAACAGTAATAATAATCCTTCTTCTTTCTTCGATTTGCTTTCTACGGCGGCTCCCCCACTTTCTTCACCGGCTTCCACCGTGCCGGAATCTTCCGAGGTTGTTAATGCTCCAACCACTCCTAATTCTTCCTCTGTTTCTTCTTCATCCAATGAAGCTGCCGCCATTGAAGAAGTCAACAACAGTACTACGACTCATGACAAGCCTTCTGCTTCTAAAGTGTTGAAGCCAATAAAGAAGAACCAGAAGAAGCAAAGAGAACCCAGATTTGCTTTCATGACAAAGAGTGATATTGATCATCTCGACGATGGTTACCGGTGGCGAAAGTACGGTCAAAAAGCTGTGAAGAATAGCCCTTACCCCAGAAGCTATTATCGTTGTACAACGGCAGGGTGTGGTGTGAAGAAGAGAGTAGAGCGGTCGTCCGGCGACCATACGATAGTCGTTACAACGTACGAAGGCCAACACACTCACCAAAGTCCAATAATGCCTCGAGGGAGTCTCAGAGTTTTACCAGAATCCACCAACAACAGCCTCACCGTCGATCACGACACCACCGCCACCGGACTTTTATTCCAACACAACACTTCCCAACCCTTCATGTATATCGCTCCACCCCCGCCATTTCTAACAATCAACTCATCGTCGGTGGCGGCCAGCCATAATCCTCCTCCTATTTCATTTCAGCCACCGTCGCCGCAAGCTTCCGTCCGAGACCATGGGCTTTTACAGGACTTAGTGCCATTGCAAATGAGAAAGGAACCAAAAGATGAGCAAAATGGATGA

>ClWRKY1

ATGGAGGCTGCTGCTGCTCCTTTCGGCTGTCCCCGGCCGGTCGTCAAGACCGAGAAGCCCGCTGGTGATGGCGACAACAATCAAGATTCTCCCTCTAAACAACATCTTCTCGTTAAGAGAGGGAACTATGCAAAACAAGAGCATGAAGCTGAAGATAAAAGCAACTCATCTGATCAAAAAGATTTAAGCTGCACAAACCTTCAGGAAAAGCAACTGGAATCCGCTCGAGCAGAAATGGGAGAAGTGAGAGAAGAAAACCAAAGACTTAAAAAGAGTTTAAACCAAATCATGAAGGATTACGAAGCCCTAAAAATGCAATTCCTAGGGATTGTCGGACAAGAATCGAAGAAATTTCCAAATCAAGACGACATGTCGAACAAGGAACAACAACACGACCAAATCGAGCTGGTTTCACTGACGCTGGGGAGATTTCCGGTGGCGGAGAAGAAAAAAACGGCCGATGAAAAGAACTCTGTGAATATTATTATTGGTGGCAACGACGAAGAAGCTGCCGTTAAAGAGGCATTATCTCTTGGTTTGAATTGCAAATTCAAACATGAAGAATCAATTATGACGGTTGCCGTTAAAGATGTCGATTCTCCGAAAAGTTTTGATCATGAGATGAGGGAGGAAGCTGGAGAGACGAATTGGTCGTCGACTAAAGGGGCGAAGACGATTAGAAGTGTTGAAGATGATGTTACGCCGCAAAATCCACCCAAACGTGCTAGGGTTTGCGTTAGAGCCCGATGCGAAACCGCTACGATGAACGATGGTTGCCAATGGAGGAAATACGGGCAAAAGATAGCAAAAGGAAACCCATGTCCACGAGCATATTATCGTTGCACAGGCTCACCAACATGTCTCGTTCGAAAACAAGTCCAAAGATGTGCTGACGACATGTCAATCTTAATCACCACCTATGAAGGCAATCACAACCACCCATTACCCGCCTCCGCCAACGCCATGGCCTCCACCACCTCGGCCGCTGCCTCCATGCTCCTCTCTGGCTCCTCCACCTCCACCGCGGCCTCCTCCTCGTCCACTAATACAAATAATCTCCAGGGCTTAAACTTTTACACAAATAATTCCAAACCAAATTTTTACTTACCTAATAATTCTTCAATCATTTCATCCACTTCCCCAACTCACCCCACAATCACTTTGGACCTCACTTCAAATCCTTCTTCCTCTTCCTCAAATTGCTCGACCCATTTTGGTAAATTCACATCTAGTTTTCCTAATTCTCGTTATCCTTTTACTAGCCAACTTGATTTCGGATCTTCTAGAAACAATGTGTTGTCGTGGAATAACGGCCTTCTTAGTTACAACAGAAATAACCACCAAACTACTACTACAACTACCAATAATATCTATCAAAATTACATCCAACAACGAAACCCCGCTACGTCACTGCCACATCAGCAACCGCCTTTACCCGACACCATTGCAGCCGCTACGAAGGCCATCACGGCAGATCCGAGCTTCCAATCTGCTCTTGCCGCTGCTCTTACGTCGATCATCAGCACCGGGGGTGCTAGTGCTGGCCCCACCAAGTCAACGTCGGCGAGAGGTGAACAATCCTTGTTTCAGTTGATGGCGGCGACGACAACAAATAAAGGAAATGGATGTGGGACGAGCTTTTTGAACAATATTACGACATCGACGACGACGAGTAATTCACCACCGACGGGGAATATGGTGTTTGTTCCGGCGAGTTCGTTGCCGTTTTCGAGTTCGAAGAGTGCATCGGCTTCTCCAGGTGATCATATTGATCTTACCAATTAA

>ClWRKY2

ATGTCAGATGAAATGTTTAAAGATCTTTTTTATGGCGGGATGGATGAGTACGAGTCTCTAGTGAGAGCTTTTGGGGAATCATCGGATTATTCGAATAATAATAATGAAATTTCGGGGACTCCCATAAATTCTTGTGGATCTTTGTCGTCTTCGGATGCTGGAGCTGAGGAGGATGATTCTGTGAAAGACAAGGATAAGCAGATCGACAAAGAACATGTTGAAGATGGAGGAGAGAGTTCTAAGGCTGGATCAGGTAAATCAAAGAAGAAAGGAGAGAAGAAAGAGAGAGAAGCGAGAGTTGCTTTCATGACGAAGAGCGAGGTTGATCATCTTGAAGATGGATATAGATGGAGAAAATATGGACAGAAGGCTGTTAAGAACAGTGCTTATCCTAGGAGCTATTACAGATGTACAACTCAAAAATGCGGAGTGAAAAAACGAGTAGAGAGATCCTTTGAAGATCCATCCATTGTAATTACAACCTACGAAGGTCAACACAACCACCCAGTCCCGACAACGCTAAGAGGAAACCTATCGGCGGCGAGCAGCGCGTTCTCGCCATCCATGCTAGCACCAATGCCAGTGGTCGGTGGTATTGGGTTTCTACCTACAGAGTTGTTGAGTAATTCTTCCAGCAACAATAACCAGGCCATCAATGGTGGCGCATCCGTTTATTCACATAACAACTTTGACTACACTTATAATGAGCGACAATCAGAATATGGGCTTCTACAGGATATTTTTCCAACTCCTTCATCGTTCTTCAACCGGCAATTGTGA

>ClWRKY3

ATGTTGTTGTTGTTTGAGATGGAGAATTATGAAGAAGGTGATTTGACTGATATTGTGAGAGGAGGAAGCTCAAGATCAAGATCAACAACAAGAAGCTCCACTTCTCATTGCAAAACGGAATTGGGTGATCAAGATGAAGATCAAGATTCTCCCTTTTATTCTTCATCACAAGATAACAATTACAATCCTCAGTGGAGTAGCTCCCAATTTTCATCACAACTACTACTACTACAAGATCATCATCATCATCATCATCATCAATACAGTTTTGGGGATCCATTTTGTAGTACTGTTGCTGCTTCACTTGATCTTCATCAAGAGCTGGATAATACTAATAATAATACTTTTTTCAATGGAATTACTACTACTACTACTACTTCTACTCAAGATGAGCTTATCAAAACGCCTTCTTCTTCCAATATTTTCTCACGTATGCTTCAGATCTCTCCTTCACCCGACAAATTCCCAACCATTTCTTCACTTAATTCCTCTTCTAATTTCCTAATTCCCAATCATTCTCTTAACTCTCCCACCACTCCACATTCTGATCACCTTCATCATTTTCTTCATCATAATGATAATAATCCCTCCCCCTCCGCCCTTCACATCTCTTCTCCTCGCAATCCACCCGGTATCAAGCGAAGGAAGAGCCAAGCAAGGAAAGTGGTTTGTGTTCCAGCTCCAGTGGCTGCTAGTAGTAGACCAAATGGGGAAGTTATTCCTTCTGATCTGTGGGCTTGGAGGAAATATGGTCAGAAACCCATTAAAGGCTCTCCATATCCTAGGGGGTACTATAGATGCAGCAGCTCAAAGGGATGCTCAGCAAGAAAACAAGTGGAGAGAAGTAGAACAGATCCAAACATGCTGGTGATTACTTACACATCTGAGCATAACCATCCATGGCCAACTCAAAGAAATGCTCTTGCTGGTTCTTCAAGGTCCCAACAATCTAAAAACAACAACATTACTACTACTACTTCTTCAACTTCAACAACAACACCAACACCCAATTCTTCAAAACTCTCTCAAGATCATCATCACAAAAATAAGGAAGAAAAAGAAGAGCATCAAGATCAAAACAATGGAACATTATTATCAGCTGCAGCAACAACAACATCAAATAACAATAATAATGTTAAGGAAGAGGAAACTGAGAACCATCATCATCAATCGATGATGAGTGAAGGGTTTGATGATGATCATGATTTCTTTGCAGATTTGGAAGAATTAGAAACAGACCCTTTAACTCTGTTGTTCACTACACAACAACCCCTTAAATTAGACCAAATCAAAGAATCAGCCACCACCACCCCCACTACTACTACAGGTTGCTTACACGACGTCGTTCCATTCAATAATCTATTTGATTGGCCACCACCTCCTCTAAACAACAATTCAACTCCATTTCATGAACAACCACCACCTACTACTCCTACCAACGGAGGTTTTTATTAA

>ClWRKY4

ATGGCTGTGGAGATCAAGTCCCACCCTTACCCACTCCTTACTATGGAAGCCAACGCCGTCCAAGAAGCCGCTTCCGGCCTCGAAAGCGTCGAGAAGCTCATCCGATTACTTTCTAATGCAAATGCCCCTCAACCTCACTCCTTACCCTCCTCCACTCAATCCCCCATCGATTTCCCCACCGACTGCCGCGCCGCTGCCGATGTAGCCGTTTCCAAGTTCAAAAAGGTTATTTCTCTCCTCGGCCGTAGCCGCCTCGGCCACGCTCGTTTCAGGAGAGCTCCTCTGCCTCAACAACCTCACTACGCTACTCCCATACAGCAGATCCCACCCCATCCCCACCTTGACAACAACCACAACAATAACAACGACTCTGTTAATTTCTCCGCCCATAATTCCTTCATTTCCTCGTTGACCGGCGACGCCGACACCAAGCATCCTTCTTCCTCTTCCTCACCTTTTCTCATTACCAACCTCTCCCAAGTCTCCTCCGTTGGGAAACCACCTCTTTCTACTTCTTCCCTCAAGAGGAAGTGCAGCTCAGATAACTTGGGATCCGGCAAGTGCGCCGCTGCTTCTTCCTCTGCCCGATGTCACTGTTCCAAGAAGAGAAAACTGAGGGTGAAGAGGGTGGTGAGAGTTCCGGCGATAAGCTTGAAAATGGCGGATATCCCACCGGACGATTATTCATGGCGGAAATATGGTCAAAAGCCCATTAAAGGCTCTCCACATCCCAGGGGTTATTACAAGTGTAGCAGTGTGAGAGGTTGCCCAGCCCGAAAACACGTAGAACGGGCCGTAGACGATCCGACGATGCTAGTGGTGACTTACGAAGGAGAGCACAATCACACACTCTCTCTGCCCGAAACCTCCAGTCTCATCCTCGAGTCCTCTTAG

>ClWRKY5

ATGGACAAAGGATGGGGTCTAACCCTCCGTGATTCTGATCAGTCAATTGGGTTCTTCTCAAACAAGCCACCACCACCGCCGACCACTGTCAATTCCTTTCAAAGAATGTTTCAAGGTTTAGAATTCCCCGGAAAACTTGGCCGGACTGACGACACCGACGCCACTCCGTCTCTAGCCGACGAGAATCGGCTGCCCGTCAACGAGGTGGACTTTTTCTCCGATAAGAAAAGAGTGGTTGATGATCGGGAGGATCAAGACTCTAAACCTTCCACTAATATTATCGCCACCACCGCCATCAATAAAGACGATAAGCCTTTTACCGCTCCCAGAACTTCCTTCAATCTTGTTAACACTGGGTTGCATCTGTTAACTGCTAACACTGGAAGCGATCAATCAACGGTGGATGATGGAATTTCTTCAGATGGTGAAGATAAACGAGCCAAAAACGAGCTAGCGCAACTTCAAGTGGAGCTTCAGCGTATGAACGCTGAGAATCACAAGCTTAGAGACATGCTAAGCCATGTGAGCAACAACTATAGTTCTTTACAAATGCATCTCTTGACCTTAATGCAACAGCAACAACAAAATCAGGCTTCCGAACCCGCTCATGAAAGAGAGATTGGGGAGAAGAAATCGACGGAAATAAAACATGAAGTTGGAAGGGTAATGGTACCAAGACAATTTATGGATCTAGGACCAAGTGGGAACAACAACAACAACAACACGGGTGAAAGCGATGAACTATTATGTAATTCCTCGTCGGATGAAAGAACTCGTTCTGGGTCTCCATTGAACAACAACAATACCACCGAAACCGCCTCCAAAAAAAGAGATCACGCGGAAATCACGGCTCCTTCCGATCACGAAAATTCCAAAAGACCCAACCCCAGAGAAGAAAGCCCCGAATCAGAATCTCAAGGTTGGGGTGGTCCGAACCATAAGCCCCCTCGGTTCAACTCTTCTAAACCACTTGACCAATCAACCGAAGCCACCATGCGTAAAGCCCGTGTCTCAGTCCGTGCTCGATCCGAAGCTCCCATGATTTCTGATGGGTGTCAATGGCGAAAATATGGCCAAAAAATGGCTAAAGGAAACCCATGTCCACGAGCCTATTATCGCTGCACAATGGCCGTGGGTTGCCCAGTTCGTAAACAAGTCCAACGTTGTGCTGAAGATAGGACTATATTGATAACAACTTACGAAGGCAACCACAACCATCCACTTCCGCCGGCAGCGATGGCCATGGCGTCAACTACGACGGCGGCGGCTACCATGTTGTTATCAGGGTCTATGTCAAGTGCAGACCATAATTTAATGAACCCAAATTTATTGGCTCGAGCCATACTTCCATGTTCTTCAAGCATGGCTACAATTTCAGCTTCGGCTCCATTTCCAACCATCACATTAGACCTCACTCACACTCCAAACCCATTACAGTTTCAAAGACCCGCAGCGACGCCTTTCCACGTGCCATTCCCCGGCGGACAACCACCGTCGGCCGCCGTTGCCGCCCAGTTGCCTCAAGTTTTGGGGCAAGCGTTGTATAATAATCAATCAAAATTCTCGGGGCTACAGCTTTCTCATGAGATGGGGGCTAATTCCTCTCATTTGGGTCATCACCAAATTACACAACCGGCGACTCCGGCCCAGCCTGGTGGTGCTTCTTTTGCCGACACGTTGAGTGCCGCCACCGCCGCTATTACCGCCGATCCTAATTTCACCGCCGCTCTCGCCGCCGCTATCTCCTCCATCATCGGCGGAGCTCATTCGAATAATAATAGCAATACAAACACCACCAATAATACTACAACAACAAACAACAACAACGGAAGCAGCAACAACAGCAAAATTAGCAGCTTCCCTGGAAATTAA

>ClWRKY6

ATGCTTAGATCTTCACTGGATCTTGGGATCCGACCCCATTTGGGAGGATTTGTCGTTCGAGCTCGGGATCAAGTAGTAATGGGGAGGACTGATGATAATGTTGCTATTATTGGGGATTGGGTGCCTCCAAGTCCCAGCCCAAGAACCTTTTTCTCAGCAATGCAAATGCTAGGGGAGGATATTGGTTCTAGACCTGCCATGGACACTACCAGCAGTGATCATAAAACTGAAGAACTCTTTCTCAGGCCTCGAGAACATACGGTATCTGAAAATGCCGTTGCAAGAGGCGGGATCCCGGGTGCCAATTCTGGTGACCGAGCAACAGAGTTCGGTACATTCTCGGAGCAGAAATTTCGTGGGGGACTTGTGGAAAGGATTGCAGCCAGAGCTGGATTTAATGCTCCAAGATTGAATACAGAGAGCATCAGATCAACAGATCACTCTTTGAATTCAGAAGTAAAGTCTCCTTACTTGACAATACCACCTGGTCTCAGCCCAACTACATTGCTAGATTCACCAGTTTTTCTTTCAAATTCACTGGCTCAACAATCTCCCACAACTGGAAAGTTCCAATTTTTAGCAAATGTTAGTAACAATCGGAGCTCGACGATGATGTCGGAGGCCAACAACAATCCGTTCGATGATAACAGTACATCATTTGCTTTCAGACCCAGTGTGGAATCTGGATCCTCCTTTATTCTCAGTGCAGCAGCAAGCAAAACAGCTTCTGCTACTATTCTTCCACAATCTTGTCCAAGAATTGAGGTTCCAGTTCCACGTTCAGAAAATTCTTTTCAATCTCATCTTGTAGAACCATCTCTGTCTTTACCCCAAAATCGAATTGGTCACCATCCTCAAGTGGGACTCTCTACATCGTACATGGAGAAGGATGATGTGGGTAAGACTGTGTCGGATGATCAAAGACCTTTTGATTCTCTGTGTGGTGGTGGTGAACATTCTCCGCCACTCGATGAGCAACCGGATGAAGGGGAGCAAAGAGGCAGTGGGGATTCTATGGCTGGTAGTGGTTGCGGTGCGCCCTCGGAGGATGGATATAACTGGAGAAAATATGGACAAAAACAGGTTAAAGGAAGTGAGTATCCTCGGAGTTATTACAAGTGCACACATCCAAATTGTCAGGTCAAGAAGAAAGTTGAACGATCTCACGAGGGCCATATAACAGAGATCATCTATAAAGGAGCACATAACCACCCAAAGCCCTCACCAAATCGACGAGTAGCAATTGGATCTTCTGACTCCCATATCAATATGCAACCAGACATCCCTGCACAAGCAGCAGGCCAACAGAGTGCTGAAGTTTCAGTTTGGGAAGATTCACAAAAAGGGATTCCAACTGGAGCTCCTGATTGGATGCAAGAGAACCTTGAGGTGACTTCTTCAGCATCCTTGGGTCCTGAATATGGCAATCAGCCTAACCCTTTACAGGCTCAAAATAGCAGTCATATTGAAACAGCTGAGGCCATTGATGCCTCATCCACATTCTCTAATGATGAAGATGAAGATGATAGGGGAACACATGGCAGTATAACATTGGGATATGAGGGGGAAGGAGATGAATCTGAGTCTAAGAAAAGGAAACTCGACGCCTATGTAACAGAGATGAGTGGGGCCACTAGAGCTATCCGTGAGCCGAGAGTTGTCGTTCAGACTACCAGTGAAGTAGATATTCTGGACGATGGCTATCGTTGGCGCAAATATGGACAGAAGGTTGTGAAAGGAAACCCAAATCCTAGGAGTTACTACAAGTGCACGAATCCTGGCTGCACAGTGAGGAAGCATGTTGAGCGGGCGTCACATGACCTGAAGTCCGTGATAACCACATATGAAGGAAAGCACAATCATGATGTTCCTGCTGCTCGCAACAGCAGCCACATCAGTTCTGGTACATCCAGTCCAGTAACGGGTCAAAACTCGACCGCAGCAATGCAAACTCATGTTCACAGGCCAGGGCCACCCCAGCCTCAAAACACCATTCCAAGATTTGAAAGGCCAGCTTTTGGCTTTGCTGGAAGACAGCAGATGGGCACTGCTTTTGGAATGAACCAACCTGGACTGGGAAATCTGACCATGGCAGCTGTTGGCCAAGCCAAGCTTCCTGTTATGCCAATGCATCCTTACTTAGCACAAGCACACCATGTTCATGAAATGGGTTTCTTGTTGCCTAAAGGAGAGCCCAATGTAGAACCTACATCTGATCTTGGCTTGAACTTTTCCAATGGTTCAACCGTGTATCAGCAAATTATGAGTAGGCTTCCACTTGGACCTGAGATGTGA

>ClWRKY7

ATGGACCCCACTGACTCCGACCTCGCCGATCCATCTCATGCCTCCTCCTCCGCTGGCGCTAAGTACAAGCTCATGTCTCCGGCCAAGCTTCCGATCTCTAGGTCCCCTTGCATTACTATCCCTCCTGGCCTCAGCCCCACTTCCTTTCTCGACTCTCCTGTTCTGCTTACCAACTTGAAGGTAGAGCCTTCTCCGACTACTGGGTCCTTTACCAAGCTTCCGATGGCACATGACTCTTCTAGCTCAGCTATTTATCCAGTGACCACCATGGCCTTCTCAAATACTAATGCTTCAGATGAAGGAAGATCCAACTACTTCGAGTTCAAACCATATGTTGGACCAAATATGGTTCCTGCAGATTTGAGTCATAGGAAATGTGAACAATCTTCTGAAGTTCAAGTTCAAGGTCAACCTCAACCATTTACTGCTCCACCCATGACTAAAATTGAGATCAATGTCGTTTCAAATGATTTGAGCCGATCAACCCAGATGGATACACATCCAGTTGGCTCAGGGCCTAGTGCTCCTGAAGCCGATGGAGATGATATCAGCCATAGTTTGAACACAAATAGCAGGGTTCAGGCTCCACAATCTGATCCAAAAGGCAGTGGCATTCCAGTGGTTTCAGATAGGTTGTCTGATGATGGATATAACTGGCGGAAGTATGGACAGAAGCATGTTAAAGGGAGTGAATTTCCTCGTAGCTATTATAAATGTACCCATCCTAACTGTGAAGTGAAAAAGCTCTTTGAACGCTCCCATGATGGGCAAATAGCTGATATTATCTATAAGGGTACCCACGATCATCCTAAGCCTCAGCCAAGCCGGCGATATTCTGCCAGTGCTTCTGTGAATGTTCAAGAAGATGGGCCTGATAAGCCTTCACCTTTAACTGGCCAAGATGACAGATTGTTCAGCATGTATGCTCAGACAGTTCATAACCTTGAGCCAAATGGAACTACAGAACCATCACTGGCCGCAAACGATAGCATCAATGAAGGTGCAGGAACAACTCTGCCATGCAAGAATCAGGATGAGGTTGATGACGATGACATATTCTCGAAGCGGAGGAAAATAGAACTTGGTGGTTTCGATGTCTGCCCAATGGTTAAACCAATCAGGGAACCACGTGTTGTGGTTCAAACTCTAAGTGAGGTAGATATACTGGATGACGGGTATCGCTGGCGCAAATATGGCCAGAAGGTAGTGAGAGGAAATCCCAACCCAAGGAGTTATTACAAATGCACAAATGTTGGATGCCCTGTTAGAAAACATGTCGAAAGAGCATCCCATGACCCGAAAGCTGTTATAACTACATATGAGGGGAAACATAATCACGATGTTCCCACTGCAAAAACTAGTAGCCATGATGTCGGGGGTCCATCGACAATTGCACCATCGAGGTATAGACTCGAGGAGAGCGACACCATAAGCCTTGATCTTGGCGTTGGCATTGGCACTGGCACGGGTGGTGAGAATAGATCAAATGATTACAGGCAGGCATTGCATTCACAACTTGTTGAAAACCAAACTCCAAATGGCAATTTCAACTTTGAAGTAGTTCAAGAGAACTCAGCCCCGACCTATTTCGGTGTGATCAATAGAGGTATAAATCAACATGGATCGAGAGAGAGTTTGAGTGAAAGCCACAATAAGGAGATTGCTCCGCTAAATCATTCGTCTCACCCATATCCGACGAGCATTGGGAGAATACTAACAGGGCCTTAA

>ClWRKY8

ATGGCTGCTTTCTCCTCTTGGTTGCTGGATTCTGTTGACACTTCTTTAGATCTCAATACTCAACCTCTCAGATTCTCCGGTGAAGCTCCGAAGGAGAGGAATTATATGGATCTTGAGAGGAAGGTTTCTGTGAAAGAAGAGACTGGAGCTTTAATGGAGGAATTGAAGAGAGTAAGTGCAGAGAACAAGAAATTGACTGAAATGTTGACTATTGTGTGTGAAAATTACAACAATTTGAGAGGGCATTTGATGGAACAAATGAACAAAAATGGAGAAAAGGAGATTTCTTCTTCAAAGAAGAGGAAGTCAGAGAGCAGCAATAACAATAACAATATGGCTGGAATGAATGGAAATTCAGAGAGTAGTTCAACTGATGAAGAGTCTTACAAGAAACCAAAAGAAGAAACCATTAACAAATCTGCTAAAATCACTAGAGTTCAAGTCAAAATTGGAGCTTCAGATTCAAATCTAGTTGTTAAAGATGGATTTCAATGGAGGAAATATGGCCAAAAGGTCACTAGAGATAATCCTTGTCCTAGAGCTTACTTCAAATGCTCTTTTGCTCCTAGTTGTCCTGTTAAAAAGAAGGTTCAAAGAAGTGTAGAAGATCAATCAGTTCTTGTAGCCACATACGAAGGCGAACACAATCATCCACACCCATCTCAGATTGAGCCCACTTCCGGTGGCGCCGCCGCCCGTAGCGTCAGCATAGCTCCGGCGGTGCTCACAGCAGCGCCGGGATCCTCGTCGGCGCCGGCGATTTCACTCGACTTGGCGAAGCCGAAGCCGACCATGGAAGCTAAAACGACCTCCAACCCAAGATTTGACTCGCCGGAACTGCAACAGTTTCTCGTCGAACAGATGGCCTCTTCCTTAACGAAAGATCCCAATTTCACGGCGGCGCTTGCGGCGGCGATTTCGGGAAAGATTTTCCCACATTGA

>ClWRKY9

ATGGAAGAGATTGTAAAGAAGGAAGACACTAGCACAAGCACCGACGGCGGCGATGGTACTGGTGCCCTTTTCCCATTTTCCGACAACGTGATACCAAACGGATTCTTTGATTTTTCTGATGCTGAAAAATGCTCTGTTGGGTTTATGGAGTTGCTTGGTCTTAATAATTGTGAATTATTTAAGGATTATTGTTCTGAGGCTTTTAATCCTCCGGCCACCCCCAATTGTTCCTCCTCTGTCTCCTCCGCCTCTAGCGACGTTGTTAACGACGACGACCCACAACAACAACACAACCCCACCACTAAACAATTGAAAGTGAAAAAGAGGAAGGAGAAGAAAGAGAAGGAAGCAAGATTTGCATTCATGACAAAGAGTGAAGTTGATCATCTAGAAGATGGCTATCGATGGAGAAAGTACGGCCAAAAAGCTGTCAAAAACAGCCCTTTTCCTAGGAGCTATTACCGTTGCACTAGTGCATCTTGTAATGTAAAGAAGAGAGTTGAACGATCTTTTGCTGATCCAACCGTTGTGGTGACCACCTACGAAGGCCAACACACTCACCCAAGCCCCATCCTAACTCGGTCCGCCCTAGCCGTCACCATTCCACCACCGCCCCCCACCGGTGGCCCGGGTGGAGGGTGTGTCGGCGTTGCAGCCATGCCATGGCTCAAAGCTACCAACAATGCTCATGATGGTGACATTATTCCAGCATTATCTCATCAATACTTTCAAAACTCCTCCTTCGTCACTCCCCAAAATTCAGCCGTAAGCTACAACCGAAACAACCCCATCGGAGCAGCGAACGCGGCCGGGATCCTCCAGGAGAAACGGTTTTGCAACCCAAATGCTACAATATTTCTTGTAGACCATGGCCTGCTTCAAGATGTTGTTCCTCCCCACATGCTGAAACAGGAGTGA

>ClWRKY10

ATGGGCTCTCTCTTCATCTTCTCCATCCTAGTCCTTTCTTTAATAGCAAACAACCTCTCCTCTCTTTGCTTTTTCTCTTTCAAATGCTCAACAAAAAGCATAGTCATAGCCATAGCCATAGCCATGGAGCTTTCTTCTGATCATTCTCTTTCTCTAAAACCAAACCCAGATGATGAACATCGTCTTTCATCTCAACCCACACCAGAAGAAGCACCTTCCAAAAAAAGAAAAGTTGTGCAAAAGACTGTTGTTACTGTAAAGATTGGGTCTAAAAAAGCTGCAATAGGGATAGGGAAGGTGAAGAATGAAGGGCCACCTCCTGATTTTTGGTCTTGGAGAAAATATGGCCAAAAACCCATCAAAGGATCTCCATATCCAAGGGGTTATTATAGATGTAGCACAACAAAGGGCTGTTCAGCAAAAAAACAAGTTGAAAGATGCAAAACAGATGGTTCAATGTTCATCATAACATACACTTCAAGCCATAATCATCCAGGTCCCAACATATCCACTCTCAATTTGGATCAAAACCAAGAAGAGATTCAACCACAGCCGTTGGATCAAGATGATGATCAAGATCTTGTTCCAAATCAGGGGCTAGAAAAGCAAGATCAAGATCATAATAATGATGAGAAAAACAGCATTATTAGTCCAGGTGAAGAAGAAGAAGAAGAAGAAGAAGAAATAGAAGTAGAAGAAGAAGAAGAAGAAGAAGAATCATTATTAGCTATGGAAGATGAGGAGAAAAAGGGAATAGAAAAGGTAAAGGAGTGTTTGGAAGAACCAATTTCTTCTTCATGTTGTCACGAGCTCATAAATTTGTCAGCCACAACAAACAAGTCAGAATTAGAAAATCATGATCATTTCTTTGATGAGCTTGAAGAACTGCCCACACCCCCACCTTTTTCAAGCTATTTTTTTGATGAAATTAGGATTTCTGCTGCCCCTTCTTGA

>ClWRKY11

ATGGACAATAAAGCGGCAGAGAGAGTTGTTATTGCCAGACCAGTTGCTTCTAGGCCAACATGTTCCAGTTTCAAGTCATTCTCTGACATCCTTGCGTGTGCCTTCAATACTTCTCCACCTAATATGTCATCCGAAACCAAAGTTGCTGCCATTAGACCGAAGACAGTGAGGTTTAAGCTGAAGGATAAATATTTTCAGGCCAAAATATCAGAAACGGTCTCTGGAATGAACTCTTGTAGTTCATCCGAGAATCTTGCTATATCAGACAGCAAAACCACTGTTTTATTCAAACCTTTGGCGAAGCATGTATCAAAAAGGACCGTCTCTCAGCTGTCGCTCATAGGGAACACCAATTTGCAAAACCGTTTACCACATCCACCAGTTGAAGTCTGTATTCAATGTCCAAATCAAGGTGAAGACAATTTCGAATCTGCACTGACCTCAAATCTCTGTATTCAATGTCTGAATCAAGACAAAGACAATTTCCAATCTGCACTGACCTCAAATCTTCCCCAGAACATCACATCCACTGTTGAAAACAGTCAATCTATTGAAAGCTCAAGAGTGACATTAAGTTATAGCAAAGAGGATCCAACATTGCTTCGTCCTCAAATTACTTGTGCTCAGCCTTCTTATGATGGATATAATTGGCGGAAATATGGGCAAAAGCAAGTTAAGGGAAGCGAGTATCCACGGAGTTATTACAAGTGCACACATCCAAGTTGTCCTGTCAAAAAGAAGGTTGAGAGATCATTAGATGGGAAGGTCGCTGAAATTGTTTACAAAGGTGAGCACAACCACCCAAAGCCTCAGCCTCTAAAGCACAACTCATCTGGGACACAAACGGAAGGGTCAGTATCAAATGGAACTACACGGGATACTAATCCAGAGTTGTGGCTTAATTACCTCAATGGACAAATAGAAGGTTGTGAAAGTCGGCTTGAGAATCACGTCGAAAAAACTTGTCAAGGCAGAGTTACATTACCCTTCGATTCGGTTGCAACTAGAGAAGTGCAAGGTGGATGTGGAATCTCTGATAATTCATGCGGTCTAAGCATAGAATGTGAAGAAGGAAGCAAAGGACTTGATTCCACGGATGATAAATTGCAAAGTAAAAGAAGGGGTGGCAAAAATCCAACAAATGAAGCTGTTACATCAATTGAAGATGTTAATGAGCACCACACAATGGCTCGAGGTTCCACTGGTATCGAGATTTCTGGCAAAGGCATTCGCTGGAGAAAATATGGGCAGAAAGTTGTGAAGGACTCGATCTCACATAAGCTTTCATTTTGCAGAAGTTACTATAGATGTACTGGTCTCAAATGCAAAGCACGAAAGTACGTCGAACGAGCATCTGAGGATCCAGATTCCTTTATCACTACTTATGAAGGAAAACACAATCATGGCATTTCACTTGGAACTGAAAATCCTGTAGCTCCTGAAATGGAATAA

>ClWRKY12

ATGAATAACATAAATCAAACGATAAATACACTCGCTGGCGGATCTTCTGACAACAGAACCAATAATTTCGGTATGGAAGTTCCGAAATTCAAGTCTCTTCAACCTCCTCCATTTCCCATGTCTCCTTCTTCTTACCTTTCAGCTTTCTCTTCTGGTTTAAGCCCCACTGAGCTTCTAAATTCCCCTCTCCTTTTCTCATTTGGTGTTTTTCCTTCTCCTACTTCTGGTGCCTTAAATTTGAGAAATGATTATGAGGAGGGTGAGCAGCAAGAAATGAAGGCAGATGTGAAAAATTACTCTGCTTCTTCGTTTAACCCACAAACAGGATCCTCTGTTTCGTCTTATTTTCAGTCCTCTTCTTCCAACGTGACACCACTGAATCCGAACGGTTTGTCGTGTGATGAAAGTGGAGCGAAATCAGAATTTGTTACTACAGAAATGGCGGCAGCTGAATCAAAGCCAATTTCTCAGCTACCAATTTACAACAGAGAACAGAAAAAATCAGAGAACGATGGATATAACTGGAGAAAATACGGGCAAAAACAAGTGAAAGGAAGCGAAAATCCACGAAGCTATTACAAGTGCACTTTCCCAAGTTGCCCAACAAAGAAGAAAGTTGAAAGATCTTTAGACGGCCAAATTACTGAGATTGTTTATAAGGGAAGCCATAATCACGCCAAGCCCCAGCCCACAAGACGTTCCGGTAACTCCGCGATTTACGATCCATCTTCGGCGGATTCCGCCCTGCTGCAGGAGGATTCCTCCGTCTCGGTGGGGGATGAGGAATTCGAACCCAATTCGCCGTTTAGCAACTCGGTGGACGACAATGAAAATGAACCGGAAGCTAAGAGATGGAAAGGGGAAAATGAGAACGAGGGATATTGTGAGGGTGGAAGCAGAACAGTGAAAGAGCCAAGAATTGTGGTTCAAACAACCAGCGAAATTGACATACTGCCGGATGGTTATAGATGGAGGAAATACGGACAAAAGGTTGTCAAAGGAAATCCAAATCCGAGGAGCTACTACAAATGCACATCTTTAGGCTGCCCGGTGAGGAAACACATCGAGAGAGCGGCTAACGACATGAGGGCCGTGATCACTACCTACGAAGGCAAACACAACCATGAAGTTCCAGCAGCCCGTGGCAGCGGTGGCAGTGGCTATAATGCCATCAACCGACCTACACCGACCAACATTCCAATGCCGGTGAGGCCGTCTGCCATCGGTAGTCATTCTTTCCCGGCAAATTTTCCGGCCACGTTCCAGCCGGCAAATTTAGCCATGTCAGAAATTGGAGCAGAAGCATCATCATTCCCATTTCAAACATCACAAGGAGTGCCTCCAAGTTTCCAAGTTTCAGGATTTGGATCCGCAGCCAAGGAAGAAGCCAGAGATGACATATACTTCATCAACTCATTTCTATCTTAG

>ClWRKY13

ATGGATTGTTCCTGGCCTGACACCACGCCCTCCGATCGAAGAAAAGCAGCCGACGAACTGCTTCGTGGCCGTGAACTTGCACAACAACTACGAGCCTATCTACAGAGAACTTCCAATTCCGGTGGGACGGCCTCCCAAGATCTGCTCTCCAGAATCCTAACCTCTTTCTCCAAAACACTTTCCATCTTGAATCGCTGCGACTCCGATGACATAAATGGCTCTATTGTGGATTCACCCGAGGATCGTGCTAGTAGAAAATCCCAGGAATCTGGGGACAGTTGCAAGAGCTCCGATCGCAGGGGTTGCTATAAGAGAAGAAAGAGTTGCCAGAGTTGGGCGAGAGAGAGCTGCAGCCTGGTGGACGATGGGCACGCGTGGAGGAAGTACGGGCAGAAGACCATTCTAAATGCGAAATACCCAAGAAACTATTACAGATGCACCCACAAATTCGACCAGGCCTGCCAAGCCACAAAGCAAGTCCAGCGACTGCAAGACCATCCCCCAAAATTCCGTACTACTTATTATGGCCATCACACCTGCTCCAATTTCCTCAAAGCTTCCGACATCGTGCTCGGCTCCTCCAATTTCGACGATTCCTGCGGAGTGCTCCTCAGTTTTGACACCCCCGCCGCACCCAACTTTTTACTCCAACAGGATGCTATGTTGGTCAAGAAGGAAGTTGCAATCGCCGAAAGCAGGGATGATGAGGCCGTATGTTCCCCCTCCGACTACATTAGTACGGCCGAGCCCTCGCCCGACGACCATCTCTCCGAGGTTTTCATGGGTTCCGTCGTCGACTTTGAGGATGATGTTTTACAATTCCAGTTTTGA

>ClWRKY14

ATGGATCATCAGTTCCAAGAAGAAAGTAGTGTGAAAAAAACAAGTAGTGGATCTATGAAATCGGAGAAGAAGATAAGAAAAGGGAGATTTGCGTTTGAAACCAGGAGCCAAGTTGATGTGCTTGACGATGGCTATCGCTGGAGAAAATATGGTCAAAAGGCTGTTAAGAACGACAAGTTTCCCAGGAGCTACTATAAGTGTTCACATCAAGGATGTAAAGTGAAGAAGCAAATTCAACGGCTTACAAATGATGAAGGAGTGGTATTAACAACCTACGAAGGAGTTCATTCCCATCCCATCGAAACCCCTCAAGATAACTTTCAACATATCTTGACTCACATGCAAATTTACCCTTCTTCCTTTTAA

>ClWRKY15

ATGGCTAAGAAGGACGATTCTGCAAGGCCTCCACTTCAGCGCCCCACCATTACTCTGCCGCCGCGCCCTTCTATGGAGGCTTTTTTCGCTGGCGGACCCACTGGAGTTAGCCCTGGCCCGATGACTCTTCTCTCCAGTTACTTCGCCGACGGTGCCGTTGACTCGCCTTCCTTCTCCCAGCTTCTCGCCGGAGCTATGGCCTCTCCGATGGCTATGGGGTTTTTCGGAACTGGGTCCACACCCAATTATTACGCCAAGGATGGAACCGCCTCGGAATTGGAATTTGGGATGAAACAATCGAAGCCGCTGAATTTAGTGGTAGCTCGGCCTCCTTTGTTCTCGGTCCCGCCGGGGCTTAGTCCTTCTGGGTTGCTTAATTCGCCTGGGTTTTATCCTCCTCAGAGTCCATTCGGAATGTCGCACCAGCAAGCATTGGCTCAGGTTACCGCTCAAGCGGCATTGGCAAATTCTCATATGCATATGCAACAAGCTGAATATCAACATTCTTCGGTACCAGCTCCTACAGAACCGTTGACACGGGATCCATCATTTTCTCTTGATGAAGCATCTCAACTGGCCATATTACCCTCCACTTCAGACACAAAAAGTCTGATTGCAGAACCGACAGAAGTCTCTCACCCTGACAGAAAATATCAACCTCCTCCTCCTCACGTCTCTGATAAACCTGCAGATGATGGCTATAATTGGCGTAAATATGGACAGAAGTTGGTTAAGGGCAGTGAATATCCCCGAAGCTATTACAAATGCACTCATTTGAATTGCCCTGTAAAAAAGAAGATTGAGCGCTCACCTGATGGTCAAATTACTGAAATTATTTACAAAGGACAGCACAACCACGAACCTCCTCCAGCCAACAAACGTGCAAGAGATAATAGTGAACCGAGTGGATGTACAAATTCTTTGATGAAGCCTGAATGTGGTTTGCAAAATCAAGCTGGAATTTTGAACAAGTCAAGTGAAAATGTGCAATTAGGGTCTAGTGACAGTGAAGAACGAGCTGATACAGAGATAACAGATGACAGAGATGAAGATGAGCCAAATCCAAAGAGGCAGAACATTGACGCAGGGACATCTGGTGTTGCCTTGTCACATAAAACCCTCACAGAACCAAAAATCATTGTTCAAACAAGAAGTGAGGTTGACCTGTTAGATGACGGTTATAGGTGGCGCAAGTACGGGCAGAAAGTGGTTAAAGGAAATCCTAACCCTAGGAGCTACTACAAATGCACTAGTGCTGGGTGCAACGTCCGAAAGCATGTTGAGAGATCTTCAACAGACTCGAAAGCTGTTGTTACTACATACGAAGGGAAACATAACCATGATGTTCCTGCAGCTAGAAACAGCAGCCACCACACGGTCAACAATACTGTCCCCCAGATCAAACCACACAAAGTTGTAGCTCAGAAACATCCATTACTTAAAGAGATGGAATATGGAACTAATGACCAGAGACCTGCCGTTTTGCGGCTTAAAGAAGAGCAAATCACCGTGTAA

>ClWRKY16

ATGGCGGTGGAGCTTCTCACAGCCTTCACCAACGCTCAACTCTCTTCTCCAATGGACCAAGACTCCGCCGTCCAAGAAGCCGCCTCCGGCCTCGACACCCTCAAAAAGCTCATCACATTACTCTCTCACTCTCACCCTTCTAATCTCGACTCCGATTGCCAAGCCGTCGCTAACGCCGCCGTCTCCCACTTCCGAAAGGCCATTTCTCTCCTCGGCCGCACCTCTAGAACCGGCCACGCCCGATTCCGCCGTGCCCCTTTGGATTCCTCTAAAATTTACAACGCTACGCCTATTCAACAAATTCCTCCGCCCCTCGACCGCCTCGATTCCGCCACCACCATCAATTTCTCTTACTCCGCTGCTCCTAGTTCCTCCTTCTTAACCTCGCTTCCTGGTTCCGACTCTGAAATTAAGCTCCAACATCAACCTTCTTCCTCCTCTTTTCAGATTACTGATCTTTCCAGAGTTTCGTCTGTTGTCTCTAAGCCTTCTTCTGGTCTCAAGAGAAAGTGCGGTTCTGAGAATTTAGGCTCAGGGAAGTGCGCTGGATCTTCTGGTGGTCGTTGCCACTGCTCTAAGAAGAGAAAAATGAGGTTGAAAAGGGTAGTGAGAGTACCAGCTATAAGCTCAAAGAACGCTGATATTCCTCCTGATGATTATTCGTGGAGGAAGTACGGGCAAAAGCCAATAAAGGGTTCTCCATATCCAAGAGGTTACTATAAGTGTAGCAGTTTAAGAGGATGTCCAGCAAGAAAGCATGTTGAGAGAGCCTCAGATGATCCATCTATGTTAATAGTGACTTATGAAGGCGATCATAATCACTCTCAATCAGTTGCAGAAGCCTCAAGTCTCATCCTTGAATCTTGGTGA

>ClWRKY17

ATGGACGCCGCCGCACCTTCCCCTTCCCCTCCTCTGCCTACTCCCACTCCCCTGCAATTTCCGGTTAACCTCAATTCCACCCTCCCCGATCCCAACTATTCGCCTCCTCCTCCTCCTCCTCCTCCCCCTCCGCCACCCCCTCCTCCTCCACCCTCCTCCTCCCACCGCCCCTTCTTCGACGAAATGAACTTCTTCCCCGCCGATGATAAATCCCGCGTCCTTCTCTCTGCCTCTCACTCCAATCTCACTCCCACCAAACTCGACTTCAATGTTAACACGGGATTGAATCTCTTGACCACGAATTCGTGTAGCGATCAATCCATGGTGGATGATGGAGTTTCACCAAACCCAGAAGAAAAAAGAATAAAAAACGAGAGAGCAGTTCTTCAAGCTGAATTGGAGAGGATAAACTCGGAGAATCTACGATTAAAAGACATGTTAAATCAAGTGACGAGCAATTACCAAACACTGCAGATGCAATTCAACACGCTAATTCAAACCCAGAAAACAGGGGACGCCGGTGACCCGATTGAGGAAAACGCCGGCAGTGGCGGAGGGAATAATAATAATAATAATAATAATAATCATAATAATATTGGTAATAAGCTGGTGCCCAGGCAATTTATGGATCTTGGATTAGCGACCAATATGGAGAACGATGAGCCGTCAATGTCGTCGTCGGAAGGGAGGAGTGGAGACCGGTCCCGGTCGCCAGGAACCACCGGAGAAGTAGCGTCGTCGAAACGGCATAGCCCAGATCAATCTTCCAATTGGGGTTCCAATAATAACAACAGCAACAACAATAATAAAGTTCCAAAATTCAGTTCTTCTTCGGGTAAAGATGTGGATCAAACTGAAGCTACCATGAGAAAGGCTAGAGTCTCCGTTCGAGCCAGATCAGAAGCCCCCATGATAACAGATGGATGTCAATGGAGAAAATATGGACAAAAAATGGCGAAGGGAAACCCTTGTCCACGAGCTTATTACCGGTGCACCATGGCCGCCGGCTGCCCAGTTAGAAAACAAGTACAAAGATGTGCAGAAGACAAAACAATTTTGATAACAACCTACGAAGGAAACCACAACCACCCATTGCCGCCGGCCGCCATGGCAATGGCTTCCACAACTTCATCGGCGGCCAGAATGCTTCTCTCAGGATCCATGTCAAGTGCAGATGGTTTAATGAACTCTAATTTTTTAGCAAGAACCCTATTGCCATGTTCTTCAAGCATGGCTACGATCTCAGCCTCAGCCCCATTTCCCACAGTCACATTGGACCTAACTCAAAACCCTAATCCTTTATTCCAACGCCCGGCTGCGGGTCACTTTCCTATCCCGTTTGCGGCCGGTCCGCCCCAAAGCTTCCCACAGATCTTCGGACATGCATTATACAATCAATCAAAATTCTCAGGCCTTCAAATGTCAAAGGATATTGAAGCACCACCACCTCCACCACCGCAGAATCCATTGGCCGACACGTTGAGTGCTGCGGGTGTAGCAATTGCATCCGACCCGAATTTCATTGCGGCATTGGCAACGGCAATGACGTCGCTGATCGGCGGATCGCATCATCAGAATGAGAATGGTAATGGCAGTAGTAATGTTGATAACAATACAAATAGCAACTCCCAACAGTAA

>ClWRKY18

ATGGGTTCTAAATCCCAAGTCCTATTGAACCCACAAGCTTTGTTTGAAGATCATCAAGATCATGAAGTGGCAGCAAATTCTCAAATGGGTTTCTTCAATTTCCCTTCAAACTTAACTTTTCTCCAACTTCCTTCAATTCCTCAAACCCATTCTCCTTCCCCTCCATTTGATCCTCCAAATTTCTCCATTTCTAGTAATAATACTAATAATAATAACAATAATAGTAATTCCAATCTCAATGAAACCCTACTTTCTTCTTCTCTTTTGCCCCTCAAGCCCTCCCTTTCCTATGAATTTGCACCTCAACACCTCCTTTCCTTGCAAACATCCACTCCAAATCTATGGCCATGGGGAGAGATTGGAGAAAAGTTGATGAATGGGAAGAGATCAAATAATGAGAATCATCAATTGGGAGTGTCAACAATGAAGATGAAGAAAATGAAAGGAAGAAGAAAAGTGAGAGAACCAAGATTCTCATTCAAGACCATGAGCGATGTTGATGTTCTTGATGATGGTTACAAATGGCGAAAATACGGACAGAAAGTGGTCAAAAACACACAACATCCTAGAAGCTATTATCGTTGTACACAAGATCACTGTAGGGTAAAGAAACGAGTAGAAAGATTAGCTGAGGATCCAAGAATGGTGATTACAACTTATGAAGGAAGACATGTTCATTCTCCTTCCCATGATTCTGAAGACTCCGAAGCTCAAACCCATCTCAATAATTTCTTTTGGTAA

>ClWRKY19

ATGGTTATGGAGTTTTTGTTCTCTAGTAATTCTGTAGAGGATCCTAGTGGCGGCCCAACCTTTGGCCATCGGAGACTGCCGGAGCATGTGGCGGCCGCGATGTCTAAACCGCCATTGCTCCCCCCCTTGTCTCCACCTCTTTACTCTCCTTCTTCTTTCTTCACTATCCCCCCTGGTATCAGCCCCACCCAATTTCTTCACTCCCCTCTCCTCCTCAACTCTCCTCATATTCTGCCATCGCCGTCTACCGGAGCAATATCCGCCGGAGATTTGACCGGAACCAGTAACAATTCCGGCCACCATCAACAGAACATCAAACAAGAACACAACAACTTTACTGAACTCTCATTTCCTCGTACTCATCACACCACAAGATCTTCATCATCATCAATGTTTCAACCCTCTTCAACAGTGCAAAGTCAGCGATGGGGTTGTGAAGCCGCCATGAACGGCGGCTCCGAAGCGGAGCAGCGGGCAGAATGGGAGTTGAGAAAACGAGGAACTGAGGATGGGTTTAATTGGAGAAAATATGGGCAAAAAGTAGTAAAAGGAAGTGAAAATCCGAGAAGCTATTACAAATGTACATACCCAAATTGCCCTGTAAGAAAGCAAGTTGAAAGATCATTGAATGGTCAAATTACTGAAATCGTTTACAAAAGTAAACATAACCATCCTAAACCAGACTTCACAAGAAGGTCATCATCGTCTTTTTCTTCTTCTTCTTCTTCTTCTTCTTCTTCATTGTTATCAGAAGCTTTACCCGCAGCTATGGCGGCAGAGCCAAAGAATTCAATTGCCTCTGATTCCCTCACCACGCCGGAGGATTCTTCAATCACGATTGGGGATGATGATGATGATTCCCACGGAGCTGATGCAAAAAGATGGAAAAGTGAGAGTGAGAAGGAAATTATGTCTTCAGTAGGAGGGAAAACAGTGAGAGAACAAAGGGTTGTAGTTCAAACAATTAGCAATGTGGATAAATTAGATGATGGATATTGGTGGAGAAAATATGGACAGAAAGTGGTCAAGGGAAATTCAAATCCAAGGAGCTATTACAAGTGCACATATGCAGGATGTGGAGTGAGAAAGCACATAGAGAGAGCATCCCACGATATAAGGGCAGTCATCACTACCTACGAAGGAAAGCACAACCACGAAGTTCCGGTAGCCCGTGGCAGCGGCGGCGGTAGACCGATACTTATTAGTAGTGAAGGAAGTAATAATGATGTTGCATCGAACGGCGGTGGCAACCGACGACCGAGTTCAGAGATCTGTAGTGGTAATAATGGATTGTGGGAGTTGACAAACATGGTGTGGAGGAGCAAAGAAGAAGCCGTGGATGACATATTTATGGGTTGTTATTAG

>ClWRKY20

ATGTCAAATGAAGATCATCATGAGAAGGAGGAGGATCCTTATTACTCACACTTTGATCCTTTCACCCACAACTTCCATCATCACAAACCCTTTGAAGCTCCTCCTTTAACAAGCCCTTATTATGAAGCTTTTGATATTGTTCCTTCTAGTGTGGGCTTGTACTCTGATTTCTTACATGCCTCAGCTGAATATGGCTACAACAACAACAACGGCCTCCTCCCTCCTGCCCACGAGATGTCGTGCTCGTCGTCTGATGTTATTTCTCCGATCGACGATGCTTCAAAGAAGTCGTTAGGCCTTGGAGATCAGTTGGTGGTGACCGGTGAGCACCCCTCGACACCAAATTGTTCCTCGACCACGTGTTCGTCCGATGAGGCTGCGGCGGGTGGTGAGGATTCTTCCAAGATTGGGGAGGTGAAAGGGTTTGATGATAAAAATGGAGAAAATAGCAAGAAAGTGAATAGAGGAAAAAAGAAGGAGAAAAGAGAGAAAGGGCCACGATTCGCTTTCTTGACGAAGACTGAAATTGATAATCTTGAAGATGGATACAGATGGAGAAAATATGGACAGAAAGCAGTAAAAAACAGCCCTTTTCCAAGTGTGAAAAAAAGAGTAGAAAGATCATCGGAAGATCCATGTTTTGTTATAACAACGTACGAAGGCAAACACAACCACTATTGTCCAATTACACTTCGAGGCCACAATCCCGCCGGAGTTTTGCCGCCGTCCGTTTCACCGCCGTACCTTTCGGGATTAGCTCCGGCGAACTTCTTCTCCGGCGAGTTCTATGAAAATTTGCAGCAACAATATTATGAGGTTCCTGATGAGTATGGGCTGTTGCAAGATTTGATTAATCCATCATTTAATTCGAAGCAAGACCCATCAAATTAA

>ClWRKY21

ATGGTTTCTTCAGGGGACCAGTTAGAGAATGAAGTTGATTCTGATCAATTGGAGCATGAAAACAGTTCAGAAAGTCAACCGCAAACGTCTCAGGACGATCCTGGTGGAACTAATGCATCAAAATCAGATCACAAATGCACAGGGGCTGCCAGTATTACACTTGAAGAAGCTGTCAAGCAGCCTGAAGTTACAATCGAACAAGTGGATCGAGGAGAAATCTCTAATATAGTGACTGAGAAAGTGACCCATAAGCCAATTACTGCAGAGCAGAACTCCCATTCTGATTTGAAAGTATGTATTACCTCTACTATACGAGAGAAAGTATCAGAGGATGGGTATAACTGGCGGAAATATGGTCAGAAACTGGTTAAGGGAAATGTCTTTGTTAGAAGCTATTACAGATGTACGCATCCTACTTGCATGGTGAAAAAACAACTGGAGCGCACTCATGATGGGAAGATCACAGACACTGTTTACTTTGGCCAGCATGATCATCCTAAAGCTCAACCTCACATTCCAGTTGCTGTTGGAGTCGTTACCATGGTTGAAGAAAAATTAGATGAACATGCTTCTGGAAATTCTCAAGATAAGACCTCCATTGCGCCTGGCCAGACACCTCATCAAACTGAGCTAGCTGATATGCGTCAACCGTCGTCAGTTATGGCCAGTGATGATGTAAAAGATGAAGCTTCGAAAAGGTCTAGGATTAATGATGAGGTTGACAGTGATGATACACCAGACTTGAAACGAGAGAAGAAAAGATGTAATATTGACGTGACAGTAGCAGACAAGTCAACTGTTGAATCTCGGGTTGTTGTTCAAACTCCTAGCGAGGTTGACATCGTCAATGATGGATACCGCTGGCGGAAGTATGGACAGAAATTGGTGAAAGGCAATCCAAATCCTAGGAGTTACTACAGATGCTCAAGTCCTGGATGTCCAGTTAAGAAACATGTAGAGAGGGCGTCTTATGATCCAAAAGTGGTGCTTACCACGTATGAGGGTCAGCATGACCATGATATGCCTCCTACAAGGACGGTGACGTTGAATTCAGTTGGATCCACAGCAGCCCATAGTGATGAGACAAAACCAAAACCGGTCGGTAGTTCCATTGGCCATGATACGGTGGTTCATGCTACTAAGGATTCTCTAAATAATTCGAGTTCTGAAGGCAAATTAATTGAAAAGAATGGCAAGTCGAATGCCACAGAAGCAAGTGATGGCATAGTCCTCGATATGGTGGTCAATCCTAGTCCGGGAGTTGCGAGTGGACAAAATAAGCAGCTCAAAGTAGCAATTGAAAGCTAA

>ClWRKY22

ATGGCCTCCTCTTCCGGGAGCTTAGACACCTCTGCTAATTCTCATCCCTCTTTCACTTTCTCTACTCATCCTTTTATGACTTCTTCTTACTCTGACCTTCTAGCCTCCGGCAACAACCATCCTCCTTCCTCCGCCGCACCCCACGCCGATCTCCGGGGTTCCGGCACCGGAGTTCCTAAATTCAAATCCCTCCCTCCTCCTTCTCTCCCTCTCTCTCCTCCTCCCCTCTCTCCTTCTTCCTTTTTCGCTATTCCTCCTGGCTTGAGTCCCGCCGAGCTTCTTGATTCCCCTGTTCTTCTCAGTGCTTCTCATGTTTTGCCGTCGCCGACCACTGGGAGTTTCCCGTCTCAGTCGTTGAATTGGAAGAGCAATTCTGGGTATAATCAGCAGAGCATTAAGGAAGAAAACAAATATCTGTCTAATTTCTCATTTCAAACTCAATCGTCAAAGCTGCCGCCGACGTCTTTTCAGCCTTCATCCACCACAGCTCCCACGACTCAGGGATGGACTTTTCAAGAGCAGCGCAAGAAAGAGGATGGTTTTTCGTCGGAGAAGAATATGGTGAAGCCGGAGTTCGGATCGATGCGGAGCTTCTCACCGGAATATGGGGTTGTTCAAAACCAAAACCAAACCAATGGCAGCGGGGAGTTGCAGTCTGATTACGGCAACAATTACCCTCAACAATCTCAGACGGTGAATCGAAGATCGGACGACGGCTACAACTGGAGAAAATACGGCCAAAAACAGGTTAAAGGAAGTGAAAATCCGAGAAGCTATTACAAGTGCACTTTCCCCAATTGCCCAACAAAGAAAAAGGTCGAAAGATCCTTAGATGGACAGATCACGGAGATCGTTTACAAAGGCAGCCATAACCATCCAAAGCCCCAATCCACAAGGAGGTCGTCGCTGTCGTCCGCCGGTTCTTCCCACACCATGGTGGCTTCGAATCAGGCCACCAGCGAGATGGCGGACCAGTCATTTACAACCCAAGGCAGCGGCCAATTTGATGGCGTTGCCACGCCGGAGAATTCCTCGATTTCAATTGGCGACGACGACTTCGATCGGAGCTCTCAAAAGAGCAAATCCGGAGGGGACGATTTTGATGAGGACGAACCAGAGGCTAAGAGATGGCGAAGAGAAGGTGACAACAATGAAGGTATTTCGGCGGTCGGTAGCCGGACGGTGAGAGAGCCGAGAGTCGTCGTCCAAACCACCAGCGACATCGACATTCTCGACGATGGTTACCGGTGGAGGAAGTATGGTCAGAAAGTTGTAAAGGGAAACCCAAATCCAAGGAGTTACTACAAATGTACAAATCCAGGATGTCCAGTAAGAAAGCATGTGGAGAGAGCTTCACATGATCTAAGAGCAGTGATCACAACTTATGAAGGGAAGCACAACCACGATGTTCCACCGGCACGTGGGAGTGGAAGCCATTCCCTCAGCCGTCCATTCCCCAACAACGAAGCTCCGGCAACTGCGATCCGGCCATCGGCGTTGACCCATCAGTCAAACAACGGGGGGCATCTGCAGGGTCTGAGGCTGCAGCAATCTTCAGATTCCCAAGCAGCATTCACAGTGGAAATGGTGCAAAATGGGAATGGATTTTCATTCCCAGAATTTGGAAACTCAATGGGAATGGGAATGGGAATGGGTTCCTACATCAACCAAACACAGCCCAGTGATAATTTGTTCACCAGAGCCAAAGAAGAGCCAAGAGATCATGACATGTTCATCCAGTCTCTCCTATGTTAG

>ClWRKY23

ATGGTGGAGGAGAATGGTGATGTACCTCTTGTTATGGAGGAAGCTAGTGGGGATGAAGCTAGCCAAGAGGAGAGCTTTGATATCACTGTCATTGCCGTTTCAGAGATAAGTGAGCTTGGAGACATCATTGAGGAAAGCATTGATATTATTGATATCCCAGGTACTGTAGAGGAAGCTGACATTGATCACAAAGCCTTTGAATCAGATAAAACACTGATGGATTTTGAATTCCTACCTGATTTTCCAAAAGAGGCTTCTGTATTGAAGTATGAAATTGCTCCAACCGCTGATAATAGTTGTTTTGATGGCAAGATTGCTAATGGCAACTGTGAGAATATGGAAAGTTGTCTTTCTTCTATAACTACTAATCAACCATGTATACCTGAAGAATCAACGAAAGGAGATGACATTGAGACGCAGCATCCTTTAGAAGATGAACAGAAAGGGTCTTACATTCCTATGGGAATGTTAAGGACATCAGAAGATGGATACAACTGGAGGAAATATGGGCAGAAACAGGTCAAAGGTAGCGAATATCCAAGAAGCTACTATAAATGTACACATTCAAATTGTCAAGTAAAGAAAAAGGTGGAGCGATCTCTTGATGGTCAAATAACAGAAATCATCTATAAGGGTGCTCACAATCATGCAAGACCTGATCCCAATCGTCGAGCCATGCTTGGATCCATGCCAACTCCAGGTGATACTCCAGAAATTGGTGAAGGTGGTGGAAACCGTTCCAAACTTGAAGCTGGGCTGGCATGGAGAAACACTCAATATGGGATGAAGGATATCAAACTTATCTCAGATTGTAGTGTTGTTGACGGTCTAGAGAGGACATCCTCTGTATCAGTTGTGACTGAGCTTGCTTATCCATTGTTAAATCCCCAAGGGAAAACTGTTGGTGTTCTTGAATCGGTGGGAACGCCAGAGCTCTCATCCACACTTGCTAGTCATGATGATGATGATGGTGGTGGTGGTGACGATGATGATCTTACAACTCAGGGAAGTATTTCAGTTTGCACAGAAGCTGATGATGCCGAACCTGAATTGAAAAGAAGGCGAAAAGAGGGCAGTTCAATTGAGACAAACTTGGCTTCAAGATCTGTACGTGAACCAAGAGTAGTTGTCCAAATTGAAACTGAAGTTGACATACTTGAAGATGGGTATCGGTGGCGGAAGTATGGGCAAAAAGTTGTCAAAGGAAATCCAAATCCAAGGAGCTACTACAAATGCACTAGTGCAGGCTGTTTAGTAAGGAAACATGTTGAGAGAGCATCACATGATCTGAAATGTGTTATTACAACATACGAAGGAAAACACAATCATGAAGTGCCTGCAGCAAGAAATAGCGGTCAAGTAAACTCGAGTAATGGCAATGCCCAACCGTCTGCTTCTCATGTGCAACCGAACATGGGTTTGTCCAGAAACTCAAACGTCCCAAAGTCTGAAACACAAATTCAAGATCTTGCTGCCCAGTTCTATCCAAAACCAGAATTTAACCATGACTATCAGAGGTCTGGTTGTTTTGACACTTTTACAAATGATATCAAACTTGGAGCTCCTTCATTCTGTCAAATGAAGTTCCCTCCACTCCGAAACACCTTGCCTTATAGTTCCTTTGGATTAAATTCAAAGCATACTGGAACAAGTATGTCTGGTTCTTTGGCATCCGTGGTCCCAGACTTTCCGATTTCCTTGCCATTGAACCAGAACCTTTCAGCTGCTGGTTATGATTTTACCAATGGAAGACCAATACCACCATTTCAGGTTTTCCTGGCTGGTCAGCAGCTGAGGGACACAGATAGATTCCTCACACCCAAACAGGAGCATGACGACGATAACATCTGTGCTTCTTTCCAACCAGTTGTTGATAGTTCGAGTGGATCTTCATCGTCATCAGTCTCATCGGTTTATCAACAAATCATGGGAAATTTTCCCTAG

>ClWRKY24

ATGGATGATAATGTTACCTTCAACAATAATAACCACCAATTCTTTTCGGCTGATCAACTTGAAGATTCCGACGAATCAAAACCCGCCCCTGATTCTCCTCCTCCTAATTCCGCCGCCGCCGCCACCGCCACCAAGAAGGGCAGAAGAGGGATGAAGAAGAAAGTTCTGTCGGTGAAAATTAATGGCGACAACCCAAGAAATTCCTCTGGTTCTACAACTCCACCTTCTGATGCTTGGGCCTGGAGAAAGTACGGTCAAAAGCCCATCAAAGGCTCTCCTTATCCCAGGGCGTATTATAGGTGTAGTAGTTCAAAGGGATGTCCAGCGAGGAAGCAAGTAGAGAGGAATCGGCTTGATCCAACTATGTTGCTCATTACATATTCGTGTGAGCATAATCATTCCGGTCCGGTTTCTAGGAACAACAATTCCAACCACAACCAAACCGATGCGGTGAAACCCGATTCACCGGAGACGGTTCCGGTTGAAGTAGAAGAGAAATTTGTGGAGATAGTGGGAGAGGAGTCGTTGATAACGGGAGATGAGTTTAGTTGGTTTGGAGAAATGGAAACGACGTCGTCTACAGTGCTTGAAAGCTCCATTTTTTCGGGGAGAGCATCCAGTGGATTGGTGGATCATTCAATTTCAAGTGACGTGGCAATGCTTTTGCCCATGGGGGATGATGACATGGACGAATCACTCTTTGCGGATCTCGGCGAGTTGCCGGAATGTTCTCTGGTGTTCCGGCGTGGCGGAGGTCGGGGATTACCGGTGGATGAACAACCGGCTGCTGCCCAGCGGCGGATCACACCGTGGTGTGGAACCACCACATGA

>ClWRKY25

ATGGACAACAATTATCAGCAAGCTGGTGATTTGACTGATGTAATTCGACTCAGTTCCGCCGCCGTTGCCGGTGGCCTCTCATCGGAATTCTCTCTCGACCCTTCTTCCAGTGACCGACGACTCTGGCCTCACCTAACCGCCGATGAATCTTCAATGAATTTTGGAGATCCGTTGTTGTTTCCGACTAGTAGTGCTCGAGATCCGTTTCTTCATCCTCATTTTTCTGCCTTCAATTTAGCTTCTGACGGCGGCAGTGAGGGTTTGGCGGCGGTGGACCAGCCGTCTAATTTACTTTCACATATGTTGCAGATCTCTCCGAGTTCCGGTGATGGTGTAATTAGTACTCCTCCATGTGAGTCGTTCGCCACGGCGGTGGGGAATTCTCCGTGCTCGGCTTCAGCTTGCAGTGGCGGTGTCGTGCTTCCGACCGGTGGCTCGAATCCTCTTTGTTTAATGGAGAATTCTGGGATTCAGATCTCTTCCCCACGTAATTCCACCACTAAAAGAAGGAAGAGCCAAGTGAAGAAGGTGGTGTGCATTCCGGCACCGGCACCGGCGAACAACCGATCAAGCAGCGGTGAAGTGGTTCCTTCTGATCTATGGGCATGGAGGAAGTACGGACAAAAGCCCATCAAAGGATCCCCATATCCAAGGGGATATTATAGGTGCAGTAGCTCAAAGGGCTGCTCCGCTCGAAAGCAGGTCGAGCGCAGTCGAACCAACCCCAACATGCTTGTCATCACCTACACCTCGGAGCACAACCACCCATGGCCCACTCAACGCAATGCTCTTGCAGGCTCAACACGCTCCCACCCTTCTCGAACCCCTACCACCCCCACCGCTCGTAAAACAAGCCCAAAACACGAGCGCACCAACAACAACCAACCAACCACAACACTAAAGGAGGAAGAGATGGATGCAGACCAAACGACAGAAAAAGCAATAGTAACAACAACAAACGATCATCAGGAAGATGGAGATCATCAGAATTTCCCTTATGACTTAATTTTCACTGAATTTGCTGACCATGACAACGACCACGACCATCATCATCTTTCAGCAGACCCTCTAAACAACATCATCTTTAGTAGCCATGGCTTTAGTACAGGAAATGGAGAAGGGAGTAAAGATCCATTTTTGGAGCTCTATGATTGGGCAGATAATTCAAATGGAAGTTTGTTCAAAGAAGCCAAGGGAGGTTGA

>ClWRKY26

ATGGGAACAGCCCACGTAAAAGCCTCTCCCCATATCCACCATTCCCATCTACTCCACTTTATCCTCTCTTCTTCTTCTTCTTCTTCTTCTTCTTCTTCTTCTTCTTCTTCTTCTTCTTCTTCTTCTTCTTCTTCTTCTTCTTCTTCTTCTTCTTCTTCTTCACCCAGGGAGCCTCTAGGAGCCGTTCCCTGGTCAGATACTTACAACACCGTCTCCGACGACCACCTCCTCACCCTCACTGGCCTTCTCCATGACGACGACGACGCCACCGCCTCCTCTCCGCTCTCCAAGACATCGGCGACAATCCAGCCGCCAAGCTCCCCGTGCCCAGTGGTGCGGCCTACTTGGGGCCGACAATTGAGGACATTGAGAATGCGCTCTCCATTGCCCCGAGATCGAAAGACCTGCAGTCCCACACTCACATTTCTCATACTGGGAAGCTTGAATAAGGTTGAGCATAAATATAGTCTCAGAATCAAGAGCTGTGGGGGGAATATGGTCGCTGATGATGGATATAAGTGGCGCAAATATGGTCAGAAGTCAATTAAAAATAGCCCCAATCCTAGGAGCTATTATAGGTGTTCAAACCCAAGATGCAGTGCAAAGAAGCAAGTAGAAAGGTCCATAGAAGATCCAGATACCTTCATCATCACCTACGAAGGCCTCCACCTCCACTTTGCTTATCCATTCTTCCTAATGGGCCAAAGCCCACAAGTCCAATCTCCAACCAAGAAGCCCAAGACAATCGACCCAGAGCCCGAAGCCTACAAAAGCCCAACTTTCCTCGCTCCAGGCCCACCGCTACCAGATGACCCAAAAGAAGCAACCGGTCCACAAGGGTTGCTCGAGGATATGGTCCCATGGATGATCCGAAATCCATCAACCAATCACAACGCTCTATCAAATTCGTCCTCTTGCTCTTCCCACCGTTCGCCTCCAATGTCCCCTCCATCACCGTCCACGTGTCCTACCTTCATTGCTTCCTGCTTTTGA

>ClWRKY27

ATGGAGAATTATCAGATGTTTTTCCCTTGCTCCAGCGGCGGCGATGGTGGCGGACGACTGTCGGCGTATCATCATGCCGACATGTCATCTGTTTCCGGGTCTGATATATTTGGGAATTTTCACGGCGGCGATATGGAGGTAAGTGGGGTTTTGGGGATGAAGACAGAGATGGATGCGGCGGCGGCGACGGCGGTGGAAGCGGACGGCGGCGGAAAAAAGAAAGGGGAGAAGAAGGTGAGGAAACCAAGATATGCTTTTCAGACAAGAAGCCAAGTTGACATTCTTGACGATGGATATCGTTGGAGAAAATACGGTCAAAAAGCTGTCAAAAACAACAAGTTTCCTAGGAGCTATTATAGATGCACGCATCAAGGATGCAATGTGAAGAAGCAAGTGCAAAGGCTAACAAGAGATGAAGGGGTGGTGGTGACAACATATGAAGGAATGCACACACACTCCATTGATAAGCCAACAGACAACTTTGAACAAATCTTGAGTAGAATGCAGATTTATAGTACTCCTTTTTGA

>ClWRKY28

ATGGAGAATTTTGTGGAACGGAATCAAAACAAGCTCAAAAATGAGCTGCTTAAAGGCAAGGAATTAGCAAAGCAACTCCAAATTCATCTCAATATGAGACCATCCGCATCATCCACGGCTGCTTCTTCTTCTTCTTCTTCTTCATTTTCAAATGATAATGGTGGTGAATTATTAGTTCAAAAGATTTTATGTTCATATGAAAAGGCACTGTCTTTGCTCAACTCAAATGGAATCCAAATATCTGAATCTCCTTCATCTATTAATGGAGGAAGTCCAAGGAGTGAAGACTCTGACCGTGAATTCAAGGACCTATTTGATCAAACAAATGCTAATTCTTCCCGCAGAAGGAACATTCTTCCAATTTGGACACAGAAATTCCAAGTTAGTCCAGGGATGGCCCTTGAAGGCTCTCTTGATGATGGCTTCTGCTGGAGGAAATATGGTCAGAAAGGCATTCTTGGTGCCAAACATCCAAGGGGGTACTATAGATGTACATACAGGAACCTCCAAGGTTGTCTTGCAACCAAACAAGTTCAACGGTCCGATGACGATCCGACTGTCTTTGAAATCACGTACCGAGGAAAGCATAGTTGCAGCCAAGTTTCCAATCTTAGTACTCCATCTACAACGACACCAAAGTTTCAGCAACAAAACCAACAAACATCACCTGATGCTCTCTTGAACTCATGGGCATCCTTAAGAGTCATAACTCAAAACCTTGACACAACAATTCATGAAACCCCAACATTGTCATTTCATTACGACGCGGCAGACCGTGTCGAAACGTCGACTGGGGACGTCAACTTTGCCGAGTTTTCTCCATGTTCTTCATTTATGTCTCCAACAACATCTGGTTCTGGGTTGAGCTATTGGTCTGCCTCATCAAGTGGGTTGAGTGAAGGATTTGTTGGGAATCAGAAGTTGGATTTGCAACCAAACAAATGTGAGATTTTTTCGTCACCAACTTCAGCTTTGGACTCTGAAACTCCTGCCTTAGACTTCCCATTTGGTGACCTTCAATTGGACCCAACTTTCACATTTGACGACACCAATTTCTTCTCCTAA

>ClWRKY29

ATGGCGGTCGATCTGATGAGCTTTCCAAAGATGGATGATCAAATGGCTATACAGGAGGCTGCATCGCAAGGATTGAAGAGTATGGAGCATTTGATTCGTCTTCTTTCTCACAAGCAACCCTCAAGCCATGTTGATTGCTCTGATTTAACTGATGCAACCGTTTCCAAGTTCAAGAAAGTGATCTCTCTCCTCAATCGGACCGGTCACGCTAGATTCCGGCGAGGTCCGGTTTCTTCGACATCCTCCTCGTCGTGCGGTTCATCTGCTCATCTCTCTTTTCCTCAAAATCAGGCGATGAATCTCACTCCTACTTCGTTTACTTCACCGGCCGCGGTTCCTGCTCCGCCGTTCACCGCTCCGGCTTCCGTGGCTCAGCCACAGGCGAAAGTCGTCGCTGCGGCGGCGAACATCCTTCAGTCGCAGCCTCAGAGCATGACTCTCGATTTCACTAGGCCGAATATTTTGAACTCAAATCCTAAGGGAACCGATTTGGAATTTTCTAAGGAGACCTTCAGCGTCTCGTCCAGCTCCTCGTTTATGTCCTCAGCCATCACCGGAGACGGAAGCGTATCCAACGGAAAGTTAGGAAAGTCGATCTTCTTAGCACCAGCGCCGACTGCTTCCGGTGGTAAGCCGCCGCTCTCGGCGGCGCCTTACAAGAAGAGGTGTCACGAACACGATCATTCCGAAGATTTGTCTGGAAAATTCTCCGGGTCAACATCTGCTTCCGGCAAATGCCATTGCTCAAAGAGAAGAAAAAATCGTACGAAGAAGACGATTCGAGTCCCAGCGATTAGTTCAAAAATCGCAGATATTCCACCGGACGAGTACTCGTGGAGGAAGTATGGTCAGAAGCCGATCAAAGGATCTCCATACCCACGGGGATATTACAAATGTAGCACGATGAGGGGATGTCCGGCGAGGAAACACGTGGAGAGAGATCCGAACGATCCGGCGATGTTGATTGTAACGTACGAAGGGGAGCACCGCCATACGCAGAGCTCGTTGCCGGAAAATATGGCGGCTGGTGTAGCTTTAGTTTTTGAGTCAACTTGA

>ClWRKY30

ATGGGTGACGATGATGAACAGCCGCCGCCGCCGCCGCCGCCGCCGTCCAAATCTTTGAGACCCACTATTAATCTGCCGCCACGGAGTTCTATGGAGTCACTCTTCGGCGGTGGGTCGGGTTTGGGATTTGGGTTTAGCCCAGGACCGATGACCCTCGTCTCGAGCTTCTTCTCTGATTCCGACGACTGCAAGTCTTTTTCTCAGCTCCTCGCCGGAGCTATGGCATCGCCGGTGGCTGCCGTTCCTCCGTCGGCGTCTGAGTTTAAGGCTTCTACCGGTCTGCTCGAATCTCCGGAGCTTTTCTCGCCTGGTCAAGGACCTTTCGGAATGACTCATCAGCAAGCTTTGGCTCAGGTTACTATGCAGGCCGCAGAAGCCTATTCTCATAATCAAATGCAAGCAGCATCTTTTTCTTCATCAGTAGCACCCTCTGCATCCTCCTCACAGCTTTTGACTTCCTTGCCGGTCGAGAAGACTAAAGATCAGCCGATGCAACCTCCGTTACAGGACTCTGCAGTTGCATCCAAGGAGCCATCAGATAACTCTCAATCAGAGCAGAGATTGCAATCTTCTTTGTGTAATGTTGATAGGCCTACTGATGATGGATACAATTGGAGAAAATATGGACAGAAACAAGTGAAGGGCAGTGAATTTCCTCGAAGCTACTACAAGTGCACGCACCCGAATTGTCCTGTCAAGAAAAAGGTTGAACGATCTCTGGAAGGACAAGTCACTGAAATTATCTACAAGGGCGAGCACAACCATAAACGACCTCAACTGAATAAGCGGGCAAAGGATGTCGGAAATTCTAACGGATATTCGAGTATTCATGGAAATCCTGAGCTATCTTCTCAAGTTCAGAGTGGATATTTGAATAAATTGAATGAGCAGACATCTGTGTCTTCCACTGCAAAGAAGGATCAAGAATTGAGTCATGTTACACACGAGCAGTTGTCGGGTACTAGTGATGGTGAGGGAGGGAGTGAGATAGAAACTGGAGTGAATCGAAAAGATGAAGACGAACCTGATGCCAAGAGACGGAACACTGAGGTTAGAATTTCTGAGCCAGCATCTTCTCATAGAACTCTCACAGAATCCAGAATCATTGTGCAGACTACAAGTGAAGTCGATCTATTAGATGATGGCTACAGGTGGCGTAAGTATGGGCAGAAAATTGTTAAAGGCAACCCTTACCCAAGGAGCTATTATAAATGCACAACTCCAGGATGCAATGTTCGTAAACATGTAGAGAGAGCTTCAACAGACCCGAAAGCTGTCATAACGACCTACGAAGGTAAACACAATCATGATGTTCCAGTTGGTAAAATCAGCAGCCACTGTTCAGTCAACAGTAACAATCCTCAACTAAAATCACAAAATATAGTTACTGAGAAAAAAATTTCAAGTAACAACACTGATCCTGGGAACTCTCGTCAACAGCCTGCTGGACTTTTACTGTTAAAGGAAGAACAAATAACATAG

>ClWRKY31

ATGGATCGTATGAAGGAAGAGAACAAAGTGTTGAGAAAAGCTGTGGAACAAACAATGAAAGATTATTATGATCTTGAAATGAAAATTGGTATCATTCAACAAAACAATAACCTCAACAATAAGGACTCTCACAACTTCCTATCATTCCATGAAAATGAGAACAAGAGGCATGAAGAACCAACAAAACAAGACCTCGAACTCGGAGAAATGGCGAAGAAGAAGAGACGAGTTCGGTCGCCATCGAAGGAGGACGAAATGAGGGAGAGTGAACTAGGGTTATCATTAGGGCTCCATACTAAGAACAATGATTTGGATCAAGAAGATAATCATAGGGAATTATTACAAGAAGAAAGAAGAGAAAAGAAGAAGGAAGATTCAATAATGTCCAACTTCTACTCAATCCAAAACAAGCCACAAAGACCTGAATTGCAAGCAATGGCACCCCCACAAAACAGAAAAGCTAGGGTTTCTGTGAGAGCAAGATGTGAATCTGCCACGATGAACGATGGTTGCCAATGGAGGAAATATGGTCAAAAAATTGCGAAGGGAAATCCATGCCCTCGAGCCTATTATCGTTGCACGGTTGCACCAGGATGCCCAGTTAGAAAACAGGTTCAAAGATGTTTAGAAGACATGTCAATTCTTATAACAACGTACGAAGGAACACATAATCATCCACTTCCTGTTGGAGCCACAGCCATGGCTTCCACAGCTTCTGCAGCAGCTTCCTTTATGCTCTTAGACTCCACTAATACTACTAATAATCTTTCTTCTCTTCCAAACCCTCAAAACCCTAATTTTCTAAACCCTCAAAACCCTAATATTCTAAACTCCTCTTCCTATTCTCCAACTAATTTCCTCCAAACTCAAAACCCTAATCAACCCTCAACCAATACATTTTACACTCCATTAATCCCCACATCTTCAACCTCAAACTTCCCCCAACCCTCCAATTGGATCAATACCAACCCTACTTCCTTTTACCATACCAACTTTCCCCTTCACGGCCGCTCCTTGAGACCACCCGACGATAATAAGCCACCGTTCACACCTGAGAGTGTGTCTGCTATTGCTTCTGACCCTAAGTTTCGAGTCGCGGTTGCGGCTGCCATTTCTTCGCTCATTAACAAAGAGAATAACAACACAACAACGTCAAATTCTCCTATCGAAGATTCTTCTTCTTTCGGTCCAAGTAAGGATGGGAAAGGCAGCAGTGGCGGCGGTGATAGTGATGGCAGAAACAAGAAATGGGTTGTTGAGTCCCTCTCATCAAATGGTAATTAA

>ClWRKY32

ATGGCTGTGGAGCTCATGGTGGGTTTTGGGGATGCTACTACTCCTACTAATTTCACGCCCAAGATGGAAGAAAATGCCGCTGTTTCTGCTGTTCAAGAAGCCGCTTCTGCTGGGATTCAAAGCGTCCAGAATTTCCTACGTTTGATGTCCCATACTCCTAATCACCAACACTCCCAAGACGATTCTTCTGCTTCCACTACTCCTAATGCTATCGCTGGATACGAAGCCGTCGCCGATTCCGTCGTCAGTAAATTCAAGAAGGTCATTTCTTTGCTCGACCGAAGCAGAACCGGCCACGCACGTTTCAGAAGGGCTCCGGTTCTTACTACTACACCGCCCCCTCCTCCTCCGCCCAAGGTCAAGCCGCAGCATCAAGATCCGAGTTCGTCGTCTCCGATTTCAGTCCCTCAGATTCAAGTAAAGAAACAAGAATCAGTTTCTGCTTTTAAGGTTTATTGCCCCACGCCCTCCCCTGTTGTGCGTTTGCCTCCTCTGCCTCACAACAACTCCCATCAGCCTTCACATCCACCCAATACCTTCCAAGCACAGCAAAATACTTCGTCGGTGGTACTCAAAAATGGGTCTGTAGATAGAAAAGATGCGACTACCACCATCAATTTTGCAGCCTCACCCCCGATTTCCGCTGCAAATTCGTATATTTCATCATTAACCGGAGACACCGAAAGCTTACAGCCATCTTTGTCATCTGGGTTCCAGTTCACCCACATGTCCCAGGTCTCTTCCGCCGGAAAGCCCCCTCTTTCATCTTCTTCGCTAAAGAGAAAATGCAATTCCATGGAGGATTCCGCCATGAAGTGTGGCTCGTCTTCCGGTCGGTGTCACTGTTCCAAGAAGAGGAAAAACAGGATAAAAAGAGTCATCCGAGTTCCCGCCGTTAGCTCAAAACTTGCCGATATCCCACCAGACGATTACTCCTGGAGAAAGTACGGGCAAAAACCCATCAAAGGATCTCCTCATCCAAGGGGATATTACAAATGTAGCAGTCTGAGAGGCTGCCCAGCACGGAAACACGTAGAACGTGCCTTAGACGATCCAACAATGCTGATTGTAACTTACGAAAACGATCACAATCACGCCCACTCCACCGAAACACCTGCAGCGCTTGTTCTCGAATCATCATAA

>ClWRKY33

ATGATGGATATTTTTTTGGATCTTAATGTTGATCCTACTTCTTCTTATGCTAATTCAGCCATGGATGAGGCTCTTCATTCTTCAGGAAGTGAGTCGTCAAGCTCGACATTGGAACAAGAGTTGGATAGAAAAATCCAAGAGAATGGGAAACTAAGTCAAATGCTAAGAATAATGTATGAGAAATACATTAATCTTCAAAAGCAAGTGATGTATCTGTTGAGCCACCAAAAGCAAAACTCAGAAATGGAAGGTGTTTGTTCAAGGAAGAGAAGGGCAGAAGGAGAAGAAGATAATTATGAGAATTTGGAAGGGATTTGCAGCACAAGAGATGAAGATTTCAACAGGTGGCTTAAGAGGCCAAGATTAAATGGAAACTCAAAAGTTTCTAAGGTTTTTGTGCAAAAAGATGCATCAGATCCAAGCTTGGTGGTGAAAGATGGATATCAATGGAGGAAGTATGGGCAAAAAGTCACAAGAGATAACCCTTCTCCAAGAGCTTACTTCAAGTGCTCCTCTGCACCAAATTGTCCTGTCAAAAAGAAGGTGCAAAGAAGTTTGGAAGATCCAACAATTTTGGTGGCCACTTATGAAGGAGAACACAGCCATGCCAGCCATTTTCAAACTGAACTTTCTTTAAGGTCCATCAATGGTGGCAAAGGCAGTGCAGTTCCCGTCTTAGCCACGATCAAACCGTCGTGTGCTACCGTGACCCTCGATTTGATTCACGAAGACGGGCTGTTTAAGAGTCCTAAAGATTACGCTTCGTCGGAGTCGGCCGAGGCAGCGGTTTGGCAGGAGTTTTTGGTACAACAAATGGCCTCTTCTTTGAAGAAGGATCCTGAATTTGCTGGCATTGTTGCAGGTGCCATTTCAGGCAAGGTTTTGGGAAACCAAACAAACAGAGAATAA

>ClWRKY34

ATGGAAACTGGATCCCAAAGATCATCAGGGCGAGGGCAGGTTGTTCCCAATATCAATTATCAAGTTCAAGTGTCGTTCCATCTCTCAAATGCACCGCATGATCCCCAGCCCATCCAGGAAATGGGATTCGTGCAATTGGAAGAGCAGAGCCAGGTTTTGAGCTTCTTGGCACCAAATAACGCCGCCATGCCACCTGCCTTTACTAACCCTACTACTTCTGCTCACACCTCACACCTCCTGCCTCGACCTTCTTGGAGTTCACAACAGCAGGTGGGCGGAACAGTGGATCCGAAGGCCGGGAACGAGGAAAACTGCACGGGTAGTGCTAGTGACGGCAGCAATTCATGGTGGAGAAACACAAACCCAGATAAGAGTAAGGTGAAGGTGAGGAGAAAGCTGAGAGAGCCGCGTTTCTGTTTCCAGACCAGAAGCGATGTAGATGTCCTCGACGATGGCTATAAATGGAGAAAATATGGCCAAAAAGTCGTCAAGAACAGCCTTCATCCTAGAAGCTACTATCGTTGCACGCATAGCAACTGCCGAGTGAAGAAGAGAGTGGAGAGACTGTCGGAGGATTGCCGGATGGTGATAACCACCTACGAAGGTAGACACAACCACTCTCCTTGCGACGACTCCAATTCTTCAGAGCACGAACCCTTTACATCCTTCTGA

>ClWRKY35

ATGGAAATTATGGAACACAAGAATTTGATCTCCCAGTTGACTCAGGGGAAGGAACTCGCCATTCAACTCAAAACCCATCTTCACCCTTCTTCTTCCCCTCAAGAAGCTTGCCTTTTCTTGACTGAAATGATTCAATCTTCTTTTGAGCGAGCACTTTTGCTTCTCAATTTCAACTCTTCTAATTCAAACAATATACTTTTTCCACATCAAATTGCTTTGCTGGAAGAAGGACAAGAAGTAGAAGTAGAAGAATCATCCACTAAGAACAAGAGGAAGATCGGTAAGAGCAGTGATGTGTTGAAGAAGAGAAAATTGCTGCCTAGATGGACTGAGGAAGTTAAGGTTTGCAATGGGACTGCTCCTGAAGGCCCTCTCAATGATGGTCATAGTTGGAGAAAATATGGCCAAAAAGATATTCATGGTGCTAATTTCCCAAGATGTTATTATAGATGCACTCATAGAAATGTACGAGGATGTTTGGCAACAAAACAAGTTCAAAGATCGGACAATGACCCAAACATTTTCGAGGTAACATACAGAGGAAGACACACATGTAGCCAATCCACTAATTTGGGATCGGTTGTTTCAATTTCAACTCAAAATCAAATCTCAGAAGAAACCAATCAAATTCAACAAAACCCAAATTCTGAAATTTTGTTCAATTTCGGAGAAAACAATTTCAATCTCAAAACAGAGGATTTCAGCAATGTTTTTCTGCCATGTTCCTTCGAGGGGCCAATGATGGACCCGGCTTTTATTGGCGGCGGCAGCTTGTCTGCAACGGCTGTGTCTCCGGCGGCCTCAGACACGGCCTGGGATTGGAGCGCTTACGACGGCGGAGACGAGGGAGGACTACAGAGGGTTCAGTCGTCGGGGGAATCGGACATGACGGAGATAATTTCGGCGACAACTTCAGGAACAAATTCGCCGATTTGTAATGGGCATTGGGATTTCTCGCTTGATAACATTGATTTCGACCACAATTTCCCCTTCGACTCTCTAGATTTCATCTCTTGA

>ClWRKY36

ATGGAAGTCGATTGGGATCTTCACGCCGTCGTCAGAGGCTACTCCGCCGTGCCCTCCGCCGCCACCATTATTCCCTCTTCTTCTTCTTCTTCTTCTTCTTCTTCTTCCAATAACTCTATTCCTTTTTCTTTTACCAGAGACCTAACCAACCAAACAAAAAGTCATTTCTTCACCCTTCAAGATCCATTTCAACCCCCCAATTGCAACTCCACTGAAGAATTGCATGAGCTTTTCAAACCCTTTTTCCCCAAATCACAACCCTCTCCTCCGCCGCCGCCTCCACCGCCTGCTCCGCCGCTTCTTTCTTCTCCGGCTGCTAAAATTCTGACCCATCACAAGCAACATCAGAGCACCCATCTCCCCAAACAGCTCCATTCAACCCCTGTTTCTGCTCCGAGATCCAAACGCCGGAAGAATCAGTTGAAGAAAGTTTGTCAAGTTCCCGCGGAGAGTCTGTCTTCGGATATTTGGGCTTGGCGAAAATATGGACAAAAACCCATTAAAGGATCTCCATATCCAAGGGGATATTACAGATGTAGCAGTTCGAAGGGTTGCATGGCCCGAAAACAAGTCGAGAGGAACAGATCCGACCCGGGAATGTTCATAGTCACGTACACGGCGGAGCACAACCACCCGGCGCCGACTCACCGGAACTCCCTCGCCGGGTCCACCCGTCAGAAACCCATAACAACGACGCCCACCGCTTCCGGATCTGAAAAACCCGACCCGAAACAGCCGGTTTGCTCGTCGGAAGAACAGAGTACGATCACAGAGAGCAAAGAAGAGAAGGAGGAATTATTAATGGCCGAAGATGAAGAAGACGACGATCTGGGGGTCTCCGATTTGATCGTCAACGACGATTTCTATGTGGGTTTTGAAGAGCTCGACAGCCCAATCACCGATGACTGCTTTTCCGATGAGTTTCCGACGAGTTTCGACCTTCCATGGTTGTTTAATGGAAACCCAGACAGCGAAATTGTAAAACTCCCCTGTTAA

>ClWRKY37

ATGGAAAAGAAATTTGAAGACTCACTGATGGGGTTTGATAATTCTGCTGGATTTTACTCTCCGGCGGTGTTTTCCGATGAGTTTCCGGCGTCGAATTTTGATTCTTTTTCCAGTATTTTCGACATGCCATGTGATGACCATAAAGCTTCTGCTGCTGCTTCTTGTTCTTCTAATTCCTTTGACTTATTTAGTAGTGGAATTGAAATCCATGATTTTTACAATATTAATAATAATGATGATAATAATAATAATATTCCTTCTTCTTCTTCTTCTAATTTCTTCAATTTGCTTTCTACGGCGGCTCCGCCTCTTTCCTCACCGGCCTCCACCGTGCCGGAGTCCTCCGAGGTTCTTAATGCTCCACCCACTCCTAATTCTTCCTCTGTTTCTTCCTCCTCCAATGAAGCTACAGCTGCCATTCAAGAAGTTGACAAGATCAACAACAACACTGAGAAGCCTTCTTCTTCTAAAGTGTTGAAGCCAAAAAAGAAGAACCAGAAGAAGCAAAGAGAACCCAGATTTGCTTTCATGACAAAGAGTGATATTGATCATCTCGACGATGGTTACCGATGGCGAAAGTACGGTCAAAAAGCGGTGAAGAACAGCCCTTATCCTAGAAGCTATTATCGTTGTACGACGGCAGGGTGCGATGTGAAGAAGAGAGTAGAACGATCGTCCGGCGACCATTCGATAGTTGTTACGACGTACGAAGGTCAACACACTCACCAAAGTCCGGTAATGCCGCGAGGAAGTATCAGAGTTTTACCAGAATCCACCAACAACAGTCTCATCGCGGATCACGACACCATCGCCACTGGACTTTTATTCCAACACAACACTACCCAACCCTTCATATGTAGCTCTCCACCGCCGCCATTTCTAACAATCAACTCGTCGGTGGCGGCGGGAAGTTCCCGTCCTGCTCCTCCTCCTACTTCCTTCCCGCCACCACCGTCTCAAGCTTCTTTGGTCCGAGACCATGGGCTTTTGCAGGACTTGGTGCCATTGCAAATGAGGAAGGAACCAAAAGATGAGCAAAATGGATGA

>ClWRKY38

ATGGGAAATTCTGGATTGTTATTCTCCGATGCGATTATTCCCAATAATAATAATATGTTCGATTTTCCATTCATCGACTCCACTGATTCTTCTTCTTCTTCTTCTTTTAAGCCTTCCACTTTCTTGGATTTGTTAGCCACCCAAGATTACACCCCTTCTTTATTCGATCTTTTCTCTCCTCCGCCGCCGCCTCCTCCTCCTCCTCCTCCACCACCCCCCTCCTCCGCCGTGCCTGAGTCATCCGAAGTCCTCAATACTCCTCCCACTCCTAACTCCTCCTCCGTCTCTTGCTCCTCCACCGAACGCCTTCTCGACGCCGATGATGCCGATCGGGATAAATCGCCTTTCAACAAACAGTTGAAAGCAAAAAAGAAAAATCAAAAAAGGGGAAGAGAACCGAGATTTGCGTTCATGACAAAGAGCGAAGTGGATCATCTAGACGACGGTTACAGATGGCGAAAATACGGTCAAAAAGCCGTTAAAAACAGCCCTTATCCAAGAAGTTATTACCGTTGCACCACCGCCGGCTGCGGCGTCAAAAAACGAGTCGAACGATCCTCGGACGACCCTTCCGTCGTCGTCACCACTTACGAAGGCCAACACACTCATCAAAGTCCAATCATGCCACGTGGAGCTACATCAGCCGGCGCCGCTGCCTTCGCCTCACCCCAACCGCCGCTTGTTTTCCCACAGCCGCAATTATTCCGAAACCACCATCAGCAATATACGTACGCTCCGGCGCCGCCTGTGGACGTTGTCACGTGCGGCCGAGTGTTTGATCCCGTGTTTCATAGTTTTGGTGGTGATGAACGTCGTCGTATTGATGGAATAACTTGTTCTAATTCTGATTCTTTCCAAGACCATGGCCTTCTACAAGATATGATCGTACCCTCCTCCAACTCCTTACAAATCCCAGAAGAAGATAAACAAAATTAA

>ClWRKY39

ATGGCGGAGCGGGAAAGCTTCGAAACTGACCAACTCGGAAGCTCCAAGGCGGCTACTGCCGGGCAAGAGGATGATGAAGAAGACATGGAAGATTCCGAGGATGAATCAGAGGTGGAATTGGAAGATGGAGGAGGAGGAGGAGGGAGTGAGTTGCAACCGACTGAGTTGAGAACCGGTTCGTGGGTTAGTGAAGCTGTGGTGAGGGGTTCACGTTCTGAAACCCTAACGGTGCCGTCTGCGAATCAATCCTCCGAGAACGGTCGGTCTGATGGTTTGCCTGTCAATTCTGCTGTGCAGTCACTCGAAGGAGCTGAATTGAAGCAAGCTCCATCTTCCCGCAATGAGCAAAAGGCTGTTGAAGCAACCCAGATAGATAAAGTTCAAGAACAAAACCAGCTTCAGGTGTCAACATTTAAGGGACCTGGTTCGGACCAATCACCAACTTCAGTTACCCAGTCTATCTCATCCTCCCAAAGTCCAAGTTTGTCTGAACATAAGCTGTCACCTAAGAAGGTCCAGAAAGAATGTAAGCCAGAGCCAAGCCAGAAAAATTCCTCCAACCATAAAACTGCATCATCTGTTCCGAATGTAAGGACACCTGCTTCTGATGGTTACAATTGGAGAAAATACGGTCAGAAGCAAGTGAAGAGTCCTAAGGGTTCACGTAGCTATTACAAGTGTACATATTCTGAATGCTTTGCTAAGAAGATCGAATGTTGTGATCACTCGGGCCATAGAACAGGGACTGTTTACAAGAGCGAACATAGCCACGATCCACCTAGGAAGATTAGTATCCCCAAGGAAAGTAAGCTCGTGCCATATGTTGAGCCTGTGGTTAAAAAAATCATTGCAGAACATTCCAGAAGAGTAATTAATGATTCAGATCCTCCCACGCCTTCAAAAGAACCTGTACGGGAAACAGCTATAGTCCTTGAAAGGAAACGACAGCACTCAAGTGACTCTGATGGAAATGATGAATTTAAAATCAAAGATGAGAATGGCGATGGACCTGAAACAAAACAAAAAGTTAAGAAAAGCAGTGCGGGAAATTCAGGTACTCCCTTAAAACCTGGAAAGAAACCCAAATTTGTGGTGCATGCAGCAGGTGATGTGGGAATCTCGGGTGACGGATACAGATGGCGCAAGTATGGTCAGAAAATGGTGAAAGGAAATCCTCATCCTAGGAACTACTACCGATGTACTTCTGCTGGGTGTCCGGTCCGTAAACATATCGAATCAGCAGTAGAAAATCCAAATGCAGTAATTATAACATACAAGGGAGTTCATGATCACGACATGCCTGTACCAAAGAAACGACATGGTCCACCGAGTGCTCCTCTTGTAGCTGCTGCAGCGCCAGCCTCCATGAGCAATACGCAACCGAAGAAAACCGACGTGGTTGAGAGCCAAATCTCTTCAACACAGTGGTCTGTGGATGCTGAAGGAGAGTTGACTGGTGAGGCCTTGGAGCTTGGGGGTGAGAAGGCAATGGAATCTGCTCGAACACTTCTGAGCATTGGATTTGAAATCAAGCCTTGCTGA

>ClWRKY40

ATGGCGGTGGATCTCGCAGCTTTTCCGGCCAAGATGGATGATCAAACCGCCATTGAAGAAGCCGCCTCCGCCGGTTTACAGAGCATGGAGCATCTAATCCTTCTCTTATCCAAACAACAACACTCCCAAACTCTTAATAACAATCTCAATTACTCTGGCCTCACCGATTTCACCGTCTCCAAATTCAAACGATTGATTTCCCTATTAAACCGTACCGGCCACGCTAGATTCCGCCGTGGACCCTCCGATTCCCCAAATCCCGTCCTCAATTCTCTCGATCCACCTCTCAAAACCCATTTCCCTAAACCTAATTTCTCCCCTGCTTCCAAATTCCCTGATTCTAAGGATTCCACTACCACCTCTTCTTTTGTTTCCACGATCACCGGTGACGGCAGCGTTTCTAACGGCAAGGTTGATCTCTCGGTCTTTGCGACGCCGCCGGTGTCCGGTGGTAAACCACCGTTGGCGTTGAAGAGGAAGTGCGATGATAGCTCTGGATTTGGTTGTAAGGTTCCGTCGAAGCTCTGTCACTGTGCGAAGCGAAGGAAATCTGGAGTGAAGAAGACAGTAAGAGTTCCGGCGATCAGTTCTAAGATCGCTGATATACCTTCCGATGAATACTCTTGGAGAAAGTACGGTCAAAAGCCCATCAAAGGCTCACCTTATCCTAGAGGGTATTATAGATGCAGCACCGTCAAAGGATGTCCGGCGAGGAAAAAGGTGGAAAGAGCTCGTGACGATCCGACGATGCTCCTCGTAACTTACGACGGAGATCACCGTCACCCGCACCCTACGGTGACCGATGCCAGCGTGGGTGTCGTTTCCCAGAAGAGTTGA

>ClWRKY41

ATGGAGGCTGAAAAAGCTTTGCCAACCATAGATGATAAAGTTCAATCAAGTGGAGGAGGAGATGAAGAAGCTGCTCCCAATCCTAAACAAGATGTTCTTCTCAAGGATGACCAACATCATGAAGAAAGAATCAAAAGGACGAGAGTTGAAATGAGAGAAGTAAGGGAAGAAAACGAGAGGCTAAAAAAGAGTTTGGATGAAATAATGAAGGATTACGAGACATTGAAAAGGAAGTTCCATGAGATGAATCATCAAATTACACAAACAAATGGAAGTACCATAAATAATATTGATCATGAAGTTGAAGAAGAAGTTGATGACTTGGTTTCACTCACATTAGGAAGATTCTCAACCCATCAAAACAAGAACACCACTTCTTCTTCCTCACTGACTAAATTAGATCATCAAAAAATCTTAGACCTCAAACAAGATTATATTATTCAAACCCCATCAATTATTGATCGCAATATTCAAAGTCCAACCCATAGTGAACCTAAAGAGGAAGAAGCCGGACAGACGTGGCCGCCAAGTAAAATGTCGAAGCCCAGCAGTTTGCCCCCGCCCCCCATCGGAGAAGATGAAGTTTCTCAACAAAATCCTCCAAAGAAAGCTAGGGTTTGTGTGAGAGCTCGGTGTGATACTCCAACGATGAACGATGGTTGTCAATGGAGGAAATATGGACAAAAGATTGCAAAAGGAAATCCTTGCCCTAGAGCTTATTATCGTTGCACTGGTGCACCCACATGTCCAGTAAGAAAGCAGGTGCAAAGGAGTGTTGATGATATTTCCATACTAATAACAACCTATGAAGGAACTCACAACCATCCTCTACCTGTTTCAGCCATGGCCATGGCTTCCACCACCTCGGCCGCCGCCTCGATGCTCCTCTCGGGCGCTTCCTCCTCCACCTCTCAACCCGGTCTTAACCCTTCATTCACCACCGCCACCGCTGCAGCGAATCTTCATGGAATGAACATGTATCTCTCTAACAACACCAATTCAAAGCAATTCTACTTGTCAAACTCTTCAATGCTATCTTCTTCTCTCAACCATCCCACAATCACTTTAGACCTAACTTCAAATCCTCCCTCAACTTCCTCCTCCTCCTCTCCTTTCCATAAGAATATTCCTTCAATTAATAATAGTTACCCCCAAAAATACCCCTTCACAAGCCTTGATTTTGGCTCTTCACAACCCAACTTCATGTCTTGGAACAATAATAATAATAGTGGTCATCAAGCTTATAGTAATATAAGCAAAAATAATGCAATAATAGGAATGCCGTCAGACTTTGCTAAACAACTCCCTCTCCATACTAACATTTATCAAGCCTATCTTCAACAATTTTCCAAATCTTCAACTCCGCCGCCCCCACCGCCTCTACCAGACACGATAGCGGCAGCAACTAAGGTAATTACGTCGGACCCGAGTTTCCAATCGGCATTGGCAGCTGCCTTGAGCACGATCATCGTTGGTGGCGGGAGTGGCGGCGAGACGGGTCCTCCAACTTCCAGCTTCGTTGGAGGAGCACAGGCAGCTAAGTCATTAACATGCTCCACCTCAAAGAGTCCTTCTTCATCTCCTGGGGATAGTAGAGAAAATGGGAAGTGA

>ClWRKY42

ATGGACCGCCGTGTTCGTACAAACCCATTTGTCTCCGAGCAAGAAGATCCTGAAGCCACGTCGGATGACGGCTCACCGGAATCACCCTCCGACGGCAATGACTCAAAATCCGCCTCCGCCGGCCCACCTCCCAAAAAGAGTAGAAGGGGAGTGCAGAAGAGAGTGGTGTCCGTACCGATCGGTGACGTGGAAGGATCTAAGAGCAAAGGGGAAGCATATCCACCGTCCGATCCATGGGCATGGAGGAAATACGGCCAAAAGCCAATCAAAGGCTCTCCTTATCCCAGGGGATATTATCGATGTAGTAGCTCCAAGGGCTGCCCAGCAAGAAAGCAAGTAGAAAGAAGCCGTGTGGACCCCACCAAGCTCGTTATTACCTACGCTTTCGACCACAATCACCAACTCCCGGCCACCAAATCTCACCACCACCACCACAATTCCTCCCCATCCTCCGCCGCGATCGCCGCCGCATCCACCGCAGCCGACTCTTCGTCCCCGGGGAGCACTACGACGTCGTCCTCCACTTCCTCCGGCGATAACACGAACGCCGCTCCATCGTCGCCGGCAGCGGCGGCGAAGTTCGAGGAGGCGGCTGCGGTTTTCGCAAGTCAGCCGGAACTGGAACTCGGCGGCGACTCGCTGATGATAAAACCATGCATCGGGGATTTCGGATGGTTGGCTGACGTGGCGTACGACAGAATCCTGGAGGGCCCGATTTGCGGGGGAGGCGACATATTCGACGACGCCGATGTAATGGTTTTGTCCACTACAGGTGATGACGAGGAGGAGTCGTTATTTGCGGATCTTGGGGAATTACCCGAAGGCTCTGTTGTTTTCGGCCGACGACGACCGGTCCAACCGGACGGACCAAACCGGACATGTGGCAGAGTTTTAGACTGCTAG

>ClWRKY43

ATGGAAGAGGTTGAAGAAGCTAACAAATCAGCCATAGAGAGCTGCCATGGAGTTTTGAATCTTTTAGTTCAACCTCCTCAAGACCAACTTCATTTCAGGAATCTAATGGTTGAAACTAAAGAAGCTGTTTTCAAGTTCAAGAAAGTTATTTCTCTCTTAAATTCTGGTTTTGGTCATGCCAGAGTTAGAAAATTCAACAAGATTCCTCTCCCTTTGCCTCAAAAATCCCTCTTAGATTCCCCAAATTACACTCCACATCCTCCAAACAAAAATCTTTACCCTTTTCACTCTGGTTTGAATGCTAGATCTTTCATTTCTTCATTGAGTATGGATGGCAGCGTCGCCGACGGAAGCTCGTTCCATTTGATCGGACCGTCGTCAACGACAACGACGTCGGCCGATAACAAGAGGAAGTTTTCTGCAAGAGGAGATGAAGGAAGCTTGAAATGTGGAAGTTCTGGCAAATGCCATTGCTCAAAGAAGAGGAAACATAGAGTGAAGAGATCAATCAAGGTACCTGCCATAAGTAACAAACTTGCAGATATCCCTTCTGATGATTATTCATGGAGAAAGTATGGGCAGAAGCCAATTAAGGGCTCTCCTCATCCCAGGGGTTACTACAAATGCAGCAGCATGAGAGGTTGTCCGGCGAGAAAGCATGTTGAGCGGTGCTTAGAAGACCCGTCGATGCTTATTGTAACGTATGAAGGAGAGCATAATCACCCGAAAATGTCGACGCAATCTGCACACACTTAG

>ClWRKY44

ATGGAGGAAGCCATTTCTTTGATTCTTCGGGGGTGTTCTTTGGCAAGGCAATTGGAATTTGATGTTTTAAATTTGGGAAGTAATAACATTAATGTTCCTCAACCTCATTTGATGGCTCGATCTTGTGATGAGATTTTGGGTGTTTTTTCCGCCGCCAAGGAGCGGTTGAGCAGCCATGAACAATGGCCGCCTTTCACGGCGGTGCAACGGGAGGTGGGGTTGGAGGAGTGGCTGAGATCTACTTGCTCCCAAGCTATGGAGTTGGCACAAATGCAGACAAGGTCGCCGGCACCACCATCTGCGGTGGTGGCAGTGCCAGTGCCGGCAGCCGTGGAGGATTCCGGGAAGCTTATAGCAATGGGTTTTTCTTCTTCTTCCTCTTCCTCGTCAACAAAAGCACGGAGACGAAAAGATGATACGGAGAAGAGGACGGTGAGGGTGGCGGCGCCGCGCATCGGAAACACAGAGTTGCCGCCCGACGACGGCTTCACATGGCGGAAATATGGACAGAAGGAGATTCTTGGCTCTAGGTTTCCCAGAGGCTATTTCAGATGCACCCACCAGAAGCTTTACCACTGCCCAGCCAAAAAGCACGTGCAACGTCTCGACGACGATCCCCACACGTTCGAAGTCACCTACCGCGGGGACCACACGTGCCACATGTCCGCCACCGCCCCCTCCGCTCCTCCGCCACCGCTGCCTGCCATCGCCGGCGGCCAACACATATCCCAGTTCCGCCCCCCCTCCACCGGCTGGCTTTCAGTGGAGGTCGTCGGTTCTGCAAGTGCCTCGGCGTCCGGCGGTCCGGCAACCGTACGATACGGAAAAGACGTGGCCGAGCAGTTTCCGGTGGTGGACCTGGCTGACGTCATGTTCAACTCCGGTACGGGCAGTAGTAATAGTATGGACTCTATCTTCGCCGCTTACGTGGCGGACGATCACCATCACAAGTGGGAGAAAGACGACAAGAAGAATTAG

>ClWRKY45

ATGGAGCCCGCGGATTTTTCTCACGCTGGTATCCCGGCGGAGACAAGGAGGAAGATCACGGCGAAGCTTCTCTCCGGCCGACAGTCAGCAGCTCGCCTTCAGAGTCTTCTCCAGTCGGCGGCGGCGGCGGACCACGACCCCCTAGCTCTTGCCACAAAGATCTTGACTTCTTTCAATGAATCCATCTCCATACTAGAGTCGGCCGCAGCGGAGTTGAGCTGTCCGGATCATTCCCTTTGCTCCGATCTGGATTCCGGCGACTCACGGGGGAGCACGGCGGTTAAGAATCATCAAGGACGTGCCAACAAAAGAAGAAGATTGATGAATACGAGAGTTGTTATGACGGCAACGACGGAAGACAAATATGGTTGGAGGAAATACGGCCAAAAGGTTATCCTCAACGCAACCTATCCAAGGAGCTATTTTAGGTGCACTCACAAGTACGATCAAGGCTGTAGAGCCACAAAGCACGTGCAAAGAATGGAGGGTATGGATTCAGAAATAATGTACAAGATCACCTACATCTGTGACCACACGTGTAGCACCGCTTCACAGATCATCGCTTCTGCCGTTGCCTCCGCTTCCGATTCTTACAACCTCATATCGTTCTCCAACTCTCACAACGGTCAATTGATCGAGGGTACTGGCCACAGTTTCTTTTGTCCAAATAATGACGATGCAATGAAGGTGAGAGAGACAACGACGACGAGTGGTTCGACAAATCATGAGGTTGATCTGTGGTCAGAGTTGAAGGATTTTGGGAGCTTGCAAACAACGACAATGACAAATCAATATTATTTCTCGACGGGAGACGATGATGCTGATTCTCTCATGTTTTGGAATGGTTGTTTACAATAA

>ClWRKY46

ATGGAAAGTGGGTGGAGCTGGGATCAAAAGTCACTCATTGGTGAGCTAATTCAGGGGCTGGAGCTTACCAAGCGATTGAGAGCAGAGTTGAGTTCAGCATCTGCAGAAGAAAGCAGAGGATCCTTAGTGCAAGGGATTTTATCTTCATATGAGAAAGCTCTTTTGATACTGAAATGGAATGGACCAATGAATCAGCCTCAGATGGTTGAAGCAACACCTGGTTTGCCAGGCTCTCCAATTTCTGTTAATGGAAGTCCTTCTAGTGATGACTCTGGTAGAGGTCTCAAGGACCCTCAGGACCCCAGAAAAGAATCAAAGAAGAGAAAGACACAGCCCAGATGGACAGAACAAGTGAAAGTGAACTCTGAGACAGGATTCGAAGGACCCCATGAGGATGGTTATAGCTGGAGAAAATATGGGCAAAAGGACATACTTGGTGCAACATATCCCAGAAGCTACTATAGATGCACTTTCCGCAATACTCAGAATTGTTGGGCAATAAAGCAAGTGCAGAGATCAGATGAGGACCCTTCTGTGTTTGAGATTACATACCGTGGAAAGCACACTTGTTCCCAAGGAAACTATTTAGCCCAATCATGTCATTCACCAGATAAGCAAGAAAAGAAGGAAAATGACCACGACCATGGCCACAATCACGATCATCATCAGCTGCAGCCTTCGCAAGAGAACCTATTCAGTAATCAAACCATTGAGAACATTGAAAAGCTCGAAAACAAGGCGTCTACCTTCTGCTTTGGCTCAAGCTCAACTTCTGTTGGATGTAAGGACATTGTAAATGGTGGCTTTTCACATTTAGCTATCGACACTCATGCTGCCTTGGGAAGCTTTACTCAGTCATTTATCTCCCCAACTACACCCGACTCAAACTACTTCACTCCATCCCCATGCCAAAGGAGCAACATAGGAGAGACTCATAATGTGCAACATCCAGAACCTGATGTCCATGAGATTTTCTCGGCCAACACTTCAGCTACCAATTCCCCTATCCTGGATTGGGATTTTCCATTTGATTCAGAGCAGATCAACCCAAATTTCCCATTTAATTCCCAAGGGTTTTTCTACTAA

>ClWRKY47

ATGGATGGTGACATAATGAATCCACAAGGTCATCTTATGGGAACTTCTTCATTGATTAATAATTGTGAAATTGATTGGGATGGGCTTTTTTCTGGATCATCTTGTTTGAGTAATATAGAGATGGAGAAGGGAATTTGTGGAAGTGAGGATCAGGTTATTGCATCAATGGGAGATTATGATAATGTTGTGAATATTGGAGGAATAAAAGAGGGTGATATTGGTGGAGGGAAGAATAATTGTAATAAAGGGAAAATGGTGATGGGTAAAAGAAGAAGTGCAATGGCTCCAAGGATTGCTTTTCAGACAAGGAGTGCTGAGGATGTTCTTGATGATGGCTATAGATGGAGGAAATATGGTCAGAAGGCAGTCAAACATAGCAATCATCCTAGGAGCTATTATCGGTGTACACATCACACATGCAACGTTAAGAAACAAATTCAAAGGCACGCCAAGGATCCGACCATAGTGGTGACAACATATGAAGGGATTCACAACCATCCATCTGAGAAATTAATGGAGACTCTAAGCCCTCTTCTTAAGCAATTGCAGTTCCTTTCTGGCATTTAG

>ClWRKY48

ATGTCTTACAATAGTAACCAGCAGCTCACTACGTCGGAGAGCGATCTCTCCGAGCAGCCGGGGTTGGAGTTTACGGACTGGATGTTTGATGGGTGGCTGAACGAAAACTCTTCATCTCTGGCCGACTCGGTGATGTACTCGGTTTATCAAGAGGGGGAGGTTGATGAATTTGTTGGGAACACCATTCAGCAAGGACAGCCTAGCAGCAGAGACAGTGGGAGAGAGAGAGAAGTTAGAGAAAGATTTGCATTCAAGACAAAATCAGAAATTGAGATTCTGGATGATGGTTTCAAGTGGAGGAAATATGGGAAGAAGATGGTGAAGAACAGCCCAAATCCAAGGAACTACTACAAATGCTCGGTCGAAGGCTGCCCGGTGAAGAAGAGAGTCGAAAGAGATCGAGAGGATCCGAAATACGTGATAACAACATACGAGGGTGTTCATACTCATGAAAGTTCTTGA

>ClWRKY49

ATGGAATCCTCAACTACCATCAAAACCTCTCTCGACCTTAATTTCAATCCTCCGCCGTACACCGCCGACGAATCTCCTCTCACTCACACTCCTTCTCCACTCAAAGAACAGGCTCCAGCCATTCTTGCGGAAAAATTGAATCGGATAAGTTCGGAGAATCAGAAACTGAATCAGATGCTAGGGCTGGTGGTTGAGAATTGCAATGTTCTGCAACATCAGGTAATCGATTTGATGATGAAATCCAGGAAACGAAAAGCTGCAGGCTGCGATAATTATTGCAATTTCAATCGGAGCGGATCCGATCAATATTGCGGTTGCTGTAGCGACGATAATGATTCGTGTTACAACAAAAGGCCTAGAGAAAACAGTAAACCCAAGGTTATGAGAGTTTTGGTTCCCACGCCCGTTTCCGATTCCACCTTGGTTGTGAAGGATGGATATCAATGGAGGAAATATGGTCAAAAGGTGACTAAAGACAATCCTTCCCCAAGAGCTTACTATAAATGCTCATTTGCCCCTAGCTGTCCGGTGAAGAGAAAGGTTCAAAGAAGTGTTGAAGATCCATCTTATTTAATAGCTACATATGAAGGAGAGCACAATCATGCAAAACCCAATTCAGGAATTGAGTATCAATTAATTGGACCAATTCATTTAAGTTCAAACCTTGATTCTGCTTGTGGTGTTTCATCATCACCTTCTTCCTCAGTCAAATCTCCATCATTAATGACTTCTATTGTCACTAACTCTTTGAAAGCTTCACTTCCACAACCTGAGACACCCTCAACTACTTCAGCTTCAGCTTCTTCAACTCAAAAGCTTCTAGTTCAACAAATGGCTACCCTTTTGACCAAAGATCCAAATTTCACTAGAGCCCTTGCCACTGCCATTACTGGAAATATGGTAGATAAAGAAATTTGGCGATGA

>ClWRKY50

ATGTCAAATGATGAAGGCAAAAATGTGTACCAGCAGTATGATCCATTCCAATACAACCAGTTGGATATGAACCGTTCGATCTTCCATCAACAAGCGGCGACGTTGGATCCTGCTTTGATGAGCTTCACTAACTTCTTCGATACCTCGTTGGATTACAACAGCCTGTCGAAGGCGTTCGACGTGTCGTGCTGTTCATCCGAAGTCATTTCTGCAGTGGACGACAGGTCGAAATCAAAGAAGGCTTCGACGACCACCCCAAATTCTTCAGTGTCGTCGTCGTCTAATGAAGCTGTAGTTGAAGAAGATTCAGTCAAGAGCAACAAAGAGGACATAAAAGGGTGCGAGAATAAAGATGAAGAAAAGTCTAAGAAACAGAACAGCATAACAAAAAAGAAAGAGAAACGGCAGAGGGAGCCCCGTTTTGCTTTCTTGACTAAGAGTGAGATTGATCACCTTGAAGATGGTTATAGATGGAGAAAATATGGTCAGAAAGCAGTTAAAAATAGTCCTTACCCAAGAAGCTACTATAGATGCACCAGCCAAAAATGTGTAGTGAAAAAACGAGTTGAAAGATCATATCAAGATCCATCTGTAGTGATTACTACATATGAAGGCCAACACAACCACCATTGTCCAGCTACACTCCGAGGCCATTCTGCAGGGATCATGTCGTCTCCATTCTATGCATCAGCATCAACATCGGTAACGGCAGCCTCGTCGGGGCCTACGCTTCCACAAGAACTCTTCTCACATTTGTTACCGACAAATAACTGCCAAACCGACCCGGCCTCGATGATGTACCAAAATCTAAGCCTTCAACAACACCTTCAAATGCCTGATCATTATGGTTTGTTGCAAGATTTGTTCACTCAAAAATAG

>ClWRKY51

ATGATGGAAGAGGTCGAAGAAGCTAATAGGGAAGCTGTTGAGAGTTGTCATAAAGTGTTGAATCTCTTGACTGTGGCTTCCTCGTCTCAAGATCAGTTGAAGCTTAGGAGTCGTTTAATGGCGGAAACTGGAGACGCTGTGTTTAAGTTTAGGAAAGTTTTGTGTCTTCTTGATTCTTCTGGGTTGGGTCATGCAAGGGTCAGGAAAAAGAAGGTCAAGAACTTTTTGTTTAATTCCTCTTCTTCCCCTTTTCCTCTTCCCCAATCTTTGTTCTTAGAAACTTCTTCCCCTAATTGCAGAATGGACCTTCTCCAGGGCAGGAATTTGCAGATGGGTCCTCTTTGTTTGGGGAACCCATCTTTGGAATTGAACTCTAATGCCAAAAGTTGTTCAACCCAACAAATACAATCTCAATCTGCTGCTCTTTATCATCATCATCATCATCATCACTTGCTTCAGAACAGGGTGTTGTTGAACAATAATCCTAATCCTAATCCTCCACAGCCTGAAATGATGTATCTTAGGAGCAATAATGGCATTAACTTAAATTTTGATAGCTCTAGTTGCACACAACACACAATGTCATCCACTAGGTCGTTTATATCTTCCTTGAGCATTGATGGGAGTGTGGCTAGCTTGGATGGGAGTGCCTTTCATTTGATCGGGGCACCCCGGTCTCTTCCGATCAGAATTCGCATTCTAAGAGGAAGTGCAGTGGGAGAGGGGAAGATGGGAGTGTTAAATGTGGAAGCAGTGGGAGATGTCACTGCTCAAAAAAGAGGTTGGGAAGCTGTGATTTTGAAACATAGAGTAAAGCGATCAATTAAGGTGCCCGCGATAAGTAACAAGCTTGCAGATATCCCTCCTGATGATTATTCATGGAGGAAATATGGCCAAAAGCCAATCAAGGGTTCACCTCACCCAAGAGGATACTATAAGTGCAGCAGCATGAGAGGCTGTCCAGCGAGGAAGCACGTCGAGCGGTGTCTCGAGGAACCTTCCATGCTCATTGTTACATATGAAGGTGAACACAACCATCCTAGAATACCTTCTCAACCTGCAAATACTTGA

>ClWRKY52

ATGGAGTTTCCCACTCTAATCCAAGAGCTTAACCAAGGCAAACAACTCGCTAACCAACTCCGTAACCATCTCCATCCTTCTTCTTCTTCTTCTTCTTCTTCTTCTTCTTCTTCTTCTTCCTCCTCCTCCTCCTCTCATGGCATTCTCTTAATCGATCAGATTCTTCGTTCCTACGAGAACGCGCTTTTGGCTCTTTCCGCCGGTGCCCCCTCCGTCACATCCCCCCTTGCTCCTCTGAATCACACAGACGTGGTGTCCAAGAAGAGAAAGGTAATGGCGAAATGGAGTGAGCAGGTTAAGGTTTCTTCTAGCTCTGCTAATGATTCAGGTCCTCGTTGCGATGGCTTCAGCTGGAGAAAGTATGGCCAGAAGGACATTCTTGGATCTAAATTCCCCAGAGGCTATTTTAGATGCTCACATCGCTTCACACAAGGATGTTTAGCAACAAAACAAGTTCAAAAATCAGACAATGATCCAACAGTTTATGATATAACCTACAAAGGAAGACACACTTGCAACAGAGTCCATTCAAACACACCACAAGAAGACCAAAACTCATTACTTCAACACCCAATGCCACCAAAACAAGAACAAAAGCCATTGCAGCAGCCACATGATCCTTCAGGGTTTACGTTCAGCTCCGACGCCGTACAGGTAAAATCCGAGAACTTAGACGATGTCGAGGGTGGCCTCTTTCAACCATTTTGTGCCCCCTCCCCAATGTTTGGATCTGAAGTTCAAGATGATCAAAGTCCTTTTAGGGAAAGTGAGTTTTCTCCGACGTTTGAATCGAATGATATGTTCGGATTCTGTAGTGATTTCGAAACCGGGTTCATCTCGATTCCTAATTCAGTGACTAATATTTCAATTGGAGACTTGGAGGAGTATTGCAGTTTTGATAACGTGGAGTTGTTCTGTTGA

>ClWRKY53

ATGGAGGAGGTGGCGGCAGCCAATTCGTTGCGATATCCGTTTGTCGATGATGACGAGAATAAGAGTTGTCTAGGGTTCATGGAGCTTCTCAGTGTGGATCAAAATTTTTCATCCCAATTTGATGTCTTTGAAACATTATCGTCTTTATCGTCTTCCCTGATTTCAAATCCTGTCGTAAACTCCGAGAATTCGGAGATTTGGAACCAATGGCCGACCACACCAACTTATTCGTCGTCGACTTCGAGCGAGATCCTCAATGGTGAGCCAAATCAAGAGGGAGGAGAGAAACAACATCAACAACACACTGTAAAAACTAACAAGCAGTTGAAAACAAAGAAGACGAGTCCAAAGAAGAAAGATCAAGAACAACGATTTGCATTCATGACAAGGAGTGAAGTTGATCATTTGGAAGATGGATATAGATGGAGAAAGTATGGTCAAAAAGCTGTGAAAAACAGCCCTTTTCCTAGGAGCTATTATCGTTGTACTAGTGTAGCATGCAATGTAAAAAAACGGGTGGAGAGATGTTTGAAAGATCCAAGCATTGTTATGACAACATATGAAGGCCAACACACTCACCCTAGTCCCATTATGGCACGATCAACTTTCTTTCCTCCGCCCATCTCAGTCACTCTCTACGATGATTATCCATATCAAAATGGTCATAGTTCAAATTTTATCAGCCACCCGAAGGGCTTCGTATCATCATCCTTTCATCAACCGTGTGGCATTGCTTCTTCAAATCGAGCTACTCATTTGCTTGGTGCCACCATTGACCATGGGCTACTTCAAGATATCACCCCTTTCCAGTACGATGAGTTGGCATGGAGAAATGGCCCGATCTAA

**The amino acid sequences of all identified WRKY families** **in three cucurbitaceae species melon, cucumber and watermelon**

>CmWRKY1

MYFVWRKRRRKKILGNNRDQNSMGNPGLLFSDAIIPNNHNNNLFDFPLIDSIDSSFKPSTFLDLLATQDYTPSLFDLFSPPPPPPQPPLPPPSSAVPESSEVLNTPPTPNSSSVSCSSTERAFDNDDVDRDKSPFNKQLKAKKNQKKNGREPRFAFMTKSEVDHLDDGYRWRKYGQKAVKNSPYPRSYYRCTTAGCGVKKRVERSSDDPSIVVTTYEGQHTHQSPIMPRGALSSTAYTASPQQHQQQPPLVFSSQPQQLYRNQFTYAPAPPVDVVTCGGGFGHVFHSFGEERRRIDDRTTPDSFQDHGLLQDMIVPFPEEEKKVN*

>CmWRKY2

MEAAAAFGRPRPVVKTEKPPVRDVSHDDDRDSPSKQQQQQQHLLVKRAGNNHAKQEHDTEDKTSCSSDKKDLSCIKLQEDQLESARAEMGEVREENQRLKQSLNQIMKDYEALKMQFLGIVGRDSKKLQDDDNDVNKEQQQQQQHDDDQIELVSLSLGRFPVSEKIKKVADEKSSMNIIIGGGDQDEEAACKEALSLGLNCKFEREESMVAVVKEVDSPNSFDHEATKEEAGETNWPSKGGKTMRSVEDDVTPQNPPKRARVCVRARCETATMNDGCQWRKYGQKIAKGNPCPRAYYRCTGSPTCPVRKQVQRCADDMSILITTYEGNHNHPLPASANAMASTTSAAASMLLSGSTTSATTAASSSTASNTLHGLNFYANNSKPNFYLPNNSSSIISSTSPTHPTITLDLTSNPSSSSSNSSTHFGKFTSNFPNSRYPFTGQLDFGSSRNNVLSWNNGLLSYNRNNHPTTTTTANNIYQNYIQQQQRNPTTSLQHQQPPLPDTIAAATKAITADPSFQSALAAALTSIIGTGGASASAGLTKSSSGRGEQSLFQLMTTTATTNKGNGCGTSFLNNITTTTTTTTSNSPPTGNMVFVPTNSKSASASPGDHIDLTH*

>CmWRKY3

MDDVSFNHNNHHQFYSSDPFEDSDELKPTPDSPAPSSTIAAAATKKGRRGMKKKIISVKINGDSPRNSSGSATPPSDSWAWRKYGQKPIKGSPYPRAYYRCSSSKGCPARKQVERNRLDPTTLVITYSCEHNHSGPVSRNNNNNNNQNNQIVLMKPGSPETVAVHQEPEVEEKFVEIGGEESLITADEFSWFGEMETTSSTVLESSIFSGRASTGLVDHSISSDVAMLFPMGDDDVDESLFADLGELPECSLVFRRGGGRGLPVDEQPAAAQRRITPWCGTTT*

>CmWRKY4

MDNNNYQQAGDLTDVIRPTSAPVAGHFSSEFSLDSFSGEGRLWSHLPADNSSMNFGDPLSFQTRDPFLLPHFSPFNFASDGGGEGLAADQPSNLLSHMLQISPSSGDGVISTPPCESLASAVGNSPSSTSVRGGGGVVVPTGGSNPLCLMENSGIQISSPRNSANKRRKSQVKKVVCIPAPAPANSRSSSGEVVPSDLWAWRKYGQKPIKGSPYPRGYYRCSSSKGCSARKQVERSRTNPNMLVITYTSEHNHPWPTQRNALAGSTRSHPSRTTTTTATNKTSPKQERINDILPTTMIKEEEIDEDQTTDKAATTTNDNQEEGDHQDFPFDLIFTEFADQMNDLEHENDHDHSHHLSADPLNNNMLMFGSHGFSTGNGEGSKDPFLELYDWAENSSGSLFKEAKGG*

>CmWRKY5

MDSLAAIPWSDSYTTVSSDDHLFTLTNLLHDDDNHDSSPLFLLPQDNADDNNRSIRLPVPGGATYFGPTIEDIENALSIGTPRSKDLHSHAHISHTGFSIVERGSLNKVEHKYSLRIKSCGGNMVADDGYKWRKYGQKSIKNSPNPRSYYRCSNPRCSAKKQVERSIEDPDTFIITYEGLHLHFAYPFFLMGQNPQPQSPTKKPKTIDPEPEAHEKPPFLDPIESTGTQGLLEDMVPWLIRNPSTHHNALSNSSSCLSHRSPPPTPPSPSTSPSFTTSCF*

>CmWRKY6

MAVDLMSFPKMDDQIAIQEAASQGLKSMEHLIRLLSHKQSSSHVDCSDLTDATVSKFKKVISLLNRTGHARFRRGPISSTSSSSSGSSAHLSQNQAMTLTPTPFTSPPNVPAPPFTAPATIAQPQTKVVAAAANFLPQPQSMTLDFTRPNILNSNPKGTDLEFSKETFSVSSSSSFMSSAITGDGSVSNGKLGTSIFLAPAPTASGGKPPLSVAPYKKRCHEHDHSEDLSGKFSGSTSISGKCHCSKRRKNRMKKTIRVPAISSKIADIPPDEYSWRKYGQKPIKGSPYPRGYYKCSTMRGCPARKHVERDPNDPAMLIVTYEGEHRHTQSSLPENMAAAAGVALVFEST*

>CmWRKY7

MAAGNDDWDLSAVVRSCNSAASATDPTSAAAESALSCLASLTFDDDPDDVAFSFSDIFQPKQPNGGFHELHQAFVSFLPNPSATDTTTTTTTTVTAVPVSEPEIPYPTPPNRHFRQGIKPIRPNPPPVALQQHPHHRQPPFSPDLPNSPMTQSLIPKSRKRQNQQKRRVCHVTADNLSTDMWAWRKYGQKPIKGSPYPRNYYRCSSSKGCGARKQVERSNVDPETFIITYTGDHSHPRPTHRNSLAGSSRNRSSSSSSRHPTPGDFDPSMTASALVPSSSSPAASPITPLNDYEGPTGEKDGEMFEDMPIDSDEEEDDEDILIPNLTVRDEIFVGFEEVGRGRGRSS*

>CmWRKY8

METGTQTQRSSGRGEDYEVQVSFHLSNDPHHIHEMGFVQLEEHSQVLSFLAPNNNNNNATNMPPPPFTTTSPHSNILPRPPSWTNHQLPGTLDPKPGNDENCTATATDATNSWWRNTNADKSKVKVRRKLREPRFCFQTRSDVDVLDDGYKWRKYGQKVVKNSLHPRSYYRCTHSNCRVKKRVERLSEDCRMVITTYEGRHNHSPCDDSNSSEHEPFTSF*

>CmWRKY9

MEEEHHQLQQPSPPPPPCSDPLATSLEIDWIAVLYGQEAIGDLPPASSTCESSERRRDEEKTNRRKNGGRRWRKAAGRRRFEFQTRSTEDILDDGYRWRKYGQKAVKHSLYPRSYYKCTYVTCNVKKQIQRLSKDRSIVVTTYEGIHNHPSHILMQTLTPLLKQIHTSFPLSKLFMNYN*

>CmWRKY10

MEVDWDLHAVVRGYSAAPSAATIVPSSSSFSNNSVPFSFGRDLTNNQTKNHFFSLQDPFQPSNCNSTQELHELFKPFFPKSQPSSSSPTPPPPPPPPAAPPLLSSPAPKILTHHKQSTHLPKQLHSTSVSAPRSKRRKNQLKKVCQVPAESLSSDIWAWRKYGQKPIKGSPYPRGYYRCSSSKGCMARKQVERNRSDPGMFIVTYTAEHNHPAPTHRNSLAGSTRQKPVTPTTTASGSEKLDPKQPVCSSEEQSTITESKEEKEELLMAEDEEDDDLGVSDLIVNDDFYVGFEELDSPITDDCFSDQFPANFDLPWLFNGNPDGEIVKLPC*

>CmWRKY11

MTKSDIDHLDDGYRWRKYGQKAVKNSPYPRSYYRCTTAGCGVKKRVERSSGDHTIVVTTYEGQHTHQSPIMPRGSLRVLPESTNNSLTVDHDTTTTGLLFQHNTSSQPFMYSSPPSPFLTINSSSVAATSTPHPPPPISFQPPSLPQASVRDHGLLQDLVPLQMRNEPKDEQDG*

>CmWRKY12

MENYDQEVGDLTDIVRGRSTTTTTRSSTTTTTTSSSSSNCKTEILADHHLQDSTSFYYSSSSQLLQLQDHQDHHHHHQYSFGDPFCSVVAPSVLDHHLHQHHHHQLVDNNTNINAFFNGISASAATTTAAAAHQDHEDHHEVMKSSPCNSNLFSRMLQISPSSNKFQTISSLTNNSPPPSNFLISNHSPTTTTTPLHPDHHLHHFLHHHDQNNNNNNNNTSAALHISSPRNPPGIKRRKSQARKVVCVPAPVAASSRPNGEVIPSDLWAWRKYGQKPIKGSPYPRGYYRCSSSKGCSARKQVERSRTDPNMLVITYTSEHNHPWPTQRNALAGSSRSSQHSSKNNTSTTTTTTSTTTQPNSSKLLHHKNKQEVQEEEEDQDHENNNNGTTTVLLSSTAAADEEASNKNNNNVKEEEMIENELMMSSTSEGGLIDEDHDFFADLEELETDPLTLLFNTTTTTQQQQHQQQPPHKLEQIIKGSAAAACLHDVVPFNHLFDWPPPPHQEQPPPSSPTNRGFY*

>CmWRKY13

MSDEMFKDLFYGGMDEYESIVRAFGITSDYSNNNNEISGTTAMNSSCSFSSSDAGGGEDDDSVKEKEKHISKDVVEDNGGENSKAAGSGKSKKKGEKREREARVAFMTKSEVDHLEDGYRWRKYGQKAVKNSAYPRSYYRCTTQKCGVKKRVERSYEDPSIVITTYEGQHNHPIPATLRGNLSAASGTFPPSMLTPMPVVGGVGFLPAELLSNASSNNQAVGGGATVYSHNSFDYTYNGRQPEYGLLQDIFPAPSSFFNRQP*

>CmWRKY14

MDKGWGLTLRDSDHQSIGFFSNKQPPPPPPPTLNSFQRMFQGLEFSAKLGHTDSTSDDNNRLAVEVDFFSAKKRLVDDLEADQDSKPTSTTSIIKDDKALTPPPPPTTSFNLVNTGLHLLTANTGSHQSTVDDGISSDGEDKRAKNELAQLQVELQRMNAENHKLRDMLSHVSNNYSSLQMHLLTLMQQQQQQQNHSSEPANQREIAGEKKSTEIKHEVGKVMVPRQFMDLGPSGNNNNMGESEELLCNSSSDERTRSGSPLNINNNNTETASKKRDHAEIMPPNSDHENSKRSIPREDSPESESQGWGPNHKTPRFNNSSNSKPIDQSTEATMRKARVSVRARSEAPMISDGCQWRKYGQKMAKGNPCPRAYYRCTMAVGCPVRKQVQRCAEDRTILITTYEGNHNHPLPPAAMAMASTTTAAATMLLSGSMSSADHNLMNPNLLARAILPCSSSMATISASAPFPTITLDLTHSPNPLQFQRPTAAPFHVPFPGGQPPSAAAQLPQVLGQALYNNQSKFSGLQLSHEMGANSSPLGHHQITQPATPAQPGGASFADTLSAATAAITADPNFTAALAAAISSIIGGTHPNNNSNTNTSNNTTTNNNGSSNNNSKISSFPGN*

>CmWRKY15

MQVREIRDERGLSFMRGRNAIGNYGGDDDKENDGKPRLRVSTMKMKRIKGRKKVREPRFSFKTMTDVDVLDDGYKWRKYGQKVVKNTLHPRSYYRCTEENCKVKKRVERLADDPRMVITTYEGRHAHSPSDHNLEDPIMGHLPSSHLTSFFC*

>CmWRKY16

MNSLQNPTFFFDHHQQLDQDSSSSFMDFLNFSGYPLPDFGLEAETTMFSLSEAGTGDGSRSMKATSIDNNTIDDGWFEGKGVKRKKERGNGCNHKVAFITKSELEILDDGYKWRKYGKKSVKNSPHPRNYYKCSSGGCGVKKRVERDRDDSSYVITTYEGVHNHESPFLMYSNGSKLCHPHPQPICPNSSSPDPYSSTTTL*

>CmWRKY17

MAVDLISHLSPLPNMEPNAVQEATSGLESVHKLIRLLSIPNPHSLPSSSQSPIDFPSDCRAAADAAVSKFKKVISLLGRSRLGHARFRRAPLPQQPHYVTPIQQIPPHPHPHLNSNNSNDESLNFSAHNSFISSLTGDADTKHPSSSSSPFLISNLSQVSSAGKPPLSSSSLKRKCSSDNLGSGKCAAASSSARCHCSKKRKLRVKRVVRVPAISLKMADIPPDDYSWRKYGQKPIKGSPHPRGYYKCSSVRGCPARKHVERAVDDPAMLVVTYEGEHNHTLSLPETSSLILESS*

>CmWRKY18

MDSSYINFLPTSSSSFFHNSLAMDDHDDELEEESSLKKIKSEVSGGKLKKKKTRKRRFAFETRSQVDVLDDGYRWRKYGQKAVKNNKFPRSYYKCSNEGCKVKKQIQRLTKDEEVVLTTYEGVHSHPIEKPHDSFQNILTHMHIYSS*

>CmWRKY19

MDCSWPDTSPFDRRKAADELLRGRELAQQLRAYLQRSSTPASQDLLTRILSSFSKTLSILNHRCDSDDINGSIVDSPEDHGSRKSEESGDSCKSSTPNNDRRGCYKRRKSCQSWARETCSLVDDGHAWRKYGQKTILNAKYPRNYFRCTHKYDQACQATKQVQRLQDHPPKFRTTYYGNHTCSNFLKASDIVLGSSNFDDSCGVLLSFDTTAAPNFFLPHHPTLVKKEEVVTPEAGSGRDDEAVCSPSDYMSTADDHLSEVFMGSVVDFEDDVLPFHFDPINFNNTPSDPLDLPS*

>CmWRKY20

MNSINQTINTLAGGSSDNRTNNFAMEVPKFKSLQPPPFPMSPSSYLSSFSSGLSPTEILNSPLLFSFGVFPSPTTGALNLRNDCEEVDQQEMKGDVKNYSVSAYNPQTGSSLSSYFQSSSSNVTLLNPSGLSCDESGAKSEFVNTEMAAAESKQNSQLAIYNREQQKSENDGYNWRKYGQKQVKGSENPRSYYKCTFPSCPTKKKVERSLDGQITEIVYKGTHNHAKPQPTRRSGNSGVYDPSAAESGVLQEDCSVSVGEEEIEPNSPFSNSIEENEKEPEAKRWKGENENEGYCGGGSRTVKEPRIVVQTTSEIDILPDGYRWRKYGQKVVKGNPNPRSYYKCTSLGCPVRKHIERAANDMRAVITTYEGKHNHEVPAARGSGGGGYNTINRPIPTNIPMALRPLSVVTSDSFPANFPAAFRPGNLGMSEIGTQASSFPFQTSQGGPPSFQVSGFGSAAKEEVRDDTYFINSFLS*

>CmWRKY21

MDNKAAERVVIARPVASRPTCSSFKSFSDILTCAFDTSPPNTSSETKIAAIRPKTVRFKVKDNPGPSSGGKISETVPRTNSHGSSDTLAVSDSKTTVIFKPLAKHGNTNLQNCLPLPPVEVCIQCPNQDDVNFQSALTSNLCIQCPNQDSDNFQSALTSNLPQDITSTVENSQSIRSSRVALSYSKKDPTSSLRPQISGAQPSYDGYNWRKYGQKQVKGSEYPRSYYKCTHPSCPVKKKVERSLDGKVAEIVYKGEHSHPKPQPLKQNSSGTQREGSISNGTTRDTNPELWLNYLNGRIEGCESRIENHIEKACQGRGVIPFDPFSNREVNAGCGISDNSCGLSVECEEGRKGLESMDDKLRNKKRGGKNPTNEGETSIEGVNEHHAMNRGSTGIEISGKGIRWRKYGQKVVKGNLYPRSYYRCTGLKCKARKYVERASEDPDSFITTYEGKHNHGISLETSISVAPEME*

>CmWRKY22

MESCLSSITTNQPCIHEESTQGDDIDTQHPLEDEQKGSYIPMGMLRTSEDGYNWRKYGQKQVKGSEYPRSYYKCTHPNCLVKKKVERSLDGQITEIIYKGAHNHAKPDPTRRAMVGSVPISGDTPEIGEGGGNHFKLEAGLTWRNTQYGVKDIKPISNCSVDGLERTSSVSVLTELSDPLLNPQEKTVGVLEPVGTPELSSTLASHDDDSGGGGDDDLTTQGSISVCTEADDAEPELKRRRKEESSIETNLASRSVREPRVVVQIETEVDILEDGYRWRKYGQKVVKGNPNPRSYYKCTSAGCLVRKHVERASHDLKCVITTYEGKHNHEVPAARNSSQVNLSNGNAQPPASHVQPNMGLSRNSNVPKSETEIQDLATHFYPKPEFNHDYQRSGCFDTFTNDMKLGAPPFCQMKFPPLRNTLPYSSFGLSSKHTATGISGSLASVVSDLPISLPLNQKLSAAGYDYTNGRPILPFQVFLAGQQLRETDRFLTPKQEHDDDNICASFQPVVDSSSGSSSSSISSVYQQIMGNFT*

>CmWRKY23

MTEPESFGTDQLGSSKAAIEGQEDDEEEMEDSDDEPELEGEGGGRVSELKPTELRTGPSVCEAVVMGSLSETLTVAFVNQSSENGRSDGLPVNSSAQSVEGAELKQAPSSHSEPLAVESTQTDKVQEQNHLQLTVFKGPDSEQSPTSVTQSISSSASPNLSEHKLSPKVQKVCKPEPNQKNFFNHKTPSSVPNARTPASDGYNWRKYGQKQVKSPKGSRSYYKCTYSECFAKKIECCDDSGQTTEIVYKSQHSHDPPRKISIPKESKLVPYVEPVVKKIIAEHSRRVINDSDPPTSSKEPLRETAIVVFERKRQYSNDSNGNDEFKIKDENDYETEAKLKVKKGSAGNSGTSLKPGKKPKFVVHAAGDVGISGDGYRWRKYGQKMVKGNPHPRNYYRCTSAGCPVRKHIESAVENPNAVIITYKGVHDHDTPVPKKRHGPPSALLVAAAAPASMSSNTQPKKTDVVESQISSTQWSVDAEGELTGEALELGGEKAMESARTLLSIGFEIKPC*

>CmWRKY24

MEPTTTTINTSLDLNLNPPPYTDDQSHPPTPNSPLKQQAPTTGILAEKLNRISSENKKLNQMLGVVVENYSVLKNQVIDLLMKSRKRKAAPGCDNCCNFNRSASDQYCGCCSDDNDSCYNNKRPRENNSKPKVMRVLVPTPVSDSTLIVKDGYQWRKYGQKVTKDNPSPRAYYKCSFAPTCPVKRKVQRSVEDPCYLVATYEGQHNHPKPNSGIEYQLVGPINLGSNTKLDSSNNVSSSPSSSIKSPSSSSSLIPSISLDYLTKSQPQIPSPSSSNSSSSTQKLLVQQMATLLTRDPNFTRALATAITGNMVDNEIWR*

>CmWRKY25

MADSPPPPPPPPFSTADSSSTSSNALFFFPPDSDSTILTQFGWNFHSLQPQPSRFHDSHPIHSDFPATSTTTTTTTTTTTTITTTPPLIEDSAGLSDAPLPLPSNPSLSSSSSGDPPDKPPEIITPRKVKKKGQKRIRQPRFAFMTKSEVDHLEDGYRWRKYGQKAVKNSPFPRSYYRCTNSKCTVKKRVERSCEDPSVVITTYEGQHCHHTVGFPRGGLTIAHETSFGSQFSPQIPHFFYPDPSPPPPPPPTTTNNHNPPTPPIDQHFPSTSSSTEQQEPPNSHLQQLPSNEGLLGAIVPPAMMRRTT*

>CmWRKY26

MGEDDEQPPPPPPPSKSKPFALRPTINLPPRTSMESLFSGGPGLGFGFSPGPMTLVSSFFSDSDDCKSFSQLLAGAMASPVAAVPPSTSEFKSSPGLLDSPGLFSPGQGPFGMTHQQALAQVTMQAVEAYSHNQMQAASFSSSVAPSASSLQLLTSLPGEKTKDQLMQLPFHNSSVASKEPSDNSQSEQRLQLSSCNVDKPADDGYNWRKYGQKQVKGSEFPRSYYKCTHPNCPVKKKVERSLEGQVTEIIYKGEHNHKRPQPNKRSKDVGNSNGYSIVHGNLELSSQVRSGYLNKLDEETSISSIRKKDQESSRVTNDQFSGNSDGEGGSEIETGVNRKDEDEPDAKRRNTEVRNSEPASSHRTLTESRIIVQTTSEVDLLDDGYRWRKYGQKIVKGNPYPRSYYKCTTPGCNVRKHVERASTDPKAVITTYEGKHNHDVPLGKTSSHSSVSGNISQLKSQNIVTEKKNSSNNTDRGNSHQQPTGLLRLKEEQIT*

>CmWRKY27

MSNDEGKNVYQQYDPFQYNQLDMNRSIFHQQAAAAALDPGLMSFTNFFDTSSLDYNSLSKAFDVSCCSSQVISAVDDMSKKKASTTTPNSSVSSSSNEAVVEEDSVKSNKLEDIKGRCENKDEEKSKKQNSNLSKKKEKRPREPRFAFLTKSEIDHLEDGYRWRKYGQKAVKNSPYPRSYYRCTSQKCVVKKRVERSYQDPSVVITTYEGQHNHHCPATLRGHSAGIMSSPFYASASTSVTAASSGPTLPQELFSHLLPTNNCQNDPASMMYQNLSLQQHLQMPDHYGLLQDLFTQK*

>CmWRKY28

MILFYILFPLGFVVRARNRVVMGRTDDNVAIIGDWVPPSPSPRTFFSAMQMLGEDIGSSKPSMDTTTSSDHKTEELFLRPREQTVSENAIARGGIPGVNSGDRGMEFGTFSEQKFRGGLVERIAARAGFNAPRLNTESIRSTDHSLNSEVKSPYLTIPPGLSPTTLLDSPVFLSNSLAQQSPTTGKFPFLPNVSSTRSSTMMPEANNKGNNNPFHDNNTSFAFRPSVESGSSFFLNAASKTASATVLPQSCPRIELPVPRSENSFQSHLAEPSLSLPQNRIGHHPQVGLSTTYVEKDDGSKAVSEEQRPFDSLGGGSGEHSSPLDEQLDEGEQRGSGDSMAGGACGAPSEDGYNWRKYGQKQVKGSEYPRSYYKCTHPNCQVKKKVERSHEGHITEIIYKGAHNHPKPSPTRRGAIGSSDSHMNMQLDIPAQAGQQSADVPLWEDSQKGVPSGAPDWMHENLEVTSSASLGPEYGNQPNTLQAQNGSHIETVEAIDASSTFSNDEDEDDRGTHGSITLGYEGEGDESESKKRKLDAYVTEMSGATRAIREPRVVVQTTSEVDILDDGYRWRKYGQKVVKGNPNPRSYYKCTNPGCTVRKHVERASHDLKSVITTYEGKHNHDVPAARNSSHISSGTSSPVTGQNSTAAIQTHAHRPGPPQPQNTIPRFERPAFGFAGRQQMGTAHGFAFGMNQPGLGNLTMAAVGQPKLPVLPMHPYLGQAHHVNEMGFLLPKGEPNVEPTSDLGLNFSNGSTVYQQIMSRLPLGPEM*

>CmWRKY29

MDPTDSDLPDPSNASSGAKYKLLSPAKLPISRSPCITIPPGLSPTSFLDSPVLLTNLKVEPSPTTGSFTKLPMAHDSSSSAIYPMTSMAFSNTNASDEGRSNYFEFKPYVGPNMVPADLSHRKGEQSSEVQGQPQPFTAPPMTKFEISVMSNDLSRSTQMDTHTVTSGASVPEADGDDINHSLNTNSRVQAPQSDPKGSGIPVVSDRLSDDGYNWRKYGQKHVKGSEFPRSYYKCTHPNCEVKKLFERSHDGQITDIIYKGTHDHPKPQPSRRYSASASMNVQEDGTDKPSSLSGQDDRSCSMYAQSMHTIEPNGTTDPSLPANDSITEGAGTTLSCKNHDEVDDDDIYLKRRKMELGGFDVCPMVKPIREPRVVVQTLSEVDILDDGYRWRKYGQKVVRGNPNPRSYYKCTNVGCPVRKHVERASHDPKAVITTYEGKHNHDVPTAKTSSHDVTGPSTIPSSRYRLEESDTISLDLGVGIGTGGENRPNEYRQALHSQLVENRAPNGNFNFEVVQENSAPTYFGVLNRGVIN*

>CmWRKY30

MEEVEEATKSAIESCHGVLNLLLQPPPSPPHQHHFKNLMVETKEAVFKFKKVISLLNSDFSHPRFRNFNKIPLPLPQNSLLDSPNYTLHPPNKNLFNFPPGSNSKVSIFLGNPDLELSQNDKNTLHIPKQSPSLNFSFPHHHHHQQQQQQQSVLAHQKQMKQQAEMTFLRNNNGMNLNFDTSNCTLTMSSARSFISSLSMDGSVIGDRSSFHLIGPSTTTTTTSGNSKRKFSARGEEGSLKCGSTSKCHCSKKRKHRVKRSIKVPAISNKLADIPSDDYSWRKYGQKPIKGSPHPRGYYKCSSIRGCPARKHVERCLEDPSMLIVTYEGEHSHPKMLTQSAHT*

>CmWRKY31

MENYQMFFPCSDGGGGLSAYHHADMSSGGASDMFGNFQGGDMEAVSGFLGMKREVDGATVEAEGGGRKKGEKKVRKPRYAFQTRSQVDILDDGYRWRKYGQKAVKNNKFPRSYYRCTHQGCNVKKQVQRLTRDEGVVVTTYEGMHTHSIDKPTDNFEQILSRMQIYSTPF*

>CmWRKY32

MDLLQCRNLHMGPLCLGNPSLELNTNAKSCSIQQIQSQSAALYHHHLLQNRVVLNNNPNPPQHEMVYLRSNNGVNLNFDSSSCTQHTMSSTRSFISSLSIDGSVANLDGSAFHLIGAPRSSDQNSYHKRKCNGRGEDGSVKCGSNGRCHCSKKRKHRVKRSIKVPAISNKLADIPPDDYSWRKYGQKPIKGSPHPRGYYKCSSMRGCPARKHVERCLEEPSMLIVTYEGEHNHPRIPSQPANT*

>CmWRKY33

MEEVVAADSLRYPFLDGNESKSCLGFMELLEVDRDFSSQFDVFETSSPSLSSSLISNPENLEIWNQWPTTPNYSSSISSTSSEIVNGELTEPNLEGGEEKQDRQPTVKTDKQLKTKKRSPKKKGVEPRFAFMTKSEVDHLEDGYRWRKYGQKAVKNSPHPRSYYRCTSVACQVKKRVERCFQDPSIVVTTYEGQHTHPSPIMARPTFFPPPISVTLYDDYLIQNSHNSNVMSHSIAWCHH*

>CmWRKY34

MESGWSWDQKSLIGELIQGMELTKQLRTELSSASGEESRGSLVQGILSSYEKALLILKWNGPTNQLQMVEATPGLPGSPISTQPRWTEQVKVNSETGFEGPHEDGYSWRKYGQKDILGATYPRSYYRCTFRNTQNCWAVKQVQRSDEDPSVFEITYRGKHTCSQGNYLAQTCHSPDKQEQKETDPDPHELQPLQENLFGNQTIQNIEKLENKASTFCFGSSSTSVGCKDIVNAGFSNLAIDTHSALGSFTRSFTSPTSPDKNYFTPSPCQRSNVGGTHSVQNLDPDVHEIFSANTSATNSPILDWDFPFDSEQINPNFPFNSQGFFY*

>CmWRKY35

MGDSNYDNNNNVVIGGVKEGDIVISGGNNHNNNNNIKYKGRMVMGKRRSAMASPRIAFQTRSVEDVLDDGYRWRKYGQKAVKHSNHPRSYYRCTHHTCNVKKQIQRHSKDPTIVVTTYEGIHNHPSEKLMETLTPLLKQLQFLSGI*

>CmWRKY36

MEDWGLQAIVKGCNGIPIGSSTTAATTRLMEDTNNLYSFLRSDQEEDGGFFSSCVYNNYYNPQISSSSIFHDEFEGLFGRNSSNNSAAASISHLLRDFKEPADQKLHHKNQIIQPTKQKQSKKSRQNRVVKEVKADKVCSDSWGWRKYGQKPIKGSPYPRSYYRCSSSKGCSARKQVERSLSDPEVFVVTYTAEHNHAEPTRRNALAGTTRKKFPALENPNLDMILSPNNSTSVASIEEDQHHPMEGVADGEVLMDMPFEFFTGLEDLLFG*

>CmWRKY37

MLLLNNNMAVELMVGFGDATTSNNFTPKMEENAAVSAVQEAASAGIQSVQNFLRLMSHTTNQQHSQDDSSTSTTPNNGYEAVADSVVNKFKKVISLLDRNRTGHARFRRAPVLSTTTTTTPPPPPPPKVKLQHQDPSSSSPISVPPIQVKKQESVSAFKVYCPTPSSVVRLPPLPHNNPHQPSHPPNTFQAQQNTSSVVLKNGSVDRKDATTTINFAPSPPISAANSYISSLTGDTESLQPSLSSGFQFTHMSQVSSAGKPPLSSSSLKRKCNSMEDSAMKCGSSSGRCHCSKKSRKNRIKRVIRVPAVSSKLADIPPDDYSWRKYGQKPIKGSPHPRGYYKCSSLRGCPARKHVERALDDPTMLIVTYENDHNHAHSTETPAPLVLESS*

>CmWRKY38

MMDIFLDLNVDPNSSYANSTMDEAHHSSQKRDQFDGEIYGVQEKLSLSLSNKGSDSSPTLEQELDRKIQENGKLSQMLRIMYEKYINLQKQVMYLLSNQKQNTEMEGVCSRKRKAEGDQEDYENLEGICSTRDEDFNRWLKRPRLNGNSKVSKVFVQKDASDPSLVVKDGYQWRKYGQKVTRDNPSPRAYFKCSSAPNCPVKKKVQRSLEDPTILVATYEGEHSHASHFQTELSLRSINGGKGSAVPVLATIKPSCATVTLDLIHEDGLFKSPKDYASSESAEAAVWQEFLVQQMASSLKKDPEFAGIVAGAISGKVLGNQTNRE*

>CmWRKY39

MEAQQALATIDDDRVQSTGEGNDEASPNSKQRIHLKVVTSMGDLEKPFMETLSVPSASNSTWKEEDDEQHHQHHEERMKRTKVEMREVKEENERLKKYLDEIMKDYETLKRKFHEIKYNHDHDQIREDHQGKRSTQTSGSTINNINNNDDDDHHHQVEAEVDDMVSLTLGSSRFSTHHQNKNTSSSSSSFSLTNKILDLKQDYVIQTPSIIDHNIHSPTHSEPKDQEEAGQTTWPPSKMSKPGGLPSPAIGEDEVSPQNPPKKARVCVRARCDTPTMNDGCQWRKYGQKIAKGNPCPRAYYRCTGAPTCPVRKQVQRSVDDISILITTYEGTHNHPLPVSAMAMASTTSAAASMLLSGPSSSSSTSSQPGLNHSCTAAATAVNLHGMNMYLSNNTSSKQFYLPNSSMLSSSLNHPTITLDLTSNPPSTSSSSPFHKIPLVNNNNNYNNYPLQNIPSQILILPLHNPISYFANKQLPLHTNIYQAYLQQISKSSMTPPQPALPSDTIAAATKAITSDPSFQSALAAALSSIIGGGETGPSVSSLVVGGGGGGQGSMGFEAAAKSLTCSTSKSTPSSSPGDSRDNGK*

>CmWRKY40

MAVDLAAFPTIFDDQTAIEEAATAGLQSMNHLIHLLSKQQQQQQQYHSESPNNIDLNSSLLTDFTVSKFKRLISLLNRTGHARFRRGPSDSPNPVLNSLDPPQKTHFSKLNFSPVSKIPESRDSTTTSSFVSTVTGDGSVSNGKLDLSVYTTPPANGGKPPLAMKRKCNDVSGFGCKVPNSKLCHCAKRRKSGMKKTVKVPAISSKIADIPSDEYSWRKYGQKPIKGSPYPRGYYRCSTVKGCPARKKVERARDDPSMLLVTYEGDHRHPHPMVADASVGVVS*

>CmWRKY41

MMGERGGGDCDKKKKMRSRRFAFQTRSQVDILDDGYRWRKYGQKAVKNNKFPRSYYRCTHQGCKVKKQVQRLTRDEGVVVTTYEGIHSHPIEKSTDNFEHILSQMQIYTSY*

>CmWRKY42

MEVSSHHSFKPNPNDEHHLLSTQPTPEEEQPLSKKRKVVQKTVVTVKIGSKKAAIGIGKMKNEGPPPDFWSWRKYGQKPIKGSPYPRGYYRCSTTKGCSAKKQVERCKTDGSMFIITYTSSHNHPGPNISTLNLDQNQQEIDQPQPLDQDDDEDDDLVPNQEQDHNNNNNDHEKNSIIVSQCEEEVEVEVEEGEEDDDDELLLLEDEEKKGMEKIKDECLDQEPIISSSSSSSCCDEVMIIKTKKSEIENHDNFFDELEELPIPPPFSSTLMRSSYSFDEIRISAAPS*

>CmWRKY43

MDRRVRTNPFLSEQEDPEPTSDDGLPESPSDANDSKPTAAPPPKKSRRGVQKRVVSVPIADVEGSRSKGEAYPPSDSWAWRKYGQKPIKGSPYPRGYYRCSSSKGCPARKQVERSRVDPTKLVITYAFDHNHQLPVTKSHHHHHNSSPSSAVVAAASAGTDSPSPGSTTTSSSTSSGDNTNAAPSSPAAKFEEVAAVFASQPELELGGDSLMIKPCIGDFGWLGEVAYDRILEGPICGGGDIFDDADVMVLSTRGDDEEESLFADLGELPEGSVVFGRRRTVQPDGPNRTCGTVLDC*

>CmWRKY44

MAPPQNRKARVSVRARCESATMNDGCQWRKYGQKIAKGNPCPRAYYRCTVAPGCPVRKQVQRCLEDMSILITTYEGTHNHPLPVGATAMASTASAASASFMLLDSSNNNNTNLSNSLHQNPNILNSSSPSFLQTQNPNNHLFTPLFPTSSTSHFPHSFYHSNFQPNHLVSPLDRRTWKPVDDNKPPPLTPDAVSAIASDPKFRVAVAAAISSLINKENEHVTTTGETATDGKGGGGSDSDSGSKKWVVESLSSKSNGN*

>CmWRKY45

MSNEGHDHEKEEDDPYYPQFDPFTHNFHHQKPFEVPPPLTNPYSEAFDIAPYNVGFYSDFSHASSQYDYHNIFSTPFEMSCSSSEVISSVDDALKKSSSLGRDLSSVVTGEHPLTPNCSSTTCSSDEVVAGGGDSSKSGEVKGFDDKKGENSKKVDEGKKKEKREKGPRFAFLTKTEIDNLEDGYRWRKYGQKAVKNSPFPRSYYKCTSQNCSVKKRVERSSEDPGFVITTYEGKHNHYCPITLRGHNPTGVLPPSVTPPLFPPSPNFFSAESFCENLHQQYQYGLFQDFINNPSFNNSNQHPSN*

>CmWRKY46

MEAPEFSHGRILPAELRRKITAKLLPGQDSAAHLQTLLQSAAATEQDKRALATKILTSITEAISILESAAGEELSCPDNSLCSDLDSDDSRRSTGVKNNPSRANKRRRSMNTRFVRTSRTTEDEYGWRKYGQKVIHNTTYPRSYYRCTHKYDQGCQATKQVQRMEGSDSEAMYKITYISDHTCRRPASPIDASAIATPSDSSNLISFSNDCNAPLTEGTGYSLISWPSDHDDAIKVGETATTSGSTGHEIDLWSDLKEFGSFQTTAMTTTTANPYYFSTADDDADSLMFWEF*

>CmWRKY47

MASSSGSLDTSANSHPSFTFSTHPFMTSSYSDLLASANNDPPSSAPLRGSGTGVPKFKSLPPPSLPLSPPPMSPSSFFAIPPGLSPAELLDSPVLLNASHVLPSPTTGTFPSHSLNWKSNFGYNQQNIKEENKYSSNFSFQTQSSKLPPTSFQPSSTTAPTTQGWSFQEQRKKEDGFSSEKNMVKPEFGSMRSFSPEYGVVQNQSQNNSSGELQSDYGNNYPQQSQTVNRRSDDGYNWRKYGQKQVKGSENPRSYYKCTFPNCPTKKKVERSLDGQITEIVYKGSHNHPKPQSTRRSSLSSVGSSQAIVALNQAANEMADQSFTTQGSGQFDGVATPENSSISIGDDDFDRSSQKSKSGGDDFDEEEPEAKRWRREGDNNECISAAGSRTVREPRVVVQTTSDIDILDDGYRWRKYGQKVVKGNPNPRSYYKCTNPGCPVRKHVERASHDLRAVITTYEGKHNHDVPPARGSGSHSLSRPFPNNDPPAAIRPLSVVTHQSNNGGHPQGLRLQQRSSDSQSAFTVEMVQNGNGFSFPEFGNSMGMGSYMNQTQPSDNLFTRAKEEPRDHDMFIQSLLC*

>CmWRKY48

MASFPYEDSNPNPNNLNPNPNHYYTPFPPILDPASFFDFELSDFLLFDDNNNNNNNNIVDQAASSSPSMTSSEKITGGGGVDSSGSSTVIDTGSSIVVSSGASTTSIRSKNGEKKRKGEMGCRVAFRTKSEQEIMDDGYKWRKYGKKSVKNSPNPRNYYKCSSEGCNVKKKVERDREDANYVITTYEGIHNHESPFVVYYNQLPSFTTSTPT*

>CmWRKY49

MGSKSQVLLNPQALLEDHQEVTPNSQMGFFNFPSNLTFFQLPSIPQTHSPSPSFDPPNFSTSNNNTNNNNNNNSNNLSETLLSSSILPLKSSISYEIAPQHLLSLQTSTPNLWPWGEIGERLLMNGKRSNNNENNNNQLGVSKMKMKKMKGRRKVREPRFSFKTMSDVDVLDDGYKWRKYGQKVVKNTQHPRSYYRCTQDHCRVKKRVERLAEDPRMVITTYEGRHVHSPSHDSEDSEAQTHLNNFFW*

>CmWRKY50

MAKKDDSAARPPLQRPTITLPPRPSMEAFFTGGPTGVSPGPMTLLSSYFADGAVDSPSFSQLLAGAMASPMAMGFFGTGSTPNYYAKDGPGSELEFGMKQSKPVNLVVARSPLFSVPPGLSPSGLLNSPGFYPPQSPFGMSHQQALAQVTAQAALANSHMHMQQAEYQHSSVPAPTEPLARDPSFSLDEASQLAIIPSTSDTKSLIAESTEVSHSDRKYQPPPPPHGSDKPADDGYNWRKYGQKLVKGSEFPRSYYKCTHLNCPVKKKIERSPDGQITEIIYKGQHNHDPPPANKRARDNVEPAGCTNSLIKPECGLQNQAGILNKSSENVQLGSSDSEGRADTEITDDRDEDEPNPKRQNIDAGTSSVALSHKTLTEPKIIVQTRSEVDLLDDGYRWRKYGQKVVKGNPNPRSYYKCTSAGCNVRKHVERSSTDSKAVVTTYEGKHNHDVPAARNSSHHTVNNTVHHIKPLKVVAQKHPLLKEMEFGTNDQRPAVLQLKEEQITV*

>CmWRKY51

MEFLSLIQELNQGKQLANQLRNHLHPSSSSHGILLVDKILRSYENALLALSAGGGASVNSAIAPVNAAVKDGDVSKKRKLMAKWSEQVKVSSGSAVEGPCCDGFSWRKYGQKDILGSQFPRGYFRCSHRFTQGCLATKQVQKSDNDPTIYDVTYRGRHTCNKALHSTNTPQEHQNSLLQHPIPPKQEEKPLQQLHDPLCFMFSSDPIRVKSENLDNANGGLLQPFCPPSPMFGSEVQDDQSPFRESECSLTFESNDTFGLCCDFETGFVSIPSSMTNISIGDLEEYCSFDNLEMFC*

>CmWRKY52

MVSTGDQLENGVDSDQLDHENSSDSQPQASQDDPGGTNASKSDHKCTGASSNTLEEAVKQPEVTIALVDRGDISNIVTEKVTHKPITAEQNPLSVLKVCITSSIREKVSEDGYNWRKYGQKLVKGNVFVRSYYRCTHPTCMVKKQLERTHDGKITDTVYFGQHDHPKPQPHIPIPVGVVTMVEEKLGEHASGNSQDKTSTTLSPTPQQTELTDMRQPPSVIASDNVKDEASKRSRTNDEASKRSRTIDEIDSDDTPDLKRDFKRKKRCNIDVTTVADKSIVESRVVVQTPSEVDIVNDGYRWRKYGQKFVKGNPNPRSYYRCSSPGCPVKKHVERASHDPKVVLTTYEGQHDHVLPPIRTVTLNSVGSTTAHSDETKPKPVSTVVHASKDPQSDSSSEGKLIEENGKLNATETSDDIILDGVVVNPSPGVASEQNKQLKVAIES*

>CmWRKY53

MAVELLTGFTNPPQLSSRSPMDQDSAVQEAASGLDTLKKLITLLSHSPPSNLDSDCQAVANAAVSHFRKAISLLGRSSRTGHARFRRAPLDSSKIYNATPIQQIPPPSLDRLDSATTINFSYSAAPTSSFLTSLPASDSEIKLQHQPSSSSFQITDLSRVSSVVSKPSSGLKRKCGSENLGSGKCAGSSGGRCHCSKKSRKLRLKRVVRVPAISSKNADIPPDDYSWRKYGQKPIKGSPYPRGYYKCSSLRGCPARKHVERASDDPSMLIVTYEGDHNHSQSVAEASSLILESW*

>CmWRKY54

MEFFFFSATLGDPSGGGRRRQPDLAVVGKGNPPLSPPLYSPSSFFTIPPGISPTQLLDSPLLLNSSPILLSPSTRDLRKSTTNNSGHHQQNIKQEHNITEFSFPRNHTTKSSSSSMFQSSSTVQTEAWGLESESDRGEWEMRNRSGSEDGFNWRKYGQKVKKVIFLFFFSSSSSSSSSSSSFEPVTMAAQLKINSIASHSLTTPENSSITIGDDHSDQAHPKRWKSESEKEIMTTGGGGKTMREERIVVQTISNVDKLDDGYWWRKYGQKVVKGNPNPRSYYKCTYPGCGVRKHIERASHDLRAVVTTYEGKHNHDIPAARGGKPIP*

>CmWRKY55

MAAFSSWLLDSVDTKLDLNSMPLRFSGEPPKERNYMDVERKVTVKEETGALMEELKRVSAENKKLTEMLTVVCENYNTLRGHLMEQMNKNGEKEISSSKKRKSESSNNNNNMVGMNGNSESSSTDEESYKKPKEETINKSAKITRVQVKIGASDSNLVVKDGYQWRKYGQKVTRDNPCPRAYFKCSFAPSCPVKKKVQRSVEDQSVLVATYEGEHNHPHPSQIEATSGGAAARSVNIAPAVVTAAPGSSSAQAVSLDLVKAKPIMEAKTIANPRFDSPELQQFLVEQMASSLTKDPNFTAALAAAISGKIFPH*

>CmWRKY56

MENFGEWDQNKLKNELLKGMELAKQLQIQLNVRSTPSSSMAASSSSSSSSSSSSSSSSNDGCELLVQKILCSYEKALSLLNSYGAQINIYESPSSFNGGSPRSEDSDREFKDPFDITNANSFRKRNILPTWTQKFQVSPGMAIEGSLDDGFAWRKYGQKGILGAKHPRGYYRCTHRNLQGCQATKQVQRSDDDPTIFEITYRGKHSCSQVSNLSTPCTTTSEFQQQNQGVVVELPDQKKAQNQQTSPDALLDSWSSLRVITQNLDTTHEPTLQFHPLCYDRVEFASTSTVDVNFTEFSSFLSPTTSGSGLSYFSASSSGLSEGFVVGNQNLNNLQPNNCEIFSSPTSALNTQTTTALDFSFGELQMEPTFSFDNTDFFS*

>CsWRKY1

MEVDWDLHAVVRGYSAAPSAATIVPASSSSSSSSSNNPVVPFSFGRDLTNNQMKNHFFSLQDPFQPSNCNSTQELHELFKPFFPKSQPSPSPPPPPPAPPAPSLLSSPPAPKILTHQKQSTHLPKQLHSTSASAPRSKRRKNQLKKVCQVPAESLSSDIWAWRKYGQKPIKGSPYPRGYYRCSSSKGCMARKQVERNRSDPGMFIVTYTAEHNHPAPTHRNSLAGSTRQKPITPSTTASGSEKLDPKQPVCSSEEQSTITESKEEKEELLMAEDEEDDDLGISDLIVNDDFYVGFEELDSPITDDCFSDPFPANFDLPWLFNGNPDGEIVKLPC

>CsWRKY2

MSDEMFKDLFYSGMDEYESIVRAFGITSDYSNINNEISGTTAMNSSCSLSSSDAGGGEEDDSVKEKEKQISKDVVEDNGGESSKAAGSGKSKKKGEKKEREARVAFMTKSEVDHLEDGYRWRKYGQKAVKNSAYPRSYYRCTTQKCGVKKRVERSYEDPSIVITTYEGQHNHLIPATLRGNLSAASGTFSPSMLTPMPVVGGVGFLPAELLSNAGNNQAVGGGATVYSHNNFDYTYNGRQPEYGLLQDIFPAPSSFFNRQP

>CsWRKY3

MAVELLSGFTNAPQLSSRSPMDQDSAVQEAASGLDTLKKLVTLLSHSPPSNLDSDCQAVANAAVSHFRKAISLLGRSSRTGHARFRRAPLDSSKIYNATPIQQIPPPSLDRLDSATTINFSYSTAPTSSFLTSLPASDSEIKLQHQPSSSSFQITDLSRVSSVVSKPSSGLKRKCGSENLGSGKCAGSSGGRCHCSKKRKLRLKRVVRVPAISSKNADIPPDDYSWRKYGQKPIKGSPYPRGYYKCSSLRGCPARKHVERASDDPSMLIVTYEGDHNHSQSVAEASSLILESW

>CsWRKY4

MDNNNYQQAGDLTDVIRLSSAPVAGHFSSEFSLDPFSGDRQLWSHLPADNSSMNFGDPLSFPTRDPFLLPHFSPFNFASDGGGEGLAADDQPSNLLSHMLQISPSSGDGVISTPPCESLATAVGNSPRSTSVRGGGGLVLPTGGSNSLCLMENSGIQISSPRNSANKRRKSQVKKVVCIPAPAPANSRSSSGEVVPSDLWAWRKYGQKPIKGSPYPRGYYRCSSSKGCSARKQVERSRTNPNMLVITYTSEHNHPWPTQRNALAGYR

>CsWRKY5

MEEVVAADSLRYPFLDASDSKSCLGTFMELLEVDQHFSSQFDVFETSSPSLSSSLISNPENLEIWNQWPTTPNYSSSISSTSSEIVNGELTTEPNLEGGEQKQDQQPTVKADKQLKTKKRSPKKKGAEPRFAFMTKSEVDHLEDGYRWRKYGQKAVKNSPHPRSYYRCTSVACNVKKRVERCLQDPSIVVTTYEGQHTHPSPIMARSTFFPPPISATLYNDYSIQNSHNSNVMSHSIAWCHH

>CsWRKY6

MAVDLMSFPKMDDQIAIQEAASQGLKSMEHLIRLLSHKQSSNHVDCSDLTDATVSKFKKVISLLNRTGHARFRRGPVSSTSSSSSGSSAHLSQNQAMTLTPTPMTLDFTRPNILNSNPKGADLEFSKETFSVSSSSSFMSSAITGDGSVSNGKLGTSIFLAPAPTASGGKPPLSAAPYKKRCHEHDHSEDLSGKFSGSTSISGKCHCSKRRKNRMKKTIRVPAISSKIADIPPDEYSWRKYGQKPIKGSPYPRGYYKCSTMRGCPARKHVERDPNDPAMLIVTYEGEHRHTQSSLPENMAAAGGVALVFESS

>CsWRKY7

MENFPSFLSTSSSSSALSLQQILFSRASNHGGDHFRQPPFVSDGFPILFRDVSTDDMSFDATSSVKDDDRNNVAVSVSTERFRVDGSSGRSGVVEYGLKKEEGEGRGRGEDCDKKKKMRNRRFAFQTRSQVDILDDGYRWRKYGQKAVKNNKFPRSYYRCTHQGCKVKKQVQRLTRDEGVVVTTYEGIHSHPIEKSTDNFEHILSQMQIYTTSY

>CsWRKY8

MEVSSHHSFKPNPNHEHHLLSTQPTPEEEQPLSKKRKVVQKTVVTVKIGSKKAAIGIGKMKNEGPPPDFWSWRKYGQKPIKGSPYPRGYYRCSTTKGCSAKKQVERCKTDGSMFIITYTSSHNHPGPNISTLNLDQNYQQEIDPPQPLDRDDDEDHHDLVPNQAQDHDNNSNNDDKNSIIISQSTEEEEVEEVEEEEEDELLLVEDEEKKGIEKIKDECLDQEPIIISSSSNSSSCCDELMIIKTKKSEIENHDHFFDELEELPIPPPFSSTLMRSSYSFDEIRISAAPS

>CsWRKY9

MDRRVRTNPFLSEQEDPEATSDDGLPESPSDCNDSKPTAAPPPKKSRRGVQKRVVSVPITDVEGSKSKGEAYPPSDSWAWRKYGQKPIKGSPYPRGYYRCSSSKGCPARKQVERSRVDPTKLVITYAFDHNHQLPVTKSHHHHHHNSSPSSAVIAAVSAATDFPSPGSTTTSSSTSSGDNTNAAPSSPAAKFEEAAAVFASQPELELGGDSLMIKPCIGDFGWLGEVAYDRILEGPICGGGDIFDDADVMVLSTRGDDEEESLFADLGELPEGSVVFGRRRTVQPNGPNRTCGTVLNC

>CsWRKY10

MDAAAAFGRPRPVVKTEKPPLRDLSDNDRSSPSKQQQQLLLRGNNHAKQEHDTTEDKTSCSSDQKDLSCIKLQEDQLESARAEMGEVREENQRLKQSLTQIMKDYEALKMQFLGIVGRDCKKVQDEDNDVNKEQQQQQHDDDQIELVSLSLGRFPVSEKKKIVDEKSCMNIIGGDHNEEAACKEALSLGLNCKFEREESMMAVAKEVDSPNSFDHESTKEEAGETNWPSKGGKTMRSVEDDVTPQNPPKRARVCVRARCETATMNDGCQWRKYGQKIAKGNPCPRAYYRCTGSPTCPVRKQVQRCADDMSILITTYEGNHNHPLPASANAMASTTSAAASMLLSGSTTSATAASSSSTASNSLHGLNFYPNNSKPNFYLPNSNSSIISSTSPTHPTITLDLTSNPSSSPSSSSTHFGKFTSNFPNSRYPFTGQLDFGSSRNNVLSWNNGLLSYNRNNHPTTTTTTANNIYQNYIQQQQRNPTTSLQHQQPPLPDTIAAATKAITADPSFQSALAAALTSIIGTGGASASAGLTKSLSGRGEQSLFQLMTTAATTNKGNGCGTSFLNNITTTTTTTSNSPPTGNMVFVPTNSLPFSNSKSASASPGDHIDLTN

>CsWRKY11

MASSSGSLDTSANSHPSFTFSTHPFMTSSYSDLLASANIDPPSSAPLRASTTGVPKFKSLPPPSLPLSPPPMSPSSFFAIPPGLSPAELLDSPVLLSASHVLPSPTTGTFPSHSLNWKSNFGYNQQNIKEENKYSSNFSFQTQSSKLPPTSFQPSSTIAPTTQGWSFQEQRKKEDSFSSEKNMVKPEFGSMRSFSPEYGVVQNQSQNNGSGELRSDYGNNYPQQSQTVNRRSDDGYNWRKYGQKQVKGSENPRSYYKCTFPNCPTKKKVERSLDGQITEIVYKGSHNHPKPQSTRRSSLSSAGSSQAIVALNQAANEMADQSFTTQGSGQFDGVATPENSSISIGDEDFDRSSQKSKSGGDDFDEEEPEAKRWRREGDNNEGISAAGSRTVREPRVVVQTTSDIDILDDGYRWRKYGQKVVKGNPNPRSYYKCTNPGCPVRKHVERASHDLRAVITTYEGKHNHDVPPARGSGSHSLSRPFPNNEPPAAIRPLSVVTHHSNNGGHPQGLRLQRSSDSQAAFTVEMVQNGNGFSFPEFGNSMGMGSYMNQTQPNDNLFTRAKEEPRDHDMFIQSLLC

>CsWRKY12

MAAFSSWLLDSVDTKLDLNSVPLRFSGEPPKERNYMDVERKATVKEETGALMEELKRVSAENKKLTEMLTVVCENYNTLRGHLMEQMNKNGEKEISSSKKRKSESSNNNNNMAGMNGNSESSSTDEESYKKPKEETISKSAKITRVQVKIGASDSNLVVKDGYQWRKYGQKVTRDNPCPRAYFKCSFAPSCPVKKKVQRSVEDQSVLVATYEGEHNHPHPSQIEATSGGAAARSVNIAPAVVSAAPGSSSAQAVSLDLVKAKPIAIMEAKTFANPKFDSPELQQFLVEQMASSLTKDPNFTAALAAAISGKIFPH

>CsWRKY13

MDCSWPDTTPFDRRKAADELLRGRELAQQLRAYLQISSTPASQDLLTRILSSFSKTLSILNHRCDSDDINGSIVDSPEDHGSRKSEESGDSCKSSTPNNDRRGCYKRRKSCQSWARESCDLVDDGHAWRKYGQKTILNAKYPRNYYRCTHKYDQTCQATKQVQRLQDNPPKFRTTYYGNHTCSNFLKASDIVLGSSNFDDSCSGVLLSFDTTAAPNFFLPHDPTLVKKEEVVTPDAGSGRDDEAVCSPSDYMSTADDHLSEVFMGSVVDFEDDDLPPFHF

>CsWRKY14

MSNINQTINTLAGGSSDNRTNNFAMEVPKFKSFQPPPFPMSPSSYLSSFSSGLSPTEILNSPLLFSFGVFPSPTTGALNLRNDYEEVDQQEMKGDVKNYSVSAYNPQTGSSVSSYFQSSSSNLTLLNPSGLSCDESGAKSEFVNTEMAAAESKQNSQLAIYNREQQKSENDGYNWRKYGQKQVKGSENPRSYYKCTFPSCPTKKKVERSLDGQITEIVYKGTHNHAKPQPTRRSSNSGVYDPSAAETGVLQEDCSVSVGEEEFEPNSPFSNSIEDNENEPEAKRWKGENENEGYCGGGSRTVKEPRIVVQTTSEIDILPDGYRWRKYGQKVVKGNPNPRSYYKCTSLGCPVRKHIERAANDMRAVITTYEGKHNHEVPAARGSGGGGYNTINRPIPTNIPMALRPLSSVTSHSFPANFPAAFRPGNLGMSETGTQASSFPFQTSHGVLPSFQVSGFGSAAKEEVRDDTYFINSFLS

>CsWRKY15

MDNKATERVVIARPVASRPTCSSFKSFSDILTCAFDTSPPNMSSETRVAAIRPKTVRFKGKISETIPGTNSHSSSDTLAVSEIKTTVLFKPLAKHVSKRTVSQLSLMASGFIIRTVYTCTSIPLYGNTNLQNCLPPPPVEVCIQCPNQDDGNFQSALTSNLCIQCPNQDNDNFQSAPTSDLPQNITSTVENSQSIGSSRVTLSYSKKDPTLLRPQISGAQPSYDGYNWRKYGQKQVKGSEYPRSYYKCTHPSCPVKKKVERSLDGKVAEIVYKGEHNHPKPQPLKQNSSGTQREGSISNGTTQDTNPELWFNYLNGRIEGCESRIENHIEKTCQDRVTIPFDPFSNQEVNARCGISDNNSCGLSVECEEGSKGLQSMDDKLRSKRRGGKNPTNEGETLIEGVNEHHAMAQDSTGIEISGKGVRWRKYGQKVVKGNLYPRSYYRCTGLKCKARKYVERASEDPDSFITTYEGKHNHGISLGTSISVAPEME

>CsWRKY16

MEAQQALATIDDDRVQSTGEGDDKQASPNPKQHIHLKVPSMGDLEKPSMENLSVPSASNSITWKEEDDEQRHEERMKRTKVEMREVKEENERLKKYLDEIMKDYETLKRKFHEIKNNHDDQIREEGKKSTQTSGSTINNNNNDNDDDHQVEAEVDDMVSLRLGSRFSTHHQNKNTSSSSLSLTNKILDLKQDYVIQTPSTIDHSIHSPTHSEPKDQEEAGQTTWPPSKMPKPGGLPSPATGEDEVSQQNPPKKARVCVRARCDTPTMNDGCQWRKYGQKIAKGNPCPRAYYRCTGAPTCPVRKQVQRSVDDISILITTYEGTHNHPLPVSAMAMASTTSAAASMLLSGPSSSTSSQPGLNHSFTAPATAANLHGMNMYLSNNTNSKQFYLPNSSMLSSSLNHPTITLDLTSNPPSTSSSSPFHKIPLINNNNYPPKYPFTNLDFASSQPNFMSWNNNNNAYSNITKNNAIIGMGSDFAKQLPLHTNIYQACLQQLSKPSTTPQPPALPDTIAAATKAITSDPSFQSALAAALSSIIGGGETGPSVSSLVVGGGGGGGQGSMGFEAAKSLTCSTSKSTPSSSPGDSRDNGK

>CsWRKY17

MDEEEDDELEEERSLKKVKSEESGGELKKKKKIRKRRFAFETRSQVDVLDDGYRWRKYGQKAVKNNKFPRSYYKCSNEGCKVKKQIQRLTNDEGVVLTTYEGVHSHPIEKPHDSFQNILTHMHIYPSSSSSF

>CsWRKY18

MLKVVSIKNLVSQVDVCSRDHRHDQNDEGSRSERANLKVSPPALALALETSSTTQAYARTTFKDQALMVKDGYKWRKYGQKITKDNQSPRAYFKCSSPGCPVKKKVQRSLENKSMVIVTYDGHHNHNHNHENASPPPLSSSQRGSSSSPPLPVETNRVALPMSLNLDLTLSRHADDHKL

>CsWRKY19

MLGVVVENYSVLKNQVIDLIMKTRKRKAAPGCDNCCNFNRSASSDQYCGCCSDDNDSCYNKRPRENNSKPKVMRVLVPTPVSDSTLIVKDGYQWRKYGQKVTKDNPSPRAYYKCSFAPTCPVKRKVQRSVEEPCYLVATYEGQHNHPKPNSGIEYQLIGPINLGSNTKLDSSNVTSSPSSSIKSPSSSSLMPSMSFDHLTKSQPQIRSPSSSNSSSSTQKLLVQQMATLLTRDPNFTRALATAITGNMVDSEIWG

>CsWRKY20

MDHFGHLTDDGVLVDANAGSFAQSRPSKRRRNGWIPTKDSHSPILSSPSLTIPPGINPTLLLDSPVMLLNTQDLPSPTTGTFPPIHQIKDEQSLLNPVMPEDGISHGSEDSFFRFAPQGELCTLQSLLRIENQNLLRFIMEAEIDHQALESEKTLMDFEFVPDIPKEAAVLKYEIAPSTDNSYFDGKIVNGNCENMESCLSSITTNQPCIHEESTQGDDIDTQHPLEDEQKGSYIPMGMLRTSEDGYNWRKYGQKQVKGSEYPRSYYKCTHPNCLVKKKVERSLDGQITEIIYKGAHNHAKPDPNRRAMAGSVPISGDNPEIGEGGGNHSKLEAGLTWRNSQYGVKDIKPISNCSVDGLERTPSVSVLSELSDPLLNPQEKTVGVLEPVGTPELSSTLASHDDDNGGGGDDDLTTQGSISVCTEADDAEPELKRRRKEDSSIETNLASRSVREPRVVVQIETEVDILEDGYRWRKYGQKVVKGNPNPRSYYKCTSAGCLVRKHVERASHDLKCVITTYEGKHNHEVPAARNSSQVNSGNGNAQPPASHVQPNMGLSRNSNVPKSETEIQDLATHFYPKPEFNNDYQRSGFDTFTNDMKLGAPPFCQMKFPPLRNTLPYSTFGLSSKHTATGISGSLASVVSDFPISLPLNQKLSAAGYDYTNGRPILPFQVFLAGQQLRETDRFLTPKQEHDDDNICASFQPVVDSSSGSSSSSISSVYQQIMGNFT

>CsRKY21

MEFLSLIQELNQGKQLANQLRTHLHPSSSSHGILLIDKILRSYENALLALSGGAAASVNSAIAPVNAAVQDGDVVMAKWSEQVKVSSASAVEGPGCDGFSWRKYGQKDILGSKFPRSYFRCSHRFTQGCLATKQVQKSDNDPTIYEVTYKGRHTCNKALHSTNTPQEHQNTFLQHPIPPKQEDKPLQQLHDPLCFMFSSDPIRVKSEDLEYANGGLFQPLRTPSPMFGSEVQDDLSPFRESECSPTFESNDMFGLWCDFETEFVSIPSSMTNISIGDLEECFSFDNLEMFC

>CsWRKY22

MDRIKEENKALRKAVEQTMKDYYDLEMKIGFFQQNNNLNNKLECDHNFLSFHGNENKRHEELTKHDLELGEMAKKKRRVGSASKEDEMRESELGLSLGLHTKNSNDDLEQEDNDRELLIEEERREIKNKENSIIMSNFNSIQNKPQRPELQAMAPPQNRKARVSVRARCESATMNDGCQWRKYGQKIAKGNPCPRAYYRCTVAPGCPVRKQVQRCLEDMSILITTYEGTHNHPLPVGATAMASTASAASASFMLLDSSNTNNTNLSNSLHLNPNILNSSSPSFLQTQNPTNHLFTPLFPTSSTSHFPHSFYHSNFQPNHLVGPLDRRTWKPTDDNKPPPFTPDAVSAIASDPKFRVAVAAAISSLINKENEHMTTSMTGETVTDGKGGGGSDSDSGNKKWVVESLSSKSNGN

>CsWRKY23

MAVELLVGFGDATPSNHFTPNMEENAAVSAVQEAASAGIQSVQNFLRLMSHTTNHQHSQHDSSTSSTPNNGYQAVADSVVNKFKKVISLLDRNRTGHARFRRAPVLTTTTTTTTPPPPPPPKVKPQHQDPSSSSPISVPPVQVKKQESVSAFKVYCPTPSSVVRLPPLPHNNPHQPSHPSNTFQAQQNTSSVVLKNGSVDRKDATTTINFAASPPISAANSYISSLTGDTESLQPSLSSGFQFTHMSQVSSAGKPPLSSSSLKRKCNSMEDSAMKCGSSSGRCHCSKKRKNRIKRVIRVPAVSSKLADIPPDDYSWRKYGQKPIKGSPHPRGYYKCSSLRGCPARKHVERALDDPTMLIVTYENDHNHAHSTETPAPLVLESS

>CsWRKY24

MAKKDDSATRPPLQRPTITLPPRPSMEAFFTGGPTGVSPGPMTLLSSYFADGAVDSPSFSQLLAGAMASPMAMGFFGTGSTPNYYAKDGPASELEFGMKQSKPVNLVVARSPLFSVPPGLSPSGLLNSPGFYPPQSPFGMSHQQALAQVTAQAALANSHMHMQQAEYQHSSVPAPTEPLVRDPSFSLDDASQLAIIPSTSDTKSLIAESTEVSHSDRKYQPPPPPHGSDKPADDGYNWRKYGQKLVKGSEFPRSYYKCTHLNCPVKKKIERSPDGQITEIIYKGQHNHEPPPANKRARDNIEPAGCTNSLIKPECGLQNQAGILNKSSENVQLGSSDSEGRADTEITDDRDEDEPNPKRQNIDAGTSGVALSHKTLTEPKIIVQTRSEVDLLDDGYRWRKYGQKVVKGNPNPRSYYKCTSAGCNVRKHVERSSTDSKAVVTTYEGKHNHDVPAARNSSHHTVNNTVHHIKPLKVVAQKHPLLKEMEFGTNDQRPAVLQLKEEQITV

>CsWRKY25

MAVDLISHLSPLPTMEPNAVQEATSGLESVHKLIRLLSIPNPHSLPSSTQSPIDFPTDCRAAADAAVSKFKKVISLLGRSRLGHARFRRAPLPQQPHYVTPIQQIPPHPHLNNNNNINDESLNFSAHNSFISSLTGDADTKHPSSSSSPFLISNLSQVSSAGKPPLSSSSLKRKCSSENLRSGKCAAASSSSRCHCSKKRKLRVKRVVRVPAISLKMADIPPDDYSWRKYGQKPIKGSPHPRGYYKCSSVRGCPARKHVERAVDDPAMLVVTYEGEHNHTLSLPETSTLILESS

>CsWRKY26

MMPSPQRDDTRVKEPSWQAAGIETKGVKRKKPRENGRTNRVAFITKSELEILDDGFKWRKYGKKSVKNSPHPRNYYKCSSGECGVKKRVERDRDDSSYVITTYEGVHNHESPFLMYCNGSKLFHPHPICPNSSSPPYSSTTTL

>CsWRKY27

MGSSASEEEMMVRLLKGGDQVAAAVDGAIKWRPPDERGVSFMRGRNAIGNYGGEDDHNNENDGKPRLRVSTMKMKRIKGRKKVREPRFSFKTMTDVDVLDDGYKWRKYGQKVVKNTLHPRSYYRCTEENCKVKKRVERLADDPRMVITTYEGRHAHSPSDHNLEDSFMGHLPSSHLTNFFC

>CsWRKY28

MDSLAAIPWSDTHTTVSDDHLFTLTNLLHDDENHASSPLFLLPQDADDNNRAIRVPVPGGATYFGPTIEDIENALSIGTPRSKDLQSHTQISHTGFSIVERANLNKVEHKYSLRIKSCGGNMVADDGYKWRKYGQKSIKNSPNPRSYYRCSNPRCSAKKQVERSIEDPDIFIITYEGLHLHFAYPFFLMGQSPQAQSPTKKPKTIDPEQPEAHEKPSFLDPIESSGSQGLLEDMVPWLIRNPSTHHNALSNSSSCLSHRSPPPTPPSPSTSPTFITSCF

>CsWRKY29

MDCILDFQDFAFTFINVGEIFCRRKVKKKGQKRIRQPRFAFMTKSEVDHLEDGYRWRKYGQKAVKNSPFPRSYYRCTNSKCTVKKRVERSCEDSSVVITTYEGQHCHHTVGFPRGGLTIAHETSFGSQFSPQIPHFFYPDPPPPPTTTNNHNPPTPPIDQPLHHFPSTPSSTEQQEPPNSNLQQLPSNEGLLGAIVPHAMMRRTT

>CsWRKY30

MDNRGGAVIEAHVVEYVSKMTNQHLTTSESDLSEQPGFEFTDWMFDGWLNENSSSLTDSVMYPVYQEGEVDEFVGNTIQQGEPSSRDYGREREIRERFAFKTKSEVEILDDGFKWRKYGKKMVKNSPNPRNYYKCSVEGCPVKKRVERDREDPKYVITTYEGVHTHESS

>CsWRKY31

MNFFPSDDKSRVLSASHSNLTPTKLPFNVNTGLNLLTTNSCSDQSMVDDGVSPNPEEKRVKNERAVLQAELERINSENLRLKDMLNQVTSNYQTLQMQFNTLIQTQKTEDVGDPIEENPDGSGGGGNNNNNNNTNISNKLVPRQFMDLGLATNTENDEASMSSSEGRSGERSRSPGNTGEVASSKRQSPDQSSNWGSNNNNNNNKVPKFSSSSGKEVDQTEATMRKARVSVRARSEAPMITDGCQWRKYGQKMAKGNPCPRAYYRCTMALGCPVRKQVQRCAEDKTILITTYEGNHNHPLPPAAMAMASTTSSAARMLLSGSMSSADGLMNSNFLARTLLPCSSSMATISASAPFPTVTLDLTQTPNPLFQRPATGHFPIPFAAAAPPQTFPQIFGHALYNQSKFSGLQMSKDMEAPQPPPPPQNPFTDTLSAAGAAIASDPNFIAALATAMTSLIGGSHHQKENGNGNSNVDNKTSSNSQQ

>CsWRKY32

MVSTGDGDQLENEVDSDQLDHENSSDSQPQASQDDPGGTNASKSDHKCTGASSNTLEEAVKQPEVTIALVDRGEISSIVTEKVTHKPITAEQNPLSVLKVCITSSIREKVSEDGFNWRKYGQKLVKGNVFVRSYYRCTHPTCMVKKQLERTHDGKITDTVYFGQHDHPKPQPHIPVPVGVVTMVEEKLGEHASGNSQDKTSIALSQTPQQTELADMRQPPSVIASDNVKDEVSKRSRTNDEVDSDDTPDLKREKKRCNIDVTTVADKSTVESRVVVQTPSEVDIVNDGYRWRKYGQKFVKGNPNPRSYYRCSSPGCPVKKHVERASHDPKIVLTTYEGQHDHVVPPIRTVTLNSVGSTTAQSDETKPKPVSTVVHASKDPRSDSSSEGKLIEENGKLNATETSDDIILDGVVVNPSPGVASEQNKQLKVAIES

>CsWRKY33

MEEVEEANKSAIESCHGVLNLLLQPPPSPHHHQHHFKNLMLETKEAVFKFKKVISLLNSDFSHPRFRNFNKIPLPLPQNSLLDSPNYTLHPPNKNLFNSPPGFNSKVSILLGNPDLELSQNDKNSLHIPKQSPSLSFSFPHHHHPQQQQQQQQQQQSLLAHQKQMKHQAEMMFLRNNNGMNLNFDTSNCTMTMSSARSFISSLSMDGSVIGDRSSFHLIGPSTTTTTTSGNSKRKFSARGEEGSLKCGSTSKCHCSKKRKHRVKRSIKVPAISNKLADIPSDDYSWRKYGQKPIKGSPHPRGYYKCSSIRGCPARKHVERCLEDPSMLIVTYEGEHNHPKMSTQSAHT

>CsWRKY34

MDIFLDLNVDPNSSYANSTMDEVAHHSSKRDQFDGEIYGDKEKLALSLSNKGSESSPTLEQELDRKIQENGKLSQMLRIMYEKYINLQKQVMYLLSNQKQSTEMEGVCSRKRKAEGEQEDYENLEGICSTRDEDFNRWLKRPRLNGNSKVSKVFVQKDASDPSLVVKDGYQWRKYGQKVTRDNPSPRAYFKCSSAPNCPVKKKVQRSLEDPTILVATYEGEHSHASHFQTELSLRSINGGKGSAVPVLATIKPSCATVTLDLIHEDGLFKSPKDYASSESAEAAVWQEFLVQQMASSLKKDPEFAGIVAGAISGKVLGNQTNRE

>CsWRKY35

MAAGNDDWDLSAVVRSCNSAGSATDPTSAAAEESALSCLASLTFDDDPNDVAFSFSDIFQPKQPNGFHELHQAFISFLPNPSTTATTVTTVPAAEPEIPYLTTPPTNRHFRQLGLNYRKNQQKRRVCHVTADNLSTDMWAWRKYGQKPIKGSPYPRNYYRCSSSKGCGARKQVERSNDDPETFTITYTGDHSHPRPTHRNSLAGSSRNRSSSSSSSSRHPTMGDSDPPMMTASVLLPSSSSPAASPITPLNDYDSTIGEKDGEMFEDMPIDSDDEDDDDDILIPNLTVRDEIYVGFDGVGRGRSS

>CsWRKY36

MAVDLAAFPAFFDDQTAIEEAATAGLQSMNHLIHLLSKQHHHHHHNIDLNSSLLTDFTVSKFKRLISLLNRTGHARFRRGPSDSPNPVLNSLDPPQKTHFSKLNFSPVSKVPESRDSTTTSSFVSTVTGDGSVSNGKLDLSVYATPPANAGKPPLAMKSKCHDVSGFGCKVPNSKLCHCAKRRKSGMKKTVKVPAISSKIADIPSDEYSWRKYGQKPIKGSPYPRGYYRCSSVKGCPARKKVERARDDPAMLLVTYEGDHRHPHPTVTDGVSQKS

>CsWRKY37

MDAPEFSHGRISPAELRRKITAKLLPGQDSAAHLRILLQSATATEQDKRALATKILTSITEAISILESAGEELSCPDHSLCSDLDSGESRRSRAVKDNPSRANKRRTSRTTEDNYGWRKYGQKAIHNTTYPRSYYRCTHKFDQGCQATKQVQRMEGDDSEIMYNITYISDHTCRRPASPIDASAITTLSDSSNLISFSSIDCNGPFTEGTGYSLISWRPSDDDVVKIGETATTSGSTDDHEIDLWSDLKDFLELPNNGYDNDNEESILFLNRR

>CsWRKY38

MEEAIISLILRGCSLARQLEFNVLNLGSNNINISDQSHFMARSCDEILGVFSAAKDRLGSHEPPPLTGVQGEVGLEEWLKSTCSQAMELSQMQMISPSPPSAIVSVPAAVKDSGKLMAMGLSSSSSSSSTKARRRKDDTEKRTVRVGAPRIGNTELPPDDGFTWRKYGQKEILGSRFPRGYFRCTHQKLYHCPAKKHVQRLDDDPHTFEVTYRGEHTCHMSATAPSAPPPPPLPTDQPISHFHPAAPWLSMDVVGSGSTSGAPSTVRYGKDVGDQFAVVDMADVMFNSGTSSSNSMDSIFAAYVGEDHKWEKEDKKK

>CsWRKY39

MEDWGLQAIVKGCNGIPIGSSTTAATTRLMEDSNWYSFLRSDQEEDEFFSSCVYNSSYNNPQISSSSIFHDEFEGLFGRNSSNNSAAASISHLLRDFKEPADQKLHHKNQIIQPTKQKQSKKSRQNRVVKEVKADKVCSDSWGWRKYGQKPIKGSPYPRSYYRCSSSKGCSARKQVERSLSDPEVFIVTYTAEHNHAEPTRRNALAGTTRKKFPALENPNLDMILSPNNSTSVASIEEDQHPMEGVTDGEVLIDMPFEFFTGLEDLLFG

>CsWRKY40

MVMGKRSTIASPRIAFQTKSVEDVLDDGYRWRKYGQKAVKHSNHPRSYYRCTHHTCNVKKQIQRHSKDPTIVVTTYEGIHNHPSEKLMETLTPLLKQLQFLSGI

>CsWRKY41

MAEPESFGTHQLGSSNAATEGQEDDEEEMDDSDDESEVDLGGEGGGTGSEFKPTELTTGSSLNEALVTGSLSETLTVASSAENGPSDGLQPDEGAELKQAPSSHSEPLAVEATQTDKVQEQNRLQLTIFKGPDSDQSPTSVTQSISSYASSNLSEHKLSPKRVQKICKPEPSQKNFFNHKTPSSVPNARTPASDGYNWRKYGQKQVKSPKGSRSYYKCTYSECFAKKIECCDDSGQTTEIVYKSQHSHDPPRKISTPKESKLVPYVEPVVKKIIAEHSRRVINDSDSPTPSKEPLREAAIVVFERKRQHSNDSNGNDEYKIKDENDDEPGTKQIVKKSSAGNSGTPLKPGKKPKFVVHAAGDVGISGDGYRWRKYGQKMVKGNPHPRNYYRCTSAGCPVRKHIESAVENPNAVIITYKGVHDHDTPVPKKRHGPPSALLVAAAAPASMSSNAQPKKTDVVESQISSTQWSVDAEGELTGEALELGGEKAMESARTLLSIGFEIKPC

>CsWRKY42

MEEVEEANREAVESCHKVLNLLTLPSSSQDHLKLRSCLMAETGEAVFKFRKVLCLLDSSGLGHARVRKKKKVNNFLFNSSSSSSSSSPFPLPQSLFLETYSPDCRMDHLQGRNLQMGPLCLGNPSLELNTNAKTCSIQQIQSQSAALYHHHHHHLLQNRVVLNNNPNPPQPEVVYLRSSNGVNLNFDSSSCTQHTMSSTRSFISSLSIDGSVANLDGSAFHLIGAPRSSDQNSYHKRKCNGRGEDGSVKCGSNGRCHCSKKRKHRVKRSIKVPAISNKLADIPPDDYSWRKYGQKPIKGSPHPRGYYKCSSMRGCPARKHVERCLEEPSMLIVTYEGEHNHPRIPSQPANT

>CsWRKY43

MDDVSFNHNNHHQFYSSDPFEDSDELKPTPDSPPPTSTIAAAATTKKGRRGMKKKIVSVKINGDSPRNSSGSATPPSDSWAWRKYGQKPIKGSPYPRAYYRCSSSKGCPARKQVERNRLDPTTLVITYSCEHNHSGPVSRNNNNNNNQNNQIVVMKPGSPETVAVHQEPEVEEKFVEIGGGEESLITADEFSWFGEMETTSSTVLESSIFSGRASTGLVDHSISSDVAMLFPMGDDDVDESLFADLGELPECSLVFRRGGGRGLPVDEQPAAAQRRITPWCGTTT

>CsWRKY44

MELLGLNYNSEFTEAFNNPPATPNCSSSVSSASSDALNDDEPPPPPPQQKEDKTFVFMIVLIRSILKMGLKGIKKRKEKEKEKKARFAFMTKSEVDHLEDGYRWRKYGQKAVKNSPFPRSYYRCTSAACNVKKRVERSFADPTVVVTTYEGQHTHPSPILSRSALAVAIPPPSFIPGAGGECVGGVVAMPWLKPSNNDAHDGNTVPAMSHQYFQNSTYITAQNVAANYNRNNHIGAANAAGILQEKRKKEIDFPYVETLVTSSMGLLT

>CsWRKY45

MFFPCSDGGGGLSAYHHADMSSGGASDMFGNFQGGDMEAVSGFLGMKREVDGGAVEAEGGGKKKGEKKVRKPRYAFQTRSQVDILDDGYRWRKYGQKAVKNNKFPRSYYRCTHQGCNVKKQVQRLTRDEGVVVTTYEGMHTHSIDKPTDNFEQILSRMQIYSTPF

>CsWRKY46

MGRTDDNIAIIGDWVPPSPSPRTFFSAMQMLGEDIGSKPSMDTTTISDHKTEELFLRPREQTVSENAFARGGIPGVNSGDRGMEFSTFSEQKFRGGLVERIAARAGFNAPRLNTESIRSTDHSLNSEVKSPYLTIPPGLSPTTLLDSPVFLSNSLAQQSPTTGKFPFLPNVCISRSSTMMSEANNKGNNNLFDDNNTSFAFRPSVESGSSFFLNAASKTASATILPQSCPRIEVPVPRSENSFQSHRVEPSLSLPQNRIGHHPQVGLSTTYVEKDNGGKAVSEEQRPFDSLGGGSGGSGEHSSPLDEQLDEGEQRGSGDSMAGGACGTPSEDGYNWRKYGQKQVKGSEYPRSYYKCTHPNCQVKKKVERSHEGHITEIIYKGTHNHPKPSPNRRGAIGSSDSHMNMQLDIPAQAGQQSADVPLWEDSQKRVPSGAPDWMHENLEVTSSASLGPEYGNQPNSLQAQNGSHIETVEAIDASSTFSNDEDEDDRGTHGRNSMPM

>CsWRKY47

MSGATRAIREPRVVVQTTSEVDILDDGYRWRKYGQKVVKGNPNPRSYYKCTNPGCTVRKHVERASHDLKSVITTYEGKHNHDVPAARNSSHISSGTSSPVTGQNSTAAIQTHVHRPGPSQPQNTIPRFERPAFGFAGRQQMGPAHGFAFGMNQPGLGNLTMAAVGQPKLPVLPMHAYLGQAHHVNEMGFLLPKGEPNVEPTSDLGFSSGSTVYQQIMSRLPLGPEM

>CsWRKY48

MDDGYKWRKYGKKSVKNSPNPRNYYKCSSEGCNVKKKVERDREDANYVITTYEGIHNHESPFVVYYNQLPSFTSASTPT

>CsWRKY49

MGSKSQVMLNPQALLEDHQEVTPNSQMGFFNFPSNLTFFQLPSIPQTHSPSPSFDPPNFSTSNNNTNNNNNSNNLSETLLSSSILPLKSSISYELAPQHLLSLQTSTPNLWPWGEIGERLLMNGKRSNNNENYNNQLGVSKMKMKKMKGRRKVREPRFSFKTMSDVDVLDDGYKWRKYGQKVVKNTQHPRSYYRCTQDHCRVKKRVERLAEDPRMVITTYEGRHVHSPSHDSEDSEAQTHLNNFFW

>CsWRKY50

MTKSEVDHLDDGYRWRKYGQKAVKNSPYPRSYYRCTTAGCGVKKRVERSSDDPSIVVTTYEGQHTHQSPIMPRGALSSTAFTPSPQQQPPLVFSQPQQLYRNQFTYAPAPPADVVTCGGGFGHVFHSFGEERRLIDGRTTTTTSTTTTDSFQDHGLLQDMIVPFPEEKEKKVN

>CsWRKY51

MEFFFSATLDDRSGNGRRTQPDLAVVAMGNPPLSPPLFSPSSFFTIPPGISPTQLLDSPLLLNSSRILLSPSTGDLRKSASNCSGHHQQNVKQEHNNITKFSFPPNHTTKSSSSSSIFQSSSTSEVQTQAWGLEWENDDRGDGWGMRNRSGSEDGFNWRKYGQKVVKGSENPRSYYKCTFPNCPVRKQVERSLNNNGQITEIVYKSKHNHPKPDFTRRSSSSFSSSSSSLCNHAGELFNHHWG

>CsWRKY52

MIRGGGGVKTMREERIVVQTISNVDKLDDGYWWRKYGQKVVKGNPNPRSYYKCTYPGCGVRKHIERASHDFRAVVTTYEGKHNHDIPTARAGKPILSNQQGRNNEVVSSSIGGDPRSSSKICSGTNGLLRLTNMLWNNKEEVVDNIYELL

>CsWRKY53

MYESPSSFNGGSPRSEDSDREFKDPFDLTNANSFRKRNILPTWTQKFQVSPGMAIEGSLDDGFAWRKYGQKGILGAKHPRGYYRCTHRNLQGCLATKQVQRSDDDPTIFEITYRGKHSCSQVSNLSTPCTTTPEFQQQNQGVVVELTDQKKAHNQQTAPDALLDSWSSLRVITQNLDTTPLSYDRVEFASASTVDVNFAEFSSFLSPTTSGSGLSYFSASSSGLSEGFVGNQNLNNLQPNNCEIFSSPTSALNTQTTTALDFSFGELQMEPTFSFDNTDFFS

>CsWRKY54

MDPTDSDLPDPSNASSGAKYKLLSPAKLPISRSPCITIPPGLSPTSFLDSPVEPSPTTGSFTKLPMAHDSSGSAIYPMTSMAFSNTNASDEGRSNYFEFKPYVGPNMVPADLSHRKGEQFSEVQGQPQPFTAPPMTKMEISVMSNDLSRSTQMDTHTVASGVSVPEANGDEINHSLNTNSRVQAPQSDPKGSGIPVVSDRLSDDGYNWRKYGQKHVKGSEFPRSYYKCTHPNCEVKKLFERSHDGQITDIIYKGTHDHPKPQPSRRYSASASMNVQEDGTDKPSSLPGQDDRSCSMYAQTMHTIEPNGTTDPSMPANDRITEGAGTTLPCKNHDEVDDDDIYLKRRKMELGGFDVCPMVKPIREPRVVVQTLSEVDILDDGYRWRKYGQKVVRGNPNPRSYYKCTNVGCPVRKHVERASHDPKAVITTYEGKHNHDVPTAKTSSHDVTGPSTIPSSRYRLEESDTISLDLGVGIGTGGENRSNEYRQALHSQLVENRAPSGNFNFEVVQENSAPTYFGVLNRSVIN

>CsWRKY55

MESGWSWDQKSLIGELIQGMELTKQLRAELGSASGEESKGSLVQGILSSYEKALLILKWNGPMNQLQMVEATPGLPSSPISVNGSPSSDDSGRVLKDPQDSRKESKKRKTQPRWTEQVKVNSETGFEGPHEDGYSWRKYGQKDILGATYPRSYYRCTFRNTQNCWAVKQVQRSDEDPSVFEITYRGKHTCSQGNYLAQTCHSPDKQEQKETDGDHHELQPLQENLFGNQTIQNIEKLENKASTFCFGSSSTSVGCKDIVNAGFSSLAIDTHSALGTFPQSFTSPTSPDKNYFTPSPCQRSNVGGTHSVQNLDPDVHEIFSANTSATNSPILDWDFPFDSDHINPNFPFNSQGFFY

>CsWRKY56

MEKKFEEESRMGFDDSAGFYSPAVFSDDFPSSNFDSFSSIFDMPCDAHKASNFDFYYYNNNNSNSNNNPSSFFDLLSTAAPPLSSPASTVPESSEVVNAPTTPNSSSVSSSSNEAAAIEEVNNSTTTHDKPSASKVLKPIKKNQKKQREPRFAFMTKSDIDHLDDGYRWRKYGQKAVKNSPYPRSYYRCTTAGCGVKKRVERSSGDHTIVVTTYEGQHTHQSPIMPRGSLRVLPESTNNSLTVDHDTTATGLLFQHNTSQPFMYIAPPPPFLTINSSSVAASHNPPPISFQPPSPQASVRDHGLLQDLVPLQMRKEPKDEQNG

>ClWRKY1

MEAAAAPFGCPRPVVKTEKPAGDGDNNQDSPSKQHLLVKRGNYAKQEHEAEDKSNSSDQKDLSCTNLQEKQLESARAEMGEVREENQRLKKSLNQIMKDYEALKMQFLGIVGQESKKFPNQDDMSNKEQQHDQIELVSLTLGRFPVAEKKKTADEKNSVNIIIGGNDEEAAVKEALSLGLNCKFKHEESIMTVAVKDVDSPKSFDHEMREEAGETNWSSTKGAKTIRSVEDDVTPQNPPKRARVCVRARCETATMNDGCQWRKYGQKIAKGNPCPRAYYRCTGSPTCLVRKQVQRCADDMSILITTYEGNHNHPLPASANAMASTTSAAASMLLSGSSTSTAASSSSTNTNNLQGLNFYTNNSKPNFYLPNNSSIISSTSPTHPTITLDLTSNPSSSSSNCSTHFGKFTSSFPNSRYPFTSQLDFGSSRNNVLSWNNGLLSYNRNNHQTTTTTTNNIYQNYIQQRNPATSLPHQQPPLPDTIAAATKAITADPSFQSALAAALTSIISTGGASAGPTKSTSARGEQSLFQLMAATTTNKGNGCGTSFLNNITTSTTTSNSPPTGNMVFVPASSLPFSSSKSASASPGDHIDLTN

>ClWRKY2

MSDEMFKDLFYGGMDEYESLVRAFGESSDYSNNNNEISGTPINSCGSLSSSDAGAEEDDSVKDKDKQIDKEHVEDGGESSKAGSGKSKKKGEKKEREARVAFMTKSEVDHLEDGYRWRKYGQKAVKNSAYPRSYYRCTTQKCGVKKRVERSFEDPSIVITTYEGQHNHPVPTTLRGNLSAASSAFSPSMLAPMPVVGGIGFLPTELLSNSSSNNNQAINGGASVYSHNNFDYTYNERQSEYGLLQDIFPTPSSFFNRQL

>ClWRKY3

MLLLFEMENYEEGDLTDIVRGGSSRSRSTTRSSTSHCKTELGDQDEDQDSPFYSSSQDNNYNPQWSSSQFSSQLLLLQDHHHHHHHQYSFGDPFCSTVAASLDLHQELDNTNNNTFFNGITTTTTTSTQDELIKTPSSSNIFSRMLQISPSPDKFPTISSLNSSSNFLIPNHSLNSPTTPHSDHLHHFLHHNDNNPSPSALHISSPRNPPGIKRRKSQARKVVCVPAPVAASSRPNGEVIPSDLWAWRKYGQKPIKGSPYPRGYYRCSSSKGCSARKQVERSRTDPNMLVITYTSEHNHPWPTQRNALAGSSRSQQSKNNNITTTTSSTSTTTPTPNSSKLSQDHHHKNKEEKEEHQDQNNGTLLSAAATTTSNNNNNVKEEETENHHHQSMMSEGFDDDHDFFADLEELETDPLTLLFTTQQPLKLDQIKESATTTPTTTTGCLHDVVPFNNLFDWPPPPLNNNSTPFHEQPPPTTPTNGGFY

>ClWRKY4

MAVEIKSHPYPLLTMEANAVQEAASGLESVEKLIRLLSNANAPQPHSLPSSTQSPIDFPTDCRAAADVAVSKFKKVISLLGRSRLGHARFRRAPLPQQPHYATPIQQIPPHPHLDNNHNNNNDSVNFSAHNSFISSLTGDADTKHPSSSSSPFLITNLSQVSSVGKPPLSTSSLKRKCSSDNLGSGKCAAASSSARCHCSKKRKLRVKRVVRVPAISLKMADIPPDDYSWRKYGQKPIKGSPHPRGYYKCSSVRGCPARKHVERAVDDPTMLVVTYEGEHNHTLSLPETSSLILESS

>ClWRKY5

MDKGWGLTLRDSDQSIGFFSNKPPPPPTTVNSFQRMFQGLEFPGKLGRTDDTDATPSLADENRLPVNEVDFFSDKKRVVDDREDQDSKPSTNIIATTAINKDDKPFTAPRTSFNLVNTGLHLLTANTGSDQSTVDDGISSDGEDKRAKNELAQLQVELQRMNAENHKLRDMLSHVSNNYSSLQMHLLTLMQQQQQNQASEPAHEREIGEKKSTEIKHEVGRVMVPRQFMDLGPSGNNNNNNTGESDELLCNSSSDERTRSGSPLNNNNTTETASKKRDHAEITAPSDHENSKRPNPREESPESESQGWGGPNHKPPRFNSSKPLDQSTEATMRKARVSVRARSEAPMISDGCQWRKYGQKMAKGNPCPRAYYRCTMAVGCPVRKQVQRCAEDRTILITTYEGNHNHPLPPAAMAMASTTTAAATMLLSGSMSSADHNLMNPNLLARAILPCSSSMATISASAPFPTITLDLTHTPNPLQFQRPAATPFHVPFPGGQPPSAAVAAQLPQVLGQALYNNQSKFSGLQLSHEMGANSSHLGHHQITQPATPAQPGGASFADTLSAATAAITADPNFTAALAAAISSIIGGAHSNNNSNTNTTNNTTTTNNNNGSSNNSKISSFPGN

>ClWRKY6

MLRSSLDLGIRPHLGGFVVRARDQVVMGRTDDNVAIIGDWVPPSPSPRTFFSAMQMLGEDIGSRPAMDTTSSDHKTEELFLRPREHTVSENAVARGGIPGANSGDRATEFGTFSEQKFRGGLVERIAARAGFNAPRLNTESIRSTDHSLNSEVKSPYLTIPPGLSPTTLLDSPVFLSNSLAQQSPTTGKFQFLANVSNNRSSTMMSEANNNPFDDNSTSFAFRPSVESGSSFILSAAASKTASATILPQSCPRIEVPVPRSENSFQSHLVEPSLSLPQNRIGHHPQVGLSTSYMEKDDVGKTVSDDQRPFDSLCGGGEHSPPLDEQPDEGEQRGSGDSMAGSGCGAPSEDGYNWRKYGQKQVKGSEYPRSYYKCTHPNCQVKKKVERSHEGHITEIIYKGAHNHPKPSPNRRVAIGSSDSHINMQPDIPAQAAGQQSAEVSVWEDSQKGIPTGAPDWMQENLEVTSSASLGPEYGNQPNPLQAQNSSHIETAEAIDASSTFSNDEDEDDRGTHGSITLGYEGEGDESESKKRKLDAYVTEMSGATRAIREPRVVVQTTSEVDILDDGYRWRKYGQKVVKGNPNPRSYYKCTNPGCTVRKHVERASHDLKSVITTYEGKHNHDVPAARNSSHISSGTSSPVTGQNSTAAMQTHVHRPGPPQPQNTIPRFERPAFGFAGRQQMGTAFGMNQPGLGNLTMAAVGQAKLPVMPMHPYLAQAHHVHEMGFLLPKGEPNVEPTSDLGLNFSNGSTVYQQIMSRLPLGPEM

>ClWRKY7

MDPTDSDLADPSHASSSAGAKYKLMSPAKLPISRSPCITIPPGLSPTSFLDSPVLLTNLKVEPSPTTGSFTKLPMAHDSSSSAIYPVTTMAFSNTNASDEGRSNYFEFKPYVGPNMVPADLSHRKCEQSSEVQVQGQPQPFTAPPMTKIEINVVSNDLSRSTQMDTHPVGSGPSAPEADGDDISHSLNTNSRVQAPQSDPKGSGIPVVSDRLSDDGYNWRKYGQKHVKGSEFPRSYYKCTHPNCEVKKLFERSHDGQIADIIYKGTHDHPKPQPSRRYSASASVNVQEDGPDKPSPLTGQDDRLFSMYAQTVHNLEPNGTTEPSLAANDSINEGAGTTLPCKNQDEVDDDDIFSKRRKIELGGFDVCPMVKPIREPRVVVQTLSEVDILDDGYRWRKYGQKVVRGNPNPRSYYKCTNVGCPVRKHVERASHDPKAVITTYEGKHNHDVPTAKTSSHDVGGPSTIAPSRYRLEESDTISLDLGVGIGTGTGGENRSNDYRQALHSQLVENQTPNGNFNFEVVQENSAPTYFGVINRGINQHGSRESLSESHNKEIAPLNHSSHPYPTSIGRILTGP

>ClWRKY8

MAAFSSWLLDSVDTSLDLNTQPLRFSGEAPKERNYMDLERKVSVKEETGALMEELKRVSAENKKLTEMLTIVCENYNNLRGHLMEQMNKNGEKEISSSKKRKSESSNNNNNMAGMNGNSESSSTDEESYKKPKEETINKSAKITRVQVKIGASDSNLVVKDGFQWRKYGQKVTRDNPCPRAYFKCSFAPSCPVKKKVQRSVEDQSVLVATYEGEHNHPHPSQIEPTSGGAAARSVSIAPAVLTAAPGSSSAPAISLDLAKPKPTMEAKTTSNPRFDSPELQQFLVEQMASSLTKDPNFTAALAAAISGKIFPH

>ClWRKY9

MEEIVKKEDTSTSTDGGDGTGALFPFSDNVIPNGFFDFSDAEKCSVGFMELLGLNNCELFKDYCSEAFNPPATPNCSSSVSSASSDVVNDDDPQQQHNPTTKQLKVKKRKEKKEKEARFAFMTKSEVDHLEDGYRWRKYGQKAVKNSPFPRSYYRCTSASCNVKKRVERSFADPTVVVTTYEGQHTHPSPILTRSALAVTIPPPPPTGGPGGGCVGVAAMPWLKATNNAHDGDIIPALSHQYFQNSSFVTPQNSAVSYNRNNPIGAANAAGILQEKRFCNPNATIFLVDHGLLQDVVPPHMLKQE

>ClWRKY10

MGSLFIFSILVLSLIANNLSSLCFFSFKCSTKSIVIAIAIAMELSSDHSLSLKPNPDDEHRLSSQPTPEEAPSKKRKVVQKTVVTVKIGSKKAAIGIGKVKNEGPPPDFWSWRKYGQKPIKGSPYPRGYYRCSTTKGCSAKKQVERCKTDGSMFIITYTSSHNHPGPNISTLNLDQNQEEIQPQPLDQDDDQDLVPNQGLEKQDQDHNNDEKNSIISPGEEEEEEEEEIEVEEEEEEEESLLAMEDEEKKGIEKVKECLEEPISSSCCHELINLSATTNKSELENHDHFFDELEELPTPPPFSSYFFDEIRISAAPS

>ClWRKY11

MDNKAAERVVIARPVASRPTCSSFKSFSDILACAFNTSPPNMSSETKVAAIRPKTVRFKLKDKYFQAKISETVSGMNSCSSSENLAISDSKTTVLFKPLAKHVSKRTVSQLSLIGNTNLQNRLPHPPVEVCIQCPNQGEDNFESALTSNLCIQCLNQDKDNFQSALTSNLPQNITSTVENSQSIESSRVTLSYSKEDPTLLRPQITCAQPSYDGYNWRKYGQKQVKGSEYPRSYYKCTHPSCPVKKKVERSLDGKVAEIVYKGEHNHPKPQPLKHNSSGTQTEGSVSNGTTRDTNPELWLNYLNGQIEGCESRLENHVEKTCQGRVTLPFDSVATREVQGGCGISDNSCGLSIECEEGSKGLDSTDDKLQSKRRGGKNPTNEAVTSIEDVNEHHTMARGSTGIEISGKGIRWRKYGQKVVKDSISHKLSFCRSYYRCTGLKCKARKYVERASEDPDSFITTYEGKHNHGISLGTENPVAPEME

>ClWRKY12

MNNINQTINTLAGGSSDNRTNNFGMEVPKFKSLQPPPFPMSPSSYLSAFSSGLSPTELLNSPLLFSFGVFPSPTSGALNLRNDYEEGEQQEMKADVKNYSASSFNPQTGSSVSSYFQSSSSNVTPLNPNGLSCDESGAKSEFVTTEMAAAESKPISQLPIYNREQKKSENDGYNWRKYGQKQVKGSENPRSYYKCTFPSCPTKKKVERSLDGQITEIVYKGSHNHAKPQPTRRSGNSAIYDPSSADSALLQEDSSVSVGDEEFEPNSPFSNSVDDNENEPEAKRWKGENENEGYCEGGSRTVKEPRIVVQTTSEIDILPDGYRWRKYGQKVVKGNPNPRSYYKCTSLGCPVRKHIERAANDMRAVITTYEGKHNHEVPAARGSGGSGYNAINRPTPTNIPMPVRPSAIGSHSFPANFPATFQPANLAMSEIGAEASSFPFQTSQGVPPSFQVSGFGSAAKEEARDDIYFINSFLS

>ClWRKY13

MDCSWPDTTPSDRRKAADELLRGRELAQQLRAYLQRTSNSGGTASQDLLSRILTSFSKTLSILNRCDSDDINGSIVDSPEDRASRKSQESGDSCKSSDRRGCYKRRKSCQSWARESCSLVDDGHAWRKYGQKTILNAKYPRNYYRCTHKFDQACQATKQVQRLQDHPPKFRTTYYGHHTCSNFLKASDIVLGSSNFDDSCGVLLSFDTPAAPNFLLQQDAMLVKKEVAIAESRDDEAVCSPSDYISTAEPSPDDHLSEVFMGSVVDFEDDVLQFQF

>ClWRKY14

MDHQFQEESSVKKTSSGSMKSEKKIRKGRFAFETRSQVDVLDDGYRWRKYGQKAVKNDKFPRSYYKCSHQGCKVKKQIQRLTNDEGVVLTTYEGVHSHPIETPQDNFQHILTHMQIYPSSF

>ClWRKY15

MAKKDDSARPPLQRPTITLPPRPSMEAFFAGGPTGVSPGPMTLLSSYFADGAVDSPSFSQLLAGAMASPMAMGFFGTGSTPNYYAKDGTASELEFGMKQSKPLNLVVARPPLFSVPPGLSPSGLLNSPGFYPPQSPFGMSHQQALAQVTAQAALANSHMHMQQAEYQHSSVPAPTEPLTRDPSFSLDEASQLAILPSTSDTKSLIAEPTEVSHPDRKYQPPPPHVSDKPADDGYNWRKYGQKLVKGSEYPRSYYKCTHLNCPVKKKIERSPDGQITEIIYKGQHNHEPPPANKRARDNSEPSGCTNSLMKPECGLQNQAGILNKSSENVQLGSSDSEERADTEITDDRDEDEPNPKRQNIDAGTSGVALSHKTLTEPKIIVQTRSEVDLLDDGYRWRKYGQKVVKGNPNPRSYYKCTSAGCNVRKHVERSSTDSKAVVTTYEGKHNHDVPAARNSSHHTVNNTVPQIKPHKVVAQKHPLLKEMEYGTNDQRPAVLRLKEEQITV

>ClWRKY16

MAVELLTAFTNAQLSSPMDQDSAVQEAASGLDTLKKLITLLSHSHPSNLDSDCQAVANAAVSHFRKAISLLGRTSRTGHARFRRAPLDSSKIYNATPIQQIPPPLDRLDSATTINFSYSAAPSSSFLTSLPGSDSEIKLQHQPSSSSFQITDLSRVSSVVSKPSSGLKRKCGSENLGSGKCAGSSGGRCHCSKKRKMRLKRVVRVPAISSKNADIPPDDYSWRKYGQKPIKGSPYPRGYYKCSSLRGCPARKHVERASDDPSMLIVTYEGDHNHSQSVAEASSLILESW

>ClWRKY17

MDAAAPSPSPPLPTPTPLQFPVNLNSTLPDPNYSPPPPPPPPPPPPPPPPPSSSHRPFFDEMNFFPADDKSRVLLSASHSNLTPTKLDFNVNTGLNLLTTNSCSDQSMVDDGVSPNPEEKRIKNERAVLQAELERINSENLRLKDMLNQVTSNYQTLQMQFNTLIQTQKTGDAGDPIEENAGSGGGNNNNNNNNNHNNIGNKLVPRQFMDLGLATNMENDEPSMSSSEGRSGDRSRSPGTTGEVASSKRHSPDQSSNWGSNNNNSNNNNKVPKFSSSSGKDVDQTEATMRKARVSVRARSEAPMITDGCQWRKYGQKMAKGNPCPRAYYRCTMAAGCPVRKQVQRCAEDKTILITTYEGNHNHPLPPAAMAMASTTSSAARMLLSGSMSSADGLMNSNFLARTLLPCSSSMATISASAPFPTVTLDLTQNPNPLFQRPAAGHFPIPFAAGPPQSFPQIFGHALYNQSKFSGLQMSKDIEAPPPPPPQNPLADTLSAAGVAIASDPNFIAALATAMTSLIGGSHHQNENGNGSSNVDNNTNSNSQQ

>ClWRKY18

MGSKSQVLLNPQALFEDHQDHEVAANSQMGFFNFPSNLTFLQLPSIPQTHSPSPPFDPPNFSISSNNTNNNNNNSNSNLNETLLSSSLLPLKPSLSYEFAPQHLLSLQTSTPNLWPWGEIGEKLMNGKRSNNENHQLGVSTMKMKKMKGRRKVREPRFSFKTMSDVDVLDDGYKWRKYGQKVVKNTQHPRSYYRCTQDHCRVKKRVERLAEDPRMVITTYEGRHVHSPSHDSEDSEAQTHLNNFFW

>ClWRKY19

MVMEFLFSSNSVEDPSGGPTFGHRRLPEHVAAAMSKPPLLPPLSPPLYSPSSFFTIPPGISPTQFLHSPLLLNSPHILPSPSTGAISAGDLTGTSNNSGHHQQNIKQEHNNFTELSFPRTHHTTRSSSSSMFQPSSTVQSQRWGCEAAMNGGSEAEQRAEWELRKRGTEDGFNWRKYGQKVVKGSENPRSYYKCTYPNCPVRKQVERSLNGQITEIVYKSKHNHPKPDFTRRSSSSFSSSSSSSSSSLLSEALPAAMAAEPKNSIASDSLTTPEDSSITIGDDDDDSHGADAKRWKSESEKEIMSSVGGKTVREQRVVVQTISNVDKLDDGYWWRKYGQKVVKGNSNPRSYYKCTYAGCGVRKHIERASHDIRAVITTYEGKHNHEVPVARGSGGGRPILISSEGSNNDVASNGGGNRRPSSEICSGNNGLWELTNMVWRSKEEAVDDIFMGCY

>ClWRKY20

MSNEDHHEKEEDPYYSHFDPFTHNFHHHKPFEAPPLTSPYYEAFDIVPSSVGLYSDFLHASAEYGYNNNNGLLPPAHEMSCSSSDVISPIDDASKKSLGLGDQLVVTGEHPSTPNCSSTTCSSDEAAAGGEDSSKIGEVKGFDDKNGENSKKVNRGKKKEKREKGPRFAFLTKTEIDNLEDGYRWRKYGQKAVKNSPFPSVKKRVERSSEDPCFVITTYEGKHNHYCPITLRGHNPAGVLPPSVSPPYLSGLAPANFFSGEFYENLQQQYYEVPDEYGLLQDLINPSFNSKQDPSN

>ClWRKY21

MVSSGDQLENEVDSDQLEHENSSESQPQTSQDDPGGTNASKSDHKCTGAASITLEEAVKQPEVTIEQVDRGEISNIVTEKVTHKPITAEQNSHSDLKVCITSTIREKVSEDGYNWRKYGQKLVKGNVFVRSYYRCTHPTCMVKKQLERTHDGKITDTVYFGQHDHPKAQPHIPVAVGVVTMVEEKLDEHASGNSQDKTSIAPGQTPHQTELADMRQPSSVMASDDVKDEASKRSRINDEVDSDDTPDLKREKKRCNIDVTVADKSTVESRVVVQTPSEVDIVNDGYRWRKYGQKLVKGNPNPRSYYRCSSPGCPVKKHVERASYDPKVVLTTYEGQHDHDMPPTRTVTLNSVGSTAAHSDETKPKPVGSSIGHDTVVHATKDSLNNSSSEGKLIEKNGKSNATEASDGIVLDMVVNPSPGVASGQNKQLKVAIES

>ClWRKY22

MASSSGSLDTSANSHPSFTFSTHPFMTSSYSDLLASGNNHPPSSAAPHADLRGSGTGVPKFKSLPPPSLPLSPPPLSPSSFFAIPPGLSPAELLDSPVLLSASHVLPSPTTGSFPSQSLNWKSNSGYNQQSIKEENKYLSNFSFQTQSSKLPPTSFQPSSTTAPTTQGWTFQEQRKKEDGFSSEKNMVKPEFGSMRSFSPEYGVVQNQNQTNGSGELQSDYGNNYPQQSQTVNRRSDDGYNWRKYGQKQVKGSENPRSYYKCTFPNCPTKKKVERSLDGQITEIVYKGSHNHPKPQSTRRSSLSSAGSSHTMVASNQATSEMADQSFTTQGSGQFDGVATPENSSISIGDDDFDRSSQKSKSGGDDFDEDEPEAKRWRREGDNNEGISAVGSRTVREPRVVVQTTSDIDILDDGYRWRKYGQKVVKGNPNPRSYYKCTNPGCPVRKHVERASHDLRAVITTYEGKHNHDVPPARGSGSHSLSRPFPNNEAPATAIRPSALTHQSNNGGHLQGLRLQQSSDSQAAFTVEMVQNGNGFSFPEFGNSMGMGMGMGSYINQTQPSDNLFTRAKEEPRDHDMFIQSLLC

>ClWRKY23

MVEENGDVPLVMEEASGDEASQEESFDITVIAVSEISELGDIIEESIDIIDIPGTVEEADIDHKAFESDKTLMDFEFLPDFPKEASVLKYEIAPTADNSCFDGKIANGNCENMESCLSSITTNQPCIPEESTKGDDIETQHPLEDEQKGSYIPMGMLRTSEDGYNWRKYGQKQVKGSEYPRSYYKCTHSNCQVKKKVERSLDGQITEIIYKGAHNHARPDPNRRAMLGSMPTPGDTPEIGEGGGNRSKLEAGLAWRNTQYGMKDIKLISDCSVVDGLERTSSVSVVTELAYPLLNPQGKTVGVLESVGTPELSSTLASHDDDDGGGGDDDDLTTQGSISVCTEADDAEPELKRRRKEGSSIETNLASRSVREPRVVVQIETEVDILEDGYRWRKYGQKVVKGNPNPRSYYKCTSAGCLVRKHVERASHDLKCVITTYEGKHNHEVPAARNSGQVNSSNGNAQPSASHVQPNMGLSRNSNVPKSETQIQDLAAQFYPKPEFNHDYQRSGCFDTFTNDIKLGAPSFCQMKFPPLRNTLPYSSFGLNSKHTGTSMSGSLASVVPDFPISLPLNQNLSAAGYDFTNGRPIPPFQVFLAGQQLRDTDRFLTPKQEHDDDNICASFQPVVDSSSGSSSSSVSSVYQQIMGNFP

>ClWRKY24

MDDNVTFNNNNHQFFSADQLEDSDESKPAPDSPPPNSAAAATATKKGRRGMKKKVLSVKINGDNPRNSSGSTTPPSDAWAWRKYGQKPIKGSPYPRAYYRCSSSKGCPARKQVERNRLDPTMLLITYSCEHNHSGPVSRNNNSNHNQTDAVKPDSPETVPVEVEEKFVEIVGEESLITGDEFSWFGEMETTSSTVLESSIFSGRASSGLVDHSISSDVAMLLPMGDDDMDESLFADLGELPECSLVFRRGGGRGLPVDEQPAAAQRRITPWCGTTT

>ClWRKY25

MDNNYQQAGDLTDVIRLSSAAVAGGLSSEFSLDPSSSDRRLWPHLTADESSMNFGDPLLFPTSSARDPFLHPHFSAFNLASDGGSEGLAAVDQPSNLLSHMLQISPSSGDGVISTPPCESFATAVGNSPCSASACSGGVVLPTGGSNPLCLMENSGIQISSPRNSTTKRRKSQVKKVVCIPAPAPANNRSSSGEVVPSDLWAWRKYGQKPIKGSPYPRGYYRCSSSKGCSARKQVERSRTNPNMLVITYTSEHNHPWPTQRNALAGSTRSHPSRTPTTPTARKTSPKHERTNNNQPTTTLKEEEMDADQTTEKAIVTTTNDHQEDGDHQNFPYDLIFTEFADHDNDHDHHHLSADPLNNIIFSSHGFSTGNGEGSKDPFLELYDWADNSNGSLFKEAKGG

>ClWRKY26

MGTAHVKASPHIHHSHLLHFILSSSSSSSSSSSSSSSSSSSSSSSSSSSSSSSSPREPLGAVPWSDTYNTVSDDHLLTLTGLLHDDDDATASSPLSKTSATIQPPSSPCPVVRPTWGRQLRTLRMRSPLPRDRKTCSPTLTFLILGSLNKVEHKYSLRIKSCGGNMVADDGYKWRKYGQKSIKNSPNPRSYYRCSNPRCSAKKQVERSIEDPDTFIITYEGLHLHFAYPFFLMGQSPQVQSPTKKPKTIDPEPEAYKSPTFLAPGPPLPDDPKEATGPQGLLEDMVPWMIRNPSTNHNALSNSSSCSSHRSPPMSPPSPSTCPTFIASCF

>ClWRKY27

MENYQMFFPCSSGGDGGGRLSAYHHADMSSVSGSDIFGNFHGGDMEVSGVLGMKTEMDAAAATAVEADGGGKKKGEKKVRKPRYAFQTRSQVDILDDGYRWRKYGQKAVKNNKFPRSYYRCTHQGCNVKKQVQRLTRDEGVVVTTYEGMHTHSIDKPTDNFEQILSRMQIYSTPF

>ClWRKY28

MENFVERNQNKLKNELLKGKELAKQLQIHLNMRPSASSTAASSSSSSSFSNDNGGELLVQKILCSYEKALSLLNSNGIQISESPSSINGGSPRSEDSDREFKDLFDQTNANSSRRRNILPIWTQKFQVSPGMALEGSLDDGFCWRKYGQKGILGAKHPRGYYRCTYRNLQGCLATKQVQRSDDDPTVFEITYRGKHSCSQVSNLSTPSTTTPKFQQQNQQTSPDALLNSWASLRVITQNLDTTIHETPTLSFHYDAADRVETSTGDVNFAEFSPCSSFMSPTTSGSGLSYWSASSSGLSEGFVGNQKLDLQPNKCEIFSSPTSALDSETPALDFPFGDLQLDPTFTFDDTNFFS

>ClWRKY29

MAVDLMSFPKMDDQMAIQEAASQGLKSMEHLIRLLSHKQPSSHVDCSDLTDATVSKFKKVISLLNRTGHARFRRGPVSSTSSSSCGSSAHLSFPQNQAMNLTPTSFTSPAAVPAPPFTAPASVAQPQAKVVAAAANILQSQPQSMTLDFTRPNILNSNPKGTDLEFSKETFSVSSSSSFMSSAITGDGSVSNGKLGKSIFLAPAPTASGGKPPLSAAPYKKRCHEHDHSEDLSGKFSGSTSASGKCHCSKRRKNRTKKTIRVPAISSKIADIPPDEYSWRKYGQKPIKGSPYPRGYYKCSTMRGCPARKHVERDPNDPAMLIVTYEGEHRHTQSSLPENMAAGVALVFEST

>ClWRKY30

MGDDDEQPPPPPPPPSKSLRPTINLPPRSSMESLFGGGSGLGFGFSPGPMTLVSSFFSDSDDCKSFSQLLAGAMASPVAAVPPSASEFKASTGLLESPELFSPGQGPFGMTHQQALAQVTMQAAEAYSHNQMQAASFSSSVAPSASSSQLLTSLPVEKTKDQPMQPPLQDSAVASKEPSDNSQSEQRLQSSLCNVDRPTDDGYNWRKYGQKQVKGSEFPRSYYKCTHPNCPVKKKVERSLEGQVTEIIYKGEHNHKRPQLNKRAKDVGNSNGYSSIHGNPELSSQVQSGYLNKLNEQTSVSSTAKKDQELSHVTHEQLSGTSDGEGGSEIETGVNRKDEDEPDAKRRNTEVRISEPASSHRTLTESRIIVQTTSEVDLLDDGYRWRKYGQKIVKGNPYPRSYYKCTTPGCNVRKHVERASTDPKAVITTYEGKHNHDVPVGKISSHCSVNSNNPQLKSQNIVTEKKISSNNTDPGNSRQQPAGLLLLKEEQIT

>ClWRKY31

MDRMKEENKVLRKAVEQTMKDYYDLEMKIGIIQQNNNLNNKDSHNFLSFHENENKRHEEPTKQDLELGEMAKKKRRVRSPSKEDEMRESELGLSLGLHTKNNDLDQEDNHRELLQEERREKKKEDSIMSNFYSIQNKPQRPELQAMAPPQNRKARVSVRARCESATMNDGCQWRKYGQKIAKGNPCPRAYYRCTVAPGCPVRKQVQRCLEDMSILITTYEGTHNHPLPVGATAMASTASAAASFMLLDSTNTTNNLSSLPNPQNPNFLNPQNPNILNSSSYSPTNFLQTQNPNQPSTNTFYTPLIPTSSTSNFPQPSNWINTNPTSFYHTNFPLHGRSLRPPDDNKPPFTPESVSAIASDPKFRVAVAAAISSLINKENNNTTTSNSPIEDSSSFGPSKDGKGSSGGGDSDGRNKKWVVESLSSNGN

>ClWRKY32

MAVELMVGFGDATTPTNFTPKMEENAAVSAVQEAASAGIQSVQNFLRLMSHTPNHQHSQDDSSASTTPNAIAGYEAVADSVVSKFKKVISLLDRSRTGHARFRRAPVLTTTPPPPPPPKVKPQHQDPSSSSPISVPQIQVKKQESVSAFKVYCPTPSPVVRLPPLPHNNSHQPSHPPNTFQAQQNTSSVVLKNGSVDRKDATTTINFAASPPISAANSYISSLTGDTESLQPSLSSGFQFTHMSQVSSAGKPPLSSSSLKRKCNSMEDSAMKCGSSSGRCHCSKKRKNRIKRVIRVPAVSSKLADIPPDDYSWRKYGQKPIKGSPHPRGYYKCSSLRGCPARKHVERALDDPTMLIVTYENDHNHAHSTETPAALVLESS

>ClWRKY33

MMDIFLDLNVDPTSSYANSAMDEALHSSGSESSSSTLEQELDRKIQENGKLSQMLRIMYEKYINLQKQVMYLLSHQKQNSEMEGVCSRKRRAEGEEDNYENLEGICSTRDEDFNRWLKRPRLNGNSKVSKVFVQKDASDPSLVVKDGYQWRKYGQKVTRDNPSPRAYFKCSSAPNCPVKKKVQRSLEDPTILVATYEGEHSHASHFQTELSLRSINGGKGSAVPVLATIKPSCATVTLDLIHEDGLFKSPKDYASSESAEAAVWQEFLVQQMASSLKKDPEFAGIVAGAISGKVLGNQTNRE

>ClWRKY34

METGSQRSSGRGQVVPNINYQVQVSFHLSNAPHDPQPIQEMGFVQLEEQSQVLSFLAPNNAAMPPAFTNPTTSAHTSHLLPRPSWSSQQQVGGTVDPKAGNEENCTGSASDGSNSWWRNTNPDKSKVKVRRKLREPRFCFQTRSDVDVLDDGYKWRKYGQKVVKNSLHPRSYYRCTHSNCRVKKRVERLSEDCRMVITTYEGRHNHSPCDDSNSSEHEPFTSF

>ClWRKY35

MEIMEHKNLISQLTQGKELAIQLKTHLHPSSSPQEACLFLTEMIQSSFERALLLLNFNSSNSNNILFPHQIALLEEGQEVEVEESSTKNKRKIGKSSDVLKKRKLLPRWTEEVKVCNGTAPEGPLNDGHSWRKYGQKDIHGANFPRCYYRCTHRNVRGCLATKQVQRSDNDPNIFEVTYRGRHTCSQSTNLGSVVSISTQNQISEETNQIQQNPNSEILFNFGENNFNLKTEDFSNVFLPCSFEGPMMDPAFIGGGSLSATAVSPAASDTAWDWSAYDGGDEGGLQRVQSSGESDMTEIISATTSGTNSPICNGHWDFSLDNIDFDHNFPFDSLDFIS

>ClWRKY36

MEVDWDLHAVVRGYSAVPSAATIIPSSSSSSSSSSSNNSIPFSFTRDLTNQTKSHFFTLQDPFQPPNCNSTEELHELFKPFFPKSQPSPPPPPPPPAPPLLSSPAAKILTHHKQHQSTHLPKQLHSTPVSAPRSKRRKNQLKKVCQVPAESLSSDIWAWRKYGQKPIKGSPYPRGYYRCSSSKGCMARKQVERNRSDPGMFIVTYTAEHNHPAPTHRNSLAGSTRQKPITTTPTASGSEKPDPKQPVCSSEEQSTITESKEEKEELLMAEDEEDDDLGVSDLIVNDDFYVGFEELDSPITDDCFSDEFPTSFDLPWLFNGNPDSEIVKLPC

>ClWRKY37

MEKKFEDSLMGFDNSAGFYSPAVFSDEFPASNFDSFSSIFDMPCDDHKASAAASCSSNSFDLFSSGIEIHDFYNINNNDDNNNNIPSSSSSNFFNLLSTAAPPLSSPASTVPESSEVLNAPPTPNSSSVSSSSNEATAAIQEVDKINNNTEKPSSSKVLKPKKKNQKKQREPRFAFMTKSDIDHLDDGYRWRKYGQKAVKNSPYPRSYYRCTTAGCDVKKRVERSSGDHSIVVTTYEGQHTHQSPVMPRGSIRVLPESTNNSLIADHDTIATGLLFQHNTTQPFICSSPPPPFLTINSSVAAGSSRPAPPPTSFPPPPSQASLVRDHGLLQDLVPLQMRKEPKDEQNG

>ClWRKY38

MGNSGLLFSDAIIPNNNNMFDFPFIDSTDSSSSSSFKPSTFLDLLATQDYTPSLFDLFSPPPPPPPPPPPPPPSSAVPESSEVLNTPPTPNSSSVSCSSTERLLDADDADRDKSPFNKQLKAKKKNQKRGREPRFAFMTKSEVDHLDDGYRWRKYGQKAVKNSPYPRSYYRCTTAGCGVKKRVERSSDDPSVVVTTYEGQHTHQSPIMPRGATSAGAAAFASPQPPLVFPQPQLFRNHHQQYTYAPAPPVDVVTCGRVFDPVFHSFGGDERRRIDGITCSNSDSFQDHGLLQDMIVPSSNSLQIPEEDKQN

>ClWRKY39

MAERESFETDQLGSSKAATAGQEDDEEDMEDSEDESEVELEDGGGGGGSELQPTELRTGSWVSEAVVRGSRSETLTVPSANQSSENGRSDGLPVNSAVQSLEGAELKQAPSSRNEQKAVEATQIDKVQEQNQLQVSTFKGPGSDQSPTSVTQSISSSQSPSLSEHKLSPKKVQKECKPEPSQKNSSNHKTASSVPNVRTPASDGYNWRKYGQKQVKSPKGSRSYYKCTYSECFAKKIECCDHSGHRTGTVYKSEHSHDPPRKISIPKESKLVPYVEPVVKKIIAEHSRRVINDSDPPTPSKEPVRETAIVLERKRQHSSDSDGNDEFKIKDENGDGPETKQKVKKSSAGNSGTPLKPGKKPKFVVHAAGDVGISGDGYRWRKYGQKMVKGNPHPRNYYRCTSAGCPVRKHIESAVENPNAVIITYKGVHDHDMPVPKKRHGPPSAPLVAAAAPASMSNTQPKKTDVVESQISSTQWSVDAEGELTGEALELGGEKAMESARTLLSIGFEIKPC

>ClWRKY40

MAVDLAAFPAKMDDQTAIEEAASAGLQSMEHLILLLSKQQHSQTLNNNLNYSGLTDFTVSKFKRLISLLNRTGHARFRRGPSDSPNPVLNSLDPPLKTHFPKPNFSPASKFPDSKDSTTTSSFVSTITGDGSVSNGKVDLSVFATPPVSGGKPPLALKRKCDDSSGFGCKVPSKLCHCAKRRKSGVKKTVRVPAISSKIADIPSDEYSWRKYGQKPIKGSPYPRGYYRCSTVKGCPARKKVERARDDPTMLLVTYDGDHRHPHPTVTDASVGVVSQKS

>ClWRKY41

MEAEKALPTIDDKVQSSGGGDEEAAPNPKQDVLLKDDQHHEERIKRTRVEMREVREENERLKKSLDEIMKDYETLKRKFHEMNHQITQTNGSTINNIDHEVEEEVDDLVSLTLGRFSTHQNKNTTSSSSLTKLDHQKILDLKQDYIIQTPSIIDRNIQSPTHSEPKEEEAGQTWPPSKMSKPSSLPPPPIGEDEVSQQNPPKKARVCVRARCDTPTMNDGCQWRKYGQKIAKGNPCPRAYYRCTGAPTCPVRKQVQRSVDDISILITTYEGTHNHPLPVSAMAMASTTSAAASMLLSGASSSTSQPGLNPSFTTATAAANLHGMNMYLSNNTNSKQFYLSNSSMLSSSLNHPTITLDLTSNPPSTSSSSSPFHKNIPSINNSYPQKYPFTSLDFGSSQPNFMSWNNNNNSGHQAYSNISKNNAIIGMPSDFAKQLPLHTNIYQAYLQQFSKSSTPPPPPPLPDTIAAATKVITSDPSFQSALAAALSTIIVGGGSGGETGPPTSSFVGGAQAAKSLTCSTSKSPSSSPGDSRENGK

>ClWRKY42

MDRRVRTNPFVSEQEDPEATSDDGSPESPSDGNDSKSASAGPPPKKSRRGVQKRVVSVPIGDVEGSKSKGEAYPPSDPWAWRKYGQKPIKGSPYPRGYYRCSSSKGCPARKQVERSRVDPTKLVITYAFDHNHQLPATKSHHHHHNSSPSSAAIAAASTAADSSSPGSTTTSSSTSSGDNTNAAPSSPAAAAKFEEAAAVFASQPELELGGDSLMIKPCIGDFGWLADVAYDRILEGPICGGGDIFDDADVMVLSTTGDDEEESLFADLGELPEGSVVFGRRRPVQPDGPNRTCGRVLDC

>ClWRKY43

MEEVEEANKSAIESCHGVLNLLVQPPQDQLHFRNLMVETKEAVFKFKKVISLLNSGFGHARVRKFNKIPLPLPQKSLLDSPNYTPHPPNKNLYPFHSGLNARSFISSLSMDGSVADGSSFHLIGPSSTTTTSADNKRKFSARGDEGSLKCGSSGKCHCSKKRKHRVKRSIKVPAISNKLADIPSDDYSWRKYGQKPIKGSPHPRGYYKCSSMRGCPARKHVERCLEDPSMLIVTYEGEHNHPKMSTQSAHT

>ClWRKY44

MEEAISLILRGCSLARQLEFDVLNLGSNNINVPQPHLMARSCDEILGVFSAAKERLSSHEQWPPFTAVQREVGLEEWLRSTCSQAMELAQMQTRSPAPPSAVVAVPVPAAVEDSGKLIAMGFSSSSSSSSTKARRRKDDTEKRTVRVAAPRIGNTELPPDDGFTWRKYGQKEILGSRFPRGYFRCTHQKLYHCPAKKHVQRLDDDPHTFEVTYRGDHTCHMSATAPSAPPPPLPAIAGGQHISQFRPPSTGWLSVEVVGSASASASGGPATVRYGKDVAEQFPVVDLADVMFNSGTGSSNSMDSIFAAYVADDHHHKWEKDDKKN

>ClWRKY45

MEPADFSHAGIPAETRRKITAKLLSGRQSAARLQSLLQSAAAADHDPLALATKILTSFNESISILESAAAELSCPDHSLCSDLDSGDSRGSTAVKNHQGRANKRRRLMNTRVVMTATTEDKYGWRKYGQKVILNATYPRSYFRCTHKYDQGCRATKHVQRMEGMDSEIMYKITYICDHTCSTASQIIASAVASASDSYNLISFSNSHNGQLIEGTGHSFFCPNNDDAMKVRETTTTSGSTNHEVDLWSELKDFGSLQTTTMTNQYYFSTGDDDADSLMFWNGCLQ

>ClWRKY46

MESGWSWDQKSLIGELIQGLELTKRLRAELSSASAEESRGSLVQGILSSYEKALLILKWNGPMNQPQMVEATPGLPGSPISVNGSPSSDDSGRGLKDPQDPRKESKKRKTQPRWTEQVKVNSETGFEGPHEDGYSWRKYGQKDILGATYPRSYYRCTFRNTQNCWAIKQVQRSDEDPSVFEITYRGKHTCSQGNYLAQSCHSPDKQEKKENDHDHGHNHDHHQLQPSQENLFSNQTIENIEKLENKASTFCFGSSSTSVGCKDIVNGGFSHLAIDTHAALGSFTQSFISPTTPDSNYFTPSPCQRSNIGETHNVQHPEPDVHEIFSANTSATNSPILDWDFPFDSEQINPNFPFNSQGFFY

>ClWRKY47

MDGDIMNPQGHLMGTSSLINNCEIDWDGLFSGSSCLSNIEMEKGICGSEDQVIASMGDYDNVVNIGGIKEGDIGGGKNNCNKGKMVMGKRRSAMAPRIAFQTRSAEDVLDDGYRWRKYGQKAVKHSNHPRSYYRCTHHTCNVKKQIQRHAKDPTIVVTTYEGIHNHPSEKLMETLSPLLKQLQFLSGI

>ClWRKY48

MSYNSNQQLTTSESDLSEQPGLEFTDWMFDGWLNENSSSLADSVMYSVYQEGEVDEFVGNTIQQGQPSSRDSGREREVRERFAFKTKSEIEILDDGFKWRKYGKKMVKNSPNPRNYYKCSVEGCPVKKRVERDREDPKYVITTYEGVHTHESS

>ClWRKY49

MESSTTIKTSLDLNFNPPPYTADESPLTHTPSPLKEQAPAILAEKLNRISSENQKLNQMLGLVVENCNVLQHQVIDLMMKSRKRKAAGCDNYCNFNRSGSDQYCGCCSDDNDSCYNKRPRENSKPKVMRVLVPTPVSDSTLVVKDGYQWRKYGQKVTKDNPSPRAYYKCSFAPSCPVKRKVQRSVEDPSYLIATYEGEHNHAKPNSGIEYQLIGPIHLSSNLDSACGVSSSPSSSVKSPSLMTSIVTNSLKASLPQPETPSTTSASASSTQKLLVQQMATLLTKDPNFTRALATAITGNMVDKEIWR

>ClWRKY50

MSNDEGKNVYQQYDPFQYNQLDMNRSIFHQQAATLDPALMSFTNFFDTSLDYNSLSKAFDVSCCSSEVISAVDDRSKSKKASTTTPNSSVSSSSNEAVVEEDSVKSNKEDIKGCENKDEEKSKKQNSITKKKEKRQREPRFAFLTKSEIDHLEDGYRWRKYGQKAVKNSPYPRSYYRCTSQKCVVKKRVERSYQDPSVVITTYEGQHNHHCPATLRGHSAGIMSSPFYASASTSVTAASSGPTLPQELFSHLLPTNNCQTDPASMMYQNLSLQQHLQMPDHYGLLQDLFTQK

>ClWRKY51

MMEEVEEANREAVESCHKVLNLLTVASSSQDQLKLRSRLMAETGDAVFKFRKVLCLLDSSGLGHARVRKKKVKNFLFNSSSSPFPLPQSLFLETSSPNCRMDLLQGRNLQMGPLCLGNPSLELNSNAKSCSTQQIQSQSAALYHHHHHHHLLQNRVLLNNNPNPNPPQPEMMYLRSNNGINLNFDSSSCTQHTMSSTRSFISSLSIDGSVASLDGSAFHLIGAPRSLPIRIRILRGSAVGEGKMGVLNVEAVGDVTAQKRGWEAVILKHRVKRSIKVPAISNKLADIPPDDYSWRKYGQKPIKGSPHPRGYYKCSSMRGCPARKHVERCLEEPSMLIVTYEGEHNHPRIPSQPANT

>ClWRKY52

MEFPTLIQELNQGKQLANQLRNHLHPSSSSSSSSSSSSSSSSSSSHGILLIDQILRSYENALLALSAGAPSVTSPLAPLNHTDVVSKKRKVMAKWSEQVKVSSSSANDSGPRCDGFSWRKYGQKDILGSKFPRGYFRCSHRFTQGCLATKQVQKSDNDPTVYDITYKGRHTCNRVHSNTPQEDQNSLLQHPMPPKQEQKPLQQPHDPSGFTFSSDAVQVKSENLDDVEGGLFQPFCAPSPMFGSEVQDDQSPFRESEFSPTFESNDMFGFCSDFETGFISIPNSVTNISIGDLEEYCSFDNVELFC

>ClWRKY53

MEEVAAANSLRYPFVDDDENKSCLGFMELLSVDQNFSSQFDVFETLSSLSSSLISNPVVNSENSEIWNQWPTTPTYSSSTSSEILNGEPNQEGGEKQHQQHTVKTNKQLKTKKTSPKKKDQEQRFAFMTRSEVDHLEDGYRWRKYGQKAVKNSPFPRSYYRCTSVACNVKKRVERCLKDPSIVMTTYEGQHTHPSPIMARSTFFPPPISVTLYDDYPYQNGHSSNFISHPKGFVSSSFHQPCGIASSNRATHLLGATIDHGLLQDITPFQYDELAWRNGPI
